# Identification of a novel four-gene diagnostic signature for patients with sepsis by integrating weighted gene co-expression network analysis and support vector machine algorithm

**DOI:** 10.1186/s41065-021-00215-8

**Published:** 2022-02-21

**Authors:** Mingliang Li, He Huang, Chunlian Ke, Lei Tan, Jiezhong Wu, Shilei Xu, Xusheng Tu

**Affiliations:** 1 Department of General ICU, the Third Affiliated Hospital of Sun Yat-Sen University, Guangzhou, China; 2 General Surgery Department, the Third Affiliated Hospital of Sun Yat-sen University, Guangzhou, 510630 Guangdong Province China; 3 Department of Medical Ultrasonic, the Third Affiliated Hospital of Sun Yat-sen University, Guangzhou, China; 4 Department of Emergency Medicine, the Third Affiliated Hospital of Sun Yat-sen University, Guangzhou, 510630 Guangdong Province China

**Keywords:** Diagnostic model, Immune infiltration, sepsis, Hub genes, WGCNA

## Abstract

Sepsis is a life-threatening condition in which the immune response is directed towards the host tissues, causing organ failure. Since sepsis does not present with specific symptoms, its diagnosis is often delayed. The lack of diagnostic accuracy results in a non-specific diagnosis, and to date, a standard diagnostic test to detect sepsis in patients remains lacking. Therefore, it is vital to identify sepsis-related diagnostic genes. This study aimed to conduct an integrated analysis to assess the immune scores of samples from patients diagnosed with sepsis and normal samples, followed by weighted gene co-expression network analysis (WGCNA) to identify immune infiltration-related genes and potential transcriptome markers in sepsis. Furthermore, gene regulatory networks were established to screen diagnostic markers for sepsis based on the protein-protein interaction networks involving these immune infiltration-related genes. Moreover, we integrated WGCNA with the support vector machine (SVM) algorithm to build a diagnostic model for sepsis. Results showed that the immune score was significantly lower in the samples from patients with sepsis than in normal samples. A total of 328 and 333 genes were positively and negatively correlated with the immune score, respectively. Using the MCODE plugin in Cytoscape, we identified four modules, and through functional annotation, we found that these modules were related to the immune response. Gene Ontology functional enrichment analysis showed that the identified genes were associated with functions such as neutrophil degranulation, neutrophil activation in the immune response, neutrophil activation, and neutrophil-mediated immunity. Kyoto Encyclopedia of Genes and Genomes (KEGG) pathway analysis showed the enrichment of pathways such as primary immunodeficiency, Th1- and Th2-cell differentiation, T-cell receptor signaling pathway, and natural killer cell-mediated cytotoxicity. Finally, we identified a four-gene signature, containing the hub genes *LCK*, *CCL5*, *ITGAM*, and *MMP9*, and established a model that could be used to diagnose patients with sepsis.

**Supplementary Information:**

The online version contains supplementary material available at 10.1186/s41065-021-00215-8.

## Introduction

Sepsis refers to a multi-stage developmental process of infection involving a range of conditions from systemic inflammatory response syndrome (SIRS) to septic shock, which can lead to multiple organ dysfunction syndrome (MODS) and even death [1, 2]. Sepsis is commonly observed in the aging population, especially in patients with cancer and immunocompromised individuals [3, 4]. Currently, the treatment of severe sepsis has improved due to early diagnosis, quick recovery, rapid application of effective antibiotics, and improvements in supportive care, including pulmonary protective ventilation, smarter use of blood products, and reduced prevalence of nosocomial infections in critically ill patients [5].

Sepsis is a heterogeneous syndrome, and its development is the pathophysiological evolution of the patient’s body, involving different cell types and molecules, and its clinical manifestations lack specificity, patients often delay timely and effective treatment because of diagnosing late. In the diagnosis of sepsis, biomarkers are still in their infancy, studies by Shang-Kai Hung et al. reviewed a number of promising biomarkers including C-reactive protein (CRP), procalcitonin (PCT), interleukin-6 (IL-6), CD64, presepsin, and sTREM-1, to distinguish between adults with sepsis [6]. Several studies have identified sepsis-related indicators, including markers of the hyperinflammatory stage in sepsis such as pro-inflammatory cytokines and chemokines, proteins synthesized in response to infection and inflammation [7, 8], and markers of neutrophil and monocyte activation [9]. More recently, studies have identified markers of immunosuppression in sepsis, such as anti-inflammatory cytokines, as well as the altered expression of cell surface markers on monocytes and lymphocytes [10, 11]. The identification of a variety of pro-inflammatory and anti-inflammatory markers can help identify patients at risk of severe sepsis, prevent the development of organ dysfunction, and help reduce the mortality rate associated with severe sepsis. Although current evidence has identified several important biomarkers associated with sepsis, it is difficult to make a rapid diagnosis and evaluation of sepsis with single biomarker because of poor sensitivity or specificity. In view of the complex pathophysiological process of sepsis, it is important to look for biomarkers with high sensitivity and specificity for the diagnosis of sepsis and treatment of multiple organ dysfunction syndrome.

The pathogenesis and progression of sepsis are not fully elucidated yet; therefore, it is vital to conduct in-depth studies of the mechanisms of sepsis at the molecular level, especially to identify sepsis-related diagnostic genes. In this study, we performed an integrated analysis using public multiple microarray datasets to assess the immune scores of samples from patients with sepsis and normal samples, followed by weighted gene co-expression network analysis (WGCNA) to immune infiltration-related genes and potential transcriptome markers in sepsis. Furthermore, gene regulatory networks were established to screen sepsis-related diagnostic markers based on protein-protein interaction (PPI) networks involving these immune-infiltration related genes. Finally, we constricted a sepsis diagnostic model based on the support vector machine (SVM) algorithm.2. Materials and methods.

### Data collection and preprocessing

Gene Expression Omnibus (GEO) datasets, including those of normal samples and samples obtained from patients with sepsis (GSE57065 [12], GSE65682 [13], and GSE145227 [14]) were downloaded from the NCBI Gene Expression Omnibus (GEO) database (https://www.ncbi.nlm.nih.gov/geo/). The data were processed via the following steps: 1) Normal samples and samples from patients with sepsis were retained; 2) Probes were transferred to Gene Symbol; 3) Probes with more than one gene were eliminated; 4) The mean expression value was calculated for genes corresponding to multiple gene symbols.

### Analysis of differentially expressed genes (DEGs) in sepsis

The “limma” package in R was used to obtain DEGs between normal samples and samples from patients with sepsis by setting the following thresholds: false discovery rate (FDR) < 0.05 and |log2 fold-change (FC)| > 1. A total of 786 DEGs, including 427 upregulated genes and 359 downregulated genes were acquired.

### Immune infiltration score analysis of data

For the GSE57065 and GSE65682 data sets, we used ESTIMATE software [15]. to evaluate the StromalScore, ImmuneScore and ESTIMATEScore of the samples. MCPCounter [16] was used to evaluate the scores of 10 types of immune cells, and GSVA’s ssGSEA method was performed to evaluate the scores of 28 types of immune cells [17]. To explore the relationship between sepsis and immune infiltration.

### Identification of co-expressed genes in sepsis using WGCNA

The WGCNA algorithm was used to identify co-expressed genes and co-expression modules according to the gene expression profiles of samples in the GSE57065 dataset. First, expression profiles of the DEGs in the GSE57065 dataset were extracted, and Pearson’s correlation analysis was performed to calculate the distances between the sequences of genes. Moreover, gene co-expression networks were constructed using the WGCNA package. The weighted co-expression network was constructed following the scale-free network law, meaning that the logarithm of k of a node with k-connectivity is negatively correlated with the logarithm of P of k of the probability of that node.

Next, the gene expression matrix was transformed into an adjacency matrix, which in turn was transformed into a topological matrix (TOM). The average-linkage clustering was performed with the hierarchical clustering module based on TOM. According to the standard of a hybrid dynamic cut tree, the minimum number of bases was set at 80 for each gene network module.

### Construction of PPI networks using STRING (search tool for the retrieval of interacting genes/proteins)

The STRING database is a database of known PPIs across 2031 species, containing 9.6 million proteins and 13.8 million PPIs [18]. It contains not only results of experimental data, text mined from PubMed abstracts, and integrated data from other databases, but also results predicted by bioinformatics methods. The study of PPI networks is helpful in the identification hub regulatory genes. Currently, there are many databases of PPI networks, among which the STRING database covers the maximum number of species and contains information on different PPIs. The PPI network was visualized using Cytoscape (http://cytoscape.org). Then, the Molecular Complex Detection (MCODE) plugin in Cytoscape was used to identify gene modules. The identified gene modules were subjected to Gene Ontology (GO) and Kyoto Encyclopedia of Genes and Genomes (KEGG) enrichment analysis to study the functions and pathways associated with the identified DEGs.

## Results

### Study workflow

**Fig. 1:**
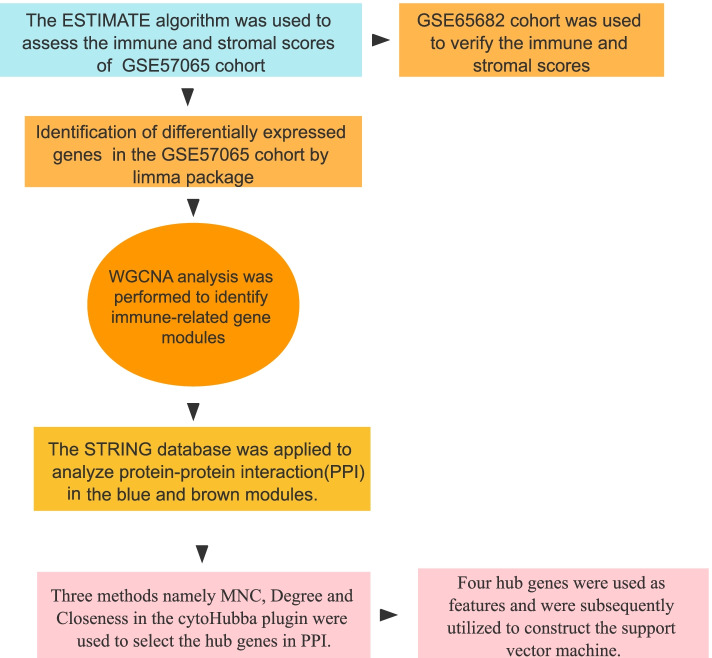
Workflow of the construction of the diagnostic model and immune infiltration analysis in sepsis

The protocol designed to identify the immune infiltration-related genes and construct the diagnostic model for sepsis is displayed in Fig. 1. Specifically, we conducted comprehensive analysis through multiple public microarray data sets to evaluate the immune scores of sepsis and normal samples. And through WGCNA to identify genes related to sepsis immunity to determine potential transcriptome markers. Further, a gene regulatory network based on these immune infiltration-related genes was constructed, and a diagnostic model for predicting sepsis was developed based on the pattern recognition of support vector machines (SVM).

### Data collection

The GSE57065 dataset consisted of 82 samples from patients with sepsis and 25 normal samples; the GSE65682 dataset consisted of 760 samples from patients with samples and 42 normal samples; the GSE145227 dataset consisted of 10 samples from patients with sepsis and 12 normal samples. Patients’ clinical characteristics are provided in Table 1.

**Table 1:** Samples information

Data set	Expression	Platforms
**GSE57065**		
Normal	25	GPL570
Sepsis	82	
**GSE65682**		
Normal	42	GPL13667
Sepsis	760	
**GSE145227**		
Normal	12	GPL23178
Sepsis	10	

Note:

For the GSE57065 cohort, the total RNA of the sample was extracted through Whole blood Paxgene tubes, and Biotinylated cRNA were prepared according to the standard Affymetrix protocol from total RNA (Expression Analysis Technical Manual, 2001, Affymetrix)

For the GSE65682 cohort, whole blood was collected in PAXgene blood RNA tubes, mixed by inversion 10X and stored at -80C.Total RNA was isolated in accordance with the PAXgene blood RNA isolation (QIAGEN) procedure using the QIAcube workstation

For the GSE145227 cohort, total RNA was extracted using RNAiso, The sample labeling, microarray hybridization and washing were performed based on the manufacturer’s standard protocols. Briefly, total RNAs were transcribed to double strand cDNAs and then synthesized cRNAs. Next, 2nd cycle cDNAs were synthesized from cRNAs, followed by fragmentation and biotin labeling

### Analysis of immune infiltration scores

**Fig. 2:**
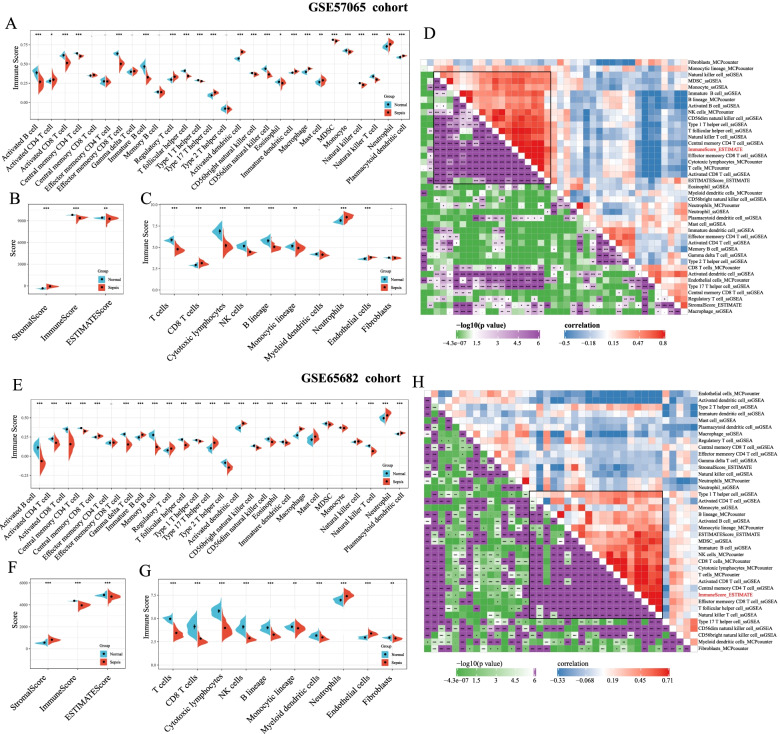
**A** Comparison of single-sample gene set enrichment analysis (ssGSEA) immune scores in samples from patients with sepsis and normal samples from the GSE57065 dataset; **B** Comparison of ESTIMATE immune scores in samples from patients with sepsis and normal samples from the GSE57065 dataset; **C** Comparison of MCPcounter immune scores in samples from patients with sepsis and normal samples from the GSE57065 dataset. **D** Correlation of immune scores evaluated by different software and algorithms in the GSE57065 dataset. **E** Comparison of single-sample gene set enrichment analysis (ssGSEA) immune scores in samples from patients with sepsis and normal samples from the GSE65682 dataset; **F** Comparison of ESTIMATE immune scores in samples from patients with sepsis and normal samples from the GSE65682 dataset; **G** Comparison of MCPcounter immune scores in samples from patients with sepsis and normal samples from the GSE65682 dataset. **H** Correlation of immune scores evaluated by different software and algorithms in the GSE65682 dataset

The ESTIMATE (Estimation of Stromal and Immune cells in malignant Tumours using Expression data) algorithm was used to assess the immune, stromal, and ESTIMATE scores of samples from the GSE57065 dataset. The scores of 28 types of immune cells were estimated by single-sample gene set enrichment analysis (ssGSEA) method using the “GSVA” package in R, and results showed that the abundance of activated B cells, immature B cells, natural killer (NK) cells, NK T cells, and myeloid-derived suppressor cells (MDSCs) was significantly lower in samples from patients with sepsis than in normal samples. However, the abundance of neutrophils was significantly higher in samples from patients with sepsis than in normal samples (Fig. 2A). The immune score and ESTIMATE score were significantly lower in samples from patients with sepsis than in normal samples, and the stromal score was significantly higher in samples from patients with sepsis than in normal samples (Fig. 2B). The “MCPcounter” package in R was used to evaluate the scores of 10 types of immune cells, and results showed that the abundance of T cells, cytotoxic lymphocytes, NK cells, and B-lineage and monocytic-lineage cells was significantly lower in samples from patients with sepsis than in normal samples (Fig. 2C), whereas that of neutrophils and endothelial cells was significantly higher in samples from patients with sepsis than in normal samples. These results indicated that patients with sepsis had abnormal immune disorders and were immunocompromised.

At the same time, Spearman correlation analysis was performed to test the correlation between immune scores and the abundance of immune cells (Fig. 2D). The results reported a significantly positive correlation between the immune score calculated by the ESTIMATE method and the abundance of B-lineage cells, NK cells, cytotoxic lymphocytes, T cells evaluated by the “MCPcounter” package and that of immature B cells, MDSCs, activated B cells, and NK T cells assessed by ssGSEA.

Similarly, the ESTIMATE algorithm was applied to evaluate the immune, stromal and ESTIMATE scores of samples from the GSE65682 dataset. The scores of 28 types of immune cells estimated by ssGSEA showed that the abundance of activated B cells, immature B cells, NK cells, NK T cells, and MDSCs were significantly lower in samples from patients with sepsis than in normal samples, whereas that of neutrophils was significantly higher in samples from patients with sepsis than in normal samples (Fig. 2E). The immune score and ESTIMATE score were significantly lower in samples from patients with sepsis than in normal samples, whereas the stromal score was significantly higher in samples from patients with sepsis than in normal samples (Fig. 2F). The scores of 10 types of immune cells evaluated by the “MCPcounter” package in R showed that the abundance of T cells, cytotoxic lymphocytes, NK cells, and B-lineage and monocytic-lineage cells was significantly lower in samples from patients with sepsis than in normal samples (Fig. 2G), while that of neutrophils and endothelial cells was significantly higher in samples from patients with sepsis than in normal samples. These results were consistent with those obtained from samples in the GSE57065 dataset.

Then, Spearman correlation analysis was performed to test the correlation between immune scores and the abundance of immune cells (Fig. 2H). The results reported a significantly positive correlation between the immune score calculated by the ESTIMATE method and the abundance of lineage-lineage cells, NK cells, cytotoxic lymphocytes, and T cells evaluated by the “MCPcounter” package in R and that of immature B cells, MDSCs, activated B cells, and NK T cells assessed by ssGSEA, which were consistent with the immunization rating scale of the GSE65682 dataset.

### Identification of DEGs in the GSE57065 dataset

**Fig. 3:**
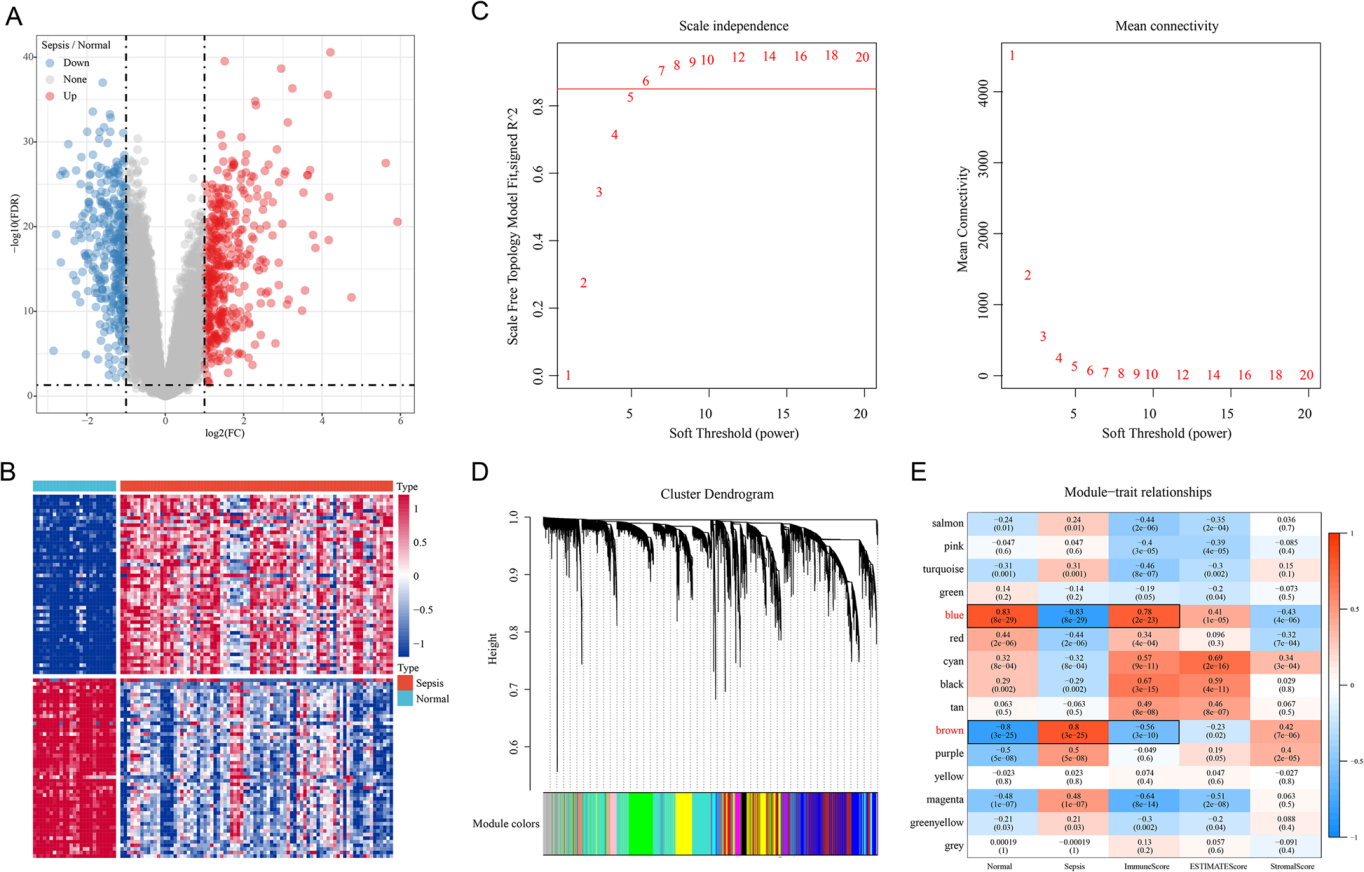
**A** Volcano plot of the differentially expressed genes (DEGs) in the GSE57065 dataset. **B** Heat map of the DEGs in the GSE57065 dataset. **C** Analysis of network topology for various soft-thresholding powers; **D** Gene dendrogram and module colors; **E** Correlation between the expression profiles of 15 gene modules and immune scores

A total of 786 DEGs, including 427 upregulated genes and 359 downregulated genes, were obtained (Supplementary Table 1). A heatmap and a volcano plot for the top 50 upregulated or downregulated genes are shown in Fig. 3A-B.

### WGCNA analysis of GSE57065 dataset

Gene co-expression networks were constructed using the WGCNA package. The power of β = 6 (Fig. 3C) was selected as the soft-thresholding to ensure a scale-free network. After identifying gene modules by the dynamic shearing method, the eigenvectors of each module were calculated, modules were clustered, and nearer modules were merged into new modules by setting the following parameters: height = 0.25; deepSplit = 2; minModuleSize = 80. Finally, 15 modules were obtained (Fig. 3D).

The correlations between each gene module within samples from patients with sepsis and normal samples and the immune Score, stromal score, and ESTIMATE score were further analyzed, as shown in Fig. 3E. The blue module had the most significant negative correlation with scores of samples from patients with sepsis and the most significant positive correlation with scores of normal samples. The blue module had a significant positive correlation with the immune score. In addition, the brown module had the most significant positive correlation with scores of samples from patients with sepsis and the most significant negative correlation with scores of normal samples. The brown module had a significant negative correlation with the immune score. A total of 3301 genes contained in the blue module and 3023 genes contained in the brown module are shown in Supplementary Table 2.

### Functional annotation analysis of DEGs in the blue and brown co-expression module

**Fig. 4:**
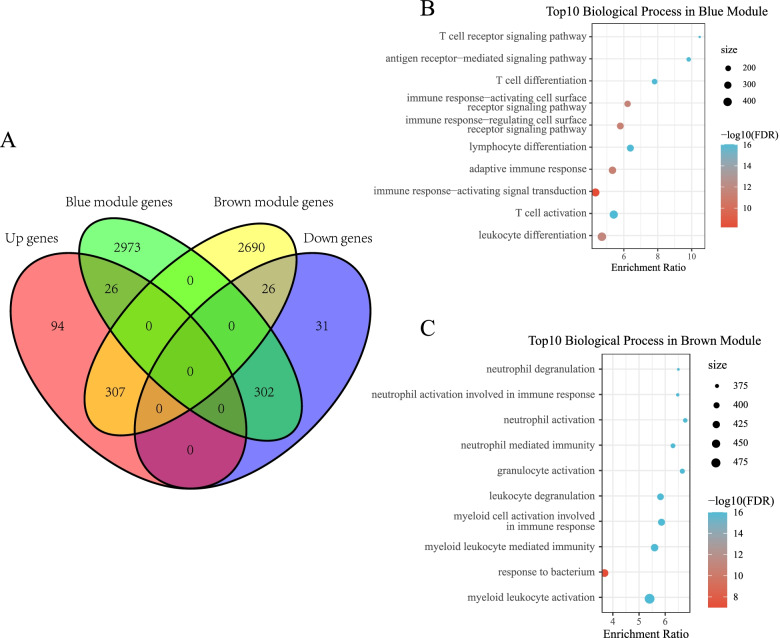
**A**Venn diagram showing the intersection of co-expressed genes and differentially expressed genes. **B** Gene Ontology (GO) analysis of differentially expressed genes (DEGs) in the blue co-expression module. **C** Biological process (BP) annotation of DEGs in the brown module

Then, we extracted the genes from the blue and brown modules that intersected with co-expressed DEGs in the GSE57065 data set. We obtained a total of 328 intersected genes (Supplementary Table 3), which included 26 intersected genes between the blue module genes and the upregulated DEGs and 302 intersected genes between the blue module genes and the downregulated DEGs. Simultaneously, there were a total of 333 intersected genes (Supplementary Table 4), which included 307 intersected genes between the blue module genes and upregulated DEGs and 26 intersected genes between the blue module genes and the downregulated DEGs (Fig. 4A). Further, the “WebGestaltR” (V0.4.2) package in R was used to perform KEGG analysis and GO functional enrichment analysis on 328 genes in the blue module that were positively related to the immune score. GO analysis was performed for functional annotation of biological process (BP). Ninety GO terms with a significant difference in BP between samples from patients with sepsis and normal samples were annotated (FDR < 0.05). The top 10 significantly enriched terms included immune biological processes such as T-cell receptor signaling pathway, adaptive immune response, and T-cell activation (Fig. 4B).

Similarly, GO functional enrichment analysis was performed on 333 genes in the brown module that were negatively related to the immune score. Eighty seven GO terms with a significant difference in BP between samples from patients with sepsis and normal samples were annotated (FDR < 0.05). The top 10 terms included neutrophil-related biological processes such as neutrophil degranulation, neutrophil activation involved in immune response, neutrophil activation, and neutrophil-mediated immunity (Fig. 4C).

### Analysis of PPI networks in co-expressed genes

**Fig. 5:**
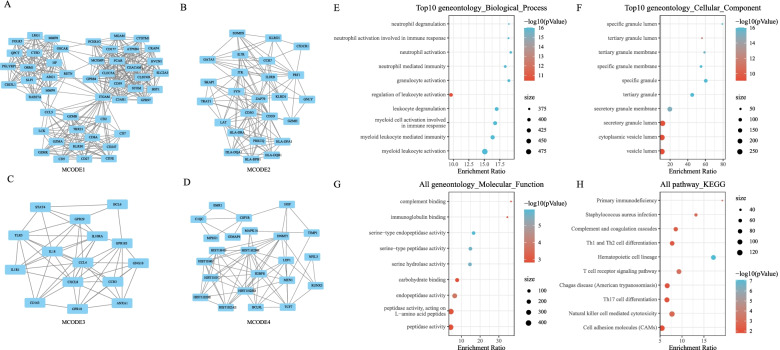
Identification of modules from the analysis of protein-protein interaction (PPI) networks using the Molecular Complex Detection (MCODE) plugin in Cytoscape. **A-D** Four modules, namely MCODE1, MCODE2, MCODE3, and MCODE4, were obtained. **E** Bubble chart of biological process (BP) function of MCODE1 module genes. **F** Bubble chart of cellular component (CC) function of MCODE1 module genes. **G** Bubble chart of molecular function (MF) function of MCODE1 module genes. **H** Bubble chart of KEGG pathway of MCODE1 module genes

The STRING database was applied to analyze PPI of 661 co-expressed DEGs in the blue and brown modules. The Molecular Complex Detection (MCODE) a plugin from Cytoscape (Version: 3.7.2) was used to screen modules of the PPI network. Finally, four modules were obtained: MCODE1, MCODE2, MCODE3, and MCODE4 (Fig. 5A-D). Next, the “WebGestaltR (v0.4.2)” package in R was used to perform KEGG and GO functional enrichment analysis on 49 DEGs of the MCODE1 module. We identified 308 terms with a significant difference in BP between samples from patients with sepsis and normal samples (FDR < 0.05). The top 20 terms included neutrophil degranulation, neutrophil activation involved in immune response, neutrophil activation, and neutrophil-mediated immunity (Fig. 5E). Forty-one GO items with a significant difference in CC between the two groups were annotated (FDR < 0.05), and the top 20 terms are shown in Fig. 5F. In addition, 9 terms were significantly different in terms of MF (FDR < 0.05) between the two groups (Fig. 5G). In the KEGG pathway analysis of MCODE module genes, 10 terms, including Primary immunodeficiency、Th1- and Th2-cell differentiation, T-cell receptor signaling pathway, and NK cell-mediated cytotoxicity, with a significant difference between the two groups were annotated (FDR < 0.05) (Fig. 5H).

### Identification of hub genes

**Fig. 6:**
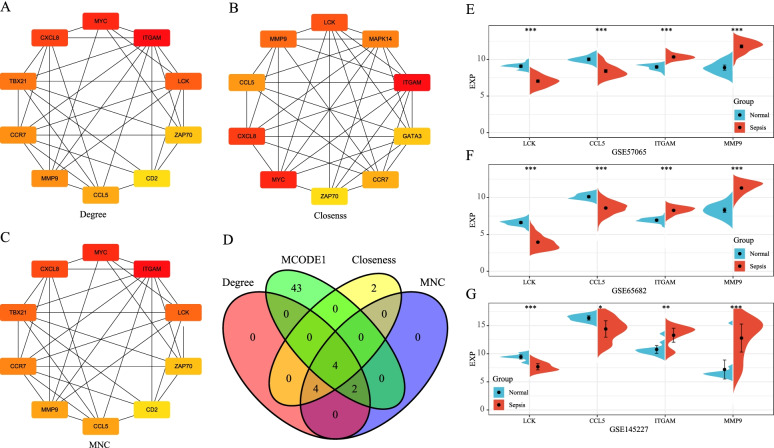
Protein-protein interaction (PPI) network analysis of hub genes. **A** PPI network of hub genes obtained by Degree algorithm. **B** PPI network of hub genes obtained by Closeness algorithm. **C** PPI network of hub genes obtained by MNC algorithm. **D** Venn diagram of identified hub genes. **E** Expression profile of hub genes in the GSE57065 dataset. **F** Expression profile of hub genes in the GSE65682 dataset. **G** Expression profile of hub genes in the GSE145227 dataset

For the PPI network of 661 co-expressed DEGs, three methods in the cytoHubba plugin from Cytoscape (Version:3.7.2), namely MNC, Degree and Closeness, were used to select the key genes in PPI. The top 10 genes were selected as key genes, and the PPI networks of genes screened by these three algorithms are shown as Fig. 6A-C.

Then, the genes obtained from these three algorithms were intersected with those in the MCODE1 module, and final four hub genes were obtained: lymphocyte cell-specific protein-tyrosine kinase (*LCK*), C-C motif chemokine ligand 5 (*CCL5*), integrin Subunit Alpha M (*ITGAM*), and matrix metallopeptidase 9 (*MMP9*). The Venn diagram of the four genes is shown in Fig. 6D.

Moreover, we compared the expressions of these four hub genes in samples from patients with sepsis and normal samples in different datasets. The results suggested that the expressions of *LCK* and *CCL5* were significantly higher in normal samples than in samples from patients with sepsis, while the expressions of *ITGAM* and *MMP9* were significantly lower in normal samples than in samples from patients with sepsis in the GSE57065 dataset (Fig. 6E). The same results were observed in the external independent datasets GSE65682 and GSE145227 (Fig. 6FG).

### Construction and verification of a diagnostic model

**Fig. 7:**
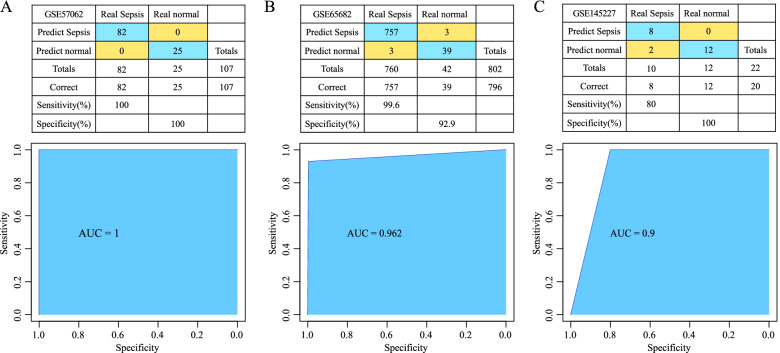
Classification and receiver operating characteristic (ROC) curve analysis of diagnostic models constructed using hub genes. **A** Results of classification and ROC curve analysis of the diagnostic model in the GSE57065 dataset. **B** Classification results and ROC curves of the diagnostic model in the GSE65682 dataset. **C** Classification results and ROC curve of the diagnostic model in the GSE145227 dataset

The default parameters of the SVM algorithm function in the R package e1071 (V1.7.6) was used to train the GSE57065 cohort, and then used the trained classifier to verify the cohorts GSE65682 and GSE145227. Four hub genes were used as features in the training dataset to obtain their corresponding expression profiles and were subsequently utilized to construct the SVM. All 107 samples were correctly classified with an overall accuracy of 100%. The sensitivity and specificity of the correctly classified model were 100%, and the receiver operating characteristic (ROC) area under the curve (AUC) was 1 (Fig. 7A). Moreover, the GSE65682 dataset was verified, and out of 802 samples, 796 samples were correctly classified was an overall accuracy of 99.3%. The sensitivity and specificity of the correctly classified model were 99.6 and 92.9%, respectively, and the AUC was 0.962 (Fig. 7B).

The GSE145227 dataset was further verified, and out of 22 samples, 20 samples were correctly classified with an overall accuracy of 90.91%. The sensitivity and specificity of the correctly classified model were 80 and 100%, respectively, and the AUC was 0.9 (Fig. 7C). These results suggested that the diagnostic model could effectively differentiate between normal samples and samples from patients with sepsis, and the four genes can be used as reliable biomarkers for the diagnosis of sepsis.

## Discussion

In this study, we evaluated the immune infiltration in patients with sepsis based on GSE57065 and GSE65682 datasets and found that the immune score was significantly lower in samples from patients with sepsis than in normal samples. We identified a total of 786 DEGs between the two groups. Meanwhile, the results of WGCNA analysis revealed that the expression of genes in the blue module was significantly negatively correlated with in both samples from patients with sepsis and normal samples, while the expression of genes in the blue module was significantly positively correlated with the immune score in both groups. The results of the WGCNA analysis of genes in the brown module were the opposite of those of genes in the blue module. By intersections of blue and brown module genes and DEGs, we obtained 328 DEGs that were positively correlated with the immune score and 333 DEGs that were negatively correlated with the immune score were obtained. We constructed a PPI network of 661 DEGs using the STRING online database and screened for network modules using the MCODE plugin in Cytoscape. The MCODE plugin identified four modules, and through functional annotation analysis, these modules were found to be related to the immune response.KEGG analysis and GO functional enrichment analysis were performed on 49 genes of the MCODE1 module. The results showed that biological processes such as neutrophil degranulation, neutrophil activation involved in immune response, neutrophil activation, and neutrophil-mediated immunity were significantly enriched in the DEGs. KEGG pathway analysis revealed that pathways such as primary immunodeficiency, Th1- and Th2-cell differentiation, T-cell receptor signaling pathway, and NK cell-mediated cytotoxicity were significantly enriched in the DEGs. Studies have shown that neutrophil activation leads to the release of neutrophil extracellular traps, which are involved in both pathogen confinement and phagocytosis as well as activation of the coagulation cascade [19, 20]. Therefore, neutrophils are a key factor in vascular cell dysfunction, immune response, and hemostasis caused by septic shock in the host. Moreover, Yoon et al. suggested that the overexpression of heme oxygenase-1 (HO-1) contributes to sepsis-induced immunosuppression during the late phase of sepsis by promoting Th2 polarization and Treg function [21]. Then, the genes identified using the three algorithms (MNC, Degree, and Closeness) were intersected with the MCODE1 genes to obtain the final four hub genes: *LCK*, *CCL5*, *ITGAM*, and *MMP9*. All these four genes have important roles in the immune reaction and inflammatory reaction after inquiry. The *LCK* gene is a protein-coding gene that encodes a key signaling molecule for T-cell selection and maturation. LCK-related diseases include immune deficiency and autoimmune cardiopathy. LCK plays a crucial role in the selection and maturation of T cells in the thymus, the function of mature T cells, and the signal transduction pathway associated with T-cell antigen receptor (TCR). Cytoplasmic binding with CD4 and CD8 surface receptors and the association of TCR with the MHC complex binding peptide antigen promote the interaction between CD4 and CD8 receptors with leads to the recruitment of relevant LCK proteins to the vicinity of the TCR/CD3 complex. Next, the interaction of LCK with the cytoplasmic tail of TCR/CD3, which contains three subunits of immunoreceptor tyrosine-based activation motifs (ITAMs), leads to the activation of the TCR/CD3 signaling pathway and phosphorylation and activation of tyrosine kinase ZAP70, eventually promoting T-cell activation. Then, LCK induces the secretion of a large number of signaling molecules, eventually leading to the release of the lymphatic factor. Additionally, LCK also participates in other receptor signaling pathways [22–24]. The *CCL5* gene is a chemokine-encoding gene located on the q arm of chromosome 17. CCL5, a member of the CC chemokine family, is involved in the host immune response and inflammation process and acts as a chemoattractant for blood monocytes, memory T helper cells, and eosinophils [25–27]. The *ITGAM* gene is a protein-coding gene associated with itGAM-related diseases, including systemic lupus erythematosus and the Shwartzman phenomenon. Moreover, ITGAM promotes the adherence of monocytes, macrophages, and granulocytes as well as mediates the uptake of opsonized particles and pathogens. The ITGAM protein is identical with CR-3, which is the receptor for the iC3b fragment of the third complement component, and may help in the identification of C3b RGD peptides. Integrin ITGAM/ITGB2, which acts as a receptor for fibrinogen, factor X, and ICAM1, recognizes the fibrinogen gamma chain of P1 and P2 peptides, regulates neutrophil migration. Furthermore, the CD177-PRTN3 complex mediates the activation of TNF-alpha primed neutrophils in association with the beta subunits ITGB2/CD18, eventually leading to the phagocytosis of neutrophilic infiltrates [28–30]. Finally, we used SVM to construct the four gene-based diagnostic model, which showed good performance in the GSE57065, GSE65682, and GSE145227 datasets. Previous studies have used a series of biomarkers to better identify patients at risk for sepsis. In 2007, Kofoed et al. showed that three or six pro-inflammatory biomarkers could be used to identify patients with bacterial infections more accurately than using a single biomarker [31]. In 2009, Shapiro et al. applied this method to the diagnosis of patients with severe sepsis [32]. The three best predictors were identified as IL-1 receptor (IL-1RA), protein C, and neutrophil gelatinase-associated lipoprotein (NGAL), all of which could be used as biomarkers for sepsis [33]. The best biomarkers for diagnosing sepsis or evaluating the occurrence of severe sepsis may include a combination of both pro-inflammatory and anti-inflammatory markers. Some studies showed that a mixture of pro-inflammatory and anti-inflammatory cytokines could identify patients at risk of severe sepsis at an early stage, thus leading to predict patient outcomes [34].

Previously, the use of gene-related network analysis in the regulation of sepsis has been explored, differential gene expression analysis and enrichment analysis were performed using transcriptome data to investigate the potential biological pathways that regulate the development of sepsis [35–38]. For example, Zhongheng Zhang’s study identified co-expression modules in sepsis through WGCNA method, and found that they were associated with clinical features and functional biological pathway [39]. In our study, we evaluated immune scores in septic patients and normal samples, and then analyzed immune invasion-related genes and potential transcriptome biomarkers by WGCNA for septic patients, and finally established a diagnosis model of sepsis based on the SVM classification algorithm, which were the innovation of the study. In addition, compared with the single data set of previous studies, this study integrated multiple sets of data. For example, SVM algorithm was used to train the GSE57065 cohort, then used the classifier to verify the cohorts GSE65682 and GSE145227 to make the results more accurate.

However, there are some limitations. This study is based on a public database with a limited number of samples, which may lead to selection bias. So, further studies with larger sample sizes will be needed to support these findings, and more cell and animal studies, as well as clinical practice, will be needed to validate our results.

Currently, huge efforts have been put into the detection of biomarkers that could help clinicians make an early diagnosis of sepsis. It is vital to find diagnostic markers of sepsis at the genomic level. According to the abundance of immune infiltration-related genes, we identified four hub genes involved in the PPI network to establish a four-gene diagnostic model for sepsis. These genes play an important role in the immune and inflammatory response to sepsis, indicating the reliability of the model in diagnosing sepsis.

## Supplementary Information

**Additional file 1.**

**Additional file 2.**

**Additional file 3.**

**Additional file 4.**

## Data Availability

We have provided detailed information about the materials and methods in our manuscript.

## Abbreviations

WGCNA: Weighted gene co-expression network analysis
SVM: Support vector machine
KEGG: Kyoto Encyclopedia of Genes and Genomes
SIRS: Systemic inflammatory response syndrome
MODS: Multiple organ dysfunction syndrome
PPI: Protein-protein interaction
ChIP-seq: Chromatin immunoprecipitation sequencing
GO: Gene Ontology
NK: Natural killer
