# Supplementary material for: Identification of a novel four-gene diagnostic signature for patients with sepsis by integrating weighted gene co-expression network analysis and support vector machine algorithm
Source: Hereditas. 2022 Feb 21;159:14. doi: 10.1186/s41065-021-00215-8 (PMC8859894; doi:10.1186/s41065-021-00215-8)
Supplement: Supplementary file 1 — Additional file 1. [file 41065_2021_215_MOESM1_ESM.docx]

"","logFC","AveExpr","t","P.Value","adj.P.Val","B"

"XIST",-2.85008134912893,5.11706539385848,-5.2096010710611,9.06075350436492e-07,4.49714914364021e-06,4.87091443138147

"CCR3",-2.78145947317074,5.49170663551402,-11.9651130377461,1.47747741686375e-21,7.91949838172882e-20,38.468207136377

"LRRN3",-2.67509952439025,4.31683672897196,-15.4580359469839,3.33112830079299e-29,8.37535847486213e-27,55.9268243574513

"GNLY",-2.66282328780489,8.35594948598131,-10.3715109097509,6.21876384725879e-18,1.69606210469004e-16,30.1928008892634

"NELL2",-2.61538270243903,6.09666682242991,-15.756649350244,7.86411342952985e-30,2.74673187607208e-27,57.3567244506723

"CD247",-2.54613135609758,8.23408074766355,-15.5587204161991,2.04493681735223e-29,5.82672457628454e-27,56.4101798491584

"IL2RB",-2.47402316097562,7.55550523364486,-17.5229975292416,1.92863890472533e-33,1.81585545573161e-30,65.5836314194

"KLRF1",-2.34706155609757,6.15989411214953,-9.67813266482225,2.38160590179348e-16,5.12660793273737e-15,26.5806331224847

"ITK",-2.32869599024392,7.90250345794392,-12.4873385274924,9.87842900079699e-23,6.64339689479065e-21,41.1515378130652

"TGFBR3",-2.31331793414635,5.69519271028037,-11.5866004051866,1.06161581186247e-20,4.70128638157093e-19,36.5121620026553

"A2M-AS1",-2.27566311219513,5.62141747663551,-9.36159289981882,1.25323628633477e-15,2.39108650049159e-14,24.9361960988814

"FAIM3",-2.27274278292684,7.76661948598131,-15.8027334542457,6.29962434265214e-30,2.33562745601181e-27,57.5764110203923

"HLA-DQA1",-2.27059354634147,5.40679336448598,-8.57755586933769,7.4707442254258e-14,1.07709032091608e-12,20.891601718181

"TRBC1",-2.19838305528456,9.08648744548287,-11.9424024947663,1.66264502307323e-21,8.7602379500367e-20,38.3510879261416

"TXK",-2.17637620000001,5.51675411214953,-12.2156092722903,4.02643360130124e-22,2.35019999019349e-20,39.7577554050568

"FGFBP2",-2.17041656097562,6.04918411214953,-8.16164133027664,6.38394416624699e-13,8.06089276501916e-12,18.7715949188067

"LBH",-2.15818236585367,7.11066785046729,-14.9202150171685,4.60715437941643e-28,9.50170743678693e-26,53.3240342095332

"SGK223",-2.13859163414635,6.55027130841122,-16.7058057891951,8.59974208783203e-32,6.20758049706675e-29,61.8273001827352

"PRF1",-2.13126659512196,7.90220728971963,-11.7391849915817,4.78934295613243e-21,2.26943592374284e-19,37.3016726883036

"HLA-DPA1",-2.12699539674798,8.01523174454829,-12.6179207444729,5.0387266736951e-23,3.57749593832352e-21,41.8193035999666

"TRAC",-2.11542693658538,8.51290509345794,-13.0092309903556,6.75945231747194e-24,5.84870104668951e-22,43.8118196483719

"CCR7",-2.11327438536586,7.14805411214953,-9.8490145135932,9.70445940329776e-17,2.20745870145392e-15,27.4699819053405

"IL7R",-2.05484187073172,9.36447257009346,-10.8828367184967,4.23265448673382e-19,1.44799578057221e-17,32.8570317249612

"LCK",-2.0540648682927,7.52011242990654,-13.1834542041906,2.77578002642409e-24,2.57980757391475e-22,44.6945685032828

"NLRC3",-2.03600217560977,6.92081046728972,-12.4544546689076,1.17059357482018e-22,7.68200200954004e-21,40.9831645303443

"RASGRP1",-2.03331554146343,7.11531738317757,-10.7403579187706,8.94535488723092e-19,2.86132437345621e-17,32.1150665738315

"CLIC3",-2.02760129268294,5.0617153271028,-10.7377923708567,9.06674499398504e-19,2.8958755581821e-17,32.101702426037

"IFIT1",-2.02232907804879,5.87964691588785,-4.95977496323526,2.62207599227775e-06,1.20273364992109e-05,3.84121359507273

"FCER1A",-1.99108527317074,3.49997476635514,-18.3711402229907,4.09643212723874e-35,6.33630269395392e-32,69.3902885214508

"GPR18",-1.98833526341464,6.11464785046729,-12.1243758824673,6.46154150736166e-22,3.6156248408764e-20,39.2885832426072

"SULF2",-1.96858007317074,6.15222654205607,-8.78135128157716,2.59358362052115e-14,4.03767457242168e-13,21.9377867559591

"KLRB1",-1.9679258195122,6.89641953271028,-10.2874049669392,9.67858592943859e-18,2.55597290612186e-16,29.7543553731015

"EOMES",-1.96529790731708,5.96297504672897,-10.5318521264761,2.67626506130174e-18,7.88496869421622e-17,31.0286003443819

"CD2",-1.96322693658538,7.88998934579439,-10.8901399457444,4.07343653408034e-19,1.39794402766259e-17,32.8950508636794

"CD3E",-1.96000720487805,7.32723280373832,-13.8410000585695,9.90829987181958e-26,1.2894327335228e-23,47.9996926638212

"GPRASP1",-1.95916575609757,4.51783925233645,-13.4595549224278,6.8135753227152e-25,7.16252299094163e-23,46.087624393901

"PCED1B",-1.94624586341464,6.55489887850467,-14.8115697798586,7.86471778880777e-28,1.54827694287847e-25,52.7940458700687

"PVRIG",-1.9412780780488,6.26714476635514,-13.7228866418896,1.79723087956246e-25,2.17424774843157e-23,47.4092234076061

"PLEKHA1",-1.93931406097562,5.79943070093458,-13.860849490395,8.96605079034775e-26,1.17672624160594e-23,48.0987798469813

"BCL11B",-1.92974231382115,5.77902059190031,-14.5795296991876,2.47562539811993e-27,4.39423508166288e-25,51.6574992446873

"EVL",-1.92745946585367,7.23731813084112,-15.4605203993085,3.29121258197332e-29,8.37535847486213e-27,55.9387664518585

"TBC1D4",-1.92008316341464,5.31162359813084,-11.7541631290373,4.42968695820664e-21,2.11754682295728e-19,37.3791035442358

"TRIB2",-1.89892238780489,5.52060546728972,-15.5375352227811,2.26581511210207e-29,6.13721354192652e-27,56.3085798454285

"UBASH3A",-1.85424096097562,5.44620130841122,-12.44005363732,1.26096081807284e-22,8.1010283921356e-21,40.9094012736462

"GZMK",-1.85081278536587,7.23063130841121,-9.92637923617381,6.46188061057033e-17,1.49980733785531e-15,27.8728884372368

"ESYT1",-1.84978714146343,7.31896691588785,-19.7141096824306,1.10748662659281e-37,2.66473587765193e-34,75.2262704324376

"BACH2",-1.84610550487806,5.03277275700935,-13.3049316008515,1.49487598692191e-24,1.47143361349064e-22,45.308384588689

"NOG",-1.84534131219513,3.40053,-16.8569916274303,4.23260085880837e-32,3.27346327133912e-29,62.5287660321749

"CHRM3-AS2",-1.84528065853659,4.60263168224299,-10.65281436908,1.41703524040322e-18,4.37744623836402e-17,31.6589789808108

"MARCKSL1",-1.83755997073172,7.23489327102804,-12.8348259684555,1.65208045991068e-23,1.3056862174951e-21,42.9253993165848

"NR1D2",-1.83610175365854,5.30541738317757,-10.1516332020029,1.9766964822651e-17,4.94859680039894e-16,29.0466221297872

"CACNA2D3",-1.81660123414635,4.30171990654206,-12.8859797382891,1.27078849866552e-23,1.03454605032338e-21,43.1856689469804

"MS4A1",-1.78173793414635,6.72791427570093,-8.67398179507763,4.53084682881618e-14,6.79941012321652e-13,21.3860683936201

"HLA-DMB",-1.78025806829269,6.50613887850467,-10.0188029492362,3.97480530878809e-17,9.49001201342957e-16,28.3543861357542

"PRKX",-1.77960431219513,4.88188588785047,-14.3149052597543,9.22230099188076e-27,1.42649234270841e-24,50.3538128779096

"CD8A",-1.77711080000001,6.98000280373832,-8.96935763198421,9.74192380898145e-15,1.63409264201002e-13,22.9064619958196

"GZMB",-1.7663758878049,7.9212791588785,-7.89097010301949,2.54802001331662e-12,2.93809229970029e-11,17.4050861769538

"GPR183",-1.76139595121953,6.3810191588785,-10.8906635838839,4.06225375163962e-19,1.39794402766259e-17,32.8977767667716

"CTSW",-1.75329829268294,7.8548108411215,-11.9118702610101,1.94877539663609e-21,1.00958687115202e-19,38.1935804207561

"CD160",-1.75001575121952,4.81408523364486,-9.50027152595871,6.05751304116285e-16,1.21234237436582e-14,25.656073061163

"CECR1",-1.73786488780489,8.37173271028037,-8.67302429116464,4.55342224744414e-14,6.82855670141294e-13,21.381153505139

"CDC25B",-1.72724512195123,7.70503682242991,-16.0919797927569,1.57488722227893e-30,6.96003730580616e-28,58.9491935784822

"MAL",-1.71807679512196,6.73042261682243,-11.2564189104667,5.96775635312319e-20,2.35824386545406e-18,34.7997399369047

"TUBB2A",-1.71323306341464,6.22525663551402,-4.60824783263375,1.11153349081022e-05,4.61469665327747e-05,2.44722621387594

"TCL1A",-1.7077231585366,6.04574457943925,-9.71253319173772,1.98794716071121e-16,4.30920878530543e-15,26.7595951833818

"GATA3",-1.68544702601627,4.39119940809969,-17.8546470714365,4.23056869079395e-34,4.36252214281634e-31,67.0833279045448

"NMT2",-1.68534472073171,3.66313521028037,-15.5711436979778,1.92561432002805e-29,5.63502406759558e-27,56.4697337035852

"MAP3K7CL",-1.68208069756098,5.83566841121495,-9.40396791384775,1.0036779326863e-15,1.95104538889783e-14,25.1560586182975

"CPA3",-1.68082872195123,4.26106186915888,-9.60834835508438,3.43554180701864e-16,7.18808288222113e-15,26.2177260871362

"LEF1",-1.6751729788618,5.34286937694704,-12.6097607524,5.2550044108069e-23,3.70674659661314e-21,41.7776156392137

"ABLIM1",-1.67441196097562,5.92061018691589,-18.3367866756831,4.77962162387589e-35,6.46891914156452e-32,69.2379264357044

"DDHD2",-1.67341171219513,4.43284953271028,-11.5165766645406,1.53036942251637e-20,6.58849897506797e-19,36.1494290008063

"TGFBI",-1.66378852195122,8.14680551401869,-6.87717788819288,4.04643306060555e-10,3.35729915430702e-09,12.412917035011

"DYRK2",-1.66189783048782,5.74221635514019,-14.1512180085737,2.0884160148633e-26,3.03521132898421e-24,49.5434660501251

"SGK1",-1.65449325853659,7.85077018691589,-9.23113506263966,2.48135737629642e-15,4.51544487257974e-14,24.2599618297816

"SH2D1B",-1.65440024878049,3.90576406542056,-12.8605404387179,1.44788260698186e-23,1.16125547608119e-21,43.0562627184514

"LGALS2",-1.65346436097562,5.17178588785047,-8.370448585357,2.17982483614715e-13,2.92647903451746e-12,19.8331604018874

"AL833181",-1.64790412682928,5.90427140186916,-13.8898326123783,7.74935976353915e-26,1.03587892394716e-23,48.2433870888241

"FYN",-1.64065677073172,7.47021118380062,-11.3093954803314,4.5222135690649e-20,1.81685593391652e-18,35.0748222591161

"MX1",-1.64054750243904,7.12097158878505,-5.87182854482437,4.76093083303123e-08,2.86542404639498e-07,7.73985901928544

"PRKCQ",-1.63457688048782,6.20313420560748,-15.0020829801925,3.08189021389074e-28,6.6077557011687e-26,53.7224768328329

"TCF7",-1.63346580000002,7.02089588785047,-15.985943417714,2.6148409401153e-30,1.08893039534994e-27,58.4471590937015

"HLA-DMA",-1.63015441463415,7.96156878504673,-9.29991788654909,1.73112182235878e-15,3.24005558022294e-14,24.6163745491973

"PPP1R16B",-1.62941450569107,5.84708021806854,-14.4813444250198,4.02909525183324e-27,6.92460775225785e-25,51.1747117502595

"THEMIS",-1.62533712682928,3.90918873831776,-8.34473442679262,2.48895212400477e-13,3.31885826633764e-12,19.7021142489548

"CD7",-1.61605018048782,5.88610803738318,-13.711660015802,1.90202655205998e-25,2.22651711510252e-23,47.3530240241276

"CCL5",-1.61336835121953,8.80080327102804,-8.86679659924046,1.6625778086387e-14,2.69081632631323e-13,22.3776411579402

"FLT3LG",-1.61138577317074,5.04786242990654,-13.8662316662223,8.72643607054914e-26,1.15226203114477e-23,48.1256401814514

"LINC01215",-1.60379729268293,4.85196570093458,-8.87997437371369,1.55229841086844e-14,2.5198667231901e-13,22.4455368198564

"CD3G",-1.60067620975611,6.8198691588785,-8.82930053061274,2.02097438277671e-14,3.21323056233698e-13,22.1845362241227

"NOV",-1.59571400487805,4.18327257009346,-21.7887097723185,1.85148663322131e-41,1.00234857606019e-37,83.7887961407529

"ZAP70",-1.57067683414636,6.46996093457944,-12.54140205969,7.47429435070472e-23,5.12202038495287e-21,41.4281711728402

"MYBL1",-1.56495506097562,5.06620364485981,-10.7014670875396,1.09734141126427e-18,3.45391399141393e-17,31.9124680033638

"ANKRD36B",-1.56274192682927,6.15307869158879,-10.1150653349224,2.39587493012776e-17,5.92267940775305e-16,28.8560296388985

"SPOCK2",-1.5618693390244,6.39225780373832,-18.6634755078105,1.10915329459431e-35,1.84759343034153e-32,70.6806032578234

"CD27",-1.55983649268294,7.85682448598131,-10.2167580854486,1.40340693203158e-17,3.60507439064577e-16,29.3860833151917

"GZMA",-1.55828540000001,7.25930037383178,-6.40113849246885,4.02055512797604e-09,2.86870251388208e-08,10.1586831784177

"STAT4",-1.55504935121952,6.7778123364486,-12.9878114300904,7.54249846146581e-24,6.35536203046857e-22,43.7030998908703

"TRG-AS1",-1.54906312682928,7.25579271028037,-9.37283114311036,1.18157183860489e-15,2.27157638770337e-14,24.9944961801413

"TRANK1",-1.54041154634147,6.96058009345794,-9.4851748128557,6.55667039609346e-16,1.30620696805339e-14,25.5776600237578

"TSHZ1",-1.53925341707318,5.5970838317757,-13.7259438871403,1.76971074598077e-25,2.15298237102324e-23,47.4245254168928

"MPEG1",-1.53259573170733,8.98342238317757,-7.77485561644125,4.59823464182787e-12,5.09594530034711e-11,16.8225771245096

"TRAT1",-1.5267562195122,5.57438252336449,-6.73398883231378,8.12586136342408e-10,6.40573454040584e-09,11.727817858528

"RARRES3",-1.52423685365855,7.70547925233645,-8.41856415491389,1.70044220792996e-13,2.32468914221738e-12,20.0785963591405

"SIDT1",-1.52346297073172,5.28842112149533,-11.7407922776127,4.74938726274839e-21,2.26537403468759e-19,37.3099823163392

"P2RY10",-1.52264313658537,4.61897420560748,-10.9682039349739,2.70403653811602e-19,9.67866301370289e-18,33.3013478468672

"RTN1",-1.52254773170733,4.93591471962617,-10.5030596470463,3.1136826667526e-18,9.0263451336717e-17,30.8785283489397

"ARL4C",-1.50838697682928,6.44836518691589,-14.4111449128222,5.71120090847543e-27,9.44092028038439e-25,50.8288601682047

"TBX21",-1.50810938536586,6.01480542056075,-11.3910946059385,2.94908781793261e-20,1.2106654921831e-18,35.4988132566914

"IL32",-1.50661316585367,8.14767205607477,-8.34324297091763,2.50816489383867e-13,3.33625987568036e-12,19.6945160220174

"SYTL2",-1.50359891869919,4.71679657320872,-11.6068696432084,9.55022390684212e-21,4.24661393639972e-19,36.6171124615719

"ZNF600",-1.50215822439026,5.54644252336449,-8.84976565781268,1.81675020834844e-14,2.92721173822809e-13,22.2899159090812

"CX3CR1",-1.49716535609757,7.69694523364486,-7.32458649974171,4.43647982323561e-11,4.23224539965494e-10,14.5880860401037

"LDLRAP1",-1.49650772439026,7.08030981308411,-13.6315560309674,2.85098171730331e-25,3.24936889937911e-23,46.9516547493511

"RUNX3",-1.49602691382115,6.09417021806854,-15.837040275407,5.34160954651213e-30,2.09726253624682e-27,57.7397821795192

"CCR6",-1.48936430731708,3.86578560747664,-15.1300068460297,1.64683802353479e-28,3.75392393680482e-26,54.343469174735

"TRDV3",-1.48921539024391,4.04038813084112,-12.0251369417647,1.08158878609454e-21,5.91459726335282e-20,38.7775927124258

"LDOC1L",-1.47049532682928,5.26714074766355,-15.5613530729159,2.01904519867443e-29,5.82672457628454e-27,56.422801684168

"ABHD14B",-1.46852046341464,6.15985579439252,-13.5872955834717,3.56647514009833e-25,3.96061636711946e-23,46.7296018616061

"MAN1C1",-1.46766884146342,4.34198943925234,-17.595407664943,1.38319844262697e-33,1.36150737614032e-30,65.9122891071051

"KDM2B",-1.46635912682928,6.44636934579439,-13.0448925796416,5.63242730289064e-24,4.97837605077946e-22,43.9927358037622

"FCRL3",-1.46207557073171,4.93552827102804,-12.5591878728415,6.81941425375898e-23,4.7030068683169e-21,41.5191271388245

"CHI3L1",-1.45801250406505,5.92170062305296,-5.40413032094368,3.88530655360268e-07,2.0362098691508e-06,5.69356606751562

"LOC93622",-1.4557890390244,5.41907018691589,-13.8138049089295,1.13628259941379e-25,1.44333975903698e-23,47.8638687402903

"IFI44L",-1.43980537073172,4.94800495327103,-3.33800676712562,0.00115618539988566,0.00324273990862893,-1.96498116187492

"IFIT3",-1.43847645365854,7.83901177570093,-4.27162163241603,4.1731513352014e-05,0.000157082551996847,1.17753862879036

"IFIT2",-1.43607509024392,7.3149846728972,-4.80272333814585,5.03726000876351e-06,2.20992434136495e-05,3.21020375391616

"RCAN3",-1.435671699187,4.8832369470405,-13.2741742639418,1.74824058924996e-24,1.70532207027964e-22,45.1531031375589

"LY9",-1.43494702113822,6.0244208411215,-13.4071818542854,8.88876716612007e-25,9.16601204677763e-23,45.8239471333723

"SEPT1",-1.4327652682927,5.60432897196262,-12.1635777273531,5.27278737509593e-22,3.00479501599216e-20,39.4902520607054

"BZRAP1-AS1",-1.42484582682928,6.17862247663551,-13.2144675382506,2.36977824594532e-24,2.25076964543623e-22,44.8514097707691

"DPP4",-1.42372016585366,3.89177953271028,-15.5850405980567,1.80040888540323e-29,5.34080197443932e-27,56.5363291074266

"PASK",-1.4191402390244,4.4999307165109,-15.4581793574394,3.32881106260545e-29,8.37535847486213e-27,55.9275137135523

"CAMK4",-1.41774778699188,4.53064196261682,-12.3328833563322,2.19410396411944e-22,1.33840341811286e-20,40.3599678703614

"CXXC5",-1.41496998211384,6.68614074766355,-12.0376844720139,1.01334830054665e-21,5.55545758185764e-20,38.8422375587191

"BLNK",-1.41374803414636,5.8270153271028,-6.52657047823425,2.2091418624531e-09,1.64113094447416e-08,10.7459793509539

"SEPT9",-1.41205731951221,6.16382371495327,-19.5203139327293,2.56361296049498e-37,5.55150386595188e-34,74.3985322116191

"BTN3A3",-1.40599185121952,6.05782331775701,-7.02710669700328,1.939154362815e-10,1.67433762865865e-09,13.1362390079701

"BTBD11",-1.40296262682927,3.72192457943925,-18.3555704393172,4.39296802159795e-35,6.34198150051357e-32,69.3212537501442

"SKAP1",-1.40019253658537,5.85215925233645,-11.133488028236,1.13640124023566e-19,4.29472405886617e-18,34.1609928517277

"ALDH1A1",-1.39468207317074,3.74683822429907,-7.62219973148603,9.95800639245399e-12,1.05036838007107e-10,16.0604761363726

"USP11",-1.39431619024391,6.43541542056075,-19.2934557514579,6.88877817578198e-37,1.3561499217869e-33,73.4234252930141

"HLA-DRA",-1.38457514390245,9.84986612149533,-7.86340977432319,2.93185754160452e-12,3.35213173513442e-11,17.2666124806396

"ZC3H12D",-1.36675028780489,5.67472869158879,-8.46674309702574,1.32570235879882e-13,1.83908293272187e-12,20.3246433992802

"KLRD1",-1.36406304390245,6.00143940809969,-8.32930306058734,2.69502983227346e-13,3.5738439080148e-12,19.6235131494566

"PYHIN1",-1.36255737560976,4.69929355140187,-8.50889533431329,1.06601594961593e-13,1.5038811328295e-12,20.5401415191811

"MAP3K14",-1.35177186341464,4.96115308411215,-14.6232621700124,1.99353172461347e-27,3.6277251677735e-25,51.872180621152

"MME",-1.33970600487806,7.42988261682243,-3.76909677027059,0.000266728387725657,0.000859524291101057,-0.587399515313793

"SLFN5",-1.33163458373985,5.94917059190031,-10.5599054435294,2.30927902560533e-18,6.88807676301423e-17,31.1748120210344

"GPR171",-1.32901774146343,6.54159682242991,-8.02871885768086,1.26142751982032e-12,1.52349207706129e-11,18.0990824617619

"CD81",-1.3288208390244,7.63490971962617,-12.1913586517069,4.56558788419039e-22,2.61555041354875e-20,39.633102488658

"MYC",-1.32777447804879,7.41755280373832,-10.8738224901287,4.43779044149873e-19,1.51339137024653e-17,32.8101036005799

"ATP6V0E2",-1.31947335609757,5.91060841121495,-15.7087346607555,9.90674079719695e-30,3.20194734273582e-27,57.1280307285889

"MSANTD2",-1.31869252682928,4.89732280373832,-8.09019441034355,9.20878583662557e-13,1.13627497032551e-11,18.4097811735579

"DNMT1",-1.31821857560977,6.75509401869159,-11.7166017167407,5.38770473642973e-21,2.5198865241336e-19,37.1849021964547

"PTPN4",-1.31559217560976,4.20677537383178,-11.5262329876752,1.45508053596163e-20,6.27704427735418e-19,36.1994648690449

"RP11-732A19.1",-1.31457738536586,4.2673376635514,-11.6356793093742,8.21701093293651e-21,3.71480943116368e-19,36.7662463778526

"ID3",-1.30910137560976,4.5362938317757,-13.3387623100389,1.25852002408948e-24,1.25591018993814e-22,45.4790770126469

"KIAA0355",-1.30805478048781,5.4353014953271,-14.0578746519314,3.3328026665093e-26,4.6562478544038e-24,49.0800425717127

"LAT",-1.30797152682928,6.48521289719626,-12.5439366025462,7.37725140366121e-23,5.07156759194551e-21,41.4411342745473

"IL10RA",-1.30777284390245,8.5610891588785,-11.5403115465787,1.35191046274035e-20,5.87863876920528e-19,36.2724069424205

"LOC102724356",-1.30764398536587,6.58916271028037,-9.58177921242395,3.94967432412343e-16,8.13798263452834e-15,26.0796055767272

"CCDC92",-1.30550029756099,6.2060823364486,-10.8674556374906,4.58864958127776e-19,1.5599247516887e-17,32.7769565090798

"LPIN1",-1.30386452845529,5.25322738317757,-15.2656905057047,8.4896667017738e-29,1.97681432717109e-26,54.9999812766219

"ETS1",-1.30309811544717,6.14599579439252,-14.9361415163525,4.2602657874633e-28,8.87077457956901e-26,53.4016090648811

"RP11-285F7.2",-1.30300698536586,5.64058429906542,-13.9396143031372,6.03350466956146e-26,8.16597147620959e-24,48.4915577611493

"BTN3A2",-1.30177461219513,6.79277593457944,-7.03702567505638,1.84669082735842e-10,1.59896400905425e-09,13.1842992752368

"DENND2D",-1.30116409268293,6.83776700934579,-15.7247837407367,9.16912792763112e-30,3.05473023496695e-27,57.2046637751564

"THEM4",-1.29705391869919,4.68530672897196,-12.8545693669858,1.4929183903528e-23,1.19295748129483e-21,43.025880494913

"BCL9L",-1.29575060243903,5.56104906542056,-15.639063567045,1.38663549631365e-29,4.28965595323888e-27,56.7949860292925

"LOC728392",-1.2943768292683,6.8391876635514,-6.6380675139893,1.2923649888348e-09,9.90310114409682e-09,11.2721600666296

"LOC283588",-1.29345219512196,3.43159626168224,-8.79811334201422,2.37704099871796e-14,3.73276452699329e-13,22.0240206300948

"HSH2D",-1.29327318048781,5.01131785046729,-9.05048809952041,6.3793212291187e-15,1.09725338535795e-13,23.3253985230869

"ITGA4",-1.28779821788619,6.00566548286604,-6.81573619813998,5.46115533550851e-10,4.4143829335736e-09,12.1182418475937

"SSBP3",-1.28703546016261,5.54001962616822,-10.8653758889872,4.63903206909832e-19,1.57458055574176e-17,32.766128711147

"DOCK10",-1.28598460487806,4.75999588785047,-10.9036331790981,3.79487261151049e-19,1.3169545897798e-17,32.9652903342513

"HLA-DPB1",-1.28406911951221,6.26000242990654,-11.7339212695552,4.92256761404108e-21,2.32240090810588e-19,37.2744583471117

"APOL3",-1.28380441463416,6.00745588785047,-9.97867429138953,4.90854652211561e-17,1.16168934356736e-15,28.1453091432787

"GZMH",-1.28298891707318,7.49671644859813,-4.58954175158365,1.19823095468333e-05,4.94524324826903e-05,2.3749449313442

"CD3D",-1.27523755609757,7.94184794392523,-7.54313153040123,1.48347073055398e-11,1.52610730024448e-10,15.6675095313092

"CD79A",-1.27440943658538,6.51725817757009,-8.98335035136436,9.05621708831621e-15,1.5237947245337e-13,22.9786792734608

"SEPT6",-1.27324251024391,6.31700917757009,-15.9624929634196,2.92565996094985e-30,1.17324382322906e-27,58.3359404423354

"PAQR8",-1.27067676341465,6.59236313084112,-15.0150545954144,2.89188660532266e-28,6.26238044382623e-26,53.7855351880597

"BAG3",-1.27004946829269,5.17583626168224,-9.18766149825792,3.11499462014848e-15,5.60259206804944e-14,24.0348489858296

"KLRG1",-1.26530943414635,6.29502841121495,-9.06589085851827,5.88632148887499e-15,1.01811734697754e-13,23.4049922018149

"LOC283070",-1.26346221219514,6.84347443925234,-5.53634466142631,2.16525925574245e-07,1.17958966498372e-06,6.2625574138103

"FAM169A",-1.26230252195123,3.77838037383178,-8.82387188468913,2.07888681689253e-14,3.29804351793463e-13,22.1565893213773

"P2RY14",-1.25907319512196,5.74319878504673,-3.05950876700746,0.00279253324925078,0.00713958766381649,-2.78246146951658

"FCRLA",-1.25889778373985,4.07033267912773,-9.74013377649127,1.71964847178699e-16,3.76151390470176e-15,26.9032107680236

"SCRN1",-1.25587167804879,5.82656308411215,-11.9524953693606,1.57764321947315e-21,8.35302296276064e-20,38.4031413596734

"TAGAP",-1.24706559024392,7.61522607476636,-10.5296325975875,2.70767830688288e-18,7.93713287172953e-17,31.0170319980621

"KLRC3",-1.24560522439025,3.30051523364486,-9.42521951179545,8.97874711477998e-16,1.76278122185458e-14,25.2663582886296

"TESPA1",-1.24509522195122,5.37595247663551,-16.3820789292629,3.96201109481669e-31,2.19993205790398e-28,60.3154042929098

"C9orf91",-1.23994452195123,4.60808654205607,-10.4234069193832,4.73343384241636e-18,1.31920862107499e-16,30.4633306576741

"PEBP1",-1.23952792357725,6.32930660436137,-10.4056870070471,5.19573101558497e-18,1.43147016720728e-16,30.3709591711722

"GIMAP6",-1.2375073390244,7.61917228971963,-8.00770430048511,1.40450595129462e-12,1.67964657846378e-11,17.9930097053866

"TIAM1",-1.23366661219513,5.40652285046729,-11.5770753016289,1.11574702433971e-20,4.92087613280577e-19,36.4628353336548

"ZNF266",-1.22793183414636,7.1771153271028,-13.7133153434886,1.88619749791524e-25,2.22651711510252e-23,47.3613112504244

"PWAR6",-1.22395792195123,3.94887635514019,-8.84642981648568,1.8485771777255e-14,2.96965421243663e-13,22.2727362810418

"CD96",-1.22303773414635,3.97033934579439,-11.1509611276822,1.03694793306069e-19,3.93259325576696e-18,34.2518171812452

"TIGD3",-1.21992750243903,5.38920588785047,-12.7219071401604,2.95075406218045e-23,2.21102350230165e-21,42.3500708007149

"ECI2",-1.21826620000001,5.73579663551402,-9.75171547247099,1.61813887464583e-16,3.55023275891138e-15,26.9634817569934

"DDX24",-1.21567851707318,6.70603471962617,-16.0236451731561,2.18315523088936e-30,9.45524530498183e-28,58.6258223760699

"DHRS3",-1.21524880487806,5.7667338317757,-16.1373513500254,1.26827868113067e-30,5.72178642497598e-28,59.1635736809883

"ABHD15",-1.21521588780489,4.78509345794393,-15.5511336620812,2.1214329449668e-29,5.96618576925404e-27,56.3738016354516

"P2RY8",-1.21454020487806,7.88880327102804,-8.00767970861014,1.40468249992976e-12,1.67964657846378e-11,17.9928856168143

"PLEKHO1",-1.21161953170733,6.99493794392523,-9.47547938754824,6.89868905590352e-16,1.37181920574463e-14,25.5273072205752

"C12orf57",-1.20576008780489,7.1421391588785,-7.16621378098873,9.75291373986529e-11,8.83309690659903e-10,13.812502713467

"OXNAD1",-1.20423409756098,5.86619700934579,-10.8366971064087,5.39298568581581e-19,1.80502480720775e-17,32.6168076691358

"RRS1",-1.20248546341465,6.01468308411215,-11.7764661903709,3.94367302436819e-21,1.91051989580969e-19,37.4943772963209

"ATP8B2",-1.19662580731709,5.64140626168224,-11.3838284740949,3.06333675590719e-20,1.2492760348243e-18,35.4611163998847

"IMP3",-1.19606472682928,7.11392644859813,-12.9947830998326,7.27811871308644e-24,6.19931437927397e-22,43.7384907008822

"ZNF121",-1.19548757073172,6.47549635514019,-8.61130963992725,6.27167767726062e-14,9.17656622304585e-13,21.0645749210569

"ATIC",-1.19511210243903,6.95947289719626,-10.536205121607,2.61571147134864e-18,7.74459636947656e-17,31.05128833414

"SLAMF6",-1.19212065853659,6.47087420560748,-13.0709846234958,4.92910981765097e-24,4.37458496316524e-22,44.1250308955001

"VPREB3",-1.1920014292683,4.82612317757009,-8.92347638618499,1.23747363944607e-14,2.03938292710841e-13,22.669779792065

"HVCN1",-1.18892410243903,7.74788710280374,-11.9915988837442,1.28746755300857e-21,6.97002746510014e-20,38.6047539291484

"BANK1",-1.18749239512196,5.91143096573209,-7.99840714694849,1.47284924030423e-12,1.75630783583635e-11,17.946103905672

"GOLGA8A",-1.18622324390245,5.39397471962617,-10.1044058836654,2.53402340823947e-17,6.24991764298698e-16,28.8004750475709

"GSPT2",-1.18336927804879,4.85762327102804,-10.2856456590295,9.76855877685819e-18,2.57659123401783e-16,29.7451841422749

"LYSMD2",-1.17976305365854,8.70577971962617,-6.27631990438433,7.26129826142644e-09,4.96036005839715e-08,9.57936609366991

"GIMAP4",-1.17919801463415,8.98275514018692,-6.72042157403822,8.67836754095612e-10,6.80905974997844e-09,11.6632055749414

"GABPB2",-1.17827901463416,5.58824542056075,-15.4960648826916,2.77006021940441e-29,7.3153236647808e-27,56.1095362460139

"CRY1",-1.17753029756098,4.50443280373832,-8.64568029069588,5.24764943398808e-14,7.76745375892083e-13,21.2408366424426

"RNF125",-1.17572598048781,5.29319355140187,-10.9792725258757,2.55145615239844e-19,9.19330831617109e-18,33.3589423995749

"PDCD4-AS1",-1.17568494146342,5.16394140186916,-9.03027187204483,7.08941410360581e-15,1.21264820231899e-13,23.220958775897

"ZNF83",-1.17507616097562,4.83528794392523,-9.33919566130644,1.40925760609878e-15,2.66295579930796e-14,24.8200279824296

"FAM171A1",-1.17432138536586,4.32457757009346,-13.7109874866197,1.90849564368192e-25,2.22651711510252e-23,47.3496569956758

"ZNF831",-1.17206717073171,3.24820429906542,-15.7928579994753,6.60615897020759e-30,2.38427287499742e-27,57.5293562535939

"EMR3",-1.17011190731708,6.20211392523365,-5.20851329659676,9.10333321410747e-07,4.51621261744553e-06,4.86636446557022

"CCL4",-1.17010003414635,7.21684822429907,-6.60607912903838,1.50779653590525e-09,1.14445615089478e-08,11.1208118326902

"BCL11A",-1.16937834146342,5.71271789719626,-10.5295034750331,2.70951710629867e-18,7.93713287172953e-17,31.0163590004505

"CTSO",-1.16866845365854,6.25317252336449,-9.13635522474884,4.07340896912696e-15,7.21256510436994e-14,23.7693401227766

"ZBTB4",-1.16484082439026,6.80389504672897,-14.9371512555782,4.23917921447487e-28,8.87077457956901e-26,53.406526294157

"CXCL8",-1.15967525365855,4.06963630841121,-6.17842570366195,1.15043751115588e-08,7.60693871880324e-08,9.12872935461206

"SATB1",-1.15816038373985,7.21265757009346,-11.7400534283178,4.7677127408598e-21,2.26558676627156e-19,37.30616250254

"MLLT3",-1.15518361788619,4.15849242990654,-13.1170237496131,3.89603378142085e-24,3.53006742831249e-22,44.3583115561392

"NR3C2",-1.15343993170732,3.68864457943925,-12.2324608612264,3.68983352974552e-22,2.17128655126737e-20,39.8443511684357

"SIGLEC17P",-1.15184652682928,4.90456009345794,-11.8525550250491,2.65343701498766e-21,1.3362832223153e-19,37.8874233973741

"SACS",-1.15105169268293,3.42209401869159,-7.73408427910286,5.65442890385794e-12,6.16859737597198e-11,16.6186098687299

"KLF12",-1.15098239390244,4.39141337616822,-13.561311949115,4.0678761143578e-25,4.44898268971809e-23,46.5991504713353

"LOC102724611",-1.14805494146342,4.21995579439252,-9.96591661943268,5.24909266691143e-17,1.23661446746379e-15,28.0788456184619

"MAP4K1",-1.14449802764228,5.70734685358255,-13.1585766417728,3.1513981667565e-24,2.89167488564034e-22,44.5686918073461

"RPS6KA5",-1.14388925365854,5.15650965732087,-7.73804918480181,5.54193607461569e-12,6.06114271190923e-11,16.6384318106244

"TMEM109",-1.13872409756099,6.41830093457944,-14.440501940283,4.93552834416845e-27,8.41565876322581e-25,50.9735612419638

"PDE4B",-1.13410266178863,5.20128339563863,-8.41673408708665,1.71658933296216e-13,2.34528340727417e-12,20.0692559300854

"TRMT13",-1.13296273414635,5.39122878504673,-7.62480740496125,9.82776803663076e-12,1.03764172029858e-10,16.0734572142132

"SLC38A1",-1.1327940390244,6.17765656542056,-12.0761650237335,8.29826034806968e-22,4.57248925795036e-20,39.0404239365352

"MTR",-1.12725066829269,4.78425705607477,-10.6305209858048,1.59318749667113e-18,4.89368443126429e-17,31.5428142928038

"FTO",-1.12688086829269,6.38483065420561,-12.3005419942039,2.59373233411302e-22,1.56454801379436e-20,40.1939927303893

"SCML4",-1.12632366243903,4.9461478317757,-10.9012365679015,3.84291839347903e-19,1.33048691616778e-17,32.9528150404099

"RUNX1-IT1",-1.12623219024391,7.15970897196262,-9.93263604600479,6.25278646370632e-17,1.45283359304249e-15,27.9054791213409

"PIK3C2B",-1.12580908780488,5.93715401869159,-13.7409092860943,1.64098130854026e-25,2.00765255573104e-23,47.4994156971088

"RHOH",-1.12313898536587,7.29981906542056,-9.61326962247453,3.34792528944222e-16,7.01832740976489e-15,26.2433125717841

"LRBA",-1.12297386585367,5.22554920560748,-10.4590529926044,3.92435474905197e-18,1.11378639699502e-16,30.6491442680174

"CDKN1C",-1.11959827560977,5.23776990654206,-7.15160128369745,1.04852379403e-10,9.44500114796987e-10,13.741240529504

"HCAR3",-1.11856323902441,9.13018411214953,-3.56873379560417,0.00053530888914044,0.00162036818483872,-1.24409999937043

"UBE2Q2",-1.11678387317074,6.18162373831776,-7.35006624586604,3.90647047604881e-11,3.76981364344194e-10,14.7134020416742

"DDX11L2",-1.1108950585366,6.65562626168224,-5.83639577650296,5.59815673821143e-08,3.33594067600354e-07,7.58165942042574

"TMEM263",-1.10886031219513,5.17344,-5.78052870325897,7.22040643586239e-08,4.22360619580227e-07,7.33324002361046

"BIN1",-1.10771491512196,6.58528570093458,-15.0598017557733,2.32227418140855e-28,5.23842160400022e-26,54.0029086106359

"FBL",-1.10730011707318,7.9847576635514,-8.32325150887228,2.78040542689262e-13,3.68254920607704e-12,19.5926975332473

"SH2D2A",-1.10714889756099,5.96038355140187,-10.5813826770718,2.06272786237077e-18,6.18675510521315e-17,31.2867439093724

"OSBPL3",-1.10685471707318,4.40099359813084,-9.23965811638679,2.37312291981963e-15,4.34100174147026e-14,24.3041098578442

"HERC5",-1.10334910243903,6.46849794392523,-4.13453732607513,7.02462907527428e-05,0.000253615109411578,0.680020783019479

"RSAD1",-1.10317298048781,5.86859691588785,-13.4060179825862,8.94146807217656e-25,9.17665834611296e-23,45.8180844406184

"KIAA1147",-1.10159758048782,5.7606538317757,-12.308866601868,2.48437595888635e-22,1.50276987122022e-20,40.236721829265

"PRSS33",-1.10124343414635,4.87719023364486,-8.54376055233079,8.89994582236066e-14,1.26878424478749e-12,20.7185427304441

"TKTL1",-1.09691414390245,4.44599560747664,-11.277093957972,5.35541488014442e-20,2.12791760054179e-18,34.9071094318444

"ITGB7",-1.09629644390245,6.17979878504673,-11.6799389458151,6.52280548576822e-21,2.99261340665913e-19,36.9952703383082

"EPHX2",-1.09354412682927,3.75639803738318,-12.7611214988407,2.41212177015646e-23,1.8522871252744e-21,42.5499937414105

"PFAS",-1.09138982439025,3.85132859813084,-10.807018684813,6.30261553828509e-19,2.08053566282871e-17,32.4622616683715

"RP11-44F14.8",-1.09122211707318,4.16351112149533,-10.1929533669462,1.59058987678464e-17,4.04749985684739e-16,29.2619984180126

"OAS2",-1.09027245528456,5.21330143302181,-7.11750310910793,1.24128383285271e-10,1.10982664741641e-09,13.5751525935631

"SIGIRR",-1.08636554634147,6.50545957943925,-13.0271747607622,6.16660810182334e-24,5.40639264959451e-22,43.90286531069

"PTCD3",-1.08594900975611,5.00754329439252,-12.4906161515143,9.71274991187016e-23,6.55232396702643e-21,41.1683153997602

"SLC39A10",-1.08281619024391,5.33981504672897,-7.09580244135578,1.38183434071063e-10,1.22487198723244e-09,13.4696000869493

"CD5",-1.08190387317074,5.79352920560748,-16.4519288796898,2.84634263367595e-31,1.81286910977214e-28,60.642759182717

"GRAMD1C",-1.08066388292684,2.80702887850467,-8.31044285998848,2.97009293491468e-13,3.91701355088779e-12,19.5274894900919

"PRKACB",-1.07962368780489,5.70189803738318,-9.74240079317855,1.69929018507713e-16,3.72074104730488e-15,26.9150079569083

"LINC00954",-1.07918269268293,4.52168719626168,-11.716624572878,5.38706275618262e-21,2.5198865241336e-19,37.1850203924202

"HMG20A",-1.07911529756098,5.18156364485981,-10.4714416129451,3.67685555725667e-18,1.05181383213201e-16,30.7137211650619

"SBK1",-1.07849390975611,5.40811144859813,-14.3670575910374,7.11226749289306e-27,1.1324717099897e-24,50.6113705400788

"FLVCR1",-1.07661104390244,4.65069939252336,-6.95571154598229,2.75445349440041e-10,2.32635298054762e-09,12.7910580988975

"RORA",-1.07615494939025,4.2634846728972,-10.7707471032159,7.62541320473106e-19,2.47198088246184e-17,32.2733565341488

"SAMD3",-1.07601861707318,4.34967140186916,-10.033683195051,3.67564510331001e-17,8.85384813261161e-16,28.4319215566895

"PHOSPHO2",-1.07597005365854,3.67720401869159,-8.38407084691691,2.03187324643874e-13,2.74498653268703e-12,19.9026174368956

"CHMP7",-1.07339536585367,6.96711794392523,-14.8403030638765,6.82656931373416e-28,1.35623264668728e-25,52.9343464447451

"LOC101928054",-1.07173053170733,4.71439859813084,-10.8023077329957,6.4605072413428e-19,2.12617453360605e-17,32.4377282689396

"FAM102A",-1.07145055365854,4.85290556074766,-18.2529202238371,6.96950379337734e-35,8.8779179203286e-32,68.8653218225429

"RRAS2",-1.07064349756098,4.27897258566978,-8.376274135137,2.11528436146937e-13,2.84689141377373e-12,19.8628607549021

"BEX2",-1.07059209268294,5.02423504672897,-7.79890404686098,4.0697589843856e-12,4.54750416960114e-11,16.9430254940102

"PIK3IP1",-1.0697474585366,6.5509223364486,-11.8486768625253,2.70754948731162e-21,1.3572218552716e-19,37.8673986601942

"LFNG",-1.0690416682927,6.40267682242991,-11.424279206301,2.4792112911656e-20,1.02652620478377e-18,35.6709451853695

"CNNM3",-1.06870980000001,6.59173355140187,-10.8299001652253,5.58896102449734e-19,1.86198386131523e-17,32.5814153722904

"ITGA6",-1.06837727804879,5.22862598130841,-9.8386748467849,1.02464070359499e-16,2.32584847341189e-15,27.4161452135395

"STK39",-1.06660972682928,5.45182495327103,-7.36950638937434,3.54477530104407e-11,3.45308633126897e-10,14.8091106843405

"GBP4",-1.06644321463416,5.15517056074766,-4.56344891974634,1.33016678421796e-05,5.43896558010571e-05,2.27445203545463

"PTER",-1.0654958390244,5.73022018691589,-8.21035011363454,4.97099540422366e-13,6.37341062631518e-12,19.0186886795419

"MEN1",-1.06508658048781,5.83765822429907,-12.5327179297383,7.8166074658245e-23,5.33970456379904e-21,41.3837516778061

"ZNF275",-1.06495069268294,5.66230196261682,-12.4418057316656,1.24960284899183e-22,8.05361598063037e-21,40.918376501892

"GPR56",-1.06317450487806,5.62465495327103,-10.0417992202319,3.52207367349371e-17,8.50284341131621e-16,28.4742126001455

"GIMAP1",-1.06219314829269,6.99050770093458,-7.84865058799979,3.16050273282863e-12,3.58891907076056e-11,17.1925102952029

"PARP1",-1.06089809756099,6.66095728971963,-12.375099674696,1.76381299651834e-22,1.0975681160806e-20,40.5765036342654

"MAML2",-1.0607053097561,5.58214023364486,-8.81126289246974,2.21986238505176e-14,3.49608145078516e-13,22.0916882369934

"NFATC2",-1.05883546707318,4.89320289719626,-16.2919399876123,6.07649878994125e-31,3.17631525388623e-28,59.8920440163787

"APBA2",-1.05737221707318,5.91986976635514,-14.1776407164032,1.82991129222717e-26,2.7328778643572e-24,49.6744742299408

"PTPRCAP",-1.05641348292684,7.38071289719626,-7.56707924188357,1.31492895243385e-11,1.36177840578455e-10,15.7863974447643

"ZNF559",-1.05578467804879,5.35929878504673,-8.43285771166637,1.57942203318138e-13,2.17157994466938e-12,20.1515630792785

"CSF1R",-1.05577671707318,6.98329448598131,-7.74222112916699,5.42596899796637e-12,5.94031135748037e-11,16.6592919310587

"AMIGO1",-1.05549728292684,4.8397138317757,-13.8125104023147,1.14371763394715e-25,1.44333975903698e-23,47.8574014970902

"LOC100128751",-1.05470128780489,5.40308504672897,-9.58274442533134,3.92971638129963e-16,8.10457221305176e-15,26.0846227601002

"CRIP1",-1.05334697073173,8.27098093457944,-6.00656785702765,2.56073196129337e-08,1.60965604127164e-07,8.34589256750848

"PJA1",-1.05282974634148,6.51659411214953,-12.9458469730388,9.35040723715984e-24,7.75797198163588e-22,43.4899803710381

"PEA15",-1.05070138536587,6.13285939252336,-8.54281412030178,8.94366766727789e-14,1.27417844299278e-12,20.7136981038173

"SPIB",-1.05050370487805,4.57445448598131,-13.3155821655165,1.4160182006953e-24,1.40659973101178e-22,45.36213380428

"C6orf136",-1.0483655487805,5.2451196728972,-14.7284561854546,1.18509618187011e-27,2.29667411798961e-25,52.3876696255965

"TC2N",-1.04802526178862,3.31027018691589,-11.3771798373225,3.17175703915054e-20,1.29106012561664e-18,35.4266210435797

"PLA2G7",-1.04521053170732,3.2679223364486,-5.87968709055308,4.5926291322323e-08,2.7702892439691e-07,7.77501268771043

"ARHGEF18",-1.04510200975611,9.07651710280374,-13.5483093916147,4.34476803299347e-25,4.68089312211311e-23,46.533845270235

"GSE1",-1.04467126016261,5.20474414330218,-16.8298517610995,4.80599805312172e-32,3.58875475311555e-29,62.4030607167564

"HNRNPA0",-1.0421628390244,5.77763355140187,-16.6025226482999,1.39807396358353e-31,9.76622312303267e-29,61.3463990715719

"EEF2",-1.0413633780488,11.0691318224299,-11.5726300860001,1.14194732843346e-20,5.015997849336e-19,36.4398137476751

"DUSP2",-1.04112118536586,5.01093102803738,-10.4234542981752,4.73225463258482e-18,1.31920862107499e-16,30.4635776351021

"CD74",-1.04002549593497,6.69059644859813,-11.2530830882468,6.07292554183032e-20,2.39107641106065e-18,34.7824147523364

"BRD1",-1.03911100243904,5.90316154205607,-12.909777184517,1.12484412837008e-23,9.22670439388408e-22,43.3066718262422

"CAMK2D",-1.0388685395122,4.56152207476635,-11.0456825127874,1.80086763453317e-19,6.60979468234164e-18,33.7044243231399

"ARHGEF3",-1.03831797560977,6.85246168224299,-8.18575865059699,5.64042674358487e-13,7.17646540142951e-12,18.8938966055091

"ZNF703",-1.03649453170732,4.39885588785047,-11.8362137890292,2.8890511809339e-21,1.42836537267405e-19,37.8030399191548

"IKZF3",-1.03414431544716,5.09526778816199,-10.0501164668373,3.3713470717667e-17,8.17542226641746e-16,28.5175531987903

"MATK",-1.03268416097562,5.43522878504673,-11.893118234882,2.14846976427522e-21,1.10249082335023e-19,38.0968148607053

"TTC39C",-1.03152081138212,4.83755965732087,-10.1477987096584,2.01696534209517e-17,5.02616622359848e-16,29.0266360516673

"UNG",-1.03046865365854,4.44751785046729,-8.01199240032056,1.37405602605385e-12,1.64848660632665e-11,18.0146485733881

"ZFP3",-1.02885878048782,4.59492878504673,-11.6996021255018,5.88706477110273e-21,2.72946678563282e-19,37.0969840652409

"ZNF304",-1.02794744878049,3.22131158878505,-10.6615677711891,1.35332307706751e-18,4.19859759797949e-17,31.7045885142896

"PTGDR",-1.02582657560977,4.13068146417445,-9.85905150464677,9.20578991189763e-17,2.10508321586212e-15,27.52224525435

"FBLN5",-1.0229034487805,4.29073308411215,-9.76870349375388,1.47997575817626e-16,3.28369621345358e-15,27.0518947705904

"PARP12",-1.02115300487806,7.15643934579439,-6.43714266867316,3.38723833554954e-09,2.44992138130679e-08,10.3267453716804

"SETD6",-1.0184789195122,4.50980864485981,-12.9388047840222,9.69385994864699e-24,7.98176947482702e-22,43.4542006801541

"MAP3K4",-1.01840521463416,6.14850172897196,-11.2473736387869,6.25725416842895e-20,2.45918038143972e-18,34.7527606794925

"ICAM2",-1.01654054390245,7.87835672897196,-8.18968998316772,5.52767704282124e-13,7.04542945039988e-12,18.9138407875395

"LINC00926",-1.01614216829269,5.87413962616822,-7.5843079896109,1.20556953069596e-11,1.25512539361639e-10,15.8720003117587

"HSP90AB1",-1.01580765528457,8.09579644859813,-6.8704852254635,4.18096269572083e-10,3.45963879158711e-09,12.3807686054482

"ANKRD46",-1.01476783414635,4.48999065420561,-8.25914407180324,3.86788653636509e-13,5.0275559990988e-12,19.2665509523925

"RP11-158G18.1",-1.01158096097563,6.78589813084112,-6.56841257777509,1.80727903633804e-09,1.35985502195623e-08,10.9429958469101

"KPNA5",-1.01038246341464,4.18139753894081,-8.8269344930785,2.04601469946783e-14,3.25065651628583e-13,22.1723554263866

"CLC",-1.00965261463416,8.48585672897196,-3.19976464357048,0.00180306514095941,0.00483953589829896,-2.37812378580294

"TRIM28",-1.0070722292683,7.14385542056075,-10.3663244225842,6.3907257088907e-18,1.7407693739123e-16,30.1657637472963

"HLA-DQB1",-1.00669558211383,4.58256953271028,-5.32471871286842,5.5005248276919e-07,2.81659648010565e-06,5.35560845393929

"NAA25",-1.00668551219513,4.74549738317757,-10.4448413085492,4.22886764783613e-18,1.19395213707811e-16,30.5750633123736

"OXCT1",-1.00578464390245,5.5747961682243,-7.51267349601125,1.72912773429445e-11,1.75877224453482e-10,15.5164692921381

"AMPD2",-1.00546046341464,7.38330420560748,-7.92235251936975,2.1714451046868e-12,2.53354761540908e-11,17.5629203045078

"ATP2B1",-1.00280456422766,6.58198236760125,-6.49209785171385,2.60554437917128e-09,1.916544277546e-08,10.5840727751669

"FBXO21",-1.00223560650408,5.14990012461059,-14.7140329748157,1.27259518817807e-27,2.43876538053064e-25,52.3170665966525

"ZHX2",-1.0020920195122,5.54519742990654,-12.6746786296724,3.76210116988161e-23,2.74304043211402e-21,42.1091183518596

"LINC00877",-1.0018986292683,5.16757663551402,-5.36469009729043,4.61908222889771e-07,2.38840080388682e-06,5.5253555161191

"MYOM2",-1.00032995609757,4.12533719626168,-6.57922677046212,1.71574646443937e-09,1.29413060562294e-08,10.9940030696655

"IFFO2",-1.00019948292684,4.98091878504673,-14.6139500589309,2.08758505929493e-27,3.76722120491932e-25,51.826486205297

"PRKCH",-1.00011756097562,5.80944809968847,-13.6104810222494,3.17166799947578e-25,3.57721200670041e-23,46.8459471178924

"TREML1",1.00018409756096,7.34229822429907,4.20825516652315,5.3159827160667e-05,0.000196547047492615,0.946114459876441

"OLR1",1.00463573658536,3.19748056074766,3.84009267450436,0.0002071310796413,0.000683336917981773,-0.348083539836589

"ZAK",1.00553367108013,5.62835595460614,10.6916483064464,1.15544495887852e-18,3.61577465094137e-17,31.8613136146337

"UBE2F",1.00935873780486,7.68562525700935,9.52188026583206,5.40834909847165e-16,1.08845538780115e-14,25.7683282690715

"C9orf72",1.01189856910568,6.0127623364486,8.58665379925475,7.12671181196082e-14,1.03230063068904e-12,20.9382122350391

"GK",1.01235820365852,7.30121231308411,7.21509326936626,7.6524974149251e-11,7.04869551340719e-10,14.051250064287

"ELL2",1.01335825853658,5.10412271028037,7.90070482041794,2.42474207279579e-12,2.81241508229206e-11,17.4540280364795

"ALOX5",1.01359099756096,8.12226077102804,12.6655596831446,3.94284201124744e-23,2.84607479178544e-21,42.0625734982212

"PSMA4",1.01876913658536,8.00100336448598,6.51512176834461,2.33369591651824e-09,1.72773282298128e-08,10.6921671519338

"TBCA",1.02045747804876,7.88302523364486,8.41633589842089,1.72012282037426e-13,2.348629235511e-12,20.0672236818247

"MTF1",1.02053907642275,7.60021856697819,14.8894649284248,5.35928519955081e-28,1.08462916818947e-25,53.1741716779469

"WFDC1",1.02097184878048,4.86252822429907,4.88432574134804,3.59347936674236e-06,1.61613282838641e-05,3.53645273540971

"ENTPD1",1.02242389878047,7.37125521028037,10.0212515640149,3.92395470771221e-17,9.38930819839866e-16,28.3671447123131

"LOC645984",1.02584487804878,3.4019114953271,6.9123865190229,3.40618251090429e-10,2.85673440254192e-09,12.5822428663491

"AZI2",1.02592347886178,5.65326193146417,8.39264542322159,1.94391722198335e-13,2.63756437606826e-12,19.946349479728

"ETS2",1.02594200975609,6.17405286604361,9.37366906767408,1.17639539058368e-15,2.26443041627462e-14,24.9988433175698

"COX7A2",1.02694939512193,9.61981074766355,8.38431168674413,2.0293495053229e-13,2.74488216975436e-12,19.9038456407637

"CTSD",1.02902088780487,6.85031504672897,9.55398107370727,4.57001300090569e-16,9.30109318934331e-15,25.9351274663137

"CASP5",1.03001824390243,5.92097177570093,5.33538598239944,5.25038776567632e-07,2.696161893899e-06,5.40083704212397

"ATP5J",1.03067424390242,7.7370585046729,7.35977870184453,3.72140860567327e-11,3.60891640644221e-10,14.7612084331722

"DTL",1.03680912439024,3.97413140186916,5.25967736082975,7.29765926360768e-07,3.67001419771074e-06,5.08098694276429

"MARCO",1.03706868780487,6.45151971962617,6.59643884706135,1.57941825448286e-09,1.19598809459116e-08,11.0752608930414

"RBX1",1.03747531707315,8.24435654205607,8.13135512258015,7.45716588887901e-13,9.32361012261403e-12,18.6181302025437

"TYMS",1.03799677886178,4.83270523364486,6.40160466653046,4.0116522053766e-09,2.8642376692196e-08,10.1608565136207

"LACTB",1.03885615447153,6.61422208722741,9.07947911457978,5.4830562316301e-15,9.5600308128784e-14,23.475224267521

"ZNF608",1.04031879756097,4.44357845794393,5.17547221849084,1.04939299912518e-06,5.15063585586033e-06,4.72843122455692

"PRO2852",1.04052698536584,8.37798280373832,10.1501518688401,1.99215688922362e-17,4.97069550776594e-16,29.03890113312

"GNS",1.04055686341462,8.37678570093458,8.8732456772539,1.607665098713e-14,2.60583740363997e-13,22.4108667217042

"GALNT2",1.04074287195121,6.55806214953271,6.48927650764495,2.64093597174534e-09,1.93854553771272e-08,10.5708384214382

"CDKN2C",1.04108174634145,5.49269074766355,8.97174347374861,9.62144910228829e-15,1.61638852063656e-13,22.9187743403492

"EMILIN2",1.04580271382113,5.89719068535826,9.06788818508075,5.82524127918356e-15,1.00916479920576e-13,23.4153146889286

"TTK",1.04849321463414,3.43112336448598,5.28759315841272,6.46523803176934e-07,3.28418319441626e-06,5.19861155673637

"UBE2C",1.05042070731706,6.56100130841121,8.20808492781318,5.02919977884969e-13,6.43660290845095e-12,19.0071901502688

"DIRC2",1.05153923902438,6.26543070093458,10.9771807697986,2.57961519106408e-19,9.26394145314969e-18,33.3480583826253

"NDUFA4",1.05311845365852,8.80947514018692,6.48946053464143,2.63861309751112e-09,1.93757770859963e-08,10.5717015780284

"ALPK1",1.05433587682926,7.07430401869159,11.2410382230959,6.46835821978249e-20,2.53754161683677e-18,34.7198539060525

"IL18",1.05439628780487,4.31059121495327,10.6212399877934,1.67283297561156e-18,5.11655340210006e-17,31.4944514579814

"NFIL3",1.05484607317072,8.98729271028037,9.59001868098782,3.78250942177084e-16,7.83830062473183e-15,26.1224356291976

"CA1",1.06314969512194,6.00717023364486,5.47110705479955,2.89194271955664e-07,1.54820814813347e-06,5.98083168860641

"AGTPBP1",1.06596768292681,7.72945481308411,8.36114166475776,2.28702694170067e-13,3.06849866310583e-12,19.7857200256324

"SIGLEC9",1.06761828780487,7.11867196261682,11.6162875421198,9.09211388612125e-21,4.07639184687279e-19,36.6658692907017

"MZB1",1.06984636829268,5.9123376635514,3.3225441170745,0.00121588277742073,0.00339172247134431,-2.01190141032757

"AGTRAP",1.07081299024388,8.277176105919,9.7329309748453,1.78596220341753e-16,3.898690676916e-15,26.865729646355

"DPH3",1.07083128536585,6.72362985981308,9.60168644838878,3.55780759477278e-16,7.40810802546197e-15,26.1830912701953

"HMGB2",1.07267261463414,6.58387397196262,14.3907202399795,6.32203928370167e-27,1.02935158412451e-24,50.7281293104423

"C14orf2",1.07315042195121,8.01889154205607,8.08070174581458,9.66762254671887e-13,1.18680479733105e-11,18.3617670812168

"IGJ",1.07607988780487,8.68651495327103,2.73076457046135,0.00737433193938079,0.0168557270579788,-3.66965258596043

"PET100",1.07659319512194,7.32180672897196,8.77737055318499,2.64783380352911e-14,4.11031118390129e-13,21.9173114937006

"RP11-373D23.2",1.07815160487803,7.49469822429907,7.52223970032621,1.64791183600036e-11,1.68407412971155e-10,15.5638873241598

"CAPG",1.07903299024389,8.66513887850467,9.20812308229479,2.79882881086593e-15,5.06761186449012e-14,24.1407868356338

"CETP",1.07905204878048,4.32704308411215,7.09718233453621,1.37244566482771e-10,1.21754653305384e-09,13.4763084453973

"SEMA4A",1.08011811219511,7.36792546728972,12.2098618385822,4.14814106282742e-22,2.39541319241407e-20,39.728216255085

"LMO2",1.08077990731706,6.87707803738318,8.15893955977209,6.4730867969338e-13,8.15917896318984e-12,18.7578991848826

"FAR1",1.0822916520325,7.50265470404984,8.39676835406256,1.90298524217186e-13,2.58526633746748e-12,19.9673805525163

"FKBP9",1.08268274634145,5.82023588785047,9.26079368641089,2.12461873268229e-15,3.9223033807532e-14,24.413608430557

"HIST1H2BE",1.08294839024389,6.55556906542056,9.03900582961439,6.77344332886037e-15,1.16135324850729e-13,23.2660757119503

"BIK",1.08430881463413,6.05028523364486,9.39469173252172,1.05367953823112e-15,2.04091506264713e-14,25.1079209580377

"PNPLA1",1.08595674146341,4.28699813084112,9.44553312169966,8.07168673100834e-16,1.59182541517151e-14,25.3718112767448

"ANO10",1.08816388292682,5.77022878504673,11.1600315443429,9.88807156383672e-20,3.76982728371275e-18,34.2989604307908

"GSR",1.09128861463413,7.21082443925234,10.7886536418381,6.94086043739925e-19,2.26703367680062e-17,32.366618629858

"CEACAM21",1.09200183414633,6.39595901869159,8.52419180071205,9.84882385250758e-14,1.39578717621762e-12,20.618394452972

"NCAPG",1.09299056341463,3.5063123364486,5.74923090817376,8.3224868494008e-08,4.83043293282697e-07,7.19462077662589

"HPSE",1.0930221219512,7.17678612149533,6.58084578610464,1.70244288660287e-09,1.28454357872422e-08,11.0016425523155

"PIWIL4",1.09610582439024,4.87542953271028,6.98107838534829,2.43187043222724e-10,2.07412974438286e-09,12.9135497658742

"UBL5",1.09724297560974,8.15340579439252,10.0561403454623,3.26622366309689e-17,7.956138742898e-16,28.5489438480421

"CDC42EP3",1.09887607195121,8.95801712616822,12.6599757044828,4.0577927203591e-23,2.91931898203908e-21,42.034068396614

"C1QC",1.10046928292682,5.12518869158878,5.79609226752886,6.72704759921383e-08,3.9542403843913e-07,7.40231937807236

"TNFRSF17",1.1014193804878,5.15210280373832,2.71702050346661,0.00766688669793071,0.0174635985530335,-3.7048481770074

"COL17A1",1.10337985853658,4.84853962616822,5.93078797521443,3.63233324414317e-08,2.22638484013361e-07,8.00418955330235

"DDIAS",1.10339354634146,5.75705691588785,7.68378975247826,7.29443190609109e-12,7.80439342521752e-11,16.3674233986931

"CORO2A",1.1033947609756,5.53402504672897,8.89350613883955,1.44664349103293e-14,2.35424335482591e-13,22.5152723295037

"SGMS2",1.1045357707317,5.27660573208723,7.81278845469685,3.7926018756843e-12,4.25317419046833e-11,17.0126135865664

"KLHL8",1.1052720211382,6.95207102803738,8.73547591061864,3.29210092115825e-14,5.05247664405967e-13,21.7019155571954

"GK3P",1.1092985414634,6.53873943925234,7.18323774847389,8.96345051951107e-11,8.17277983157946e-10,13.8955900070332

"LOC102724387",1.11138898536585,4.14247626168224,4.75120436867676,6.22389199169386e-06,2.69287474685576e-05,3.00606396069345

"PTTG1",1.11395720487804,7.41016242990654,6.77114979544628,6.78437922438363e-10,5.42124472708589e-09,11.9050628960815

"CHMP5",1.11429704878047,7.56145327102804,5.90845329181535,4.02497210748485e-08,2.44490241199395e-07,7.90389844758807

"CSF2RA",1.1149040414634,6.87258974299065,12.2564960515442,3.25796469499437e-22,1.94852450860753e-20,39.9678261971691

"C2orf76",1.11543632682926,4.72287196261682,7.4408484283278,2.48000724062123e-11,2.4760053847696e-10,15.1610517098225

"AMPH",1.11580770243902,4.7895061682243,6.72882674749712,8.33185611172324e-10,6.55857303160185e-09,11.7032278399897

"IFI27",1.11720421463414,5.86960439252336,2.49835360586996,0.013974344582111,0.0296042293020558,-4.24375681229711

"BAZ1A",1.12354667560974,9.26346257009346,9.64276167878301,2.86769345124858e-16,6.08822565556746e-15,26.3966664923726

"HIST1H2AE",1.12838430731706,4.78472551401869,6.99309807785354,2.29236971000891e-10,1.95978152665784e-09,12.9716492860455

"BEX1",1.13089853170731,4.25501710280374,5.4442432398889,3.2562781883779e-07,1.72999764890391e-06,5.86536983067831

"PPP4R2",1.13113744715446,6.34010433021807,9.50339755234521,5.95899537584371e-16,1.19483374873977e-14,25.6723111213849

"PHTF1",1.13370706707316,5.38121373831776,12.353445753859,1.97275507952975e-22,1.21363668315957e-20,40.4654531493274

"MMADHC",1.13473139024388,9.82876523364486,10.0043264924489,4.28918710964249e-17,1.0195647295204e-15,28.2789581725622

"EMB",1.13493068292681,8.29697196261682,9.60366991288434,3.52095764144436e-16,7.34550459783021e-15,26.1934029862265

"ARL4A",1.13763611219511,7.51087168224299,9.06559134054768,5.89553596610538e-15,1.01889729725468e-13,23.4034442732948

"TIMP1",1.13836079999998,10.6266678504673,9.28217285785537,1.89966652603836e-15,3.53109687737002e-14,24.5243971956164

"ZBTB8OS",1.14206494634145,5.61184654205607,8.02573286382035,1.28083792925767e-12,1.54349167268085e-11,18.0840061536201

"PDCD10",1.14383490243901,8.76119775700935,6.70762480209194,9.23346117147335e-10,7.21582828106299e-09,11.6023115076756

"TOP2A",1.14404522439024,3.81651373831776,7.28474736195904,5.41144996705696e-11,5.09721396418523e-10,14.3924395581055

"TMEM45A",1.14472673658536,3.14399401869159,5.6145431088557,1.52712827348898e-07,8.51440853820904e-07,6.60269749977552

"POR",1.14513182439023,6.92937485981308,9.77754462987898,1.41280024788029e-16,3.14755034648639e-15,27.0979113526111

"RP2",1.14609739999999,6.08420523364486,8.70551378932951,3.84657852230768e-14,5.84544967723318e-13,21.5479756234575

"PLP2",1.14670268292681,10.5574814953271,9.28687173578064,1.85350376012539e-15,3.44980219187832e-14,24.548750929315

"LGALS1",1.14801656097558,10.6060343925234,8.67235492995171,4.56927055560814e-14,6.84758158350825e-13,21.3777177160268

"ITGA2B",1.14803557317072,5.58054446261682,6.7597559272706,7.17055344472995e-10,5.70456777537205e-09,11.8506760939721

"NRN1",1.1502615902439,4.32188168224299,7.30334935853302,4.93227645242003e-11,4.67241579677482e-10,14.4837475623132

"FAM126B",1.15032664024389,7.58515523364486,9.50896961873979,5.78734098314164e-16,1.16149090815507e-14,25.7012561923193

"HIST1H4H",1.15574057804877,5.34032453271028,6.76639403216767,6.94300079729089e-10,5.53166601417713e-09,11.8823575300131

"FLOT2",1.15579241707315,8.74849985981308,11.8394614520134,2.84061198388571e-21,1.41410235657575e-19,37.8198116052087

"POMP",1.15639283170731,6.38121299065421,11.0841813224487,1.47162642951018e-19,5.44753338992187e-18,33.9046412537039

"FLOT1",1.16186343658535,8.05728511682243,13.7144487632682,1.87543553175975e-25,2.22651711510252e-23,47.3669854356041

"ALOX5AP",1.16231092682925,12.474414953271,12.110260545798,6.95248557090713e-22,3.87033611922864e-20,39.2159427421466

"CD58",1.16307055414633,6.65877917757009,10.9600128571841,2.82280232915386e-19,1.00870931415556e-17,33.2587239328113

"HEPACAM2",1.16659426341463,4.09995271028037,4.30585584791789,3.65816937917601e-05,0.000139394083945199,1.30359050181564

"PDZD8",1.16724887439023,5.74457607476636,10.5963316275632,1.90683877499902e-18,5.77518792623828e-17,31.3646494318951

"KBTBD7",1.16915068292682,6.02308663551402,5.81422598429095,6.19386669046004e-08,3.66470445852219e-07,7.48292930469008

"SIGLEC5",1.17455495609755,9.22691102803738,11.4660515353564,1.99281301150226e-20,8.44508136283393e-19,35.8875497893681

"B4GALT5",1.17747028780486,8.92024060747664,11.178607650326,8.97086954610021e-20,3.48143691793548e-18,34.3954998411598

"GJB6",1.17929853170731,3.71565869158879,4.65791971395489,9.09758899576963e-06,3.82625337611099e-05,2.64011574883176

"KIF1B",1.18437767195121,6.12937511682243,14.0037528472775,4.37213239744543e-26,5.99231183966334e-24,48.8109095610133

"SH3GLB1",1.18734551707316,9.34165180685358,12.9947566952029,7.27910227850977e-24,6.19931437927397e-22,43.738356669345

"TSPAN2",1.19063506178861,6.49957143302181,7.40318181001415,2.99512893487256e-11,2.9441451241337e-10,14.9750993751821

"GCA",1.19211596097559,10.7667957943925,9.94185174363881,5.95706248626688e-17,1.39610593225227e-15,27.9534836570304

"SRPK1",1.1931015317073,8.26958869158879,14.8964476836172,5.17832566361864e-28,1.05789285137417e-25,53.2082123875879

"H2BFS",1.19393580487803,8.4575908411215,9.48374526773319,6.6060145249073e-16,1.31482761522856e-14,25.5702354565638

"CYYR1",1.19689612195121,3.6386964953271,6.20558643665427,1.01286223382157e-08,6.76960854117473e-08,9.25342330779368

"ANKRD55",1.19937435121951,6.71007429906542,6.00541918418575,2.57437607204635e-08,1.61729370003376e-07,8.34069679606638

"SLC40A1",1.19957639756097,7.68038373831776,13.5389703356432,4.55524968564394e-25,4.88336296745641e-23,46.4869294629576

"ANXA1",1.20424039999998,8.40461042056075,8.43024631969911,1.60087300488512e-13,2.1982818592763e-12,20.1382303862124

"TMEM167A",1.20429577804877,8.49709985981308,10.6868452380581,1.18497908333016e-18,3.68688535194176e-17,31.8362897452424

"FAR2",1.20506422926828,5.81271143302181,8.94379943672008,1.11307877989283e-14,1.84561416375032e-13,22.7745961859955

"HIST1H2BH",1.20534968780487,6.65769672897196,9.76291908296702,1.52564367155693e-16,3.37464899975131e-15,27.0217891959825

"CPEB4",1.20544470365852,8.22007614485981,14.6591140218356,1.66948152295497e-27,3.08996772475128e-25,52.0480117580497

"MARC1",1.20721497560975,8.49475140186916,9.51130293054981,5.71693547456532e-16,1.14842521059102e-14,25.7133774122778

"SPTLC2",1.20784098341461,6.84705562616822,11.3594219729649,3.48055702067625e-20,1.41145060454577e-18,35.3344776390379

"LOC100505812",1.20819351951218,6.92853051401869,10.5227445488685,2.80753391745838e-18,8.19368557716459e-17,30.9811306899598

"CDK5RAP2",1.20877548943088,6.43326890965732,11.8370755574155,2.87611770602995e-21,1.42836537267405e-19,37.8074903531992

"NDUFAF1",1.20973738536585,6.3456276635514,11.0478611005005,1.78040691778943e-19,6.54579147788286e-18,33.7157555746306

"CLIC2",1.21912468780487,4.75805327102804,4.82288070617739,4.63547306634823e-06,2.04400670437326e-05,3.29046427862848

"NQO2",1.22699164390243,7.71387995327103,12.9810330941956,7.80878406528016e-24,6.55423329200162e-22,43.6686863199729

"CARD16",1.22998154390243,6.12542387850467,9.31477097257904,1.60157878442505e-15,3.01584248493257e-14,24.6933765560083

"FTX",1.2306811512195,6.58769962616822,8.15990749032675,6.44100927797888e-13,8.1234744271772e-12,18.7628056671013

"OAT",1.23269606829266,9.1549861682243,8.59916367105758,6.67928752677181e-14,9.71390002634275e-13,21.0023177571791

"LOC101926918",1.23297501951218,7.52716794392523,8.47914684881411,1.24334999183204e-13,1.73484175728884e-12,20.3880341494652

"ZWINT",1.23520873170731,6.06282056074766,5.10076630359171,1.44456921529144e-06,6.91929802192795e-06,4.41852319947197

"SLC25A40",1.23597087560975,7.30234523364486,7.1696557071726,9.58792995007817e-11,8.68730640455827e-10,13.829295787321

"GLRX",1.23870239512194,9.15339574766355,9.36755999590949,1.21466089500878e-15,2.32980351474006e-14,24.9671504449591

"HIST1H4D",1.2387805707317,4.55920345794393,7.86066018198235,2.97317017283138e-12,3.39399051621843e-11,17.2528046132857

"ROPN1L",1.24167673658535,6.84029714953271,13.7105817558744,1.91240906677012e-25,2.22651711510252e-23,47.347625675403

"WDFY3",1.24174316341462,6.88319205607477,12.8611788363123,1.44314899046906e-23,1.16125547608119e-21,43.0595108521169

"GNG10",1.24542945853657,10.7841104672897,9.9330531863295,6.23908909829984e-17,1.45120810336931e-15,27.9076519655047

"TMEM260",1.24705582682926,7.39746724299065,11.6992194388275,5.89882454710765e-21,2.72946678563282e-19,37.0950047080745

"GMNN",1.25022792682926,5.45202859813084,6.22624773487889,9.19186753922481e-09,6.17783648547217e-08,9.34845196721817

"MCTP1",1.25164274471544,6.81167498442368,12.2129249521457,4.08282454374958e-22,2.37670874986283e-20,39.7439595345642

"SNX3",1.25584998170729,10.684591588785,11.4715601396461,1.93625108897459e-20,8.22147398661663e-19,35.9161077146169

"RP11-6I2.3",1.26004913170731,5.16734672897196,6.1179695054372,1.52616522766363e-08,9.93958135490406e-08,8.8521183591963

"PROS1",1.26147450731707,5.3689246728972,6.3681869060225,4.70180862341341e-09,3.31006715669758e-08,10.0052413934872

"RP11-173M1.8",1.26256261463413,6.23506214953271,9.41947600589267,9.25319100733466e-16,1.80846436158693e-14,25.2365461370021

"GMFG",1.26669459024389,10.2710227102804,10.0152658997032,4.04942474555914e-17,9.64689690484963e-16,28.3359564119488

"MAB21L3",1.27213569756097,3.61833813084112,5.66607404802584,1.211646767348e-07,6.87944697087599e-07,6.82826947723536

"MAOA",1.2721495512195,4.72713408099689,6.25186055225281,8.14841884183036e-09,5.52974020745335e-08,9.4664606186973

"RAB27A",1.27301384682925,9.08318078504673,11.9309373422241,1.76478778394501e-21,9.25338485746471e-20,38.2919492060031

"PRC1",1.27369172682926,4.39874140186916,6.06374997172384,1.96437690477194e-08,1.25779366862319e-07,8.6051656097308

"MEF2A",1.27523224146341,6.15971738317757,10.2781900916427,1.01592212040254e-17,2.66664163846266e-16,29.7063185285888

"MGST1",1.2768147707317,6.09040796261682,12.7871222360726,2.11053226422342e-23,1.64401353171792e-21,42.6824788530743

"NABP1",1.27929696585364,7.66507087227414,8.14482311323958,6.95938108496127e-13,8.74668586156914e-12,18.6863578690569

"DSC2",1.2824346012195,6.09420644859813,6.11255269873343,1.56521104391138e-08,1.01755164082561e-07,8.82739841723635

"CEBPD",1.28783082926828,8.33417677570093,12.5976653946273,5.59283937258234e-23,3.90686892300873e-21,41.7158126056438

"LTB4R",1.28810889430894,7.57697420560748,12.430036163476,1.32791630019348e-22,8.48260397660468e-21,40.8580814955366

"MILR1",1.2932153609756,5.81831943925234,9.45759401281769,7.57703970748495e-16,1.49982445032529e-14,25.4344321434015

"WIPI1",1.29573757073169,7.29933074766355,9.60119317144102,3.56703163338449e-16,7.42017963697802e-15,26.180526826489

"CKLF",1.29855439756096,8.70598303738318,14.3779828545285,6.73577999409163e-27,1.08853220725414e-24,50.6652869753786

"FABP5",1.29911450731706,4.87459028037383,7.41503448346076,2.82252189209912e-11,2.7922207205759e-10,15.0335807137446

"NSUN7",1.30018723414633,4.14829257009346,12.6665855342856,3.92208197586852e-23,2.84055803302451e-21,42.0678099836717

"CTNNAL1",1.30217191707316,5.43062654205607,5.66243854510042,1.23163229469354e-07,6.98376468750682e-07,6.81231869108417

"PECR",1.30313158292682,6.19858518691589,11.9370628777832,1.70945665771155e-21,8.98502036959794e-20,38.3235465351983

"BLOC1S1",1.30351696097559,8.46622252336449,11.6208607196317,8.87767115898949e-21,3.98850557983231e-19,36.6895431135981

"SLC22A15",1.30552219512194,7.30124364485981,11.6020799750762,9.7920061433731e-21,4.34520272612181e-19,36.5923143677156

"C9orf84",1.30820314878048,4.37586060747664,10.8102114947298,6.19780735950045e-19,2.05219447048903e-17,32.4788887060196

"KIF11",1.30856302439024,3.57819,6.09336594275268,1.71158987973556e-08,1.1058055136251e-07,8.73992405368916

"PPP1R3D",1.30913739756097,7.86101037383178,13.410341228852,8.74727509805548e-25,9.06326517934887e-23,45.839860968084

"GSTO1",1.31201955853657,9.51471953271028,10.978376022422,2.56348692367172e-19,9.22131384254338e-18,33.3542776463004

"AGPAT9",1.3152881414634,8.87173102803738,11.1652351503491,9.62206315434046e-20,3.68137416267213e-18,34.3260046806496

"IFNGR1",1.31825755609754,9.71089732087227,14.7030110166007,1.34381192580827e-27,2.55265326784019e-25,52.2630967211691

"DYSF",1.31997084878047,9.74305364485981,13.4394590708817,7.54512494613208e-25,7.8552731109851e-23,45.9864818986102

"CTD-2165H16.3",1.32516448780486,6.97367261682243,9.54626513964915,4.75883952937137e-16,9.64912640529373e-15,25.8950305150146

"FAM105A",1.32529855284552,5.90960535825545,10.4662156991667,3.77929521088227e-18,1.07543545061308e-16,30.6864806988785

"CCPG1",1.32809119999998,7.91830831775701,10.2731469868773,1.04322946638316e-17,2.73169698845554e-16,29.6800290833761

"SMPDL3A",1.3292122707317,4.65168705607477,7.31877962405622,4.56691362278558e-11,4.3528395467175e-10,14.5595466508269

"BPGM",1.33026414634145,7.43741476635514,4.48270191499304,1.83360145477166e-05,7.32865254763386e-05,1.96593581825773

"NME8",1.33176390731706,6.23472691588785,8.83530204863737,1.95882080270131e-14,3.1235835406846e-13,22.2154354587926

"TPST2",1.3349154926829,9.27381214953271,15.8273029190736,5.59757846508148e-30,2.12658880107613e-27,57.6934272505012

"QPCT",1.33872714634144,10.4465224299065,12.4157315681045,1.42975505397791e-22,9.07957351727026e-21,40.78478528332

"RP11-330O11.3",1.34252799512195,4.35329242990654,10.2395432197213,1.24490923271419e-17,3.22856400412285e-16,29.5048566264876

"SUCNR1",1.34642307317072,5.16147224299065,5.91252388770506,3.95042924422118e-08,2.40231803660797e-07,7.9221625737789

"EMR1",1.34734678048779,7.99864813084112,7.21395411714834,7.69592046147603e-11,7.08567846910133e-10,14.045679520859

"C1QA",1.35092025365853,5.40244028037383,5.99018202780036,2.76227504550994e-08,1.7268206151997e-07,8.271821796624

"C8orf88",1.35350694634145,5.40226420560748,9.15968702310668,3.60568101571196e-15,6.43171518906445e-14,23.8900592782087

"PADI4",1.35376228455283,6.83530401869159,10.6962009153082,1.12813122751083e-18,3.54567223973105e-17,31.8850322351977

"VNN2",1.35477416585363,11.2783693457944,10.9577366788734,2.85672321737261e-19,1.01914895011868e-17,33.2468790426305

"OSCAR",1.35699264878047,7.07804869158879,12.6356564798868,4.59893901422546e-23,3.27598764319251e-21,41.9098935600175

"SHCBP1",1.35719208292682,4.05501672897196,6.30838318902766,6.24119519102072e-09,4.30286793573874e-08,9.72768132579468

"SERPINB2",1.35767300487804,5.63734448598131,5.86114625317593,4.99945939081942e-08,3.00314266596933e-07,7.69211275992003

"IDI1",1.35775161300812,7.73536246105919,9.66487061259781,2.55338130860013e-16,5.46918617583935e-15,26.5116510165087

"MCTP2",1.35891702341462,7.62100048598131,14.4339144281335,5.09982350204021e-27,8.62786546380319e-25,50.9410998177685

"CSTA",1.36027788292682,9.51183074766355,8.84994168039991,1.81508605003965e-14,2.92670799803489e-13,22.2908224558442

"EXOC6",1.36574683902438,6.15836271028037,13.6977314530288,2.04061298984907e-25,2.36307349172095e-23,47.2832809128089

"ITGAM",1.36591058536584,10.0382985981308,14.1636442786468,1.96256723198205e-26,2.891115197862e-24,49.6050871194409

"HAT1",1.36647319512193,6.81679775700935,6.87393947961298,4.11098503039887e-10,3.40433578712381e-09,12.3973597065628

"LINC01127",1.36824354634145,6.71712607476636,7.05531042126489,1.68753939378462e-10,1.46879684776551e-09,13.2729596643149

"MANSC1",1.37094266829266,8.82522056074766,13.3799785427924,1.02061579965147e-24,1.03762606297899e-22,45.6868830036961

"NDUFA1",1.37194810731706,7.69954308411215,11.7569184972657,4.36653460316815e-21,2.09197581485855e-19,37.3933462696408

"NDUFB3",1.37230394634145,7.29225738317757,11.7377977568101,4.82409911491113e-21,2.28091411208298e-19,37.2945006011084

"RAB32",1.37828537804877,7.05719808411215,13.7603662562656,1.48756529418501e-25,1.84075579689008e-23,47.5967480628829

"XK",1.38431569756096,6.57732785046729,4.48293430190026,1.83191755509932e-05,7.32327388880854e-05,1.96681831364488

"ACSL1",1.38465390731706,11.4512644392523,13.9893884663131,4.69897757560218e-26,6.39977103142549e-24,48.7394263356508

"GCLM",1.38539275772357,5.93070246105919,8.64513551989973,5.26250211706342e-14,7.78411771482297e-13,21.2380419292065

"SERPINB10",1.38577585365853,3.86015644859813,4.97374863734489,2.47266979298843e-06,1.13878486531613e-05,3.89798051405919

"MAN1A1",1.38646711951218,7.23869780373832,10.1772707131652,1.72734922730411e-17,4.35964423278211e-16,29.1802528884789

"LAMTOR5",1.38666347073169,9.38913387850467,12.4095846547361,1.47588914018555e-22,9.31789484860586e-21,40.7532839036562

"KIAA0101",1.38748524878047,5.19558925233645,8.58377508530985,7.23382611499482e-14,1.04435693159807e-12,20.923463013671

"VMP1",1.38856343414633,7.47898392523364,8.94503245941881,1.10594615928703e-14,1.83519264975943e-13,22.7809566367978

"DRAM1",1.39039924390243,7.51759560747664,12.7441472366231,2.63197657241708e-23,1.99284799565356e-21,42.4634714067919

"KCNJ2-AS1",1.39133560487804,3.77656439252336,7.41714415159597,2.79285093178102e-11,2.76665996924602e-10,15.0439930364375

"UBE2J1",1.3925564113821,7.48576123052959,13.8778551683018,8.2306646985038e-26,1.09346652789018e-23,48.1836380838022

"NUSAP1",1.39791264634145,5.74781154205608,7.61240368131796,1.0462757400024e-11,1.09981014193344e-10,16.0117229557165

"PGM2",1.39921161463413,7.35896314641745,10.525260603791,2.77063873747496e-18,8.09692062888261e-17,30.9942447111433

"CDKN3",1.40287404634145,4.3898373364486,7.03592335945823,1.85674691505605e-10,1.60702855497757e-09,13.1789570032983

"ACSL4",1.40868920325202,6.00964221183801,12.7667451742567,2.34342347354649e-23,1.80711616923846e-21,42.5786537121048

"CKAP4",1.41039689918698,7.24637171339564,12.8782477845992,1.32218367336696e-23,1.0723553350847e-21,43.1463435568929

"CTSG",1.41079119512194,5.55865308411215,4.69848728014431,7.71731922157376e-06,3.28133806682073e-05,2.79867047448899

"VSTM1",1.41386625365853,6.00466327102804,7.69550584476511,6.87453867610666e-12,7.41375174457618e-11,16.4258949326645

"ZNF438",1.41498196341462,6.47255570093458,12.6529160088795,4.20794690637977e-23,3.01732086945874e-21,41.9980263615846

"YOD1",1.41509979512194,6.64434140186916,6.84569252142958,4.71901241449704e-10,3.86206401496347e-09,12.2617828041783

"HMMR",1.41650359512194,4.63968841121495,6.94831026716759,2.85631356751225e-10,2.41050157071231e-09,12.7553506015483

"CNIH4",1.4236599707317,7.43079400311527,18.1376119655117,1.17234304354379e-34,1.41039381155226e-31,68.3515277361621

"PLAC8",1.42536266341461,11.5117228037383,7.20311036116269,8.12169240455511e-11,7.45234106019665e-10,13.9926680563357

"JAK2",1.43527797398373,6.87379971962617,13.7705874526587,1.4128288398562e-25,1.75832232914287e-23,47.647863224685

"BC043356",1.43578747804877,4.91216551401869,11.5858027591482,1.06604607429392e-20,4.71127096710914e-19,36.5080314962903

"IL1R1",1.43843589999999,5.55267934579439,10.690090138206,1.16494452794744e-18,3.63206831430148e-17,31.8531956386269

"HIST1H1C",1.43918784390242,7.86875140186916,7.35320515553023,3.84568454560252e-11,3.71944166302021e-10,14.7288500142735

"HIST1H2BD",1.44957854878047,8.26190397196262,10.1960606915961,1.5648060164931e-17,3.99126905620236e-16,29.2781955006984

"DKFZp667F0711",1.45218669756097,4.93959514018692,9.53980669149228,4.92286924605784e-16,9.97237918834261e-15,25.8614702662663

"CHIT1",1.45337830243902,5.40555953271028,6.73714087266613,8.002562505176e-10,6.31543334728813e-09,11.7428366415865

"mir-223",1.45482296585364,9.86265345794392,10.1271946324636,2.24781273548558e-17,5.57575999850403e-16,28.9192462663002

"EMC2",1.45840509756097,6.17740224299065,7.52901929357705,1.59265760369079e-11,1.6299149531155e-10,15.5975039480826

"G0S2",1.45982161951218,6.47689140186916,6.03134360507662,2.28316400677367e-08,1.4444030548257e-07,8.45808051791098

"NEDD4",1.46100273658536,5.29871775700935,7.57727635556353,1.24906738561399e-11,1.29853836944171e-10,15.8370557422399

"PLBD1",1.46424511219511,11.6665061682243,17.3966019622436,3.45097331177992e-33,3.11378446110809e-30,65.0083058769713

"WSB1",1.46585520780487,7.76278095327103,11.7662784233159,4.15866910141711e-21,2.00570110002645e-19,37.4417252249864

"PYGL",1.46691775609755,10.0602619626168,14.6867337748029,1.45637501641025e-27,2.74241747655339e-25,52.1833681305732

"LDHA",1.47194883902437,11.0955991588785,10.5306146033125,2.69373462139512e-18,7.91490138755921e-17,31.0221502892789

"ACN9",1.4763339414634,5.5635285046729,8.50773393014082,1.07244136909944e-13,1.51196079738596e-12,20.5342011757447

"PCMT1",1.4771236113821,8.57327753894081,11.9608431281395,1.51063962517471e-21,8.07725952670578e-20,38.4461895502954

"HGF",1.4777063404878,4.67381528971963,7.7859364929601,4.34675299714346e-12,4.824650751058e-11,16.8780636454939

"CD63",1.48176236097559,11.1907414018692,16.4123264160593,3.43310470491061e-31,2.06510784402331e-28,60.4572370022721

"GAS7",1.48278017170731,7.90502276635514,13.1031165768725,4.1828209605237e-24,3.7741244958392e-22,44.2878644082367

"SIPA1L2",1.48533622926828,6.52904065420561,11.1267224872534,1.17742633436879e-19,4.41127461431768e-18,34.1258229753489

"ENTPD7",1.48892499999999,5.5633903271028,7.06582481534608,1.60225412613021e-10,1.40302519617265e-09,13.3239809197864

"CENPW",1.48963196585365,5.42717551401869,8.26850099474709,3.68605912190145e-13,4.8143311390094e-12,19.3141196580253

"CKS2",1.49105250731706,5.67468495327103,6.04203301808427,2.17275555439371e-08,1.3789865630245e-07,8.50655431505768

"AGFG1",1.49384573951218,7.0379368411215,13.3428220288202,1.23280350167071e-24,1.23594258466108e-22,45.4995528313261

"CHPT1",1.49420458048779,7.45268056074766,13.8143640124413,1.1330863980628e-25,1.44333975903698e-23,47.866661918764

"LY96",1.49553065853657,8.94541560747663,8.35969449118688,2.30416137303508e-13,3.08575229023344e-12,19.7783443155826

"ITGA7",1.4957527804878,5.5585538317757,8.58428909300235,7.21458342723401e-14,1.0429359420344e-12,20.9260964880467

"F5",1.49723768455283,7.05347339563863,10.6899694723649,1.16568343497554e-18,3.63206831430148e-17,31.8525669741191

"CD55",1.49765474796746,10.6479107476636,12.9700814192698,8.25909896732933e-24,6.90543583542535e-22,43.6130760067704

"ERLIN1",1.50130412926828,7.2802573364486,8.81592758478137,2.16663091316941e-14,3.42220221916e-13,22.1156966456253

"STOM",1.50625537398373,7.4369545482866,12.4613422003609,1.12969910313059e-22,7.48123366308655e-21,41.0184373366555

"ATP9A",1.50800998780487,5.49796420560748,9.62607503304706,3.1302600895221e-16,6.6132470476684e-15,26.3098944972516

"S100A9",1.51547692682925,13.4806495327103,23.4292875734623,2.73783688629237e-44,2.96439288863306e-40,90.18615844611

"TSPO",1.51568886829266,10.9771463551402,14.6616340287379,1.64880310409438e-27,3.07800269130722e-25,52.060365242009

"PDGFC",1.51873350731706,5.15791242990654,7.22329181146864,7.34706135365617e-11,6.7817823364631e-10,14.0913505931737

"EXOSC4",1.51929112032519,6.85601629283489,9.1804241098058,3.23515690855016e-15,5.80425210063411e-14,23.9973847852548

"LOC441081",1.52359926341462,7.0780853271028,7.57168651417008,1.28475491955456e-11,1.33116592262938e-10,15.8092833658256

"DDAH2",1.54036590569105,7.26285719626168,11.6864168152804,6.3061091865287e-21,2.90550626455913e-19,37.0287814395882

"TPST1",1.54238324878048,6.70157813084112,6.46495585784767,2.96636751227375e-09,2.15776582056728e-08,10.4568599336579

"SLC37A3",1.54519359024389,7.34356785046729,10.1501277196689,1.99240992876511e-17,4.97069550776594e-16,29.0387752631093

"CEP55",1.54578235609755,3.48075065420561,6.25057386039793,8.1979339622377e-09,5.56159962256446e-08,9.46052689008137

"CD59",1.54670418439024,6.93831254205607,12.4756819662622,1.04909560390687e-22,7.01177941438374e-21,41.0918630775377

"CAMP",1.54773937560974,7.64953962616822,5.98162774528477,2.87360916771122e-08,1.79176523255936e-07,8.23319311081807

"BCL6",1.56291420650405,10.4234485358255,15.6163329871723,1.54760900087458e-29,4.65464901582485e-27,56.6861981429097

"DLGAP5",1.56570215121951,3.92261869158878,6.78255268417119,6.41850113269209e-10,5.14216211721965e-09,11.9595296730229

"ADAM9",1.56622131219511,5.17495828660436,14.0316451813424,3.80122478684365e-26,5.27663607430124e-24,48.9496500867127

"GPR97",1.576134102439,8.66886827102804,13.6389990972663,2.74567842666662e-25,3.14590827140029e-23,46.9889767199147

"CCNA1",1.57728644390243,5.32791037383178,6.4065926701382,3.9176010556034e-09,2.79985646399642e-08,10.1841153708089

"MPO",1.58145886585365,5.53957813084112,5.18430109474594,1.01032726131456e-06,4.98034073384174e-06,4.76523661642577

"LRG1",1.59031406829267,9.02708056074766,12.6364561229279,4.5800438971492e-23,3.27329539910119e-21,41.9139773405675

"C1QB",1.59417989268292,4.92231635514019,7.64072891363228,9.06847783361003e-12,9.60283068395233e-11,16.152743616997

"KDM5D",1.59879510731706,5.24640644859813,4.56427128591621,1.32580204266672e-05,5.42419105118984e-05,2.27761335460732

"METTL9",1.60123408048779,8.3699736682243,15.3707602009094,5.08990294598693e-29,1.23844773365558e-26,55.5068335431554

"ACER3",1.60398189756096,5.95874063862928,11.4311729784385,2.39143286119329e-20,9.92078134274727e-19,35.7066975266322

"ZDHHC20",1.60439242682926,7.06874238317757,10.2152498645516,1.4145838572033e-17,3.62948026394993e-16,29.3782214305063

"APOBEC3B",1.60484817560974,5.58884644859813,3.53899404290792,0.000592325199232682,0.00177484463669347,-1.33917365298064

"ADM",1.60790291707316,9.65724906542056,14.2789719538175,1.10320566055616e-26,1.69432046662012e-24,50.1761768784248

"CLEC4E",1.61056517073169,8.22096789719626,10.3132866017622,8.44683281495776e-18,2.25266212571318e-16,29.8892763925831

"CSGALNACT2",1.61497345365853,8.20919560747664,10.1871833659937,1.63960012388951e-17,4.15755745700556e-16,29.2319221865593

"LOC101928429",1.6175823317073,7.11833093457944,12.2407557025676,3.53466123348129e-22,2.09134122980977e-20,39.8869686352592

"MKNK1",1.61908937886178,8.23266426791277,14.6018738574851,2.21622758013712e-27,3.96631473122887e-25,51.767213549028

"KCNE1",1.63316207560975,4.84016855140187,10.2955022175737,9.27505167174261e-18,2.45539418033724e-16,29.7965662365925

"SORT1",1.63449403739837,6.61796037383178,9.55906262436266,4.44976131341445e-16,9.0905265322632e-15,25.9615358750439

"ATP11B",1.64156084796747,6.47669699376947,11.8805734319804,2.29338931207467e-21,1.16580623363796e-19,38.0320680885933

"ATP6V1C1",1.64210250853657,6.86623859813084,10.1792560803011,1.70940605935466e-17,4.32443787562209e-16,29.1906014731068

"FAM132B",1.64310247317072,5.24578962616822,5.10685114814917,1.40758391183254e-06,6.75110290381696e-06,4.44366280740063

"PLSCR1",1.64567992845527,7.81420420560748,8.93336323308577,1.1753155506236e-14,1.94137744078978e-13,22.7207669679053

"FGD4",1.65696200650405,6.39848641744548,13.6107672085012,3.16707942238651e-25,3.57721200670041e-23,46.8473828683417

"MGAM",1.67091335121949,10.295316635514,10.8027427055091,6.44576434747888e-19,2.12455139946203e-17,32.4399935118762

"BST1",1.67877331707316,8.35604056074766,12.680071561935,3.65914674838241e-23,2.68606179105834e-21,42.1366416637798

"C5orf30",1.67882398048779,6.11601018691589,7.01919324240339,2.01619800635952e-10,1.73670516418916e-09,13.097914113609

"CARD6",1.6869741317073,8.20724289719626,15.9636724725639,2.90917392230664e-30,1.17324382322906e-27,58.3415361616782

"PLA2G4A",1.6955477707317,4.59328214953271,11.1670259427822,9.53218515193658e-20,3.65344193743693e-18,34.3353115810878

"ATP8B4",1.7016869609756,5.65512214953271,7.47820392300511,2.05611556650617e-11,2.07190240077669e-10,15.3457661938684

"PGS1",1.70674851707315,8.75520429906542,16.4683025244989,2.63425144500007e-31,1.72862772852959e-28,60.7194047595554

"BCAT1",1.71237668048779,5.43324577102804,8.92228771482169,1.24516414931386e-14,2.05049655158873e-13,22.6636502891264

"HTATSF1P2",1.72785595609755,4.61859009345794,7.35460932208293,3.81879701790562e-11,3.69673891027028e-10,14.7357612502481

"SLC22A4",1.73130324390242,8.91749514018692,16.0175025205818,2.24826002739744e-30,9.54628841044933e-28,58.5967254265469

"FAM20A",1.73415365975609,4.62957577102804,7.827539612294,3.51867098514963e-12,3.96651848950626e-11,17.086583138763

"MAPK14",1.73851722073169,8.58010880841121,16.1739781727689,1.06508550231269e-30,4.90732479842155e-28,59.3364449158499

"ARG1",1.74197221707316,4.56056196261682,13.7189245145572,1.83353669429698e-25,2.19365950911609e-23,47.3893908771988

"CR1",1.75090665463413,7.52261807476636,16.2890504851655,6.16048213637597e-31,3.17631525388623e-28,59.8784556171232

"MMRN1",1.75347802926829,3.69934570093458,6.64095062140615,1.27451284567636e-09,9.78361420528946e-09,11.2858161527548

"LILRA5",1.75780827195121,6.4373410046729,13.2245166729237,2.25145286862619e-24,2.14780669031278e-22,44.9022109695734

"ECHDC3",1.75799798536583,7.73197439252336,9.37820247228384,1.14877943596206e-15,2.21521092482266e-14,25.022363201683

"SLC2A3",1.76084711382112,9.43529090342679,16.1753225590355,1.05828465717361e-30,4.90732479842155e-28,59.3427869142736

"LINC00266-1",1.78452394634145,5.50145429906542,10.3262306330786,7.89092496755766e-18,2.11221236307121e-16,29.9567539531189

"ASPH",1.78777202439024,5.18567916926272,12.7169836359307,3.02640594170691e-23,2.25989036785045e-21,42.3249605792168

"CLEC1B",1.78805280487804,6.03048345794393,6.69623827173463,9.75680122074924e-10,7.60559864777987e-09,11.5481682342277

"LIN7A",1.79620236463414,6.20696871495327,13.082719952153,4.64219754044399e-24,4.1712360057392e-22,44.1845124580658

"AIM2",1.79693173170731,7.97590186915888,10.5540097617898,2.38197563876454e-18,7.0756766059597e-17,31.1440848721702

"AP5B1",1.80120585853658,6.7505861682243,11.8910999479492,2.17115008893668e-21,1.10887394282839e-19,38.0863986485562

"CEACAM1",1.80974720878048,7.15511091588785,7.53453002973008,1.54910264299409e-11,1.58834364271008e-10,15.6248358511632

"PTX3",1.81035148292682,4.42350336448598,6.81997050541309,5.34963756745602e-10,4.33102781782087e-09,12.1385161452944

"PRTN3",1.8510355414634,4.71274140186916,4.96958966833122,2.51624931178178e-06,1.15713270007718e-05,3.88107455946495

"PSTPIP2",1.85726374146339,7.96613121495327,12.0222029661035,1.09819888534756e-21,5.99030147662502e-20,38.7624753773677

"KLHL2",1.86012510731705,9.43543803738318,11.5138369347789,1.55243289936686e-20,6.67022508646614e-19,36.1352317845349

"TP53I3",1.86934773658536,6.39369504672897,10.9355399893673,3.20967374653423e-19,1.13017048749917e-17,33.1313631644862

"SLPI",1.88057825365852,7.93339841121495,8.70823732568564,3.79254849003212e-14,5.77956633016506e-13,21.5619649248403

"OPLAH",1.88133633658536,6.13726897196262,11.4395700089767,2.28870801572261e-20,9.53114847701406e-19,35.7502430422032

"CEACAM6",1.88398656829267,6.15484799065421,5.06945931309756,1.65034938953956e-06,7.79954527651311e-06,4.28946847636786

"DPY19L3",1.88619990731706,7.50801579439252,11.1592425067461,9.92904523434274e-20,3.77879568628633e-18,34.2948595477182

"FOLR3",1.89149159999998,8.39562271028037,7.15325840112371,1.03995241483744e-10,9.37949585310485e-10,13.7493193582296

"GPR160",1.90125749268291,8.87224925233645,10.5185102636525,2.87073811571154e-18,8.35562283544804e-17,30.9590608873107

"LRRN1",1.90412876585365,4.14699046728972,7.62923869936775,9.61031806737543e-12,1.01517774511715e-10,16.0955193672716

"ECRP",1.9221677707317,5.17683355140187,12.212042954755,4.10152543997426e-22,2.3791562849666e-20,39.7394264602195

"SERPINB1",1.92711790731706,9.28572801401869,15.2201792355365,1.06000080302687e-28,2.44194865846244e-26,54.7800205823974

"MAP2K6",1.9293696609756,5.84837775700935,14.7279872761419,1.18784345977759e-27,2.29667411798961e-25,52.3853746493924

"FCER1G",1.94207777073168,11.032841682243,17.9786513560325,2.40774033144757e-34,2.74419036197353e-31,67.6403803856524

"PFKFB2",1.94710109024389,5.92145672897196,10.6923078501391,1.15144735577766e-18,3.60847937617442e-17,31.8647497908049

"TXN",1.9501723609756,6.88824481308411,15.5451862444531,2.18340602506384e-29,6.06175095804584e-27,56.3452790179555

"MS4A3",1.95357888536584,6.47629542056075,4.97922658291268,2.41638883285077e-06,1.11499893832055e-05,3.92026155743194

"C3AR1",1.95392859512194,9.07955682242991,10.1883667570574,1.62942683937609e-17,4.14145988341422e-16,29.238090613593

"SLC51A",1.95834366829268,5.7255423364486,6.7050540132521,9.34913790701043e-10,7.30095857830187e-09,11.590084027971

"IL10RB-AS1",1.96945819268291,5.94530200934579,12.229319323223,3.75037009383108e-22,2.19498011843005e-20,39.8282091959884

"RP1-193H18.2",1.98552054634146,6.22626747663551,14.5500721344559,2.86481450745044e-27,5.04370391535279e-25,51.512769058304

"CYP1B1",2.01844724878048,7.60853322429907,9.06603820176866,5.88179384223035e-15,1.01811734697754e-13,23.4057536838745

"CD163",2.01892658211381,6.92847956386293,7.16265541834302,9.92643632245194e-11,8.97523918842158e-10,13.7951445498183

"RAB13",2.01954859024389,6.56984224299065,14.333302473834,8.4143807634875e-27,1.33002493017023e-24,50.4447035140236

"FCAR",2.04054988390243,6.59400325233645,16.3857195590733,3.89424382372055e-31,2.19993205790398e-28,60.3324816599924

"TNFAIP6",2.04307162682926,9.03011224299065,10.3226853333561,8.03944091984484e-18,2.14930979159556e-16,29.9382722066922

"GALNT14",2.05489689268291,6.90124719626168,12.8363129199754,1.63952196907797e-23,1.30050726155251e-21,42.9329681212242

"CST7",2.05613075609755,10.8802303738318,15.6600292280088,1.25311547024928e-29,3.93278485626783e-27,56.895270317618

"UPP1",2.06982996585364,8.79918523364486,16.895940590718,3.52771194359522e-32,2.82935563476128e-29,62.7090020105481

"FKBP5",2.07822910243901,9.28227968847352,10.831716208344,5.53591192128064e-19,1.84715212103748e-17,32.5908717801854

"ST3GAL4-AS1",2.08137593658535,6.83327074766355,13.2301247585866,2.18801628589853e-24,2.10125503387474e-22,44.930557257401

"CD24",2.09643028536584,7.57215225856698,7.39996422833273,3.04376616486671e-11,2.98788559837663e-10,14.959229002175

"EIF1AY",2.12436102195121,4.74210158878505,4.72182053414152,7.01778101370296e-06,3.0081165449671e-05,2.89027779834397

"NAIP",2.12549623414632,7.74718669781931,14.067810805853,3.17089777476484e-26,4.45881761769692e-24,49.1294179122478

"ORM1",2.13296537560974,6.86827457943925,7.87192619378811,2.80748427758514e-12,3.22013093385096e-11,17.3093883769925

"FGF13",2.13896835609755,5.43016579439252,6.892200796725,3.75985538909778e-10,3.13395953551034e-09,12.4851244254424

"RRM2",2.14179394878048,6.40018640186916,6.9742198947447,2.5152023423721e-10,2.13930505593354e-09,12.8804147876388

"GGH",2.14282283902438,6.29326289719626,10.7287501640833,9.50787731986141e-19,3.02339329459029e-17,32.054599843403

"FCGR1B",2.16288637073169,9.34602588785047,11.1022402037711,1.33867443845389e-19,4.98949999392754e-18,33.9985412987747

"IL18RAP",2.1677816926829,10.8215722429907,15.0342730167689,2.63179651090407e-28,5.75672257006339e-26,53.8789240034607

"SULT1B1",2.19817368780487,6.4289885046729,15.4057175820373,4.29452679399806e-29,1.05679520140941e-26,55.6751683291707

"UGCG",2.21388454146339,9.08883859813084,11.8210284536918,3.12676087734438e-21,1.54236917537341e-19,37.7246109144324

"RPS4Y1",2.22480719024388,7.57016962616822,4.18812221834759,5.73819031622726e-05,0.000210611036098138,0.8731052613889

"ST6GALNAC3",2.24871148292682,5.21120280373832,11.6830276724736,6.4185667513201e-21,2.95104167727891e-19,37.0112491267339

"HK3",2.25505353658534,9.70849186915888,15.3564288693098,5.45733917067068e-29,1.31309644156526e-26,55.4377789065859

"PFKFB3",2.26483879024388,8.84449644859813,12.4536944689978,1.17519725702486e-22,7.68848839905539e-21,40.9792711266199

"AZU1",2.28885387317072,5.79129813084112,5.61572480696043,1.51906363353955e-07,8.47380808456954e-07,6.60785767269967

"TLR5",2.29447365365851,8.52378887850467,20.4416031828125,4.943378764432e-39,1.52926953062536e-35,78.2906067020814

"SLC26A8",2.31432959999999,5.95917878504673,20.1521025309998,1.69033829802456e-38,4.57553448046522e-35,77.0792651319651

"ZDHHC19",2.33761104878048,6.45665724299065,10.1208314866763,2.32430954837391e-17,5.75233408800423e-16,28.8860820186343

"PGLYRP1",2.34347125365852,8.8168708411215,14.1709871497451,1.89181078603689e-26,2.80597003915266e-24,49.6414919687818

"NLRC4",2.34403475121949,7.32790028037383,14.6258859318026,1.96780852380083e-27,3.61126216804297e-25,51.8850535825343

"DHRS9",2.38993686666665,8.35779853582554,9.93571487432409,6.15239238177362e-17,1.43316333048953e-15,27.9215165303307

"CYSTM1",2.41754887804875,10.2099570093458,12.2484033291139,3.39739962739748e-22,2.0156353131861e-20,39.9262564321729

"BCL2A1",2.42205149756097,9.02629121495327,10.4627707146402,3.84838120016777e-18,1.09365741324978e-16,30.6685233618621

"DAAM2",2.43602656097559,6.38971747663551,8.08974846370015,9.22984791633881e-13,1.13822526553711e-11,18.4075252594445

"ELANE",2.43688945365852,6.65166915887851,6.22717736465926,9.15178918433854e-09,6.15472033499538e-08,9.35273116724242

"VSIG4",2.44210738048779,6.46310794392523,8.73901156408446,3.23216532991313e-14,4.96753301769118e-13,21.7200870164601

"RNASE3",2.48478059024389,7.01489317757009,10.5383484669836,2.5864014313095e-18,7.67240041027495e-17,31.0624594702893

"ALPL",2.49221955365853,7.80535406542056,13.3908695929493,9.65670087329201e-25,9.86395553826125e-23,45.7417663565131

"METTL7B",2.50917837560975,6.02630934579439,10.0523106456364,3.33267021010198e-17,8.09069208517469e-16,28.5289870830391

"CA4",2.51225266341461,7.98815654205607,13.8120442371864,1.14640701248839e-25,1.44333975903698e-23,47.8550725333374

"LOC100134822",2.54120741463414,7.46511336448598,15.7712766647315,7.32926378203556e-30,2.60188864262262e-27,57.4264827115155

"MS4A4A",2.56428046016258,6.90466364485981,10.379068415145,5.97644046976587e-18,1.63202797443607e-16,30.2321980477576

"PCOLCE2",2.57042570243902,4.36180214953271,8.65467602391818,5.00834204504058e-14,7.42846897159957e-13,21.2869899409244

"S100P",2.58091012682926,10.7715865420561,12.4697719212097,1.08159258137337e-22,7.20673456912011e-21,41.061603060847

"DACH1",2.61738179837397,5.42384626168224,14.2333530319068,1.3852796070543e-26,2.11255140075781e-24,49.9504530519901

"CLEC5A",2.62152796585364,5.5806638317757,8.7626004357819,2.85919500048324e-14,4.41940526305957e-13,21.8413533022126

"GRB10",2.63634227804877,6.56346205607477,16.1917053023738,9.78834065808328e-31,4.71036704335097e-28,59.4200523415587

"HPGD",2.69808108170731,6.33913920560748,8.1009556206994,8.71478070466857e-13,1.07962572173683e-11,18.4642281372607

"RGL4",2.73689952682926,8.16496542056075,11.896198674679,2.11431055929362e-21,1.08753907747039e-19,38.1127122694555

"BMX",2.7420645609756,6.42545327102804,13.8402876860005,9.94390517193758e-26,1.2894327335228e-23,47.9961357702144

"DEFA4",2.80986482926827,8.36287635514019,5.69811998878284,1.04869854126064e-07,6.00147117098288e-07,6.96911013771069

"GYG1",2.84794149268291,10.4926014018692,17.1953907075482,8.75018510028722e-33,7.57941033386879e-30,64.0881466591905

"ANKRD22",2.89420909268292,7.13444621495327,10.3599578280367,6.60833106187916e-18,1.79552583619816e-16,30.1325746485796

"TCN1",2.89632859512194,8.15535990654206,9.12132587474006,4.40627615848845e-15,7.75755367577783e-14,23.6915982097639

"MMP9",2.90325151707315,11.1203925233645,15.0485775477992,2.45358171308048e-28,5.47755793781008e-26,53.9484059504408

"IRAK3",2.95582363739836,7.5908585046729,22.8124951198878,3.06002041320687e-43,2.20882473493316e-39,87.8185501986518

"IL18R1",2.98591633658534,7.78842953271028,12.5663147586117,6.57344149119692e-23,4.56243190679068e-21,41.555566638185

"SAMSN1",3.04058190081299,8.02193663551402,15.5155229052859,2.52076924297908e-29,6.73916764897678e-27,56.2029545303532

"GADD45A",3.06659022439023,8.53848598130841,15.7339337072574,8.77357070037318e-30,2.96861989869658e-27,57.2483397760423

"LTF",3.10389341951219,10.0506274766355,8.029765833024,1.25469116990754e-12,1.51620185738548e-11,18.1043689811612

"RNASE2",3.12422536097559,9.93520336448598,18.9780431958355,2.75209418020548e-36,4.96638328936248e-33,72.056617139447

"CRISP3",3.14727671219511,6.04299448598131,8.30019631118499,3.13106801544773e-13,4.11927569104013e-12,19.4753406403783

"S100A12",3.24331692682924,12.3120485981308,21.3549703223697,1.09264868280156e-40,4.73226144521355e-37,82.0431213829309

"CEACAM8",3.48991883414633,7.23067803738318,7.67380787031422,7.67220128219878e-12,8.18431126926181e-11,16.3176272521467

"CLEC4D",3.52610211463414,8.07952528037383,14.4133465624447,5.64900207179638e-27,9.40993383575004e-25,50.8397155011397

"LCN2",3.56518281951218,9.15621457943925,8.81636756047194,2.16167625387339e-14,3.41686856041083e-13,22.1179612333265

"IL1R2",3.62395204878047,11.0347402803738,15.4559608211362,3.36484039396447e-29,8.37535847486213e-27,55.9168492049647

"TDRD9",3.63568440975608,6.11658663551402,15.4631546932394,3.24941441988901e-29,8.37535847486213e-27,55.9514279673028

"VNN1",3.69445639512194,8.28975644859813,15.8338735103282,5.42353738304419e-30,2.09726253624682e-27,57.7247080159116

"GPR84",3.77117016585365,7.2565208411215,11.9226777663296,1.84224856758972e-21,9.63620597370905e-20,38.2493401160718

"RETN",3.83194221951218,7.55049261682243,11.191622018607,8.37951522909336e-20,3.26364032888519e-18,34.4631274455519

"ANXA3",4.14905397560974,9.7700808411215,20.8895213840991,7.52769319524121e-40,2.71686993571581e-36,80.1439130350065

"OLAH",4.16870527967479,6.36849258566978,11.6305185091483,8.44130948322288e-21,3.80034421744681e-19,36.7395346268734

"HP",4.18131650243901,8.16573579439252,14.1423680967942,2.18295763649475e-26,3.15146317455291e-24,49.4995695796806

"MCEMP1",4.21310222439023,9.80324934579439,24.2426574240787,1.21076140435489e-45,2.62190382113051e-41,93.2410694403262

"OLFM4",4.74924144878047,7.02892719626168,8.42656444545143,1.63161107619435e-13,2.23907083998661e-12,20.1194336778337

"CD177",5.62491373658535,9.44082635514019,16.303017509486,5.76505867026492e-31,3.12105863761467e-28,59.9441281865486

"MMP8",5.92518899756096,7.95249864485981,12.6780106062515,3.69815372908912e-23,2.70552429065624e-21,42.1261236776772
