# Supplementary material for: Identification of a novel four-gene diagnostic signature for patients with sepsis by integrating weighted gene co-expression network analysis and support vector machine algorithm
Source: Hereditas. 2022 Feb 21;159:14. doi: 10.1186/s41065-021-00215-8 (PMC8859894; doi:10.1186/s41065-021-00215-8)
Supplement: Supplementary file 2 — Additional file 2. [file 41065_2021_215_MOESM2_ESM.docx]

Genes GS.Normal GS.Sepsis GS.ImmuneScore GS.ESTIMATEScore GS.StromalScore p.GS.Normal p.GS.Sepsis p.GS.ImmuneScore p.GS.ESTIMATEScore p.GS.StromalScore Module

A1BG -0.194711943797408 0.194711943797408 -0.174147560001058 -0.0880044927628956 0.101474363629924 0.0444589290921125 0.0444589290921125 0.0728175291908633 0.367384285617205 0.298331736669691 grey

A1BG-AS1 0.0314792149484116 -0.0314792149484116 -0.255900629760339 -0.269009451958349 -0.0878239666316255 0.747544587037731 0.747544587037731 0.00780438474145486 0.00508159938316274 0.368371708383164 green

A1CF 0.0581442448947243 -0.0581442448947243 -0.166640714068536 -0.184685135012125 -0.0733172472499591 0.551918312624163 0.551918312624163 0.0862507468465809 0.0568612867198725 0.452952072007522 turquoise

A2M 0.0214845525173383 -0.0214845525173383 -0.174246695494946 -0.18894218660212 -0.0695864997555901 0.82614142314036 0.82614142314036 0.0726521327536084 0.0512869669441589 0.476329888732362 turquoise

A2M-AS1 0.668471733676905 -0.668471733676905 0.715589879998664 0.403270494410524 -0.346322538756987 3.62583708028113e-15 3.62583708028113e-15 4.68471227327935e-18 1.65610486595902e-05 0.000258117940084914 brown

A2ML1 -0.335152382316038 0.335152382316038 -0.378074171317607 -0.264848569896615 0.0951416269543773 0.000417518547855641 0.000417518547855641 5.94518105384193e-05 0.00583614183619137 0.329656137528839 turquoise

A2MP1 -0.0899506564929616 0.0899506564929616 -0.100715040327381 -0.00999272265098675 0.128062855399392 0.356842822625499 0.356842822625499 0.301980670948306 0.918634450882873 0.188660124741627 turquoise

A4GALT 0.223295635577765 -0.223295635577765 -0.107091455874343 -0.157400028224232 -0.112778483339618 0.0207814968146761 0.0207814968146761 0.272242810514917 0.10541611743995 0.247443309514119 green

A4GNT -0.126722293983313 0.126722293983313 -0.236705063418514 -0.170349751108699 0.0518776751065846 0.193368103601275 0.193368103601275 0.0140991038465495 0.0793858296518881 0.595639548189256 turquoise

AA06 -0.176345393737038 0.176345393737038 -0.351555802916001 -0.208248843269646 0.152960485899409 0.0692212571513099 0.0692212571513099 0.000204766491401975 0.0313580544956323 0.115736751739956 turquoise

AAAS 0.525399857080527 -0.525399857080527 0.0779234026326935 -0.170457422833865 -0.401314104685013 6.23172110426294e-09 6.23172110426294e-09 0.424990854254896 0.0791932849117936 1.83577447973792e-05 brown

AACS 0.306027227719135 -0.306027227719135 0.23201961704944 0.00722091824669283 -0.321819447088159 0.00134751005740619 0.00134751005740619 0.0161833974697656 0.941155684278099 0.000724280321733708 red

AACSP1 0.00366500717674554 -0.00366500717674554 -0.0563647596409991 -0.121802865643441 -0.12543842922968 0.970113477314683 0.970113477314683 0.56417442290143 0.211372880194784 0.197956146114144 grey

AADAC -0.143703776724105 0.143703776724105 -0.170355722433223 -0.045673303008307 0.167814079181934 0.1397560640933 0.1397560640933 0.0793751415704564 0.640397514937742 0.0840297044957595 turquoise

AADACL2 -0.0637310295873293 0.0637310295873293 -0.0295833283000629 -0.0420542826298244 -0.0287349029123861 0.514298450021182 0.514298450021182 0.762282922952385 0.667127415737222 0.76890643161575 turquoise

AADACP1 -0.165295790032762 0.165295790032762 -0.12765718681857 -0.0350403469522787 0.124370675042822 0.0888537473712697 0.0888537473712697 0.19007593239208 0.720109123019994 0.201831263538833 turquoise

AADAT -0.0746045224945875 0.0746045224945875 -0.16593161971 -0.109264850246829 0.0535845120107135 0.445036410938115 0.445036410938115 0.0876154755477098 0.262574355742182 0.583577764617189 turquoise

AAED1 0.0401894473875836 -0.0401894473875836 0.149966740897514 0.153678691221768 0.0447338071701398 0.681068045831777 0.681068045831777 0.123126921697753 0.114016018618116 0.64729427835915 turquoise

AAGAB -0.216802343911716 0.216802343911716 0.163143185286026 0.242489043800582 0.176396127074747 0.0248949251004861 0.0248949251004861 0.0931493774278665 0.0118512351446654 0.0691399727950845 green

AAK1 0.642021460148275 -0.642021460148275 0.240298490099077 -0.0783232239384033 -0.47883367743879 9.1628800328997e-14 9.1628800328997e-14 0.012662741191115 0.422611594304039 1.8172909225387e-07 blue

AAMDC -0.144418001563082 0.144418001563082 -0.451144236820607 -0.373716508415656 0.0156951992880432 0.137777840935394 0.137777840935394 1.07819233986598e-06 7.33931117039109e-05 0.872522381775252 greenyellow

AAMP 0.692488633487622 -0.692488633487622 0.523954355641285 0.258201490892996 -0.316457519747514 1.43189752271465e-16 1.43189752271465e-16 6.97354443204939e-09 0.00724909691495606 0.00089763184228686 blue

AANAT -0.0158554292326431 0.0158554292326431 -0.24767609125033 -0.237367438953983 -0.0459968255450905 0.871232120748693 0.871232120748693 0.0101084711837087 0.013824152830533 0.638029591688441 pink

AAR2 0.687447134900437 -0.687447134900437 0.60086526428689 0.307090363968971 -0.344273607981878 2.89547396337663e-16 2.89547396337663e-16 7.79869785517779e-12 0.00129371409966875 0.000282302640787898 blue

AARS 0.373727407247036 -0.373727407247036 0.180398641282242 -0.0502856467580808 -0.345033103619026 7.33547249898993e-05 7.33547249898993e-05 0.0629667117091754 0.606989940858304 0.000273103770531284 red

AARS2 0.695741412850383 -0.695741412850383 0.445378036895572 0.117217885910488 -0.442448357140389 9.02127139237813e-17 9.02127139237813e-17 1.53231550183482e-06 0.229200910302982 1.82743904800062e-06 brown

AASDH 0.190834420768166 -0.190834420768166 0.107109222814439 -0.0604622909480137 -0.256770283091501 0.0489581982500166 0.0489581982500166 0.272162812376135 0.536148788976607 0.00759023639715128 yellow

AASDHPPT 0.266859178879235 -0.266859178879235 0.288085000356574 0.102108989102212 -0.241600967307744 0.00545992699235507 0.00545992699235507 0.00262076183172665 0.29530439665872 0.0121745815513847 yellow

AASS 0.311842840017571 -0.311842840017571 0.182347193500829 0.0332867545849688 -0.206088886626776 0.0010763207175362 0.0010763207175362 0.0601281640934919 0.733577163147123 0.033198135747017 brown

AATF 0.448259000522395 -0.448259000522395 0.324664012878992 0.180636454280705 -0.161075334290828 1.28655442245802e-06 1.28655442245802e-06 0.000645313363861972 0.062614535536421 0.0974289778227432 blue

AATK -0.136889261724744 0.136889261724744 -0.0688699917959992 -0.0422527315569897 0.027494409726656 0.159727085426309 0.159727085426309 0.480893120913991 0.665650447385963 0.778620618799192 turquoise

AB488780 0.539948041027911 -0.539948041027911 0.253655943223925 0.012382624294122 -0.344216989640979 1.9499466425603e-09 1.9499466425603e-09 0.0083819755387575 0.899267083405791 0.000282999724250313 brown

ABAT -0.0732971734549558 0.0732971734549558 0.236078984003992 0.291566517619433 0.154623398251161 0.453076126204966 0.453076126204966 0.0143633486690298 0.00231093786894287 0.111782925482229 purple

ABCA1 -0.469443238660112 0.469443238660112 -0.0428323952308891 0.242108542844142 0.472319051994858 3.38318229230035e-07 3.38318229230035e-07 0.661343597418751 0.0119888439235107 2.80235306255158e-07 purple

ABCA11P 0.473462087273259 -0.473462087273259 0.352208283307389 0.106114018941276 -0.327134091715948 2.59896815602298e-07 2.59896815602298e-07 0.00019888331132936 0.276668308480745 0.000583230111010423 blue

ABCA12 0.041489924124821 -0.041489924124821 -0.114893340871954 -0.144663013016017 -0.0799415122431471 0.671334626534956 0.671334626534956 0.238631690273948 0.137104144862296 0.413060369837264 grey

ABCA13 -0.398889405100736 0.398889405100736 -0.364189665111826 -0.290362593891103 0.0318752045490871 2.08386607076317e-05 2.08386607076317e-05 0.000115132913900294 0.00241412152301933 0.744477474500678 magenta

ABCA17P 0.216717743687672 -0.216717743687672 -0.167240480071941 -0.317090751003948 -0.297031082974456 0.0249528027168784 0.0249528027168784 0.0851096909856439 0.000875349324772215 0.00189085780952981 green

ABCA2 -0.332785649402225 0.332785649402225 0.0270261696412814 0.131006649103461 0.183291631038805 0.000461246205321045 0.000461246205321045 0.782296334424396 0.178615164407837 0.0587904688327835 turquoise

ABCA3 0.205436895233831 -0.205436895233831 -0.0442421027171903 -0.0935722975620278 -0.0950101790639471 0.0337711533645823 0.0337711533645823 0.650915824163567 0.337732045445708 0.330327810158925 green

ABCA4 -0.0714647821869014 0.0714647821869014 -0.312413607620162 -0.230985791493521 0.0580378280693278 0.464479450225554 0.464479450225554 0.0010525861838656 0.0166776627813638 0.552647614249097 turquoise

ABCA5 0.198728359028624 -0.198728359028624 0.254548988148435 0.0306922797647441 -0.314447265772413 0.0401665486451956 0.0401665486451956 0.00814781622346861 0.753651408278621 0.000971847304581344 yellow

ABCA6 0.121878719579494 -0.121878719579494 -0.0941387317681381 -0.128358438649169 -0.0821697168679655 0.211086488840161 0.211086488840161 0.334802792740537 0.187633359900124 0.40011776073016 green

ABCA7 0.0558908911609587 -0.0558908911609587 0.298023121737412 0.331391466592828 0.132983191618186 0.567459806930931 0.567459806930931 0.00182247189777129 0.000488937570042923 0.172094376607739 black

ABCA8 -0.320322376885652 0.320322376885652 -0.346108722784202 -0.254841648471873 0.0660901279365915 0.000769304457918283 0.000769304457918283 0.000260549158155125 0.00807234522503213 0.498817255862351 turquoise

ABCA9 -0.150080858409123 0.150080858409123 -0.210714004557043 -0.143477797955263 0.0600334966392652 0.122838715650427 0.122838715650427 0.0293640775494639 0.1403864338014 0.539048915087686 turquoise

ABCB1 0.826098579104735 -0.826098579104735 0.694437042860167 0.348276253757448 -0.409143695936125 6.56668703344365e-28 6.56668703344365e-28 1.08653907858384e-16 0.000236854204998574 1.21088469227036e-05 blue

ABCB10 -0.183435048375764 0.183435048375764 -0.358688208628452 -0.307288834606781 -0.00475501341672919 0.058589479931611 0.058589479931611 0.000148378089913854 0.00128389037162019 0.961231062724257 yellow

ABCB11 -0.183278319376742 0.183278319376742 -0.35076838466129 -0.335679881042347 -0.0643130964872866 0.0588091527349522 0.0588091527349522 0.000212080785668968 0.000408307706602383 0.510455985728192 turquoise

ABCB4 0.652786923850101 -0.652786923850101 0.415707264998116 0.0881129173495847 -0.449093574465835 2.55806905928959e-14 2.55806905928959e-14 8.47378257433667e-06 0.366792018288555 1.22265845189648e-06 blue

ABCB5 -0.310041333606443 0.310041333606443 -0.376855212675853 -0.224805167996319 0.161305452700761 0.00115446956697347 0.00115446956697347 6.30795033791522e-05 0.0199136313177944 0.0969452022159702 yellow

ABCB6 0.0583361766841268 -0.0583361766841268 -0.29230645691881 -0.307428691218867 -0.100570153935793 0.550604134264778 0.550604134264778 0.00224951286456477 0.001277008681916 0.302680235461616 green

ABCB7 0.423094252752288 -0.423094252752288 0.37442592418113 0.216453666634624 -0.171972955698896 5.61916406162731e-06 5.61916406162731e-06 7.09331526263034e-05 0.0251342052094013 0.0765226361915944 turquoise

ABCB8 -0.0824142877269012 0.0824142877269012 -0.266889262226659 -0.211288693620661 0.0258997084539613 0.398711949198506 0.398711949198506 0.00545446571352584 0.0289150099486815 0.791158635505941 turquoise

ABCB9 0.170051190737601 -0.170051190737601 -0.101454087158184 -0.235032015387886 -0.252569357198888 0.0799216954093693 0.0799216954093693 0.298428796647963 0.0148148596217693 0.00867486748973124 green

ABCC1 0.583559336507904 -0.583559336507904 0.546312999609366 0.33305865957854 -0.221682409345762 4.20755517043684e-11 4.20755517043684e-11 1.1524354816368e-09 0.000455995489827914 0.0217447042497893 blue

ABCC10 0.233447368935363 -0.233447368935363 0.0323645419705911 -0.0499416627262507 -0.13130684666725 0.0155216875774581 0.0155216875774581 0.740692857428865 0.609454834894514 0.177613284935676 turquoise

ABCC11 -0.310198906279357 0.310198906279357 -0.541213834499731 -0.407621760783651 0.0878716664172961 0.0011474333574949 0.0011474333574949 1.75783570245192e-09 1.31397443077844e-05 0.368110647014217 blue

ABCC12 -0.283281507273917 0.283281507273917 -0.208083603703751 -0.0137358494830542 0.276305667303826 0.00310964984777451 0.00310964984777451 0.0314957093894404 0.888326192762414 0.00396586612990179 turquoise

ABCC13 -0.17521226489383 0.17521226489383 -0.517008845171446 -0.432700428156166 0.0104840450439734 0.0710569907380467 0.0710569907380467 1.18848777749398e-08 3.24561846078165e-06 0.914648589782959 pink

ABCC2 -0.629255158413847 0.629255158413847 -0.287542222659147 -0.0130598278819826 0.391858579232985 3.90271682830725e-13 3.90271682830725e-13 0.00267230030798288 0.893789370063422 2.99289469995965e-05 brown

ABCC3 -0.151760581647287 0.151760581647287 -0.350955597988157 -0.225616089142144 0.122639181087137 0.118656381083845 0.118656381083845 0.000210320182307763 0.0194604237941591 0.208230594666699 salmon

ABCC4 -0.245200991949255 0.245200991949255 -0.565595293618076 -0.517034193498269 -0.062601273345251 0.0109098211846583 0.0109098211846583 2.18623755564592e-10 1.18620320232568e-08 0.521798520508981 pink

ABCC5 0.0537215080057079 -0.0537215080057079 0.0987066048887766 -0.11375500550124 -0.335063456302384 0.582614531450419 0.582614531450419 0.311772772184118 0.243347089050932 0.000419090023378806 black

ABCC6P1 -0.24212671605422 0.24212671605422 -0.273714584306556 -0.101404517783426 0.222105004996134 0.0119822399446979 0.0119822399446979 0.00433410314060804 0.298666164210757 0.0214887552012987 turquoise

ABCC8 0.00943319717734534 -0.00943319717734534 -0.113023552943241 -0.133679399746739 -0.0640039976904691 0.923176060684527 0.923176060684527 0.246410871154575 0.169839850190939 0.512494630777463 turquoise

ABCC9 -0.101297661095512 0.101297661095512 -0.185153554700365 -0.119468664493808 0.063953652479342 0.299178279866492 0.299178279866492 0.05622457950847 0.220321536039461 0.512827075747164 turquoise

ABCD1 -0.274557811640012 0.274557811640012 -0.26912090538135 -0.106348132451198 0.207105906132689 0.00421104457457095 0.00421104457457095 0.00506264249124848 0.275603947169405 0.0323206879350197 blue

ABCD2 0.588837767895722 -0.588837767895722 0.610173969855244 0.329075461961407 -0.320386902078359 2.54298365704304e-11 2.54298365704304e-11 3.01857229608789e-12 0.000538335671687924 0.000767312238616562 blue

ABCD3 -0.0781249432583573 0.0781249432583573 -0.0984632008373745 -0.108514954725912 -0.042286143274788 0.423790561977998 0.423790561977998 0.312973346017239 0.265883540771225 0.665401904117812 yellow

ABCD4 0.309938431982893 -0.309938431982893 0.130495846119117 0.0148407541676815 -0.162718999073852 0.00115908569918724 0.00115908569918724 0.180329432733947 0.879408415216289 0.0940149236608596 grey

ABCE1 0.430324778289496 -0.430324778289496 0.458807391510149 0.197273699424895 -0.325999051554679 3.72330579703011e-06 3.72330579703011e-06 6.69038700598765e-07 0.0416793137264174 0.000611044095094061 turquoise

ABCF1 0.719544510318813 -0.719544510318813 0.560790681444745 0.286920146076092 -0.320784714499579 2.52122862459772e-18 2.52122862459772e-18 3.34131446111993e-10 0.00273248983277463 0.000755133666603595 blue

ABCF2 0.580946956137009 -0.580946956137009 0.423490497716561 0.188545555255978 -0.289953040478668 5.38044096436044e-11 5.38044096436044e-11 5.49518810084825e-06 0.0517865090079135 0.00245015889731567 blue

ABCF3 0.534675208086177 -0.534675208086177 0.176358719683672 -0.0917408047343465 -0.409529737748518 2.98999662732581e-09 2.98999662732581e-09 0.0691998989888327 0.347314070221731 1.18597257260166e-05 blue

ABCG1 0.216316581091433 -0.216316581091433 0.521236110276803 0.501411050133788 0.0999717185034618 0.0252288162380067 0.0252288162380067 8.60380392628893e-09 3.77121382383096e-08 0.305580947623741 black

ABCG2 -0.251154838417137 0.251154838417137 -0.476727072048107 -0.303540649004955 0.17155743226162 0.00906964036014983 0.00906964036014983 2.09255029367379e-07 0.00148136337602659 0.0772475586679551 pink

ABCG4 0.143477817226261 -0.143477817226261 -0.104952397633283 -0.0798834945576049 0.0156199217181348 0.140386379953043 0.140386379953043 0.281990252872837 0.413400599803518 0.873128674461219 green

ABCG5 -0.334035796014921 0.334035796014921 -0.562615869986788 -0.484748316821135 -0.0121299993388819 0.000437649765250053 0.000437649765250053 2.8463101428033e-10 1.21685606379732e-07 0.901311723109527 blue

ABCG8 0.0766969529722381 -0.0766969529722381 -0.117023174873512 -0.144828415186132 -0.0771554397061733 0.432337154914709 0.432337154914709 0.229980679416762 0.136650764551271 0.42958242368502 turquoise

ABHD1 -0.00401574692496018 0.00401574692496018 -0.299446050564006 -0.286180976101027 -0.0542514353750665 0.9672548628998 0.9672548628998 0.00172829024812261 0.00280559397359841 0.57889546081408 green

ABHD10 0.451715574297221 -0.451715574297221 0.528281449388511 0.420227616225199 -0.0478701373639542 1.04092190585081e-06 1.04092190585081e-06 4.97221191957907e-09 6.59788127551325e-06 0.624390538565828 turquoise

ABHD11 -0.352602341720508 0.352602341720508 -0.436882597291727 -0.260096352238984 0.187875723639377 0.00019540644342971 0.00019540644342971 2.54225805360248e-06 0.00681833638517718 0.0526392427851612 blue

ABHD12 0.546436010830686 -0.546436010830686 0.34863228917359 0.16086778622301 -0.229115666137011 1.14065746653096e-09 1.14065746653096e-09 0.0002331582614522 0.0978669334150365 0.0176050002749772 blue

ABHD13 -0.380975547409902 0.380975547409902 -0.237245120756625 -0.104708494549149 0.163991447954793 5.15854363303514e-05 5.15854363303514e-05 0.0138745727767658 0.283116325041596 0.0914374211398656 yellow

ABHD14B 0.794707075981357 -0.794707075981357 0.6855466610549 0.34116777337974 -0.408400037790654 1.66749258194769e-24 1.66749258194769e-24 3.76184902821483e-16 0.000322976747510265 1.26026793378705e-05 blue

ABHD15 0.832856115678079 -0.832856115678079 0.64797079412342 0.284052697682863 -0.451172160114863 9.87317689997009e-29 9.87317689997009e-29 4.55560189597672e-14 0.00302601483689383 1.0763417620554e-06 blue

ABHD16A -0.109060769977411 0.109060769977411 -0.377306811146233 -0.250545009414352 0.118297512963401 0.263472144411393 0.263472144411393 6.17120904234567e-05 0.00924465558000011 0.224910922602426 grey

ABHD16B -0.247582028826135 0.247582028826135 -0.333793873161031 -0.198566552079627 0.143808901856377 0.0101379539297167 0.0101379539297167 0.000442127506683289 0.0403325169108971 0.139463549348209 turquoise

ABHD17A 0.025122435907417 -0.025122435907417 0.012200988187676 -0.0668521210546439 -0.130957267786391 0.79728936278823 0.79728936278823 0.900737103561994 0.493869488440861 0.178780365748001 greenyellow

ABHD17B -0.0963813304691378 0.0963813304691378 -0.0634740056508589 -0.0389571141445408 0.0253149098981228 0.323364429704716 0.323364429704716 0.515999876874689 0.690340028328883 0.79577006226835 green

ABHD17C -0.127681330036001 0.127681330036001 -0.248617065072509 -0.260438263623463 -0.0837731720069327 0.189991455844248 0.189991455844248 0.00981765080235855 0.00674307085698873 0.390954519925708 magenta

ABHD2 -0.266167713324115 0.266167713324115 0.00809690588810227 0.0899446846039834 0.140899940069167 0.00558680277160298 0.00558680277160298 0.934032318664315 0.35687487949411 0.147730689906063 brown

ABHD3 0.037761260022591 -0.037761260022591 0.417570459920346 0.41968967303717 0.110621624034365 0.699381564065465 0.699381564065465 7.64670836450677e-06 6.79859881738148e-06 0.256658598332239 tan

ABHD4 -0.00439730246282106 0.00439730246282106 -0.0120899284757426 0.020611111250709 0.0523665213394677 0.964145564430188 0.964145564430188 0.901636099413049 0.83310483979793 0.59217355665054 yellow

ABHD5 -0.517543809096044 0.517543809096044 -0.270415453854651 -0.130591620088313 0.167849692462749 1.14115519418114e-08 1.14115519418114e-08 0.00484702708309027 0.18000709859183 0.083963013104636 purple

ABHD6 0.51388142099345 -0.51388142099345 0.435091576867312 0.330540073068757 -0.0658151643988931 1.50511848371844e-08 1.50511848371844e-08 2.82371195652736e-06 0.00050659113579545 0.500609001073901 blue

ABHD8 -0.35389295280847 0.35389295280847 -0.314619927913002 -0.129828312479673 0.232790938272228 0.00018440926022426 0.00018440926022426 0.000965259686377765 0.182587809923722 0.0158229463898803 brown

ABI1 -0.392016456571113 0.392016456571113 -0.267999581343284 -0.121727911894729 0.179405243065723 2.968930065879e-05 2.968930065879e-05 0.00525626977522863 0.2116561447066 0.0644553223094342 brown

ABI2 0.73055092202497 -0.73055092202497 0.53949023261039 0.162174297565241 -0.501701231490426 4.24511907831736e-19 4.24511907831736e-19 2.02426990378559e-09 0.0951356873438411 3.69299612063456e-08 brown

ABI3 0.567310484130052 -0.567310484130052 0.536807919259306 0.439307352845994 -0.0277849872666043 1.87592284062263e-10 1.87592284062263e-10 2.51747878850832e-09 2.20327319009003e-06 0.77634201214405 brown

ABI3BP -0.22683328795654 0.22683328795654 -0.320152794129208 -0.23086759766457 0.0693813320033737 0.0187968354107398 0.0187968354107398 0.000774562921677319 0.0167349940937086 0.47763414839044 blue

ABL1 0.624174873996264 -0.624174873996264 0.324567761997672 0.0918928229206902 -0.311457633998457 6.82080423586681e-13 6.82080423586681e-13 0.000647850868320528 0.346512313050929 0.00109261360494772 brown

ABL2 0.0740150750852502 -0.0740150750852502 -0.0209288051058668 0.0103798088122513 0.0477392545050246 0.448651328946814 0.448651328946814 0.830570535973755 0.91549403455343 0.625339419663979 grey

ABLIM1 0.87067017789213 -0.87067017789213 0.707963621664518 0.29169838796169 -0.524583074694629 3.91928270781716e-34 3.91928270781716e-34 1.50285381148165e-17 0.00229988118828049 6.64104379604571e-09 blue

ABLIM2 -0.0365084198837342 0.0365084198837342 -0.156063598750196 -0.0732163721672364 0.100519289902695 0.708899213049992 0.708899213049992 0.108444211894582 0.453575661853432 0.302926077351899 yellow

ABLIM3 -0.298585348034083 0.298585348034083 -0.344089071677522 -0.18628981122257 0.179455132811462 0.00178471711003096 0.00178471711003096 0.000284580503006687 0.054704386831587 0.0643798856556852 salmon

ABO 0.2186731213427 -0.2186731213427 -0.0781963842727413 -0.189181421240525 -0.208287702636086 0.023644041528677 0.023644041528677 0.4233655586565 0.0509875870751369 0.0313257562020649 green

ABR 0.393883094532932 -0.393883094532932 0.56398862535351 0.476090410150099 -0.00453144358478991 2.69888331944328e-05 2.69888331944328e-05 2.52132650527811e-10 2.18326669572635e-07 0.963052572848329 black

ABRA -0.12909531564003 0.12909531564003 -0.104945826387278 0.0171117514319476 0.180127139809162 0.185091390217265 0.185091390217265 0.282020552248189 0.861127478187705 0.0633707482694585 turquoise

ABRACL -0.267967202514533 0.267967202514533 -0.0575948427513463 0.0832817693571111 0.224183191397996 0.00526195750002472 0.00526195750002472 0.555688502928703 0.393749289682443 0.0202673679745726 yellow

ABT1 0.577142625635403 -0.577142625635403 0.546809549826324 0.399376952013422 -0.109912748185731 7.66743969129294e-11 7.66743969129294e-11 1.10559655115731e-09 2.03158475054464e-05 0.259737936532557 blue

ABTB1 -0.276195020504305 0.276195020504305 0.0915346978271504 0.226404976150222 0.252218982667594 0.00398099958938773 0.00398099958938773 0.348402952190891 0.0190280818261591 0.00877121512695776 purple

ABTB2 -0.132001846602067 0.132001846602067 -0.316116662669548 -0.140537261823008 0.216782202055874 0.17530963042576 0.17530963042576 0.000909839494966379 0.14878675347813 0.0249086943611362 turquoise

AC000111.3 -0.077141763980556 0.077141763980556 -0.105273172153137 -0.0497199163295116 0.0672431614678467 0.429664445994838 0.429664445994838 0.280513838449808 0.61104612928427 0.491340476070937 green

AC002059.10 0.00699902189755312 -0.00699902189755312 -0.00978397928143293 0.0351878909990081 0.0737705218586312 0.942960862485643 0.942960862485643 0.920328500952475 0.718979774936545 0.450155914523081 yellow

AC002064.5 0.0246830512072342 -0.0246830512072342 0.0351047959813816 0.0336470465645015 0.0065251379830713 0.800760491195964 0.800760491195964 0.719615735865339 0.730803267117856 0.946816973338819 turquoise

AC003973.4 -0.293485513515813 0.293485513515813 -0.23114966251379 -0.0356158667905779 0.272405302637507 0.00215467665152346 0.00215467665152346 0.0165984564460763 0.715707342736472 0.00453155093719919 turquoise

AC003989.4 -0.0628170927590305 0.0628170927590305 -0.299862329121608 -0.29673661265641 -0.0715558483885601 0.520361496197543 0.520361496197543 0.0017015838244564 0.00191159948381384 0.463909036402668 green

AC004692.5 0.00217503070666716 -0.00217503070666716 0.207685934031516 0.165895240822726 -0.0176507053784765 0.982260931276289 0.982260931276289 0.0318290886053187 0.087685951008744 0.856799412682572 yellow

AC004941.5 -0.192047561433845 0.192047561433845 0.0935373334168641 0.228629895510916 0.253109276042239 0.0475117288865716 0.0475117288865716 0.337913388921392 0.017853045803242 0.00852822294627542 brown

AC005162.4 -0.134337034387428 0.134337034387428 -0.10007691662908 -0.015375276101659 0.118014536457404 0.167730349553138 0.167730349553138 0.305069723515992 0.875099578910788 0.22602984609977 turquoise

AC005224.2 0.338845688416464 -0.338845688416464 0.396592491080745 0.191058106630936 -0.246963083957179 0.000356846458534702 0.000356846458534702 2.34760345967081e-05 0.0486887840334635 0.0103338435494099 blue

AC005256.1 0.152557610638808 -0.152557610638808 -0.128538275927987 -0.151276323068352 -0.0715122544471092 0.116710762842684 0.116710762842684 0.187010653396718 0.119850681997132 0.464182048964475 green

AC005306.3 0.271267512388156 -0.271267512388156 0.26736478153568 0.148331940361808 -0.133367317801858 0.00470961746806382 0.00470961746806382 0.00536878397189146 0.127313000420512 0.170847749542265 brown

AC005498.3 0.0724418846017949 -0.0724418846017949 -0.112323797281082 -0.0814610018324534 0.0235577703343726 0.45837926270326 0.45837926270326 0.24936672615598 0.404208060401823 0.809667730366188 turquoise

AC005523.2 -0.454038952461731 0.454038952461731 -0.464909820007742 -0.367917405894795 0.0453512323872771 9.01576462429985e-07 9.01576462429985e-07 4.53701436131143e-07 9.66963755661776e-05 0.6427584012532 blue

AC005523.3 -0.067282231663621 0.067282231663621 -0.207265683014431 -0.174473426824012 0.00249588016733196 0.491088170931214 0.491088170931214 0.0321846343193238 0.0722749961517338 0.979644677786055 turquoise

AC005537.2 -0.136982560759787 0.136982560759787 -0.131627320278685 -0.0147280702921363 0.164539255070126 0.159440008727955 0.159440008727955 0.17654829439657 0.880317225304483 0.0903451536372771 turquoise

AC005592.3 -0.262843917359635 0.262843917359635 -0.29391352231645 -0.214117721498489 0.0600114490929455 0.00623399743345874 0.00623399743345874 0.00212115411223921 0.0267884041346081 0.539198241603386 turquoise

AC005606.14 -0.156832454257053 0.156832454257053 -0.139081569953557 -0.0809744066519803 0.062909519254246 0.106693952739261 0.106693952739261 0.153082719637251 0.407030634301531 0.519746693795254 yellow

AC005785.2 -0.219673605843723 0.219673605843723 -0.378226224748428 -0.230638158157714 0.153385942615227 0.022997415027914 0.022997415027914 5.90132057171322e-05 0.0168467734214567 0.114714996430232 turquoise

AC005838.2 -0.137972036478643 0.137972036478643 -0.119554123348509 -0.066347102669716 0.0596033177558766 0.156419075559349 0.156419075559349 0.219989259963282 0.497145773175787 0.5419661706768 turquoise

AC006026.13 0.287845957093432 -0.287845957093432 0.223428800733115 0.0791992305006387 -0.187365518531889 0.00264334837073392 0.00264334837073392 0.0207036518167169 0.417425616373598 0.0532964961406028 brown

AC006129.2 0.243774512082875 -0.243774512082875 0.415656598223069 0.199436478846002 -0.260201167324992 0.0113965008608279 0.0113965008608279 8.49740893503605e-06 0.0394469191801294 0.00679518456798125 blue

AC006538.1 -0.0923659559716949 0.0923659559716949 -0.128247079308481 -0.0802793902964073 0.048488620497394 0.344024409262159 0.344024409262159 0.188019709456201 0.411082220689281 0.619914950199934 green

AC007292.3 -0.367095005186945 0.367095005186945 0.00527804800714513 0.112468094600028 0.183161245255365 0.000100510218395551 0.000100510218395551 0.956970492475183 0.248755206096337 0.0589736843077178 turquoise

AC007349.5 -0.0881602226548361 0.0881602226548361 -0.164789375382521 -0.0887855544028159 0.0866754373114908 0.366533797856999 0.366533797856999 0.0898498942051639 0.363130874569992 0.374691869906974 turquoise

AC007362.3 -0.216153727001495 0.216153727001495 -0.225525534941709 -0.0726326881610797 0.201522146744016 0.02534160639043 0.02534160639043 0.0195105881990527 0.457193239121076 0.0373897547804359 turquoise

AC007365.3 -0.0599065910394988 0.0599065910394988 -0.117748055880992 -0.102546715073636 -0.00439643506437906 0.539908717484102 0.539908717484102 0.2270871173241 0.293228181341807 0.964152632273823 turquoise

AC007389.3 0.216417964637955 -0.216417964637955 -0.117597848846572 -0.198064613010768 -0.166623641985005 0.0251588161176194 0.0251588161176194 0.227684598427093 0.0408510155819346 0.086283403912172 green

AC007401.2 0.339157740510007 -0.339157740510007 0.125430060196612 0.0179528495929335 -0.150146589356306 0.000352111940424163 0.000352111940424163 0.197986309115475 0.854374892657881 0.122672946732676 brown

AC007680.2 -0.19826270809615 0.19826270809615 -0.224531522746536 -0.11991867809835 0.119887665732969 0.0406457238942934 0.0406457238942934 0.0200686016827699 0.218575800447072 0.218695791400681 blue

AC007787.2 -0.520065158437855 0.520065158437855 -0.287669997133652 -0.0849152449079691 0.270166306243781 9.4134039709459e-09 9.4134039709459e-09 0.00266008623163946 0.384505206206624 0.00488787645953215 blue

AC008088.4 -0.0863608982032374 0.0863608982032374 -0.191475608466256 -0.0910633845466061 0.121235398159212 0.376434182216764 0.376434182216764 0.0481892138444511 0.350900969497446 0.213524176304574 turquoise

AC008746.12 0.114477152340686 -0.114477152340686 0.181433838212731 0.100581084044272 -0.0906337949690374 0.240348268423135 0.240348268423135 0.0614453926931422 0.302627423881505 0.353187568302025 grey

AC008753.4 0.113263962750399 -0.113263962750399 -0.123809335722871 -0.140859435581557 -0.0606526880585909 0.245400958646566 0.245400958646566 0.203890213393147 0.147848351940077 0.534863535394956 green

AC009120.6 -0.184500105975308 0.184500105975308 0.000456269638967974 -0.0436797649396537 -0.0747435999023839 0.0571144141786468 0.0571144141786468 0.996278476968532 0.655067578199744 0.444185880856463 green

AC009133.15 0.24514560321656 -0.24514560321656 -0.139353321754948 -0.193166732622498 -0.126992148977201 0.0109283731717089 0.0109283731717089 0.152273758680091 0.0462088828255342 0.192413615732723 green

AC009502.4 -0.297779423441173 0.297779423441173 -0.235717561405385 -0.0579094481832968 0.241169377750416 0.00183906036389517 0.00183906036389517 0.0145178433272221 0.553528054012416 0.0123344855816134 turquoise

AC010145.4 -0.118010916341054 0.118010916341054 -0.165509456191249 -0.0747095209126338 0.111587062269055 0.226044185801261 0.226044185801261 0.0884360935694539 0.444394206823327 0.252505085431031 turquoise

AC010524.4 -0.269709802948199 0.269709802948199 -0.255315460917167 -0.123946780821716 0.157378880272645 0.00496352025707101 0.00496352025707101 0.00795146611864704 0.203384687891843 0.105463514256845 turquoise

AC012065.7 0.158743852245657 -0.158743852245657 -0.00490639045266926 -0.189293098826562 -0.314001707739119 0.102438351893476 0.102438351893476 0.95999784797295 0.0508483276243765 0.000989036787994612 yellow

AC012360.6 0.00263091851021534 -0.00263091851021534 -0.16309284817753 -0.145524572632038 -0.0120040704659036 0.978543618284055 0.978543618284055 0.093251758820525 0.134755033952169 0.902331180152526 turquoise

AC012499.1 -0.0404777725666983 0.0404777725666983 -0.137839689858784 -0.101626591782275 0.0260926129109494 0.678905485539614 0.678905485539614 0.156820641109468 0.2976037103812 0.789639058488042 turquoise

AC012531.25 0.0820734066359901 -0.0820734066359901 -0.0246522101546833 -0.0680016212496037 -0.0798448651673858 0.400672164119516 0.400672164119516 0.801004281318944 0.486454825716233 0.413627222714606 turquoise

AC013463.2 -0.0798416406530909 0.0798416406530909 -0.259764519061094 -0.18713949325531 0.0566015608545661 0.413646142904921 0.413646142904921 0.00689209387285053 0.0535898168780539 0.562536042898674 turquoise

AC013733.4 -0.155686625465335 0.155686625465335 -0.23356135250988 -0.180208646129431 0.0306292897236506 0.10931051325133 0.10931051325133 0.0154698866502383 0.0632492329654092 0.754140890158645 turquoise

AC016831.7 -0.489453805669904 0.489453805669904 -0.366955035392337 -0.136302722086589 0.297162793908342 8.79629115767206e-08 8.79629115767206e-08 0.000101173091649756 0.161540670623926 0.00188164656957398 blue

AC016999.2 -0.0807952862606801 0.0807952862606801 -0.245814055038926 -0.197180060567448 0.0194852434994452 0.408072561721708 0.408072561721708 0.0107063160897018 0.0417782999672211 0.84209969915643 green

AC017002.2 -0.147316733565872 0.147316733565872 -0.0509828690498513 -0.00116732088463502 0.0714263810091405 0.129966947783989 0.129966947783989 0.602007356674055 0.990479038941623 0.464720099972804 grey

AC017104.6 0.183533629612034 -0.183533629612034 0.0144915810854586 -0.0429699145956381 -0.0937479086469009 0.0584516519905933 0.0584516519905933 0.882225045377373 0.66032345346373 0.336822161138829 green

AC018755.16 0.0172017701697266 -0.0172017701697266 -0.053484129684485 0.0248687123973286 0.119188278855217 0.860404293791184 0.860404293791184 0.584284027179547 0.799293294965792 0.221414206310947 green

AC018755.17 -0.169506725952796 0.169506725952796 -0.234210008999084 -0.212245123012554 -0.0227748349478299 0.0809063751525971 0.0809063751525971 0.0151779440760877 0.0281805662556598 0.815879550780293 turquoise

AC018766.6 -0.215994354494004 0.215994354494004 -0.349236701279227 -0.237033285732457 0.100799211753935 0.0254524013705378 0.0254524013705378 0.000227005861320466 0.0139622704412464 0.30157474793256 turquoise

AC018816.3 -0.0935279076753597 0.0935279076753597 -0.151535775626322 -0.108835950714518 0.0335845419315503 0.337962286599714 0.337962286599714 0.119209659310402 0.264463581972061 0.731284244166276 yellow

AC024560.2 -0.115163844144132 0.115163844144132 -0.143630638142472 -0.0966093013116718 0.0429405291586648 0.237520577094693 0.237520577094693 0.139959849369565 0.322215892056253 0.660541387854268 turquoise

AC025442.3 0.0500263095305975 -0.0500263095305975 -0.21036058289389 -0.204665191595434 -0.0442572004835324 0.608847872583766 0.608847872583766 0.0296431706327825 0.0344601333165713 0.650804503240208 green

AC064852.4 0.0679701740978367 -0.0679701740978367 -0.0472848878399458 -0.0585623417442412 -0.0312476077290531 0.48665687815601 0.48665687815601 0.628638253720901 0.549057507805249 0.749340317228298 grey

AC067956.1 -0.124973676236128 0.124973676236128 -0.135793113110771 -0.0490522106212586 0.11231896101463 0.199636192751315 0.199636192751315 0.163128789294149 0.615848634191449 0.249387239772086 turquoise

AC068039.4 -0.266800699147878 0.266800699147878 -0.107138534419625 0.036077760560892 0.215453123963799 0.00547055722343673 0.00547055722343673 0.272030867432226 0.712181332415952 0.0258317508706717 brown

AC068138.1 -0.167821958755264 0.167821958755264 -0.2160311721984 -0.168571063907725 0.0251278190255828 0.0840149451329796 0.0840149451329796 0.0254267692589343 0.0826212022870182 0.797246860627235 turquoise

AC068831.3 0.0450156046128512 -0.0450156046128512 -0.123931439893021 -0.141892107411903 -0.0622284323515722 0.645222455663636 0.645222455663636 0.203441067463308 0.1448705056272 0.524285797152784 green

AC073283.7 0.645073658659894 -0.645073658659894 0.359989385924306 0.152750385061345 -0.259236020842098 6.41456302559114e-14 6.41456302559114e-14 0.000139795292395385 0.116243913119957 0.00701102374700132 brown

AC073321.4 -0.221446773772186 0.221446773772186 -0.104144291634964 0.0111602278352553 0.168878534093093 0.0218885514573104 0.0218885514573104 0.285732677938512 0.909166523079395 0.0820545144617456 blue

AC074212.6 0.0259103767983866 -0.0259103767983866 -0.175505051810486 -0.158132723520965 -0.0155177926481012 0.791074576666149 0.791074576666149 0.0705789315033754 0.1037842631297 0.873951349032968 green

AC079305.10 -0.365192820786274 0.365192820786274 -0.420879899748679 -0.258229770945557 0.168000657933493 0.000109874054371494 0.000109874054371494 6.36199401916038e-06 0.00724249503332 0.083680775670581 blue

AC079741.2 -0.168938613713022 0.168938613713022 0.119515231075208 0.157429153445824 0.0949398853965121 0.0819441474691261 0.0819441474691261 0.220140434636897 0.105350869226323 0.330687354878351 grey

AC079767.4 0.381835828211002 -0.381835828211002 0.331101150551324 0.0736769168467459 -0.351761151140932 4.94469035021142e-05 4.94469035021142e-05 0.000494892698023488 0.45073255342001 0.000202897791713754 blue

AC079807.4 -0.331521133336585 0.331521133336585 -0.320626748645197 -0.222499814274594 0.0842565895308613 0.000486299109191668 0.000486299109191668 0.000759948276691086 0.0212519754628434 0.388216783916634 blue

AC083843.1 0.46401186301524 -0.46401186301524 0.579027326235986 0.402011339236654 -0.151832403666776 4.80614355231873e-07 4.80614355231873e-07 6.43706073007748e-11 1.76974116332813e-05 0.118480036215907 blue

AC083949.1 -0.335407019081028 0.335407019081028 -0.298990508045188 -0.214501538650905 0.0666703871461697 0.000413048629373445 0.000413048629373445 0.0017579505183121 0.0265104065952931 0.495047165866004 blue

AC084219.3 -0.105181592804533 0.105181592804533 -0.2065604588443 -0.151435291169981 0.0405561949946564 0.280934817795067 0.280934817795067 0.0327888133022133 0.119457608717632 0.678317733808346 turquoise

AC087501.1 -0.0197048629796605 0.0197048629796605 -0.031420283172014 -0.0171274674859208 0.0161892134317031 0.840343466832466 0.840343466832466 0.748001377674952 0.861001211687315 0.868545392131044 yellow

AC090627.1 0.115232779768173 -0.115232779768173 0.249566332633233 0.186109934553722 -0.043664236598169 0.237237995565957 0.237237995565957 0.00953173857813125 0.0549427671842032 0.655182374345232 yellow

AC091133.1 0.453114705906269 -0.453114705906269 0.519870116813359 0.409788303077278 -0.0534657516955166 9.54743754947728e-07 9.54743754947728e-07 9.55515680472469e-09 1.16955723917257e-05 0.584413372123567 brown

AC091633.3 0.150933647240984 -0.150933647240984 -0.080064511208392 -0.15891041521281 -0.154254301495652 0.120701396311133 0.120701396311133 0.412339614596967 0.102073908038909 0.112651311749635 grey

AC092192.1 -0.411893163865921 0.411893163865921 -0.355682906901557 -0.235629873553046 0.112460925866082 1.04365917259279e-05 1.04365917259279e-05 0.000170104324561788 0.0145555435800071 0.248785562173935 blue

AC092620.2 -0.0731983419670275 0.0731983419670275 0.320344777414352 0.361561307015197 0.152016004535664 0.453687171261291 0.453687171261291 0.000768612304522487 0.000130041724304688 0.118030161082369 black

AC092660.1 -0.159951131964931 0.159951131964931 -0.132045856666807 -0.0543660282628516 0.0979106415752597 0.0998197749154189 0.0998197749154189 0.17516449742154 0.578092686928722 0.315709928704751 turquoise

AC092667.2 0.0802104136498056 -0.0802104136498056 0.122915996393068 0.035359458971179 -0.117002909089602 0.411485602086659 0.411485602086659 0.207197907592547 0.717667300561954 0.230061945539699 yellow

AC100830.4 0.406577103968503 -0.406577103968503 0.456204958809936 0.254307648339475 -0.225514889331635 1.38945123297762e-05 1.38945123297762e-05 7.87768273571933e-07 0.00821052032121196 0.0195164928662201 blue

AC104667.3 -0.346396694970972 0.346396694970972 -0.442515704845085 -0.26997329620408 0.179233824028833 0.000257279646866157 0.000257279646866157 1.82008812752646e-06 0.00491973241029189 0.064715067604681 blue

AC106801.1 -0.338128066709876 0.338128066709876 -0.280011104235426 -0.211382869507963 0.0446331341537816 0.000367957820299704 0.000367957820299704 0.0034878785404423 0.028841978540804 0.648035098106536 turquoise

AC108056.1 -0.199037056738043 0.199037056738043 -0.250311190210064 -0.211239611712181 0.00211347536257426 0.0398514953689371 0.0398514953689371 0.00931254320787805 0.0289531340854813 0.98276288492167 turquoise

AC112198.1 -0.0519498898320068 0.0519498898320068 -0.0950929283753668 -0.0181211083833674 0.106181166071752 0.595126961472029 0.595126961472029 0.329904875857984 0.85302530599832 0.276362752264832 grey

AC114730.11 -0.0954076304546939 0.0954076304546939 -0.32042099276683 -0.33079985635493 -0.0997307936978505 0.32829958197382 0.32829958197382 0.000766261593459196 0.000501143439148142 0.306753862002005 turquoise

AC114752.3 -0.167272416532576 0.167272416532576 -0.37573685702491 -0.308634644543029 0.0175093952230668 0.0850492708311176 0.0850492708311176 6.6588451021603e-05 0.00121904706124629 0.857933797787814 turquoise

AC124997.1 -0.027803118203755 0.027803118203755 -0.0675685807115395 -0.00149151965426353 0.0947569676700141 0.776199898363023 0.776199898363023 0.489241099105753 0.987834972710604 0.331624129051855 turquoise

AC136289.1 0.178038158464346 -0.178038158464346 -0.197211495428996 -0.349092070995262 -0.308156611524153 0.0665505838995854 0.0665505838995854 0.0417450481253335 0.000228464251351502 0.00124172998580111 green

AC139100.3 -0.0940549910777136 0.0940549910777136 -0.0411575489924623 0.113908118624111 0.252462762136579 0.335234829160106 0.335234829160106 0.673817203874991 0.242709109551931 0.00870408035360713 yellow

AC141928.1 -0.0892507916809089 0.0892507916809089 -0.227656494682237 -0.167878574241821 0.043040785607472 0.360611838385272 0.360611838385272 0.0183591811495644 0.0839089585129323 0.659797961974425 yellow

AC144652.1 0.362885786289454 -0.362885786289454 0.0229491940930537 -0.134915222401117 -0.261876759035553 0.00012231815589945 0.00012231815589945 0.814495180868947 0.165891767158055 0.00643441317543339 brown

AC145343.2 0.172874444740594 -0.172874444740594 -0.00428099705693203 -0.0862621609415004 -0.140148106309649 0.0749686932979386 0.0749686932979386 0.965093283128355 0.37698212757199 0.149926222442471 green

ACAA1 -0.648664150925519 0.648664150925519 -0.325365656039443 -0.0325227340077877 0.413305983525756 4.19507363827986e-14 4.19507363827986e-14 0.000627089137842689 0.739470690646078 9.66431624732523e-06 purple

ACAA2 0.381441758139646 -0.381441758139646 0.14312695133193 0.00834384674904838 -0.191925151658962 5.04160122445122e-05 5.04160122445122e-05 0.141369391184795 0.932025158163943 0.0476560569440032 red

ACACA -0.0203227931556514 0.0203227931556514 -0.277339060715503 -0.279153259868612 -0.0741616962668479 0.835406288930286 0.835406288930286 0.0038269927825019 0.00359368955388728 0.447750609930983 tan

ACACB 0.625690921072215 -0.625690921072215 0.349402926626629 0.0632794928706378 -0.39574786476957 5.78017026651409e-13 5.78017026651409e-13 0.000225340354471869 0.517289407529285 2.4522243776395e-05 brown

ACAD10 0.136632158934782 -0.136632158934782 0.0539368586529528 -0.00766666745791707 -0.0906631568106619 0.160520172094817 0.160520172094817 0.581101858778271 0.937530320740484 0.353030987443825 yellow

ACAD8 -0.127509119611101 0.127509119611101 -0.324360044008081 -0.342260165304892 -0.11349745630451 0.190594612557844 0.190594612557844 0.000653358224356378 0.000308091667243003 0.244422842669654 brown

ACAD9 0.479493577595641 -0.479493577595641 0.268581867014801 0.0659300246637842 -0.274884214109379 1.73841179597989e-07 1.73841179597989e-07 0.00515491450096142 0.49986012823281 0.00416425511275443 red

ACADL -0.238519619706423 0.238519619706423 -0.396129063650079 -0.358100644505367 -0.0370303326690187 0.0133569924252827 0.0133569924252827 2.40447672407191e-05 0.000152411832551924 0.704928770070466 turquoise

ACADM -0.0423109261200646 0.0423109261200646 -0.0584027927873984 -0.0873762545081569 -0.0641120088836463 0.665217572756091 0.665217572756091 0.550148361517912 0.370827573537596 0.511781773351606 yellow

ACADS 0.334336166869266 -0.334336166869266 0.007678319943242 -0.0716938927642734 -0.132657717929671 0.00043214827865904 0.00043214827865904 0.937435565642473 0.463045097754923 0.173155894407288 green

ACADSB 0.533851227249935 -0.533851227249935 0.452857686931883 0.210338815039677 -0.295272367388428 3.19443795263312e-09 3.19443795263312e-09 9.70049276353269e-07 0.0296604337179161 0.00201782698996542 turquoise

ACADVL -0.26187675805277 0.26187675805277 -0.33838526979228 -0.257482503214942 0.050490468687988 0.00643441338195617 0.00643441338195617 0.000363939250544153 0.00741873975134983 0.605524331744038 magenta

ACAN -0.33526449911608 0.33526449911608 -0.371335612238085 -0.164991793012796 0.254809591288335 0.000415544981031324 0.000415544981031324 8.22425883054768e-05 0.0894506692268 0.00808058191297441 yellow

ACAP1 0.0448535304760034 -0.0448535304760034 -0.328038701440053 -0.398860727943212 -0.204202834480502 0.646413721943051 0.646413721943051 0.000561899949814523 2.08698019479017e-05 0.0348785696808607 grey

ACAP2 -0.552907598663977 0.552907598663977 -0.128831016595832 0.0557864301747403 0.280114928318709 6.60509973320623e-10 6.60509973320623e-10 0.186000226139325 0.568185259685229 0.00347526376780909 brown

ACAP3 0.406804240955595 -0.406804240955595 0.491348813787666 0.303335984874043 -0.192957288451055 1.37270181936829e-05 1.37270181936829e-05 7.70796675357131e-08 0.00149290113723031 0.0464504194197103 blue

ACAT1 -0.372481166909809 0.372481166909809 -0.259391140744347 -0.138809772423481 0.138037535117677 7.78674975259468e-05 7.78674975259468e-05 0.00697592962719851 0.153895035281655 0.156220624443595 yellow

ACAT2 0.0422525316054035 -0.0422525316054035 -0.204684769790533 -0.301057488720954 -0.215923460972118 0.665651934895924 0.665651934895924 0.0344425086088503 0.00162697396269614 0.0255018187883709 red

ACBD3 -0.121038569577936 0.121038569577936 0.0507229690370876 0.0221029327579533 -0.0355425869557173 0.214273987827571 0.214273987827571 0.603862564833267 0.82121955586661 0.716267299270738 yellow

ACBD4 0.417401937903401 -0.417401937903401 0.320795596632622 0.131768286497536 -0.238392363897343 7.71826487459754e-06 7.71826487459754e-06 0.000754803022198045 0.176081327104279 0.0134079045302858 brown

ACBD5 -0.20480788689397 0.20480788689397 -0.0762267407498929 0.0624175613684431 0.215621389556937 0.0343318498388146 0.0343318498388146 0.435172780143059 0.523023341918679 0.0257133003493893 yellow

ACBD6 0.225501103530139 -0.225501103530139 0.268650539131213 0.187170648706106 -0.0693429966579937 0.0195241415502612 0.0195241415502612 0.00514307661885554 0.0535493066116924 0.477878061029448 blue

ACCS 0.227290139952071 -0.227290139952071 0.0733879504787467 -0.0567146190757471 -0.201861076973768 0.0185528543168379 0.0185528543168379 0.452515283168341 0.561754615147143 0.0370641105195968 brown

ACD 0.481089904042779 -0.481089904042779 0.456970291733327 0.228162019854614 -0.270963295761112 1.56086603234202e-07 1.56086603234202e-07 7.50922081495068e-07 0.0180948011308485 0.00475827367685339 blue

ACE -0.053733066571689 0.053733066571689 -0.182407145320164 -0.160719936725166 -0.00996823003969273 0.582533295431527 0.582533295431527 0.0600425157632032 0.098179861097821 0.91883320117631 turquoise

ACE2 -0.235252994947194 0.235252994947194 -0.321875554875646 -0.249259795267071 0.040666240510357 0.0147185478515198 0.0147185478515198 0.000722640777920802 0.00962325476978973 0.677493302071195 turquoise

ACER1 -0.12621480020697 0.12621480020697 -0.11296553163063 -0.06109711116455 0.0590215961062555 0.195172404663121 0.195172404663121 0.246655036245631 0.531869481296009 0.545923419312567 turquoise

ACER3 -0.739776398031923 0.739776398031923 -0.732002919897421 -0.414049251104923 0.351672221853894 8.9026794832263e-20 8.9026794832263e-20 3.33411379685849e-19 9.2799514551586e-06 0.000203705114883051 brown

ACHE -0.128902253111221 0.128902253111221 -0.389947862324831 -0.282784366854567 0.0818161908266372 0.18575494816938 0.18575494816938 3.29766379582536e-05 0.00316465960138416 0.402155037622083 turquoise

ACIN1 0.379254811718061 -0.379254811718061 0.491035343699417 0.362334347380151 -0.0924369587216573 5.61245875305653e-05 5.61245875305653e-05 7.87865675587525e-08 0.000125480522916764 0.343652023569341 blue

ACKR1 -0.117781822318098 0.117781822318098 -0.367205892017991 -0.285091166525118 0.0451591263684046 0.226952955816805 0.226952955816805 9.99879478569378e-05 0.00291658942414125 0.644168300348584 pink

ACKR2 -0.0162161849471096 0.0162161849471096 -0.201481768436182 -0.107993331001909 0.10692706354345 0.868328355252151 0.868328355252151 0.0374287097841103 0.268202013628226 0.272983762432905 turquoise

ACKR3 0.626136103111435 -0.626136103111435 0.471150498663578 0.213351867468173 -0.316500222779754 5.50496640926492e-13 5.50496640926492e-13 3.02588606876065e-07 0.0273505297170781 0.000896113044136898 blue

ACKR4 -0.2049462432118 0.2049462432118 -0.359927484870198 -0.212857418998396 0.157197485394838 0.0342078513887959 0.0342078513887959 0.000140192932555438 0.0277187509281229 0.105870741513903 turquoise

ACLY 0.525634897286537 -0.525634897286537 0.268399092842105 0.093309224931295 -0.228182323927827 6.11848682119436e-09 6.11848682119436e-09 0.00518653993579264 0.339097998893188 0.0180842514897786 blue

ACMSD -0.128642791256519 0.128642791256519 -0.258802140007311 -0.264751959453318 -0.076425057525801 0.186649449401319 0.186649449401319 0.00711001557902742 0.00585478545940841 0.433975538518491 turquoise

ACN9 -0.632932501282426 0.632932501282426 -0.662537047224822 -0.424812726963067 0.233397390160897 2.58862876896921e-13 2.58862876896921e-13 7.69878761699961e-15 5.0999060139036e-06 0.0155444483122356 brown

ACO1 0.360267781443322 -0.360267781443322 0.350920398797118 0.209943480579074 -0.149171293049144 0.000138019843509922 0.000138019843509922 0.00021065016861646 0.0299754410499829 0.125150320030838 blue

ACO2 0.177999887624641 -0.177999887624641 -0.127785004484304 -0.166587049883239 -0.098565828570777 0.0666100258865533 0.0666100258865533 0.189629011243055 0.0863534338413333 0.31246677638832 red

ACOT11 0.0876273490031069 -0.0876273490031069 -0.0820044825811944 -0.0488306621250091 0.0352486788418199 0.369448996956695 0.369448996956695 0.401069199918962 0.617445734854359 0.718514661563232 turquoise

ACOT12 -0.204929240747648 0.204929240747648 -0.299232642234123 -0.194663053570553 0.100667720896399 0.0342230690693914 0.0342230690693914 0.00174212828108459 0.0445134406892605 0.302209029753811 turquoise

ACOT13 -0.570899857978404 0.570899857978404 -0.335041842021229 -0.111813621080563 0.292750145427021 1.35783620701315e-10 1.35783620701315e-10 0.000419472806368296 0.251537110214438 0.00221339162972248 greenyellow

ACOT4 0.691545700420494 -0.691545700420494 0.477224740590077 0.172743558526402 -0.394123103019682 1.63524282317377e-16 1.63524282317377e-16 2.02415030381971e-07 0.0751927195022852 2.66588150142885e-05 brown

ACOT6 -0.31284578911582 0.31284578911582 -0.408196425865739 -0.301778007712401 0.0758751716659079 0.00103493313639179 0.00103493313639179 1.27411561281957e-05 0.00158344073612925 0.437299819208275 turquoise

ACOT7 -0.134829541002793 0.134829541002793 -0.307779011218846 -0.255050547150164 0.0105478075833032 0.166163278667472 0.166163278667472 0.0012599181326387 0.00801885254405683 0.914131468042501 salmon

ACOT8 -0.465262490103394 0.465262490103394 -0.541186312786832 -0.333720316484185 0.21317861559542 4.4352846215394e-07 4.4352846215394e-07 1.76181235644918e-09 0.000443497296487691 0.0274790760479892 greenyellow

ACOT9 -0.310666345649441 0.310666345649441 -0.139248319570443 0.167503906327705 0.484601555789614 0.00112678965410451 0.00112678965410451 0.152585952067742 0.084612339829254 1.22913933716182e-07 brown

ACOX1 -0.387630042798795 0.387630042798795 -0.090517303656259 -0.00276116837957858 0.125645553519899 3.70635401560062e-05 3.70635401560062e-05 0.353809219753797 0.977481640615237 0.197210700689631 purple

ACOX2 -0.567087386913636 0.567087386913636 -0.550747726257738 -0.313160710454816 0.261817186646218 1.91374465370636e-10 1.91374465370636e-10 7.93656120500092e-10 0.00102224026926845 0.00644694257230488 blue

ACOX3 -0.0144882727935301 0.0144882727935301 -0.140618235305599 -0.104922305725343 0.0245032170020121 0.882251739033985 0.882251739033985 0.148550479884887 0.282129021565199 0.802182300291908 yellow

ACOXL -0.267395877031735 0.267395877031735 -0.219137578954238 -0.159496786369262 0.0449916946195657 0.00536322296574944 0.00536322296574944 0.0233419480915549 0.10079900123606 0.645398140944932 yellow

ACP1 0.162731566075082 -0.162731566075082 -0.016337155704222 -0.0698733925369642 -0.094991877105835 0.0939891898173663 0.0939891898173663 0.867355037126602 0.474509329746172 0.330421398727978 purple

ACP2 0.103559043396218 -0.103559043396218 0.0472521931999059 0.197667243699289 0.267234923809713 0.288463590241879 0.288463590241879 0.628875909446484 0.0412654325605375 0.00539206276025004 yellow

ACP5 0.398301325106068 -0.398301325106068 0.207225038938786 0.0433919514222653 -0.224768813444437 2.14860745903666e-05 2.14860745903666e-05 0.0322191977089618 0.657196596272204 0.019934160080563 brown

ACP6 -0.266674655504897 0.266674655504897 -0.294988670498971 -0.333240192418923 -0.140487936658206 0.00549353160659753 0.00549353160659753 0.00203901559087025 0.000452534616499217 0.148930818565319 brown

ACPP -0.488602675131269 0.488602675131269 -0.400821759821825 -0.157379346700518 0.310176104100599 9.33146245186351e-08 9.33146245186351e-08 1.88378905642119e-05 0.105462468718386 0.00114844913669335 brown

ACPT -0.0980452932342078 0.0980452932342078 -0.223287185151219 -0.123687350420489 0.111703903553095 0.315041635399719 0.315041635399719 0.0207864451952121 0.204339631865757 0.25200556059807 turquoise

ACR 0.08185552999474 -0.08185552999474 -0.0343467775675338 -0.102125180075636 -0.123764508285683 0.40192803367543 0.40192803367543 0.725425952339365 0.295227427462087 0.20405528424687 yellow

ACRBP -0.165530151481009 0.165530151481009 -0.250840644670775 -0.21637431305612 -0.00583334446526044 0.0883957234228172 0.0883957234228172 0.00915944349329834 0.0251889349480358 0.952448449730185 salmon

ACRC -0.320550602351388 0.320550602351388 -0.0219601118160541 0.057507537455524 0.129159031351258 0.000762279144395501 0.000762279144395501 0.822355700562697 0.556288761620049 0.184872778429899 brown

ACRV1 -0.439380613778587 0.439380613778587 -0.4132857202133 -0.227947318916806 0.208429408898775 2.19372943598304e-06 2.19372943598304e-06 9.67500191514159e-06 0.0182066814454685 0.0312082140639622 turquoise

ACSBG1 -0.0381719738983233 0.0381719738983233 -0.193026150518055 -0.179143084131169 -0.025926883571859 0.69627144857015 0.69627144857015 0.0463708905311066 0.0648529068148962 0.79094451994696 salmon

ACSBG2 0.0975091453442384 -0.0975091453442384 0.00844544259609569 -0.0200522231298891 -0.046171133837512 0.317708048448469 0.317708048448469 0.931199499717492 0.837567331789591 0.636755301270154 green

ACSF2 0.721059871167116 -0.721059871167116 0.507116633046318 0.223569464614721 -0.350954696388598 1.98282599970894e-18 1.98282599970894e-18 2.48855919909512e-08 0.0206216955018216 0.000210328628673185 brown

ACSF3 0.184645073555723 -0.184645073555723 0.154128237614202 0.139448879775989 0.0146063662152685 0.0569160142920197 0.0569160142920197 0.11294910425374 0.151990061489362 0.881298959009301 brown

ACSL1 -0.803383411757025 0.803383411757025 -0.536014440533743 -0.195385422881384 0.440366240049665 2.20225681951738e-25 2.20225681951738e-25 2.68423530165811e-09 0.0437136466382082 2.06907563472961e-06 brown

ACSL3 -0.521945810652468 0.521945810652468 -0.554771945038213 -0.338610299234657 0.224445434550838 8.14604809067824e-09 8.14604809067824e-09 5.63092820896544e-10 0.000360456577143142 0.0201175690403361 brown

ACSL4 -0.77591062423103 0.77591062423103 -0.496672249445636 -0.124327623394881 0.504243862735511 9.76294018055726e-23 9.76294018055726e-23 5.29585364697845e-08 0.201988642693197 3.07085644738582e-08 brown

ACSL5 0.319693897099636 -0.319693897099636 0.315194941933548 0.288772197832498 0.0359709061722989 0.000788957841599993 0.000788957841599993 0.000943613844959251 0.00255679565384972 0.712996502883061 turquoise

ACSL6 0.185556522754871 -0.185556522754871 -0.116539613567748 -0.267299188099327 -0.285578308852764 0.0556815309651379 0.0556815309651379 0.231925259966199 0.00538053129724738 0.00286649554537753 pink

ACSM1 -0.140990932024909 0.140990932024909 -0.23551012721034 -0.145321022005427 0.0926092113881719 0.147466623355584 0.147466623355584 0.0146071643619139 0.135307245141586 0.342749670772717 turquoise

ACSM2A -0.00311362612976604 0.00311362612976604 -0.214699546090942 -0.199304778942877 -0.0289179022806064 0.974608118508287 0.974608118508287 0.0263679534683271 0.0395799370592299 0.767476362338009 green

ACSM2B -0.315349625508374 0.315349625508374 -0.2463906526008 -0.0987498657857194 0.187266084551221 0.000937867065136699 0.000937867065136699 0.0105179592167168 0.311559704380917 0.0534253717784363 blue

ACSM3 0.00907602085006594 -0.00907602085006594 -0.284699192985271 -0.349740989308182 -0.183290511877054 0.926076524347659 0.926076524347659 0.00295746710860817 0.000221987928681221 0.0587920394720066 pink

ACSM5 -0.258083722400313 0.258083722400313 -0.496461307601694 -0.388668146007246 0.05558381778217 0.00727664683759505 0.00727664683759505 5.37585963472102e-08 3.51777651566177e-05 0.569593592013893 yellow

ACSS1 0.562371912147611 -0.562371912147611 0.406001281249987 0.173558284246554 -0.290192064397529 2.90813299601333e-10 2.90813299601333e-10 1.43277813171193e-05 0.0738069478742516 0.00242906823073701 brown

ACSS2 -0.43067119337399 0.43067119337399 -0.625795530232288 -0.374804832377376 0.265316114269755 3.64973540921998e-06 3.64973540921998e-06 5.71433082381421e-13 6.96509640567031e-05 0.0057466596757472 brown

ACSS3 -0.43098930270662 0.43098930270662 -0.282721531412046 -0.132215650117525 0.18281365152347 3.58338810030856e-06 3.58338810030856e-06 0.00317167436067022 0.174605393741837 0.0594643998058722 blue

ACTA1 -0.211746594991574 0.211746594991574 -0.313144275237302 -0.208960439701258 0.0964477677674241 0.0285613850080756 0.0285613850080756 0.00102289916065169 0.0307710485966102 0.323029441394239 turquoise

ACTA2 -0.0543453935106952 0.0543453935106952 0.124737137392632 0.284666339691888 0.303232277636291 0.578237204391932 0.578237204391932 0.200495193818655 0.00296091656635299 0.00149877863755601 grey

ACTA2-AS1 -0.235541417552527 0.235541417552527 -0.157819663741104 -0.138883705008074 -0.00833278095054694 0.014593660254289 0.014593660254289 0.104479070371346 0.15367375544297 0.932115093079787 turquoise

ACTB -0.00513950255182937 0.00513950255182937 -0.138549396330875 -0.100913738673298 0.0283235638718031 0.958098952316733 0.958098952316733 0.15467624045354 0.30102300856698 0.772123710545873 brown

ACTC1 -0.146688412838098 0.146688412838098 -0.282126309824401 -0.183294192664267 0.0953207389115872 0.131630578144587 0.131630578144587 0.00323881916776253 0.0587868739594102 0.328742315485474 turquoise

ACTG2 -0.186587713760661 0.186587713760661 -0.368684619765498 -0.245930387056926 0.113710100029111 0.054311463801975 0.054311463801975 9.32602804256147e-05 0.0106680776796037 0.243534417657817 turquoise

ACTL10 0.381372094680931 -0.381372094680931 0.11035926318588 -0.051503047902963 -0.246253714970929 5.05891652466458e-05 5.05891652466458e-05 0.257795361798508 0.598301865526099 0.0105624275319248 brown

ACTL6A 0.132596824115136 -0.132596824115136 0.209213629838575 0.0846871349170762 -0.157589978062512 0.173355031391137 0.173355031391137 0.030564443861809 0.38578819646034 0.104991148046041 red

ACTL6B -0.0816641070482862 0.0816641070482862 -0.29336443161323 -0.233684045318443 0.0260338151323119 0.403033339157019 0.403033339157019 0.00216424651543776 0.0154142953704864 0.790102146250475 green

ACTL7A -0.251973465436778 0.251973465436778 -0.309700342395087 -0.193109285669407 0.11837473325903 0.00883928894417869 0.00883928894417869 0.00116983083705128 0.0462750282095344 0.224606262706847 turquoise

ACTL7B -0.0287229184142824 0.0287229184142824 -0.277338889481748 -0.211904000134923 0.0399016850805774 0.769000112935211 0.769000112935211 0.00382701542848063 0.028440676232401 0.68322896357752 turquoise

ACTL8 0.117930188726502 -0.117930188726502 -0.133703333382573 -0.177883385978506 -0.109204579132487 0.226364123520632 0.226364123520632 0.169762735932389 0.0667912407040529 0.262839283115972 green

ACTL9 -0.227503299647431 0.227503299647431 -0.27666287918896 -0.13871908584767 0.163059599411847 0.0184399543684415 0.0184399543684415 0.00391736008540588 0.154166785608257 0.0933194327714744 blue

ACTN1 -0.111489060489114 0.111489060489114 -0.0423893073009704 -0.023497843886098 0.0211776358998883 0.252924590978059 0.252924590978059 0.664634715743443 0.810142775455462 0.828586776686818 yellow

ACTN2 -0.134421206716463 0.134421206716463 -0.383555382598015 -0.301212710426068 0.0413552543537544 0.167461755524639 0.167461755524639 4.54182628460297e-05 0.0016175048756149 0.67234007835839 yellow

ACTN3 -0.271389066786888 0.271389066786888 -0.227015247692485 -0.0725100016240334 0.203875158854171 0.00469030058671266 0.00469030058671266 0.0186993306478653 0.457955656270816 0.0351777005629135 yellow

ACTN4 0.349202421181738 -0.349202421181738 0.291703619557415 0.205642683550627 -0.071204343629515 0.000227350746413917 0.000227350746413917 0.00229944353118464 0.0335893979959549 0.466112887397834 brown

ACTR10 -0.568822579102199 0.568822579102199 -0.551568497177763 -0.324982265459477 0.242948010904686 1.63790181735579e-10 1.63790181735579e-10 7.40273861341274e-10 0.000636987853695207 0.011687087418515 yellow

ACTR1A -0.499578305251873 0.499578305251873 -0.640318406932569 -0.397687374006006 0.247414694524775 4.30303681216232e-08 4.30303681216232e-08 1.11606669641464e-13 2.21820720682022e-05 0.0101905894373201 brown

ACTR1B 0.65733565106385 -0.65733565106385 0.484956948037214 0.184688426070075 -0.38499605541155 1.46895804272842e-14 1.46895804272842e-14 1.19959580383333e-07 0.0568567927559646 4.22813798107614e-05 brown

ACTR2 -0.522653344011552 0.522653344011552 -0.337176680394312 -0.168162583395987 0.200248694823267 7.71297002581855e-09 7.71297002581855e-09 0.00038318034721884 0.0833788901497813 0.0386348160822267 brown

ACTR3 -0.621253873819827 0.621253873819827 -0.311747248764357 -0.10465031940568 0.271359980313871 9.35974254618545e-13 9.35974254618545e-13 0.00108034310386296 0.283385355836495 0.00469491643361803 brown

ACTR3B 0.28939276664061 -0.28939276664061 0.208911197599212 0.00848759298714023 -0.286398977787237 0.00250024429112957 0.00250024429112957 0.0308113674362497 0.9308569694216 0.00278385262847656 blue

ACTR3BP2 -0.313117774642223 0.313117774642223 -0.309630400504045 -0.128894376938287 0.227191001360134 0.00102396239111926 0.00102396239111926 0.00117300455512806 0.185782055414579 0.018605565820252 blue

ACTR3C -0.140450147774417 0.140450147774417 -0.183957640420473 -0.144972955007494 0.0189731307867165 0.149041260373482 0.149041260373482 0.0578618588432493 0.136255503975197 0.846197908120227 grey

ACTR5 0.658615144865274 -0.658615144865274 0.642234197639109 0.307137375319138 -0.403757814407577 1.25458366865217e-14 1.25458366865217e-14 8.93911019720192e-14 0.00129138101260439 1.61399371062468e-05 blue

ACTR6 0.0210682280462604 -0.0210682280462604 0.108344492170295 0.0565994116078074 -0.0599968123460134 0.829458879415174 0.829458879415174 0.266639698763753 0.562550902917786 0.539297386565324 yellow

ACTR8 0.0769301155115203 -0.0769301155115203 -0.0848891942814334 -0.165919911519689 -0.159196632979158 0.430934983690301 0.430934983690301 0.384651596067531 0.0876381524714207 0.101450032956619 grey

ACTRT2 -0.244117696528173 0.244117696528173 -0.22247024810256 -0.127659766049144 0.103789885135577 0.0112777116440919 0.0112777116440919 0.0212696293241243 0.190066906424427 0.287384361821805 turquoise

ACTRT3 0.0969009302101851 -0.0969009302101851 -0.106981209020685 -0.129748447379789 -0.0660364840295954 0.32075046914198 0.32075046914198 0.272739567020389 0.182859384933707 0.499166551730657 green

ACVR1 0.39961055626771 -0.39961055626771 0.515613864800943 0.371816141704665 -0.111743334507817 2.00697308233036e-05 2.00697308233036e-05 1.32091911155773e-08 8.0380194052888e-05 0.251837137071453 turquoise

ACVR1B -0.67064451268625 0.67064451268625 -0.664762827367602 -0.401247782740882 0.276571416296827 2.74034537564265e-15 2.74034537564265e-15 5.81630673541686e-15 1.84217470750604e-05 0.0039297290468389 brown

ACVR1C 0.582613558528307 -0.582613558528307 0.464468014784775 0.13332210238118 -0.442619675185745 4.60045056366762e-11 4.60045056366762e-11 4.66758722694963e-07 0.170994142638923 1.80879484514279e-06 blue

ACVR2A 0.716767373156025 -0.716767373156025 0.621167438203074 0.363291543581052 -0.278180539322354 3.89982270616741e-18 3.89982270616741e-18 9.44732372406069e-13 0.000120038701337771 0.00371714415288871 blue

ACVR2B 0.474918615837578 -0.474918615837578 0.163725301128461 -0.128482771138024 -0.45365909735996 2.36010839903569e-07 2.36010839903569e-07 0.0919718516082996 0.187202684168118 9.23078339267703e-07 brown

ACVR2B-AS1 -0.161383328378455 0.161383328378455 -0.255617214907742 -0.209606889911893 0.0125224971241657 0.0967819144258082 0.0967819144258082 0.00787531794972194 0.0302458700994133 0.898135294294113 turquoise

ACVRL1 -0.414927385996324 0.414927385996324 -0.546744408497003 -0.293721572067139 0.289024447886367 8.84439404702777e-06 8.84439404702777e-06 1.1116353466491e-09 0.00213612932148324 0.00253367092558508 blue

ACY1 0.0593483917010239 -0.0593483917010239 -0.116891219001926 -0.145331234391546 -0.0781982796929598 0.543698605907181 0.543698605907181 0.230510185749802 0.135279499031953 0.423354286131628 green

ACY3 -0.0157103813307615 0.0157103813307615 -0.161057047278727 -0.15418445879407 -0.0296235409947319 0.872400113094932 0.872400113094932 0.0974675038523171 0.112816221166938 0.761969410796767 turquoise

ACYP1 0.439813968234221 -0.439813968234221 0.408148393572105 0.141042564088103 -0.348434512831863 2.13807135501836e-06 2.13807135501836e-06 1.2774030888927e-05 0.147316940858999 0.000235204706051029 turquoise

ADA 0.576652426738856 -0.576652426738856 0.630241652296589 0.452631727140385 -0.139713082275554 8.02285124918113e-11 8.02285124918113e-11 3.49758816385809e-13 9.83697225247613e-07 0.151207749035629 blue

ADAD1 0.0824538285351939 -0.0824538285351939 -0.0318457180441679 0.0136828246264968 0.0690600409868749 0.398484941272968 0.398484941272968 0.744705723461554 0.888754520465812 0.47968047072807 yellow

ADAD2 0.0794653740868006 -0.0794653740868006 -0.151966620224206 -0.227773519601772 -0.167528937542197 0.415857393421707 0.415857393421707 0.118151036846043 0.0182976847181516 0.0845652014895662 turquoise

ADAL 0.446181747247537 -0.446181747247537 0.135795283790942 -0.0795736819173506 -0.33048853841763 1.4596152327699e-06 1.4596152327699e-06 0.16312200018266 0.415220183866439 0.000507678271752375 brown

ADAM10 -0.322173487989222 0.322173487989222 -0.0914541802371852 0.0596382444802841 0.232832116171295 0.000713991566045738 0.000713991566045738 0.348828914332746 0.541729026045906 0.0158039005208527 green

ADAM11 0.0144311465232988 -0.0144311465232988 -0.283933672599641 -0.265907104823497 -0.0421993683866705 0.88271269525189 0.88271269525189 0.00303878960286675 0.00563529744676311 0.66604748071257 green

ADAM12 0.425237000657769 -0.425237000657769 0.242931099865325 0.110079400558925 -0.163068514146857 4.97886345270225e-06 4.97886345270225e-06 0.0116931001260411 0.259011746122534 0.0933012840675056 brown

ADAM15 -0.389860727588229 0.389860727588229 -0.65702508083522 -0.525346640227469 0.0549424031571412 3.31223212772319e-05 3.31223212772319e-05 1.52612051479576e-14 6.25763660343409e-09 0.574062773355078 blue

ADAM17 -0.571721228659882 0.571721228659882 -0.0813669221576642 0.240915857227051 0.525778967025786 1.26034218915854e-10 1.26034218915854e-10 0.404752882654636 0.0124292673636787 6.05005672543061e-09 purple

ADAM18 -0.109663589267606 0.109663589267606 -0.326919430108853 -0.326501868571124 -0.083084242733829 0.260826239865432 0.260826239865432 0.000588399582394354 0.000598575888777207 0.394876039959029 turquoise

ADAM19 -0.192456679047997 0.192456679047997 -0.184739056533552 -0.173590076961303 -0.0284401625392952 0.0470319819558945 0.0470319819558945 0.0567876931856071 0.0737532915266943 0.771211341852498 turquoise

ADAM2 -0.237066605224182 0.237066605224182 -0.285854824381178 -0.154473940666048 0.14957207073536 0.0139484446107296 0.0139484446107296 0.00283840656009829 0.112133930120057 0.124127682817313 blue

ADAM20 -0.190525811747968 0.190525811747968 -0.33352705749398 -0.287470925637925 -0.00736881232582415 0.0493319200021916 0.0493319200021916 0.000447114835130685 0.00267913758969445 0.939952696567638 turquoise

ADAM21 -0.0772750746347235 0.0772750746347235 -0.177324533915602 -0.13844404100485 0.020496532277612 0.428865276840038 0.428865276840038 0.0676660889438028 0.154993179864593 0.83401927891719 turquoise

ADAM22 -0.105299457952438 0.105299457952438 -0.245001508467527 -0.197703112634489 0.0174281691665597 0.280393083806857 0.280393083806857 0.0109767659912899 0.0412278812637782 0.858585981255021 turquoise

ADAM23 0.277547139839603 -0.277547139839603 0.113707547191387 -0.0177929035856874 -0.193897810512837 0.00379956244509532 0.00379956244509532 0.243545070128206 0.855658192870255 0.045373939311105 grey

ADAM28 0.710807970123191 -0.710807970123191 0.548849543831164 0.178726918119793 -0.487101519462971 9.77277897317263e-18 9.77277897317263e-18 9.31655545259069e-10 0.0654881488235429 1.03518978548809e-07 blue

ADAM30 -0.0348976855630434 0.0348976855630434 -0.195217898997154 -0.15834415150824 0.0125065937697714 0.72120167046656 0.72120167046656 0.0438980525868782 0.103317069297908 0.898263966961229 turquoise

ADAM32 -0.0709483178167139 0.0709483178167139 -0.0939956987957116 -0.0895095172313368 -0.016482796161062 0.467721707995286 0.467721707995286 0.335540944845916 0.359215652146335 0.866183492192261 turquoise

ADAM33 -0.279926805500427 0.279926805500427 -0.202667967337911 -0.0997006760624976 0.122700441942328 0.00349815114936837 0.00349815114936837 0.0362984174024619 0.306900692628165 0.20800173864979 turquoise

ADAM3A 0.00230645896637783 -0.00230645896637783 -0.152056291771978 -0.197786901665324 -0.116538566852974 0.981189221683973 0.981189221683973 0.117931622658858 0.0411402734209023 0.231929481638129 yellow

ADAM5 -0.186847332699998 0.186847332699998 -0.270182888116167 -0.155130181254579 0.12589419653186 0.0539709309036821 0.0539709309036821 0.00488514824959529 0.110599090681159 0.196318505978331 turquoise

ADAM6 0.00497168181073495 -0.00497168181073495 -0.0407325591951973 0.0683962332272976 0.174656722747789 0.959465971503044 0.959465971503044 0.676996643842769 0.483923191268995 0.0719712647419357 turquoise

ADAM7 -0.332653550402811 0.332653550402811 -0.41804615219966 -0.310785270577551 0.0747794990171041 0.000463806730454674 0.000463806730454674 7.44808124255135e-06 0.00112159181175732 0.44396648784435 yellow

ADAM8 -0.268529921206454 0.268529921206454 0.135142686647478 0.217500646696101 0.174328263512005 0.00516388517289198 0.00516388517289198 0.165172562973365 0.0244215559062951 0.0725162730722225 brown

ADAM9 -0.803912295338073 0.803912295338073 -0.650265566640588 -0.305956567928317 0.417324357940528 1.94026867254792e-25 1.94026867254792e-25 3.46486178076673e-14 0.00135115660457805 7.75141792758051e-06 brown

ADAMDEC1 -0.231849997718401 0.231849997718401 -0.177736874452602 0.14213832206483 0.496994914239041 0.0162636106119379 0.0162636106119379 0.0670197027339429 0.144167245897531 5.17566966016245e-08 grey

ADAMTS1 0.625621236116679 -0.625621236116679 0.409716566561846 0.182168601057799 -0.280937192184004 5.82443549839757e-13 5.82443549839757e-13 1.17408996717536e-05 0.0603838965874085 0.00337679420208229 brown

ADAMTS10 0.48064362704966 -0.48064362704966 0.114910371273549 -0.0722933374469444 -0.288069559262506 1.60867975731307e-07 1.60867975731307e-07 0.238561630116524 0.459303799109006 0.00262221554774166 green

ADAMTS12 -0.150046754072033 0.150046754072033 -0.325309557578317 -0.2238242982186 0.088752534149347 0.122924792364058 0.122924792364058 0.000628528673552494 0.0204739293653958 0.36331007494382 turquoise

ADAMTS13 0.0493928320886609 -0.0493928320886609 -0.171135004738465 -0.128389460034099 0.0286386300085921 0.613396655013131 0.613396655013131 0.0779901690525454 0.187525837389821 0.769659078673939 green

ADAMTS14 -0.283710197380346 0.283710197380346 -0.179754303266254 -0.0036735268288952 0.252583517593425 0.00306290586078702 0.00306290586078702 0.0639290253142204 0.970044035441728 0.00867099327473856 turquoise

ADAMTS15 -0.152779072754087 0.152779072754087 -0.23608267098495 -0.137881189738887 0.106052479870003 0.116174562755515 0.116174562755515 0.0143617800155591 0.156694639526586 0.276948544208976 turquoise

ADAMTS16 0.0515292115203899 -0.0515292115203899 -0.0428370546871467 -0.0759097602588804 -0.0670751937102404 0.598115758937199 0.598115758937199 0.661309022553987 0.437090292438376 0.492425949132153 turquoise

ADAMTS17 0.144863077897014 -0.144863077897014 0.158428505452795 0.116427457459042 -0.030632621776573 0.136555896030365 0.136555896030365 0.103131132363678 0.232377920461802 0.754114995060887 yellow

ADAMTS18 -0.318779669817014 0.318779669817014 -0.383636420663294 -0.193463006299704 0.224229655180649 0.000818367700777218 0.000818367700777218 4.52361915872171e-05 0.0458689930407255 0.0202407571586296 turquoise

ADAMTS19 -0.186622326493403 0.186622326493403 -0.289339336713341 -0.228955115861846 0.028259269783154 0.0542659616912058 0.0542659616912058 0.00250506851769471 0.017686649447151 0.772626935984415 turquoise

ADAMTS2 -0.671136228805663 0.671136228805663 -0.412225342060871 -0.052555577637617 0.504390185673758 2.5712459411125e-15 2.5712459411125e-15 1.02499621595073e-05 0.590835570470089 3.03828788089808e-08 blue

ADAMTS20 -0.18473579149692 0.18473579149692 -0.284349128330335 -0.174889889074057 0.112775951051441 0.0567921471719465 0.0567921471719465 0.00299440901473358 0.071586385373186 0.247453993190589 turquoise

ADAMTS3 -0.375702113637646 0.375702113637646 -0.680862610861705 -0.524851108581618 0.0901047019186564 6.67003228684295e-05 6.67003228684295e-05 7.11120931434371e-16 6.50396105530251e-09 0.356016530758715 brown

ADAMTS4 -0.47136949938942 0.47136949938942 -0.54462123199256 -0.323735718650827 0.235059508473307 2.98274145518043e-07 2.98274145518043e-07 1.32675663633518e-09 0.000670170679026698 0.0148028472938539 blue

ADAMTS5 0.706730734834047 -0.706730734834047 0.419317333610682 0.0985443944276509 -0.436598257681626 1.80815690027504e-17 1.80815690027504e-17 6.9408820964722e-06 0.312572531129101 2.5850948828286e-06 brown

ADAMTS6 0.158313978285467 -0.158313978285467 0.0122108189445595 -0.0274211698826033 -0.0640913198027872 0.103383642281763 0.103383642281763 0.900657532492175 0.779195236772063 0.511918278633376 yellow

ADAMTS7 -0.0587959181958477 0.0587959181958477 -0.197406682866379 -0.13105264696637 0.0619480288073009 0.547462419583381 0.547462419583381 0.0415390724403295 0.178461382869593 0.526160347333533 green

ADAMTS8 -0.116584965811633 0.116584965811633 -0.26101374481294 -0.138336280339233 0.141176942853465 0.23174239425074 0.23174239425074 0.0066180500507021 0.155317857829684 0.146927910558644 yellow

ADAMTS9 -0.274250630936334 0.274250630936334 -0.348547812369057 -0.257799213015368 0.0645855675290256 0.00425550732872298 0.00425550732872298 0.000234030342428031 0.00734358534519035 0.508662389125908 turquoise

ADAMTS9-AS2 -0.231572507409272 0.231572507409272 -0.407931398076134 -0.30929549026316 0.0627429515655381 0.0163955785333235 0.0163955785333235 1.29235457789707e-05 0.00118831055616482 0.520854935360369 turquoise

ADAMTSL1 -0.171341029690766 0.171341029690766 -0.367387614603816 -0.277615994910591 0.0580996731002358 0.077627273013586 0.077627273013586 9.91375009672292e-05 0.00379052437575888 0.552223717780891 turquoise

ADAMTSL2 0.00683981741534187 -0.00683981741534187 -0.0616266972864202 -0.025387807406413 0.0456704785842055 0.944256206297129 0.944256206297129 0.528312651632913 0.795194841969249 0.640418203380697 turquoise

ADAMTSL3 -0.063401126627941 0.063401126627941 -0.216236846989398 -0.17703748237737 0.0110637888376555 0.516482838820393 0.516482838820393 0.0252839851862959 0.0681190490667 0.909948135570193 turquoise

ADAMTSL4 0.176126093796207 -0.176126093796207 0.192265388916572 0.275678225780447 0.190758565475053 0.0695735073179521 0.0695735073179521 0.047255793752989 0.00405236955117446 0.0490498402860782 yellow

ADAMTSL5 0.186289334492649 -0.186289334492649 0.0149137559022878 -0.00117522313001425 -0.0234664832248323 0.0547050174892975 0.0547050174892975 0.878819730243453 0.990414589298099 0.810391403164457 green

ADAP1 -0.112198736910744 0.112198736910744 0.0393872609491433 0.154659166887342 0.205611709213777 0.249897558680562 0.249897558680562 0.687098343786497 0.111699048831837 0.033616702166301 yellow

ADAP2 -0.231686538770427 0.231686538770427 0.26295320015692 0.550552843054867 0.555204239652201 0.0163412360752279 0.0163412360752279 0.00621170370832445 8.06864321562405e-10 5.42562975644578e-10 grey

ADAR 0.347876663515768 -0.347876663515768 0.641212280640383 0.610861236954141 0.112869220118052 0.000241066765576436 0.000241066765576436 1.00646325009678e-13 2.81081584147729e-12 0.247060702772094 black

ADARB1 0.659368030963591 -0.659368030963591 0.479463761762706 0.227050780622876 -0.305234671594332 1.14296146059479e-14 1.14296146059479e-14 1.74190424856809e-07 0.0186803409886625 0.00138892896361137 brown

ADARB2 -0.15301760354683 0.15301760354683 -0.101817757914765 0.0229471547893264 0.185520923522058 0.115599172551668 0.115599172551668 0.296691123368025 0.814511369162134 0.0557293311800315 turquoise

ADARB2-AS1 -0.217467011306722 0.217467011306722 -0.332695395586892 -0.202361567757303 0.135790449021652 0.024444179316906 0.024444179316906 0.000462994218426482 0.0365875935542104 0.163137121899438 turquoise

ADAT1 0.425053050752779 -0.425053050752779 0.334389311575215 0.240824694111479 -0.0729908361936363 5.03100641279098e-06 5.03100641279098e-06 0.000431181549955865 0.0124635048932678 0.454971606152198 brown

ADAT2 0.548914339039504 -0.548914339039504 0.48610314229191 0.202592269220096 -0.356279073448487 9.26586416813045e-10 9.26586416813045e-10 1.10885752149142e-07 0.0363696811795544 0.00016557303848123 blue

ADAT3 -0.379723861216107 0.379723861216107 -0.436772044785388 -0.192341322470168 0.302637997006398 5.4851646532827e-05 5.4851646532827e-05 2.55883280122656e-06 0.0471668451243704 0.0015328659145697 blue

ADCK1 0.657208062487497 -0.657208062487497 0.442737473365061 0.176489866518726 -0.338113308446278 1.49218583823123e-14 1.49218583823123e-14 1.79607976554739e-06 0.0689899878726645 0.000368189640727787 brown

ADCK2 0.733856632248518 -0.733856632248518 0.571130403829022 0.304285014434994 -0.306218977478539 2.44372132121153e-19 2.44372132121153e-19 1.32976147244723e-10 0.00144008001130535 0.00133765945017508 brown

ADCK3 0.438801880262643 -0.438801880262643 0.545214080326398 0.334202707462703 -0.218159670990308 2.27020020869136e-06 2.27020020869136e-06 1.2629681596993e-09 0.000434584770959391 0.0239818859433566 black

ADCK4 -0.398933834892609 0.398933834892609 -0.399623807897505 -0.142527021880265 0.333642786552058 2.07904993778758e-05 2.07904993778758e-05 2.00558536092901e-05 0.143062262458287 0.000444945302259108 blue

ADCK5 0.269002575112581 -0.269002575112581 0.00669097292619467 -0.131216026114589 -0.232193466314974 0.00508277112091263 0.00508277112091263 0.945467389042781 0.177915953366677 0.0161015398250191 brown

ADCY1 -0.199283068072211 0.199283068072211 -0.269534278481291 -0.183120588486675 0.0774848854429658 0.0396019011864557 0.0396019011864557 0.00499288171111707 0.0590309098359041 0.427609232570867 yellow

ADCY10 -0.170472100482398 0.170472100482398 -0.26379005915596 -0.189538433325701 0.0583286881567415 0.0791670665179072 0.0791670665179072 0.00604331632610965 0.0505435033390974 0.550655380574729 turquoise

ADCY10P1 -0.272714373822146 0.272714373822146 0.110019995483909 0.150550543215379 0.0969442788802262 0.00448423056165962 0.00448423056165962 0.259270445508385 0.121657982469193 0.320533010934133 brown

ADCY2 -0.230392433942882 0.230392433942882 -0.358694742386825 -0.217896051430297 0.146876375764088 0.0169672021514287 0.0169672021514287 0.000148333796543585 0.0241569418356417 0.131131204668787 yellow

ADCY3 -0.329809602644599 0.329809602644599 -0.459392180821939 -0.291854411164097 0.166419665868942 0.000522202131126145 0.000522202131126145 6.44804334605067e-07 0.00228686109964265 0.0866743502587886 brown

ADCY4 -0.305288488258147 0.305288488258147 -0.265721117694022 -0.139526963958548 0.145935165898865 0.0013860803115791 0.0013860803115791 0.00567013489116196 0.151758536086227 0.1336463648775 turquoise

ADCY5 -0.336998734342603 0.336998734342603 -0.306951843533544 -0.202607792365102 0.0983066501675364 0.000386091146242514 0.000386091146242514 0.00130061092574787 0.0363550577982247 0.313747104854725 turquoise

ADCY6 -0.19550432856615 0.19550432856615 -0.247885647073073 -0.141035091152372 0.117697243089437 0.0435831499672675 0.0435831499672675 0.0100430588233057 0.147338597977119 0.227289112787417 turquoise

ADCY7 0.204430360455637 -0.204430360455637 0.431471328321072 0.463983003494594 0.16573417064679 0.0346721258015684 0.0346721258015684 3.48502100381999e-06 4.81503986154236e-07 0.0879985279534396 tan

ADCY8 0.19829135050244 -0.19829135050244 -0.114910817091773 -0.242449727665843 -0.245775495778533 0.040616112381512 0.040616112381512 0.238559796288854 0.0118653896474831 0.0107190170206355 green

ADCY9 0.562370853027303 -0.562370853027303 0.579795088912213 0.374666317313185 -0.199318606059376 2.90840419023398e-10 2.90840419023398e-10 5.992444433449e-11 7.01171586602824e-05 0.0395659539624873 brown

ADCYAP1 -0.239419929024831 0.239419929024831 -0.273160162879517 -0.0750023741564075 0.266088268117027 0.0130015858703024 0.0130015858703024 0.00441675514838306 0.442605779263373 0.00560154666066485 turquoise

ADCYAP1R1 -0.093595381143112 0.093595381143112 -0.210160131585134 -0.148261422054739 0.0511224030598398 0.337612354954183 0.337612354954183 0.0298024616706304 0.127495995206014 0.601012386414819 turquoise

ADD1 0.0461794100137952 -0.0461794100137952 -0.157493830239892 -0.280117430214243 -0.248352915765385 0.636694824113541 0.636694824113541 0.105206089022639 0.00347496028957837 0.00989853834654186 grey

ADD2 0.239601606702013 -0.239601606702013 -0.296754793067334 -0.367693293868538 -0.196382038573338 0.0129308749788613 0.0129308749788613 0.001910312965971 9.77221121569004e-05 0.0426298787217773 green

ADD3 -0.14375773000483 0.14375773000483 -0.322953816426593 -0.360351593010493 -0.146207605320619 0.139605878928277 0.139605878928277 0.00069178541821174 0.000137489454577887 0.132914581616866 yellow

ADD3-AS1 -0.408349251214771 0.408349251214771 -0.550818012749643 -0.409746619237594 0.0980959296996481 1.26370862744608e-05 1.26370862744608e-05 7.8894364287765e-10 1.17218905775458e-05 0.314790557426795 blue

ADGB 0.0527883222849855 -0.0527883222849855 -0.14465809096577 -0.177204707403407 -0.0922805738782474 0.589190278928284 0.589190278928284 0.137117654088418 0.0678548743300743 0.344472545902527 green

ADH1A 0.236965690426241 -0.236965690426241 0.109853996437532 0.0439681779919497 -0.0835944460345285 0.0139903555598918 0.0139903555598918 0.259994279155538 0.652936894175304 0.39196961687452 brown

ADH1B -0.258098993067323 0.258098993067323 -0.359285363968767 -0.244968517599958 0.101808281573198 0.00727306930934715 0.00727306930934715 0.000144380182082271 0.0109878720196732 0.296736317926839 turquoise

ADH1C -0.0127207344726021 0.0127207344726021 -0.369645695911092 -0.395635649088593 -0.138825993212813 0.896531595620008 0.896531595620008 8.91162677317823e-05 2.46644859478953e-05 0.15384646611444 turquoise

ADH4 -0.0878188547189468 0.0878188547189468 -0.17456378463981 -0.117855184105726 0.0514429724006291 0.368399692665088 0.368399692665088 0.072125139255998 0.226661665267917 0.598729291544109 yellow

ADH5 0.345929904573807 -0.345929904573807 0.342546686168684 0.167327786140678 -0.209396466877398 0.000262598660083903 0.000262598660083903 0.00030429368718576 0.0849445988468459 0.030415978908051 yellow

ADH6 -0.0266522996851985 0.0266522996851985 -0.150261220749689 -0.129828998352404 -0.00385759107913685 0.785234686735226 0.785234686735226 0.122384266471495 0.182585478944408 0.968543822410895 turquoise

ADH7 -0.0482587145313169 0.0482587145313169 -0.118477768489274 -0.0875465785495877 0.0220964273190492 0.621577032168202 0.621577032168202 0.224200207085981 0.369892105552003 0.821271298804479 turquoise

ADHFE1 0.693413629467125 -0.693413629467125 0.6035168741427 0.284389007619649 -0.386595964836176 1.25638920384988e-16 1.25638920384988e-16 5.96977434049692e-12 0.00299017976212278 3.90357096834913e-05 brown

ADI1 0.0398541161348503 -0.0398541161348503 0.0266900978213673 -0.0268956636078182 -0.0840475834983144 0.683586424175037 0.683586424175037 0.784937482374221 0.783321673659053 0.389399031298835 greenyellow

ADIG -0.127443757158881 0.127443757158881 -0.204765833584842 -0.112875230903276 0.103375228547938 0.190823904034668 0.190823904034668 0.0343696140161659 0.247035371725734 0.289324882575254 yellow

ADIPOQ -0.208528365707666 0.208528365707666 -0.33559649679825 -0.287290003015046 -0.00408233818705149 0.0311263530065725 0.0311263530065725 0.000409751149178562 0.00269655858580468 0.966712173624118 blue

ADIPOR1 0.11635801907164 -0.11635801907164 -0.217858217761008 -0.250545807237353 -0.111281520345096 0.232658482368477 0.232658482368477 0.0241821545630534 0.00924442468529989 0.253814564660251 pink

ADIPOR2 -0.064810644120224 0.064810644120224 -0.0746015257405752 -0.00180698207310544 0.104348068010544 0.507183235865933 0.507183235865933 0.44505474770257 0.985262283654746 0.284785862959485 turquoise

ADIRF -0.014202907816228 0.014202907816228 -0.129095662674552 -0.092858080001892 0.0283753403906827 0.884554759909169 0.884554759909169 0.185090199014188 0.341448601166675 0.771718527922185 green

ADIRF-AS1 0.0545121186765352 -0.0545121186765352 -0.244217360566307 -0.268139560562181 -0.103170364327442 0.577070007985282 0.577070007985282 0.0112434177891612 0.005231743700369 0.290286814715851 turquoise

ADK 0.464218121480371 -0.464218121480371 0.393489712584008 0.136671047096991 -0.334743261883893 4.74301510455107e-07 4.74301510455107e-07 2.7538036550105e-05 0.16040002560573 0.000424793607705297 red

ADM -0.808819315693534 0.808819315693534 -0.432669618557608 -0.0714448040170778 0.501787663647566 5.88027455258e-26 5.88027455258e-26 3.25142473738219e-06 0.464604639457433 3.66999981727961e-08 brown

ADM2 -0.137019326258734 0.137019326258734 -0.375024194688432 -0.371729295585684 -0.0905335820568302 0.159326988760802 0.159326988760802 6.89185737640885e-05 8.07138489218357e-05 0.353722309747006 turquoise

ADM5 0.0722534539400886 -0.0722534539400886 -0.113967395499162 -0.156334305449156 -0.101070753562154 0.459552203592362 0.459552203592362 0.242462430939761 0.107825425940293 0.300267659020294 green

ADNP 0.470212407777501 -0.470212407777501 0.557101345510706 0.261995416750233 -0.357748349668645 3.21750746731416e-07 3.21750746731416e-07 4.60671874583283e-10 0.00640952114055303 0.000154878947663933 blue

ADNP2 0.513367686065797 -0.513367686065797 0.53490950448451 0.329293323971967 -0.211649905556233 1.56430712074106e-08 1.56430712074106e-08 2.93419586408501e-09 0.00053350079414322 0.0286357483340263 blue

ADO 0.511591613051145 -0.511591613051145 0.54118578688052 0.34063224615236 -0.201454341289751 1.78657577113945e-08 1.78657577113945e-08 1.76188842948885e-09 0.000330513707017912 0.0374551895563813 turquoise

ADORA1 0.116408988072336 -0.116408988072336 -0.117845162045545 -0.0656910684231956 0.0582554370660883 0.232452521870131 0.232452521870131 0.226701443383754 0.50141874785811 0.551156782419117 green

ADORA2A-AS1 -0.203717265062124 0.203717265062124 -0.182705329421798 -0.107012835667132 0.0815553860243264 0.0353226085490085 0.0353226085490085 0.0596180042170592 0.272596999413027 0.403661909541444 turquoise

ADORA2B -0.525872459781045 0.525872459781045 -0.474276849999151 -0.239772710290622 0.276188387677891 6.00604277656347e-09 6.00604277656347e-09 2.46265679253756e-07 0.0128645864835547 0.00398190841846902 brown

ADORA3 -0.452708594118699 0.452708594118699 -0.338840561562004 -0.041304146259227 0.417813031333593 9.79034153128505e-07 9.79034153128505e-07 0.000356924731818383 0.672721805531373 7.54480629475951e-06 purple

ADPGK 0.126332430806559 -0.126332430806559 0.196056772238315 0.255767114132506 0.151527769379857 0.194753111187267 0.194753111187267 0.0429811001382725 0.00783773050750228 0.1192294004571 yellow

ADPRH 0.206625565774365 -0.206625565774365 0.177629079757295 0.326676290910303 0.298331678409547 0.0327326368217186 0.0327326368217186 0.0671881963944073 0.000594305602455338 0.00180166299797502 grey

ADPRHL1 -0.155803974008517 0.155803974008517 -0.195695953702226 -0.0715977702972859 0.160328189147541 0.109040264918804 0.109040264918804 0.0433735276747836 0.463646578305338 0.0990128154293555 turquoise

ADPRHL2 0.547968947792266 -0.547968947792266 0.593602669194576 0.434184939270878 -0.118247649524198 1.00324535585545e-09 1.00324535585545e-09 1.60146677981105e-11 2.97718122603221e-06 0.22510780509285 blue

ADPRM 0.235273720461016 -0.235273720461016 0.257345450730651 0.113060813716937 -0.178765773908538 0.0147095428015066 0.0147095428015066 0.0074514721721297 0.246254158552968 0.0654286255706137 yellow

ADRA1A -0.0516423467918674 0.0516423467918674 -0.309530704271457 -0.305947342097207 -0.0732577056573023 0.597311305472283 0.597311305472283 0.00117754197680495 0.00135163338460197 0.453320088664148 turquoise

ADRA1B -0.333207419622834 0.333207419622834 -0.279149145396945 -0.0909328137629535 0.247691210728725 0.00045315762821024 0.00045315762821024 0.00359420391360211 0.351594984951434 0.0101037391896777 blue

ADRA1D -0.160441798256394 0.160441798256394 -0.233659949605754 -0.129314036860376 0.117095213501224 0.0987706834664491 0.0987706834664491 0.0154251992790704 0.184341733185554 0.229691966248889 yellow

ADRA2A -0.122649886304441 0.122649886304441 -0.123704699395998 -0.0556230946578887 0.0837687045113202 0.208190589546939 0.208190589546939 0.204275671449676 0.569320455325593 0.390979874444645 yellow

ADRA2B 0.0173193226581243 -0.0173193226581243 -0.199592998434557 -0.188353936286322 -0.032094606849279 0.859460085024102 0.859460085024102 0.0392893198505595 0.0520292794206867 0.742779822289623 turquoise

ADRA2C 0.0978891820618031 -0.0978891820618031 -0.175764699460193 -0.172386873397266 -0.0393208628174739 0.315816519708066 0.315816519708066 0.0701571601809725 0.0758059608309298 0.687598367116913 green

ADRB1 0.107004529500743 -0.107004529500743 0.0656914816851352 -0.0165663454206169 -0.122682760851764 0.272634437348015 0.272634437348015 0.501416050121433 0.865511545046613 0.208067772545479 grey

ADRB2 0.477440583351322 -0.477440583351322 0.485404334996614 0.246937370038787 -0.280057784977948 1.99515150781089e-07 1.99515150781089e-07 1.16336671887793e-07 0.010342053077434 0.00348220169161469 blue

ADRB3 0.109691977830869 -0.109691977830869 -0.105394841697476 -0.0716162932882073 0.0302792325672247 0.260702084540345 0.260702084540345 0.279955191604394 0.463530638448982 0.756862877184001 green

ADRBK1 -0.352059719735247 0.352059719735247 -0.0912621987320091 0.0822606271069383 0.270926271093598 0.000200208925436409 0.000200208925436409 0.349845868866998 0.399594859291426 0.00476422588035556 yellow

ADRBK2 0.135317331587061 -0.135317331587061 0.494194148101127 0.568778213780499 0.253170950307737 0.164621936453735 0.164621936453735 6.31190547511995e-08 1.64445143298384e-10 0.00851161173893434 blue

ADRM1 0.172717340416497 -0.172717340416497 0.0628348016560268 0.0193308194887862 -0.0576833165791105 0.075237659390532 0.075237659390532 0.520243671582134 0.84333504679267 0.555080528243461 red

ADSL -0.0225673944208069 0.0225673944208069 0.0219664008988435 -0.0264753796643097 -0.0765334508215152 0.817527316708704 0.817527316708704 0.82230566298169 0.786626207341319 0.433321960250654 yellow

ADSS 0.0244022562965913 -0.0244022562965913 0.0316134810325651 0.0157311319895532 -0.0188357513186239 0.802980801371617 0.802980801371617 0.746504193325968 0.872233002388831 0.847297997284536 yellow

ADSSL1 0.373411039651787 -0.373411039651787 0.0197122152562652 -0.0846549513401828 -0.171968062406225 7.44766488068081e-05 7.44766488068081e-05 0.84028468633867 0.385969418188535 0.0765311411075942 brown

ADTRP 0.348574655804731 -0.348574655804731 0.377992099707337 0.187885478524887 -0.225563034634602 0.000233752902647535 0.000233752902647535 5.9689812027159e-05 0.0526267417605681 0.0194898009645249 blue

AEBP1 0.259286475390996 -0.259286475390996 0.0118909583795014 -0.0452701921918856 -0.0939050733953598 0.00699959186797052 0.00699959186797052 0.903247014311982 0.64335301489002 0.336009170617211 yellow

AEBP2 0.261194796894035 -0.261194796894035 0.293193457748554 0.154208518030854 -0.160588601735638 0.00657914432672848 0.00657914432672848 0.00217782505803052 0.112759392428231 0.0984584955274003 yellow

AEN 0.34579598084578 -0.34579598084578 0.0449679672115353 -0.159750640819938 -0.3357040087463 0.000264143354224115 0.000264143354224115 0.645572503914682 0.100250956720229 0.000407890912359092 brown

AES 0.554623312353832 -0.554623312353832 0.590348837675514 0.2968316859938 -0.346531922630902 5.70322453681919e-10 5.70322453681919e-10 2.19791654187653e-11 0.00190488038004771 0.000255757450178405 red

AF001548.5 0.413264966415202 -0.413264966415202 0.514248030348563 0.309804839393958 -0.214956045732933 9.68595776476496e-06 9.68595776476496e-06 1.46419770247739e-08 0.00116510369802844 0.0261843887300856 black

AF007147 0.324962731589275 -0.324962731589275 0.487979066761337 0.357576764784127 -0.0961059335031051 0.000637496010846323 0.000637496010846323 9.74314019306882e-08 0.000156093922178978 0.324755407564458 black

AF067845.1 0.0850629550030885 -0.0850629550030885 -0.155128269922912 -0.163298772483816 -0.0536190325249203 0.383675793921802 0.383675793921802 0.110603537106469 0.092833488336569 0.583334978357657 turquoise

AF070581 0.186522076218239 -0.186522076218239 0.0973862029055857 -0.0095222441760653 -0.156369827782447 0.0543978374819819 0.0543978374819819 0.318321524254612 0.922453106486586 0.107744433298135 grey

AF086184 -0.269277993061883 0.269277993061883 -0.139349092124683 0.0281487292997804 0.248381902811491 0.00503603074826828 0.00503603074826828 0.152286324982512 0.773492349025204 0.00988963346906462 red

AF090939 0.676150276068534 -0.676150276068534 0.490736826805921 0.150208124516888 -0.451801151268345 1.33364048545938e-15 1.33364048545938e-15 8.04455315813122e-08 0.122517915461138 1.03544575796125e-06 brown

AF131215.4 0.615811721971825 -0.615811721971825 0.500565473994518 0.126107305334621 -0.506830852423466 1.67313826161915e-12 1.67313826161915e-12 4.00826755887886e-08 0.195556139613916 2.54137826957969e-08 brown

AF131215.8 0.249798795828403 -0.249798795828403 0.253494291958652 0.198471936582902 -0.0283522171833816 0.00946284795855865 0.00946284795855865 0.00842498939649314 0.040429831000736 0.771899473449861 brown

AF186192.1 -0.0871080305691742 0.0871080305691742 -0.206353332971786 -0.145109664089976 0.0509870701287835 0.372303667090282 0.372303667090282 0.0329680700189675 0.135882458447244 0.601977389504651 turquoise

AF198444 -0.199540994288362 0.199540994288362 -0.139665896528721 -0.0197058136284906 0.167670457873502 0.0393416241889011 0.0393416241889011 0.151347245365585 0.840335866469494 0.0842990866679283 turquoise

AF213884.2 -0.215530222172926 0.215530222172926 -0.25920434880621 -0.188751740735174 0.0530604612648084 0.0257774200046968 0.0257774200046968 0.00701820836613385 0.0515263277940907 0.587269152222833 turquoise

AF279780 0.112634678620516 -0.112634678620516 -0.107858507458452 -0.202267089766812 -0.187774506983785 0.248050523225813 0.248050523225813 0.268803486555233 0.0366771499917504 0.0527690978150677 green

AF289551 0.448081312363768 -0.448081312363768 0.443226928610088 0.265132236242145 -0.188468908956789 1.30056116045963e-06 1.30056116045963e-06 1.74414905741265e-06 0.00578170511561656 0.0518835032068144 blue

AF520793 0.150746435176753 -0.150746435176753 0.559333000439553 0.449123546567981 -0.0435675459674493 0.121168125412814 0.121168125412814 3.79514766721325e-10 1.22042068082486e-06 0.655897358411883 black

AFAP1 -0.124620227454912 0.124620227454912 0.115984609892258 0.0713116039361063 -0.0460434009734438 0.200920737878167 0.200920737878167 0.234171283017162 0.465439784053616 0.637688996025294 grey

AFAP1-AS1 -0.348815121463888 0.348815121463888 -0.334837202536739 -0.138934792350893 0.246454448326045 0.000231281107080308 0.000231281107080308 0.000423112893701268 0.153520990642752 0.0104972986759291 turquoise

AFAP1L1 -0.0678087519012205 0.0678087519012205 -0.164744445088985 -0.13849915225493 0.0022899644068595 0.487694742125143 0.487694742125143 0.0899387009241986 0.154827328646597 0.981323721992512 yellow

AFAP1L2 0.0564474846640323 -0.0564474846640323 -0.10249913170046 -0.194521819384206 -0.182354050653597 0.56360180778459 0.56360180778459 0.293453409164054 0.0446712261770841 0.0601183627783492 salmon

AFF1 -0.275539540168483 0.275539540168483 -0.116203361964729 0.111218718669778 0.355953782885673 0.00407171598429605 0.00407171598429605 0.233284216826493 0.254084293751143 0.000168031411658992 blue

AFF2 0.0244686026350185 -0.0244686026350185 -0.265762258504753 -0.287917843078811 -0.105696090672956 0.802456043409435 0.802456043409435 0.00566241231366182 0.00263653769339574 0.278575212554264 turquoise

AFF3 0.702448986343814 -0.702448986343814 0.468940245529752 0.0742240897409802 -0.549296884534593 3.41184469514156e-17 3.41184469514156e-17 3.49587895862866e-07 0.447367623588712 8.97194556588648e-10 blue

AFF4 -0.0361459477711683 0.0361459477711683 -0.120404549983864 -0.0631654545515408 0.0662242523122478 0.711661315326421 0.711661315326421 0.216701981397863 0.518046193301536 0.497944481689362 yellow

AFG3L1P 0.298045747534477 -0.298045747534477 0.503059551499215 0.383507069157076 -0.0738379617414264 0.00182093866274035 0.00182093866274035 3.34702432347358e-08 4.55271361130486e-05 0.449740717248978 blue

AFG3L2 0.671780299741231 -0.671780299741231 0.540939312943928 0.282034151872815 -0.300489546270178 2.36500111473839e-15 2.36500111473839e-15 1.79789047020921e-09 0.00324932852413353 0.00166205061042942 red

AFM 0.00413934643590907 -0.00413934643590907 -0.194235721962556 -0.146561645594961 0.031077105835402 0.966247593722823 0.966247593722823 0.0449922767480999 0.131968188439742 0.750663133967363 turquoise

AFMID -0.253852448460488 0.253852448460488 -0.412337245083039 -0.283651930812241 0.112581422431134 0.00832994817540696 0.00832994817540696 1.01878015622232e-05 0.00306922188626154 0.248275657403162 green

AFP -0.143928674676209 0.143928674676209 -0.288207525179283 -0.197367073649433 0.080207128523844 0.139130843205174 0.139130843205174 0.00260925233566622 0.0415808021721115 0.411504819561578 turquoise

AFTPH -0.239537525214172 0.239537525214172 -0.100841299572397 -0.0209275423918245 0.109697677724052 0.0129557778058859 0.0129557778058859 0.301371910613307 0.830580605462805 0.260677161328547 yellow

AGA -0.21001719606818 0.21001719606818 -0.201895539898557 -0.14639188913248 0.0423938247620448 0.0299164896037624 0.0299164896037624 0.0370311322533496 0.132421324114007 0.664601129150563 yellow

AGAP1 0.652365919230961 -0.652365919230961 0.504825614938028 0.230750346201459 -0.335476319142877 2.69154512828142e-14 2.69154512828142e-14 2.94331195814784e-08 0.0167920367248219 0.000411839777824674 brown

AGAP11 0.0103877660208318 -0.0103877660208318 -0.0872342867135153 -0.143333939829539 -0.117511376574084 0.915429491468947 0.915429491468947 0.371608406007212 0.140788848633982 0.228029061044494 pink

AGAP2 -0.0199435553734172 0.0199435553734172 -0.306183458321281 -0.369174416579664 -0.185318600744573 0.838435602207469 0.838435602207469 0.00133947919155612 9.11264468522294e-05 0.0560016366483322 green

AGAP2-AS1 -0.207010396725872 0.207010396725872 -0.344945191043608 -0.272717060224604 0.0340957793460868 0.0324022480462687 0.0324022480462687 0.00027415419929365 0.00448382120141269 0.7273533128581 turquoise

AGAP3 0.0323629198999758 -0.0323629198999758 0.0704898470376789 0.160545287829002 0.170813110164782 0.740705392638517 0.740705392638517 0.470610219482897 0.0985505247250813 0.0785598839486466 yellow

AGAP4 0.263147324771078 -0.263147324771078 0.333353524850637 0.131058894048129 -0.257676747260892 0.0061722766260336 0.0061722766260336 0.000450386231487011 0.17844050491582 0.00737256594673778 brown

AGAP6 0.324221951715882 -0.324221951715882 0.303826598711643 0.133729352576957 -0.210633770647829 0.000657043261294908 0.000657043261294908 0.00146537880085797 0.169678931352321 0.0294272410874372 brown

AGAP9 0.23407855783383 -0.23407855783383 0.327103959649788 0.196771223472486 -0.13722170225995 0.0152367172784826 0.0152367172784826 0.000583953227087687 0.0422127847912636 0.158705938620513 blue

AGBL1 -0.103677570214221 0.103677570214221 -0.220549068022728 -0.135688387990279 0.0874061570109357 0.287909119229497 0.287909119229497 0.0224440701557706 0.163456581546234 0.370663235831391 turquoise

AGBL2 0.314664944183216 -0.314664944183216 0.118602897253857 -0.0807929071032841 -0.307802548408062 0.000963548881851171 0.000963548881851171 0.223707778003302 0.408086411599933 0.00125877735414381 blue

AGBL3 0.366229887598815 -0.366229887598815 0.288305265371402 0.0615599191379716 -0.310694707981059 0.000104672862771779 0.000104672862771779 0.00260010368821764 0.528760491116614 0.00112554803480754 blue

AGBL4 -0.127643675676465 0.127643675676465 -0.198493283460515 -0.11431849277116 0.0918958531397751 0.190123219458014 0.190123219458014 0.0404078581505909 0.241004915328163 0.346496343228379 turquoise

AGBL5 -0.146381484784504 0.146381484784504 -0.546232500495717 -0.539372130410844 -0.128368323330683 0.132449135313299 0.132449135313299 1.16020621890365e-09 2.0438801446096e-09 0.187599093964482 tan

AGER 0.0493352361235458 -0.0493352361235458 0.299613289852754 0.291177442587315 0.0624853523378407 0.61381096383915 0.61381096383915 0.00171751566123013 0.00234384008438407 0.522571205172632 yellow

AGFG1 -0.789255789146176 0.789255789146176 -0.715551113193912 -0.39465645471552 0.360877273791794 5.6642736228598e-24 5.6642736228598e-24 4.71300642747796e-18 2.59389337774925e-05 0.000134205681857466 brown

AGFG2 0.159980923334926 -0.159980923334926 -0.0707747181099554 -0.14472571816727 -0.143570817327032 0.0997558293614899 0.0997558293614899 0.46881430066249 0.136932130723789 0.140126694958363 yellow

AGGF1 -0.208460258589643 0.208460258589643 -0.129633372860819 -0.0588157061198452 0.0868898764162001 0.0311826744459032 0.0311826744459032 0.183251205563908 0.547327392179603 0.373506858780576 yellow

AGK 0.689489287236348 -0.689489287236348 0.621722843299409 0.325262392709943 -0.343482698178994 2.18069174901144e-16 2.18069174901144e-16 8.89807379806883e-13 0.000629741309296187 0.000292185450996122 blue

AGL -0.202117323477679 0.202117323477679 -0.346851402734861 -0.332146442735894 -0.0639596851412404 0.036819491745553 0.036819491745553 0.000252194207234666 0.000473758061565001 0.512787234378695 yellow

AGMAT 0.758802698524077 -0.758802698524077 0.599447293845327 0.240908028925184 -0.454485950131928 2.85557251418561e-21 2.85557251418561e-21 8.98776951030122e-12 0.012432204161767 8.76882596782212e-07 blue

AGO1 0.147989838994087 -0.147989838994087 0.424282869840776 0.402748065092463 0.0722217915187268 0.128202636786127 0.128202636786127 5.25497199070183e-06 1.7023949430714e-05 0.459749457877975 black

AGO2 -0.0149630096076977 0.0149630096076977 0.166524831861195 0.0631964873056247 -0.132576655019964 0.878422586329754 0.878422586329754 0.0864726104965939 0.517840196588683 0.173421026216478 yellow

AGO3 0.44021774682356 -0.44021774682356 0.622514241560287 0.35539822580377 -0.293507808387874 2.08741692644445e-06 2.08741692644445e-06 8.16848004725386e-13 0.000172308413157691 0.00215291872177252 blue

AGO4 -0.539744158763827 0.539744158763827 -0.24613366874367 -0.042278924512995 0.282678182361049 1.98271664281194e-09 1.98271664281194e-09 0.0106015461173068 0.665455600014795 0.00317652185656652 brown

AGPAT1 0.165657261911695 -0.165657261911695 -0.0384939993683759 -0.0666304658316535 -0.0575895233431146 0.0881480910995586 0.0881480910995586 0.693836435276525 0.495306063109288 0.555725067061573 salmon

AGPAT2 -0.726455964528346 0.726455964528346 -0.634094306797576 -0.304913275848964 0.395810207379552 8.32024416301994e-19 8.32024416301994e-19 2.27114209269732e-13 0.0014060523108323 2.44435523273785e-05 blue

AGPAT3 0.508504348674462 -0.508504348674462 0.409957414381779 0.304786155923795 -0.073308455566645 2.24665668027324e-08 2.24665668027324e-08 1.15893686419768e-05 0.00141287779443717 0.45300640147098 blue

AGPAT4 0.203360609537324 -0.203360609537324 0.115117670806942 0.137693423940798 0.0677971806089869 0.0356517800527614 0.0356517800527614 0.237709982259136 0.157265336166848 0.487769184941384 turquoise

AGPAT4-IT1 0.375347959121955 -0.375347959121955 0.257839595942199 0.13232580089704 -0.146801260499476 6.78507246828713e-05 6.78507246828713e-05 0.00733405113720361 0.174243387135465 0.131330594358936 brown

AGPAT5 0.573832409903333 -0.573832409903333 0.492966996406603 0.183155077377178 -0.399129869480791 1.03969335829807e-10 1.03969335829807e-10 6.88151227358534e-08 0.0589823628453093 2.05792446871611e-05 turquoise

AGPAT6 -0.134916029149659 0.134916029149659 -0.324852184103855 -0.229057948043899 0.0792170416912469 0.165889212256919 0.165889212256919 0.000640378819867344 0.0176343157204908 0.417320558678007 turquoise

AGPAT9 -0.732216308301465 0.732216308301465 -0.441348986641746 -0.263377950569966 0.188740513294965 3.21740155311902e-19 3.21740155311902e-19 1.95148202004556e-06 0.00612572486450654 0.0515404676570134 brown

AGPS -0.327610934885001 0.327610934885001 -0.171150959144489 -0.0562553561333645 0.151010480720915 0.000571895562478404 0.000571895562478404 0.0779620181315645 0.564932127816738 0.120510248422247 yellow

AGR2 0.0350483790678228 -0.0350483790678228 -0.231874351512823 -0.275124012986658 -0.132788664198001 0.720047627184627 0.720047627184627 0.0162520725511616 0.0041301769799472 0.172728240525423 turquoise

AGR3 -0.195640420267856 0.195640420267856 -0.23485356274301 -0.144430150636029 0.0931748991897285 0.043434190152828 0.043434190152828 0.014893035485849 0.137744376062493 0.339796804570158 turquoise

AGRN 0.114001240855298 -0.114001240855298 -0.079535035652534 -0.0284706390421479 0.0662263826728033 0.242321662660136 0.242321662660136 0.4154474869057 0.770972918724772 0.497930625471643 green

AGRP -0.307931963906152 0.307931963906152 -0.218686409145597 -0.0777760558792501 0.182951195160389 0.00125252173890822 0.00125252173890822 0.0236353526149467 0.425869627596889 0.0592698248061088 turquoise

AGT -0.0539120921790114 0.0539120921790114 -0.185452613133544 -0.139479334394069 0.0304435095086943 0.581275731996923 0.581275731996923 0.0558211480517913 0.151899729773681 0.755585114127473 turquoise

AGTPBP1 -0.626896934135875 0.626896934135875 -0.603416233384455 -0.374047872718753 0.234377811860456 5.06370933918905e-13 5.06370933918905e-13 6.03090687118182e-12 7.22343451351449e-05 0.0151032036236233 brown

AGTR1 -0.111254809095856 0.111254809095856 -0.316017483791693 -0.320558738740259 -0.0887007594327743 0.253929263710797 0.253929263710797 0.000913419916869652 0.000762029774567884 0.363591165262627 turquoise

AGTR2 -0.130582387656024 0.130582387656024 -0.293457063790993 -0.174084385984538 0.127256063070922 0.180038152612327 0.180038152612327 0.0021569217691245 0.0729230862901142 0.191483447244035 turquoise

AGTRAP -0.684019696700337 0.684019696700337 -0.211452170584102 0.11446476661268 0.498600942312247 4.63572770573123e-16 4.63572770573123e-16 0.028788337044122 0.240399484676238 4.61521055487595e-08 purple

AGXT -0.182945636795604 0.182945636795604 -0.292153308801285 -0.220724381692524 0.0462713117025974 0.0592776777922417 0.0592776777922417 0.00226210388329805 0.0223346430894204 0.636023424056366 turquoise

AGXT2 -0.0878407152100221 0.0878407152100221 -0.374884823528022 -0.332576454384413 -0.0243258691245069 0.36828003032295 0.36828003032295 6.93830590937056e-05 0.000465307145430309 0.803585084469889 turquoise

AHCTF1 0.146059679030399 -0.146059679030399 0.38122374098841 0.280352740478465 -0.0733793684721187 0.133311537291208 0.133311537291208 5.09597605990983e-05 0.00344652334644572 0.452568288388793 tan

AHCY -0.0375006546657552 0.0375006546657552 -0.000349607378550073 -0.0142942488733301 -0.0237415527276898 0.701357573414889 0.701357573414889 0.997148454071953 0.883817491838582 0.808211294067674 red

AHCYL1 0.274060661822244 -0.274060661822244 0.117213521094028 0.0178667161245576 -0.138462339030792 0.00428321388108264 0.00428321388108264 0.229218369957738 0.855065923267117 0.154938099238091 blue

AHCYL2 0.387527651107447 -0.387527651107447 0.359033855254917 0.21556831955115 -0.151312784062058 3.72545783692848e-05 3.72545783692848e-05 0.000146051668840504 0.0257506088784036 0.119760438415617 blue

AHDC1 0.274967087052822 -0.274967087052822 -0.0591933725655377 -0.0887567535353492 -0.0653151708808541 0.00415244960469637 0.00415244960469637 0.544753416435401 0.363287173366329 0.503875707240121 green

AHI1 0.310570082472478 -0.310570082472478 0.184532224175531 0.0649743094003187 -0.155488665261521 0.00113101308385111 0.00113101308385111 0.0570704089941985 0.506109064228049 0.109767589176333 brown

AHNAK 0.654243429015872 -0.654243429015872 0.652383948109111 0.444731807143019 -0.184993364999027 2.14398930213064e-14 2.14398930213064e-14 2.68569341359654e-14 1.59324537732709e-06 0.0564416584583328 brown

AHNAK2 0.00357623803788011 -0.00357623803788011 -0.281474701898974 -0.286732426804626 -0.0810623495744451 0.970837028878413 0.970837028878413 0.00331378591322891 0.0027508910081953 0.40651965110497 green

AHR -0.0460967703340964 0.0460967703340964 0.278009851473223 0.423035821471146 0.317239640589378 0.63729881054162 0.63729881054162 0.00373919499422075 5.63766756801568e-06 0.000870184041881226 turquoise

AHRR -0.160980885126846 0.160980885126846 -0.135850179449128 -0.0534583221008908 0.104927790923153 0.0976280870049331 0.0976280870049331 0.162950375990425 0.584465665603188 0.282103723199991 turquoise

AHSA1 0.46535278756378 -0.46535278756378 0.239289414877184 0.049939056589693 -0.259831071055245 4.40958861677085e-07 4.40958861677085e-07 0.0130525912155843 0.609473526477435 0.00687724479219445 turquoise

AHSA2 0.384735857968262 -0.384735857968262 0.534080941260437 0.367150593239718 -0.146245998536502 4.28325015397686e-05 4.28325015397686e-05 3.13613271544554e-09 0.000100248085486255 0.132811702169835 yellow

AHSG -0.048499433168824 0.048499433168824 -0.164744688028996 -0.159089723561477 -0.0326340278939129 0.619836828101727 0.619836828101727 0.0899382205553081 0.101682714627107 0.738611239842159 green

AHSP -0.28962023584366 0.28962023584366 -0.435023893793067 -0.295466790829595 0.12520657550973 0.00247979953890507 0.00247979953890507 2.83490538669454e-06 0.00200342121108072 0.19879300091522 pink

AICDA -0.200067189761704 0.200067189761704 -0.249735756407599 -0.155374856321437 0.0960389717665806 0.0388150703718951 0.0388150703718951 0.00948148634436328 0.110031039061228 0.325094197857965 turquoise

AIDA 0.266412214348884 -0.266412214348884 0.0169615262767638 -0.0560558749945269 -0.119499757969713 0.0055416431970002 0.0055416431970002 0.862334598773849 0.566314931037182 0.220200599181449 turquoise

AIF1 -0.245720037608881 0.245720037608881 0.129809578940386 0.38336591463821 0.463336383545804 0.0107373073886015 0.0107373073886015 0.182651485381045 4.58466236854753e-05 5.0185211774242e-07 brown

AIF1L -0.49734626090054 0.49734626090054 -0.354155761358396 -0.0723763885731877 0.387161590951309 5.04776620056079e-08 5.04776620056079e-08 0.000182241371050572 0.45878677384708 3.79451190729714e-05 turquoise

AIFM1 0.310077402086403 -0.310077402086403 0.0801574450703709 -0.0811842399238933 -0.253111654543633 0.00115285551479404 0.00115285551479404 0.411795524020882 0.405812044225246 0.00852758179684658 red

AIFM2 -0.349761844425086 0.349761844425086 -0.379114531396115 -0.266182818308116 0.0943765436053804 0.000221782635997417 0.000221782635997417 5.65106148740592e-05 0.00558400341133528 0.333577798896641 blue

AIFM3 0.0988302584551523 -0.0988302584551523 0.0653521040417312 0.117715843441955 0.105566168790507 0.311164007220119 0.311164007220119 0.503634025852117 0.227215156392052 0.279169805881804 yellow

AIG1 -0.515969991190711 0.515969991190711 -0.6719130444438 -0.501123746006826 0.117463600527035 1.28582740453718e-08 1.28582740453718e-08 2.32453461957306e-15 3.85021410656979e-08 0.228219533967887 brown

AIM1 -0.167712659932008 0.167712659932008 -0.154743158190979 -0.078569375317194 0.08953867138791 0.0842198593433102 0.0842198593433102 0.111502283499411 0.4211506277381 0.359058534873731 brown

AIM1L -0.169132301240416 0.169132301240416 -0.147576507970458 -0.164410927898821 -0.0663786069494842 0.0815891489208421 0.0815891489208421 0.129283851553463 0.0906000913103007 0.496941057322977 turquoise

AIM2 -0.71208206365034 0.71208206365034 -0.311862348100159 0.0363101048226009 0.510613142422071 8.04577370911494e-18 8.04577370911494e-18 0.00107550151585797 0.710409944599069 1.92162457793979e-08 brown

AIMP1 0.292787055079678 -0.292787055079678 0.338097322436397 0.201687858397915 -0.144710975515825 0.00221041050163186 0.00221041050163186 0.000368440898222745 0.0372302403554708 0.136972558324026 yellow

AIMP2 0.0759092092377001 -0.0759092092377001 -0.0734685765500435 -0.0673472618504024 -0.00844811576008521 0.437093629903978 0.437093629903978 0.452017479564769 0.490668375095454 0.931177776184676 red

AIP 0.504137957800755 -0.504137957800755 0.59535716272232 0.371951870457926 -0.226329282250903 3.0946366863018e-08 3.0946366863018e-08 1.34815726906448e-11 7.98613088567546e-05 0.0190692030018985 blue

AIPL1 -0.0918757199606039 0.0918757199606039 -0.221216200177304 -0.143011802990714 0.0759452044689726 0.346602457517626 0.346602457517626 0.0220300976895432 0.141693132729964 0.436875641726879 turquoise

AIRE -0.106812429828609 0.106812429828609 -0.0774445324321305 -0.0283677796728074 0.0633908824218731 0.273501245254689 0.273501245254689 0.427850644015599 0.771777691296076 0.516550744650101 green

AJAP1 -0.0699146529741957 0.0699146529741957 -0.206721170190648 -0.152293463026748 0.0393320484458317 0.474247810208703 0.474247810208703 0.0326502933019786 0.117352815283943 0.687514122248166 yellow

AJUBA 0.18578488868035 -0.18578488868035 -0.117568618952114 -0.178850611533559 -0.134076226323587 0.0553756968810924 0.0553756968810924 0.227800994903807 0.0652988154569605 0.168564613242621 green

AK000798 0.495154450232884 -0.495154450232884 0.323454770621151 0.0819050569037383 -0.326795690992773 5.8978529884379e-08 5.8978529884379e-08 0.0006778648840209 0.401642349844041 0.000591398529146546 brown

AK021537 0.285029317582184 -0.285029317582184 0.109834782383454 -0.0180976703512623 -0.188838638424764 0.00292300549650811 0.00292300549650811 0.260078150375114 0.853213274746551 0.051416996845302 yellow

AK021804 -0.0535050579424906 0.0535050579424906 -0.301162183559943 -0.237432264705269 0.0309036983125191 0.584136749342872 0.584136749342872 0.00162058169551639 0.0137974961565766 0.752009235058664 turquoise

AK021933 -0.32071460472702 0.32071460472702 -0.228210190423744 -0.128541948178747 0.11055811303787 0.000757267072664129 0.000757267072664129 0.018069781189923 0.186997953513727 0.256933465120306 turquoise

AK021977 -0.0198786743079329 0.0198786743079329 -0.309397650481653 -0.295780272644271 -0.0562045905281757 0.838954103421441 0.838954103421441 0.00118362248406234 0.00198038963909791 0.565283883123611 turquoise

AK024936 0.245136491611002 -0.245136491611002 0.170782343232148 0.0443323413857364 -0.170702761501612 0.0109314276719798 0.0109314276719798 0.0786145120563397 0.650250578238729 0.0787559544151207 brown

AK025288 -0.0773173607948167 0.0773173607948167 -0.145247804768416 -0.0484043962977704 0.127030866610325 0.42861195834993 0.42861195834993 0.135506296528104 0.620523619009117 0.192276950429011 grey

AK026905 0.344449500524896 -0.344449500524896 0.457322709125222 0.301600983603843 -0.146908504209743 0.000280147158303644 0.000280147158303644 7.34510996068675e-07 0.00159403733306264 0.131045992001584 black

AK054988 -0.0511769212308729 0.0511769212308729 -0.269283568916541 -0.265827083757422 -0.0631572286918574 0.600623834588656 0.600623834588656 0.00503508845475489 0.0056502628830638 0.518100803902906 green

AK055458 -0.0374864720026586 0.0374864720026586 0.0335296418921418 0.149488034924866 0.205274677267692 0.701465169069835 0.701465169069835 0.731706789291142 0.124341577075697 0.0339150103369648 turquoise

AK055981 0.723274306797857 -0.723274306797857 0.568267091786487 0.290049161340929 -0.326242187478525 1.39183290963454e-18 1.39183290963454e-18 1.72175013419981e-10 0.00244165774196829 0.000604985683638932 brown

AK056098 -0.201902725211008 0.201902725211008 -0.250431876645825 -0.119225100386266 0.158355974688209 0.0370242595865983 0.0370242595865983 0.00927744827305894 0.221270493913433 0.103290992271383 turquoise

AK056982 -0.204351034766542 0.204351034766542 0.129402875575701 0.338453755665558 0.387745001726803 0.0347439842266531 0.0347439842266531 0.184037876022968 0.000362876058819496 3.685014688633e-05 purple

AK074476 0.316801568565778 -0.316801568565778 0.284330016935887 0.143693751027382 -0.165661232804495 0.000885461782118898 0.000885461782118898 0.00299643770970465 0.139783985270379 0.0881403640273644 brown

AK090844 -0.633029624420329 0.633029624420329 -0.215031012560437 0.0529478040342602 0.399412951621821 2.56052200404151e-13 2.56052200404151e-13 0.0261309445090363 0.588064093329298 2.02777359654922e-05 brown

AK091729 -0.225700572065342 0.225700572065342 -0.0416588511419885 -0.0351539811886555 0.000355574156260172 0.0194137228827709 0.0194137228827709 0.670074227770785 0.719239277764883 0.997099786713972 grey

AK093205 -0.233588170817807 0.233588170817807 -0.292699536247627 -0.258086433145097 -0.0163131691232059 0.015457720627368 0.015457720627368 0.00221748514813445 0.00727601166556174 0.86754801495648 grey

AK094644 -0.403902958082652 0.403902958082652 -0.377624543725658 -0.178833094339664 0.240387842259073 1.60164703158343e-05 1.60164703158343e-05 6.07666451836119e-05 0.0653256013655512 0.0126287152493662 turquoise

AK096159 -0.00544131611627559 0.00544131611627559 0.00115463937076418 0.0568512267322072 0.0947647192377805 0.955640774616116 0.955640774616116 0.990582467779768 0.560811110675867 0.331584396774252 grey

AK097370 -0.24967787019632 0.24967787019632 -0.273232813771005 -0.0895967772991449 0.241438824195514 0.00949862953204044 0.00949862953204044 0.00440584494503978 0.358745518116334 0.0122344420226324 turquoise

AK097453 0.244233544785461 -0.244233544785461 0.450794433977521 0.343253248996401 -0.0668612219412838 0.011237857509736 0.011237857509736 1.10163220774091e-06 0.000295111736835765 0.493810551486884 black

AK098263 -0.32457414270177 0.32457414270177 -0.025621425086675 0.0638878170252747 0.145252419373528 0.000647682368368108 0.000647682368368108 0.793352150162159 0.513261974627591 0.135493744494249 brown

AK1 0.379161929904203 -0.379161929904203 0.150992618634953 -0.0243354406315905 -0.258678696711216 5.63799059300403e-05 5.63799059300403e-05 0.120554665232904 0.803509359910235 0.0071384047709837 brown

AK130486 0.213627982274825 -0.213627982274825 -0.0966955552929752 -0.250113318037333 -0.285000724918604 0.0271467199867465 0.0271467199867465 0.321782021703689 0.00937033597369255 0.0029259759250512 green

AK131021 -0.175159585805202 0.175159585805202 -0.197544825160378 -0.110204182601202 0.0975086495046842 0.0711432816690951 0.0711432816690951 0.0413938079858414 0.258468914883054 0.317710521124761 turquoise

AK2 0.112033681573157 -0.112033681573157 0.261049455141392 0.175778009538811 -0.0777222328433151 0.250599344583899 0.250599344583899 0.00661036026639921 0.0701355943525679 0.426190887622219 yellow

AK3 0.222720268500333 -0.222720268500333 0.148672222000073 0.000575080666654861 -0.213086211819489 0.0211207391246076 0.0211207391246076 0.126432800303937 0.995309415176423 0.0275478459474957 yellow

AK5 0.730488079501006 -0.730488079501006 0.421058111987952 0.114925450483415 -0.411320229878765 4.28958260683347e-19 4.28958260683347e-19 6.29894328441409e-06 0.238499608772088 1.07660219346276e-05 brown

AK6 -0.0714578788174789 0.0714578788174789 -0.0538047234174984 -0.0895591181747499 -0.0744348261783312 0.464522706726409 0.464522706726409 0.582029792469236 0.358948368669439 0.44607543009046 yellow

AK7 -0.244070530117901 0.244070530117901 -0.3819378097395 -0.233219885504938 0.154351027662966 0.0112939732380947 0.0112939732380947 4.91989574159282e-05 0.0156255194810715 0.11242323568562 turquoise

AK8 0.134085346395137 -0.134085346395137 -0.0731394944896291 -0.0653927313915143 -0.00560677932078843 0.168535388584853 0.168535388584853 0.45405122486148 0.503368240302433 0.954293303216233 turquoise

AK9 -0.466220939872263 0.466220939872263 -0.473337443730651 -0.302106027026099 0.169110286070697 4.16965421342509e-07 4.16965421342509e-07 2.62044429583269e-07 0.00156397441503689 0.0816294371809656 brown

AKAP1 0.700165270208091 -0.700165270208091 0.683954650175814 0.292768442322116 -0.488199476141806 4.76510734597064e-17 4.76510734597064e-17 4.67702920424428e-16 0.00221191336790064 9.59569518159645e-08 blue

AKAP10 -0.31430406478425 0.31430406478425 0.00570596244848599 0.149608313333889 0.245539833581946 0.00097734198455115 0.00097734198455115 0.953485653830859 0.124035522810018 0.0107969284776977 brown

AKAP11 0.673385159218187 -0.673385159218187 0.731440660806514 0.421135638428546 -0.33884321694018 1.9184683097596e-15 1.9184683097596e-15 3.66172207419762e-19 6.27169908175031e-06 0.000356884189357584 blue

AKAP12 0.554559124900793 -0.554559124900793 0.480735185803652 0.272709588366254 -0.229621974079677 5.73472109227534e-10 5.73472109227534e-10 1.59875784348602e-07 0.0044849598632228 0.0173496422840278 brown

AKAP13 0.0805984565653729 -0.0805984565653729 0.200396311392689 0.261031255732048 0.154208314196861 0.409219306713469 0.409219306713469 0.0384887342086473 0.0066142782975419 0.112759873797657 black

AKAP14 0.0329574078397478 -0.0329574078397478 -0.147587839133605 -0.231121958648618 -0.179512992284297 0.736115814836657 0.736115814836657 0.129254118090062 0.0166118240739081 0.0642924882121015 yellow

AKAP17A 0.110809197010632 -0.110809197010632 0.297464353832316 0.186110523623296 -0.112627896210811 0.255847983547012 0.255847983547012 0.00186070968468535 0.0549419851327583 0.248079187225822 tan

AKAP3 -0.377215744899166 0.377215744899166 -0.374927825746733 -0.169782105470994 0.251856748957459 6.19855871606759e-05 6.19855871606759e-05 6.92394342179831e-05 0.0804071370982513 0.00887181339039999 turquoise

AKAP4 0.127319876667052 -0.127319876667052 -0.0288894884579907 -0.0470114421352669 -0.0381418495942379 0.191259025765499 0.191259025765499 0.767698354337737 0.630627082932464 0.696499393516211 green

AKAP5 0.471232921214919 -0.471232921214919 0.186841540421547 -0.0106612842918412 -0.287101582704942 3.00957904586071e-07 3.00957904586071e-07 0.0539785092261242 0.913211247256389 0.00271480991869684 brown

AKAP6 -0.19290374124772 0.19290374124772 -0.312442290722643 -0.226677528779402 0.0653865058180296 0.0465123393368926 0.0465123393368926 0.00105140613057843 0.0188806482354026 0.503408963485364 turquoise

AKAP7 0.642117153981752 -0.642117153981752 0.528704256563858 0.354072451661618 -0.160686781498227 9.06156041458155e-14 9.06156041458155e-14 4.8093300160302e-09 0.000182926017133605 0.0982501430963127 blue

AKAP8 0.445977550088762 -0.445977550088762 0.53648524061087 0.380847019531063 -0.126476792215778 1.47776992193742e-06 1.47776992193742e-06 2.58405662305565e-09 5.19122573347445e-05 0.194239426534176 blue

AKAP8L 0.143207789630489 -0.143207789630489 0.0694063058831878 -0.0462441142919806 -0.178368863493822 0.141142447853628 0.141142447853628 0.477475285584319 0.636222087695959 0.0660387269868304 yellow

AKAP9 0.556811659727182 -0.556811659727182 0.363860473613621 0.153128643260225 -0.264168186758838 4.72357838360179e-10 4.72357838360179e-10 0.000116908998425206 0.115332074062711 0.00596857036967898 blue

AKIP1 -0.180191109981155 0.180191109981155 -0.0470756266704174 0.111178025646001 0.256353122328187 0.0632753610407148 0.0632753610407148 0.630160017417676 0.254259172567384 0.00769230374429611 red

AKIRIN1 -0.27765547175626 0.27765547175626 -0.195015442964758 -0.124627791826459 0.069402465354686 0.00378535124649255 0.00378535124649255 0.0441217744078234 0.200893184452337 0.477499713940492 yellow

AKIRIN2 -0.471576457683795 0.471576457683795 -0.210493118868181 0.0414692035817961 0.373409954527299 2.94250775423918e-07 2.94250775423918e-07 0.029538246117065 0.67148928955279 7.44805243210987e-05 brown

AKNA 0.466427194975482 -0.466427194975482 0.677145838154921 0.497189262887508 -0.131671323123437 4.11450382992577e-07 4.11450382992577e-07 1.16886160464488e-15 5.10454178252965e-08 0.176402432203863 black

AKNAD1 -0.225406462438118 0.225406462438118 -0.239883858476627 -0.197331940360429 0.0106892035019185 0.0195767203443636 0.0195767203443636 0.0128216846974097 0.041617845624665 0.912984858525342 turquoise

AKR1A1 0.178733249260676 -0.178733249260676 0.327945499714103 0.348451866551646 0.1188369707567 0.0654784471375625 0.0654784471375625 0.000564064029003488 0.000235024479889374 0.222788658146335 red

AKR1B1 0.597638484951858 -0.597638484951858 0.576814491096004 0.249911762178371 -0.406627222482429 1.07604768405333e-11 1.07604768405333e-11 7.90362790607066e-11 0.00942952861593601 1.38573898626699e-05 blue

AKR1B10 -0.190996492234986 0.190996492234986 -0.0997145431688343 0.023056990898522 0.182678962790762 0.048762871611005 0.048762871611005 0.30683308149244 0.81363958526088 0.0596554420471692 turquoise

AKR1C1 -0.232525444808078 0.232525444808078 -0.52012062511842 -0.367848790295751 0.124961407092077 0.0159462224801841 0.0159462224801841 9.37346106479157e-09 9.70093526881797e-05 0.199680683420988 blue

AKR1C3 0.326353837436935 -0.326353837436935 0.120979875879753 0.0605563520843317 -0.0714780043671613 0.000602222114486621 0.000602222114486621 0.214497941207929 0.535513648732484 0.464396606235139 turquoise

AKR1C4 -0.117712579507793 0.117712579507793 -0.217081943863195 -0.231146678286414 -0.079495750687442 0.227228132815406 0.227228132815406 0.0247044565885426 0.0165998959434484 0.415678620781776 grey

AKR1C6P -0.223276517120869 0.223276517120869 -0.213905159293124 -0.112714305988178 0.116807141229995 0.0207926936004925 0.0207926936004925 0.0269434270114365 0.247714170757244 0.230848014269437 blue

AKR1D1 -0.156928150568996 0.156928150568996 -0.270166584957963 -0.198822798740068 0.0517622885710322 0.106477658211588 0.106477658211588 0.00488783059155849 0.0400699442806318 0.596458983969085 turquoise

AKR1E2 0.266354886140677 -0.266354886140677 -0.0209751023152258 -0.0743985982969701 -0.095989379758337 0.00555220253425058 0.00555220253425058 0.830201358707489 0.446297422942533 0.32534525272759 green

AKR7A2 0.264768570212713 -0.264768570212713 0.262948276724895 0.186043397229011 -0.0630447523389669 0.00585157619797554 0.00585157619797554 0.00621270656612744 0.0550311613303499 0.518847816606339 red

AKR7A3 0.123185950529921 -0.123185950529921 0.0962501091106834 0.117216966389231 0.0602323215620252 0.206194349696456 0.206194349696456 0.324026725850722 0.229204588392378 0.53770321097095 brown

AKT1 0.586294791950736 -0.586294791950736 0.687350679504363 0.513704889798085 -0.118351800548496 3.2448184970601e-11 3.2448184970601e-11 2.93433866305822e-16 1.525211106112e-08 0.224696709562123 blue

AKT1S1 -0.0034006634396849 0.0034006634396849 -0.244352906804369 -0.260044869303389 -0.0892455395659606 0.97226819529294 0.97226819529294 0.0111969235573863 0.00682973362689344 0.360640215605182 pink

AKT2 0.701944077906969 -0.701944077906969 0.416583645051444 0.148507180671899 -0.347918759536329 3.67437911441176e-17 3.67437911441176e-17 8.07478223050913e-06 0.12685912456248 0.00024061973854252 brown

AKT3 0.755320025319656 -0.755320025319656 0.573343176372629 0.181072177839102 -0.518390167524292 5.48654894604631e-21 5.48654894604631e-21 1.08724444089797e-10 0.0619734407420259 1.06993800155229e-08 blue

AKTIP -0.0916926902175857 0.0916926902175857 0.0248093494585496 0.0048876376697571 -0.0274309747523217 0.347568072548474 0.347568072548474 0.799762337685972 0.960150615150134 0.779118303784982 green

AL022341.3 0.155035711486478 -0.155035711486478 -0.135079940053173 -0.131688863372768 -0.0288704966112958 0.110819026939981 0.110819026939981 0.165370725843175 0.176344313849007 0.767846744638242 green

AL022344.5 -0.300019621007999 0.300019621007999 -0.199236500873599 -0.0637384463179282 0.178756408393593 0.00169159051108025 0.00169159051108025 0.039649045979914 0.514249396048268 0.06544296859669 turquoise

AL109706 -0.286848737402979 0.286848737402979 -0.40172335790249 -0.306716753372466 0.0581783182743495 0.00273947656395353 0.00273947656395353 1.79674009900022e-05 0.00131239246637433 0.551684897581123 brown

AL132709.8 -0.134500197996316 0.134500197996316 -0.271519493962666 -0.180909170707187 0.0840941158437306 0.167209984013332 0.167209984013332 0.00466965233338113 0.0622126489907469 0.389135633381701 turquoise

AL133493.2 -0.188289500634982 0.188289500634982 -0.329070777131376 -0.198009708937716 0.137952975953082 0.0521111262543209 0.0521111262543209 0.000538440079732216 0.0409080673057381 0.156476861496608 blue

AL590762.11 0.217160822361002 -0.217160822361002 0.0774031534094022 0.0790427335744806 0.0226200976887832 0.0246509486725608 0.0246509486725608 0.428098274761218 0.418349359653705 0.817108602309455 grey

AL832163 -0.00501994543214396 0.00501994543214396 -0.0781997205448425 0.0196270453309471 0.145883803728578 0.959072817802064 0.959072817802064 0.423345717151008 0.840965662529619 0.133784669291376 turquoise

AL833181 0.800869704227931 -0.800869704227931 0.662853162803405 0.291317648818881 -0.460277820494088 3.99980780745328e-25 3.99980780745328e-25 7.3992933490575e-15 0.00233193510319647 6.0968372608246e-07 blue

AL928742.12 -0.0092315266333373 0.0092315266333373 -0.144662659018006 -0.126860677356403 -0.00688384646012539 0.924813612557151 0.924813612557151 0.137105116423474 0.192878208806649 0.943897955783098 turquoise

ALAD -0.0723294449083917 0.0723294449083917 -0.520487217339586 -0.484877122011579 -0.0730062548797138 0.459078976733079 0.459078976733079 9.11352260042481e-09 1.20617213318059e-07 0.45487609703675 magenta

ALAS1 -0.529753311731838 0.529753311731838 -0.426128382762141 -0.200187668026476 0.274004548665156 4.42698985052458e-09 4.42698985052458e-09 4.73333349124444e-06 0.0386953439403974 0.00429142864053252 brown

ALAS2 -0.126511390996396 0.126511390996396 -0.437393039014379 -0.367670543481895 0.00615065874747139 0.19411645859634 0.19411645859634 2.46703764263415e-06 9.78268023157251e-05 0.949865076981128 pink

ALB -0.101290808877148 0.101290808877148 -0.326509280295491 -0.233267430685292 0.0744631440451946 0.299211139135459 0.299211139135459 0.000598393860514936 0.0156037684428399 0.445901950440538 turquoise

ALCAM -0.580025873071389 0.580025873071389 -0.509662965151488 -0.213511150396155 0.371681173979168 5.86468528596881e-11 5.86468528596881e-11 2.06207956340442e-08 0.0272327993527275 8.08992828115082e-05 brown

ALDH16A1 0.571272480701306 -0.571272480701306 0.500966161029864 0.281173126942046 -0.244395705853828 1.3127393470744e-10 1.3127393470744e-10 3.89421445588248e-08 0.00334900611526909 0.0111822778931911 blue

ALDH18A1 0.640395826164939 -0.640395826164939 0.467547596824237 0.111007158647294 -0.484902799842695 1.10613417328211e-13 1.10613417328211e-13 3.82683377302271e-07 0.254994376704387 1.20405296824107e-07 red

ALDH1A1 0.590822424022302 -0.590822424022302 0.667487795430272 0.514645026492611 -0.0881582024755908 2.09939732766953e-11 2.09939732766953e-11 4.11283815552696e-15 1.42108671284699e-08 0.366544822909826 blue

ALDH1A2 -0.026947241055404 0.026947241055404 -0.269360227666979 -0.257478280470796 -0.0488861396774934 0.782916403663433 0.782916403663433 0.00502214940079572 0.00741974637379799 0.617045640052311 green

ALDH1A3 -0.0852689517008058 0.0852689517008058 -0.191015083212761 -0.122596305601245 0.0670883579251372 0.382520898730353 0.382520898730353 0.0487405072660448 0.208390874303021 0.49234083110203 turquoise

ALDH1B1 -0.00715123918542561 0.00715123918542561 -0.207147684916112 -0.19388560881152 -0.0305996644714101 0.941722507927342 0.941722507927342 0.0322850655489546 0.0453877709860714 0.754371135214753 pink

ALDH1L1 -0.175978447025005 0.175978447025005 -0.258563330095509 -0.152165208348322 0.114193087193064 0.0698114807482235 0.0698114807482235 0.00716502712281056 0.117665542407996 0.241524813712357 turquoise

ALDH1L1-AS2 -0.210594963939913 0.210594963939913 -0.248212323298722 -0.162362227033814 0.0819939679247213 0.0294578326836484 0.0294578326836484 0.00994182858761072 0.0947478227705491 0.401129789877178 turquoise

ALDH1L2 -0.412070180903219 0.412070180903219 -0.443277738741113 -0.302510041616758 0.125144428317997 1.03367428608804e-05 1.03367428608804e-05 1.7388403859945e-06 0.00154029673994667 0.199017748077645 blue

ALDH2 0.0330716430994569 -0.0330716430994569 0.284681842145118 0.342290123943945 0.170677823404774 0.7352349498507 0.7352349498507 0.00295928842333191 0.000307692512200387 0.0788003194126263 black

ALDH3A1 0.00914220706661148 -0.00914220706661148 -0.178502279133114 -0.102714650519118 0.082794016223056 0.925538982463871 0.925538982463871 0.0658331368618549 0.292434201504059 0.396535059535171 green

ALDH3A2 0.487875933490044 -0.487875933490044 0.476879064824057 0.270369275077928 -0.228039312993576 9.81287206254202e-08 9.81287206254202e-08 2.07143021085008e-07 0.00485457533303365 0.0181586705951268 blue

ALDH3B1 -0.381988007927423 0.381988007927423 -0.470396247352604 -0.177902675083673 0.375540760803336 4.90773383815994e-05 4.90773383815994e-05 3.1790681695993e-07 0.0667612094717934 6.72221714298419e-05 blue

ALDH3B2 -0.273534459141044 0.273534459141044 -0.334144471721113 -0.239721685306046 0.0745091894833441 0.00436080298695704 0.00436080298695704 0.000435651903288211 0.0128843233677739 0.445619949820288 turquoise

ALDH4A1 -0.200571184127802 0.200571184127802 -0.580140428999894 -0.433527512608364 0.0999795006355905 0.0383162785610522 0.0383162785610522 5.80224755333954e-11 3.09336242993311e-06 0.305543110160978 tan

ALDH5A1 0.309861235102343 -0.309861235102343 0.0951549497165016 -0.0312353909286894 -0.189985598564711 0.00116255975544739 0.00116255975544739 0.329588109556532 0.749435075565492 0.0499917896184696 purple

ALDH6A1 0.452356530538566 -0.452356530538566 0.10387314394043 -0.16674134765983 -0.432374205567294 1.00056513467014e-06 1.00056513467014e-06 0.286995771142279 0.0860584454908492 3.30759789158861e-06 turquoise

ALDH7A1 0.0746391859587004 -0.0746391859587004 -0.264604960126303 -0.28839166865915 -0.108166089246435 0.44482434038315 0.44482434038315 0.0058832545065905 0.00259204027533355 0.267432639873729 green

ALDH8A1 0.20347069241293 -0.20347069241293 0.320770798878095 0.202285308049111 -0.118750452578416 0.0355499061581878 0.0355499061581878 0.000755556674116781 0.036659866427866 0.223128072096361 turquoise

ALDH9A1 0.750372783080187 -0.750372783080187 0.762737996921317 0.511347185066317 -0.230895131773605 1.3618183706712e-20 1.3618183706712e-20 1.34710809398115e-21 1.81943445304288e-08 0.0167216231345405 black

ALDOA -0.514533012052325 0.514533012052325 -0.473029755940618 -0.252501956668426 0.252802316970142 1.43312604649263e-08 1.43312604649263e-08 2.67418454635112e-07 0.00869332885570013 0.0086113242513866 brown

ALDOAP2 0.00124822084901297 -0.00124822084901297 -0.174862682306814 -0.195001202857168 -0.0789765058844414 0.989819232753156 0.989819232753156 0.0716312087728431 0.0441375459243164 0.418740634061381 turquoise

ALDOB -0.324997447693879 0.324997447693879 -0.439625508593654 -0.309184436682078 0.108565183188056 0.000636593157173562 0.000636593157173562 2.16210999848972e-06 0.00119342591432922 0.265661009236414 yellow

ALDOC 0.53621059192645 -0.53621059192645 0.408470621325723 0.129857801715881 -0.36786929374271 2.64205266266199e-09 2.64205266266199e-09 1.2555006610457e-05 0.182487608629491 9.69157314691435e-05 brown

ALG1 0.475620291882694 -0.475620291882694 0.275424642854911 0.0484596622825455 -0.314368595729002 2.25264329216966e-07 2.25264329216966e-07 0.00408780651971029 0.620124195594081 0.000974862406130981 brown

ALG10 -0.263978084046745 0.263978084046745 -0.274653306378114 -0.165811667513784 0.114213569307103 0.00600604546051607 0.00600604546051607 0.00419730710065362 0.0878480251648799 0.241439847160498 yellow

ALG12 0.414008081126533 -0.414008081126533 0.329614054521626 0.160148526751605 -0.202952724144302 9.30085945577048e-06 9.30085945577048e-06 0.000526455551014212 0.0993966777611646 0.0360313911343934 blue

ALG13 0.0747932108398884 -0.0747932108398884 0.179340049509698 0.14907215934885 -0.00537183706111616 0.443882705827986 0.443882705827986 0.0645540077158839 0.125404268594355 0.956206623925997 yellow

ALG14 -0.504500408222995 0.504500408222995 -0.463999686496926 -0.338433894278795 0.0940487504321526 3.01397287815744e-08 3.01397287815744e-08 4.80989520997619e-07 0.000363184096880596 0.335267040171276 magenta

ALG2 0.153180877437503 -0.153180877437503 0.119988258489676 -0.0271056790419852 -0.218736585345552 0.115206593844509 0.115206593844509 0.218306754334055 0.781671840089248 0.0236025669441094 yellow

ALG3 0.356504041662205 -0.356504041662205 0.122792482758462 -0.0321627124515474 -0.231351580425027 0.000163892400943416 0.000163892400943416 0.207658234035248 0.74225309707826 0.016501308237636 brown

ALG5 -0.191700576677265 0.191700576677265 -0.155856747685765 -0.174205647896423 -0.0710696784729797 0.0479217880866357 0.0479217880866357 0.10891889945179 0.0727205792583037 0.466958721433798 yellow

ALG6 -0.223728179246948 0.223728179246948 -0.142362548480643 -0.100119457251147 0.0351610232412451 0.020529557024515 0.020529557024515 0.143529037878131 0.304863150695885 0.71918538416945 green

ALG8 0.444040333931653 -0.444040333931653 0.358914666736035 0.130154850865616 -0.296013646857987 1.66097957586926e-06 1.66097957586926e-06 0.000146850032117687 0.181480507562251 0.00196339941566391 red

ALG9 0.419841491417664 -0.419841491417664 0.182020727625836 -0.0899363836226735 -0.414621515443777 6.74137684505445e-06 6.74137684505445e-06 0.0605963133648498 0.356919441810207 8.99387388253862e-06 red

ALK -0.201818532331006 0.201818532331006 -0.213163954295398 -0.0734208641305341 0.182386800118422 0.0371048563352778 0.0371048563352778 0.0274899777127134 0.452312029972467 0.060071570128502 turquoise

ALKBH1 0.428748409252403 -0.428748409252403 0.291684258622275 0.0959041900882163 -0.257307432461793 4.07613999330182e-06 4.07613999330182e-06 0.00230106357547875 0.325776807494575 0.00746057471225131 blue

ALKBH2 0.392448190318597 -0.392448190318597 0.265255103289462 0.0256348060071131 -0.338440264504369 2.90431060085626e-05 2.90431060085626e-05 0.00575826681443464 0.793246640617357 0.000363085272307205 blue

ALKBH3 0.500923290187781 -0.500923290187781 0.578842466916727 0.351041687514744 -0.238017486933355 3.90626759936824e-08 3.90626759936824e-08 6.54877038149454e-11 0.000209515122984307 0.0135588677913442 blue

ALKBH3-AS1 -0.125714337368431 0.125714337368431 -0.313878319402601 -0.247182720186619 0.0326746165767594 0.196963594471251 0.196963594471251 0.000993845879087772 0.0102639542207834 0.738297879686547 turquoise

ALKBH4 0.433978985977001 -0.433978985977001 0.149613754371998 -0.0810178717550701 -0.352834246041959 3.01312621659499e-06 3.01312621659499e-06 0.124021691533285 0.406778037666425 0.000193386696671555 brown

ALKBH5 0.500143964889635 -0.500143964889635 0.377980617126518 0.21626071042639 -0.177418344536141 4.13168957421148e-08 4.13168957421148e-08 5.97231814605896e-05 0.0252674630176524 0.0675185887245665 brown

ALKBH6 -0.251928463378941 0.251928463378941 -0.248724009063887 -0.103458155500719 0.182639764024158 0.00885181685027975 0.00885181685027975 0.00978506796895571 0.288936104618907 0.0597111356823454 greenyellow

ALKBH7 0.095991456041095 -0.095991456041095 0.152029341248614 0.0370237394220138 -0.156098109111639 0.325334739249187 0.325334739249187 0.117997533811963 0.70497887898028 0.108365173845663 red

ALKBH8 0.678985423816675 -0.678985423816675 0.669889118026316 0.326492627150174 -0.410746941635322 9.14828197857515e-16 9.14828197857515e-16 3.02132835721818e-15 0.000598802924626735 1.11054314348086e-05 blue

ALLC -0.112573782659592 0.112573782659592 -0.212483891812107 -0.116378152452943 0.108546410197227 0.248307965176926 0.248307965176926 0.0279997068836602 0.232577110198888 0.265744166055971 turquoise

ALMS1 0.645754370466874 -0.645754370466874 0.60517175639212 0.294694787471658 -0.371498751072077 5.92093216242532e-14 5.92093216242532e-14 5.04635511413436e-12 0.00206117694101017 8.16058490015495e-05 blue

ALMS1-IT1 0.200288035402381 -0.200288035402381 0.00697794964826627 -0.0108646450596149 -0.028474898072988 0.0385958391928618 0.0385958391928618 0.943132305273565 0.911562414105617 0.770939601263239 grey

ALMS1P -0.0592586628630858 0.0592586628630858 -0.181558396996177 -0.0785486648851232 0.128182986970559 0.544309033737844 0.544309033737844 0.0612643809220875 0.42127343637803 0.188242333259935 yellow

ALOX12 -0.232489100378633 0.232489100378633 -0.386456112346475 -0.327739463524535 0.000538905064262592 0.0159631628221325 0.0159631628221325 3.93098456738759e-05 0.000568875220070904 0.995604474622321 salmon

ALOX12B 0.159299517093786 -0.159299517093786 -0.0894324974499865 -0.130611684501064 -0.0927677004478489 0.101226506919957 0.101226506919957 0.359630931151633 0.179939623870427 0.341920739097622 green

ALOX12P2 -0.196693482033637 0.196693482033637 -0.28771649897511 -0.246057541828081 -0.00308526615515689 0.0422958280988112 0.0422958280988112 0.00265565357516654 0.0106264187876221 0.974839320550164 turquoise

ALOX15 0.636808598773553 -0.636808598773553 0.360236104838405 0.128760553459671 -0.300281236603174 1.66934596402613e-13 1.66934596402613e-13 0.000138220798302192 0.186243073304071 0.00167508692740473 brown

ALOX15B -0.395638185026338 0.395638185026338 -0.349636048564215 -0.0701925022412455 0.384358154393981 2.46612629228448e-05 2.46612629228448e-05 0.000223023610494188 0.472488762495795 4.36444725226157e-05 blue

ALOX5 -0.77466820951193 0.77466820951193 -0.596296856788613 -0.295168574015565 0.357916861208759 1.25997519608415e-22 1.25997519608415e-22 1.22886534178056e-11 0.00202555584264297 0.00015369427843963 brown

ALOX5AP -0.759720181973551 0.759720181973551 -0.712038184559455 -0.394589908322067 0.355932154612625 2.39982896760243e-21 2.39982896760243e-21 8.09997773754858e-18 2.60277480389917e-05 0.000168196060483773 blue

ALOXE3 0.144768672680985 -0.144768672680985 -0.133311889065816 -0.12854698502587 -0.0260871395560765 0.136814391706527 0.136814391706527 0.17102722290899 0.1869805354169 0.789682163212874 turquoise

ALPI 0.0861581507248258 -0.0861581507248258 -0.0831492724671854 -0.0857472915104271 -0.0257184786164805 0.377559859367642 0.377559859367642 0.394504879116211 0.379847258282419 0.792586960581209 green

ALPK1 -0.735256435102087 0.735256435102087 -0.395001274605755 -0.0774423427596013 0.437379336786602 1.92936542190386e-19 1.92936542190386e-19 2.5483261182052e-05 0.427863745944121 2.46902905192367e-06 blue

ALPK2 -0.0274539544487484 0.0274539544487484 -0.107874458055215 -0.134453116350396 -0.0727301059338599 0.778938004118894 0.778938004118894 0.268732280402334 0.167360015125815 0.456588351743227 turquoise

ALPK3 -0.250303615113526 0.250303615113526 0.0253493087784219 0.141539306215957 0.203570826999597 0.00931474989191739 0.00931474989191739 0.795498613629626 0.145882718226682 0.035457451400306 turquoise

ALPL -0.789524351437796 0.789524351437796 -0.478888280029601 -0.196417559788181 0.356364085087804 5.33763010176634e-24 5.33763010176634e-24 1.8106369239401e-07 0.0425916683322014 0.000164936084898003 brown

ALPP -0.252837021804055 0.252837021804055 -0.357318760024771 -0.300747850857427 0.00436760170925957 0.00860189317284843 0.00860189317284843 0.000157937476905856 0.00164601333539132 0.964387577373031 turquoise

ALPPL2 -0.0215763117786789 0.0215763117786789 -0.279744082216457 -0.256972078161931 -0.0330766516328884 0.825410654110852 0.825410654110852 0.00352051094032311 0.0075412937262974 0.735196337028427 green

ALS2 0.20779617730327 -0.20779617730327 0.16943690610243 0.0356137189876945 -0.183553499828788 0.0317363707493741 0.0317363707493741 0.0810333455742372 0.715723752697884 0.0584239033035367 yellow

ALS2CL 0.346403935680199 -0.346403935680199 0.084607677930796 -0.0624802798992961 -0.227794813372312 0.000257197929794916 0.000257197929794916 0.386235701779794 0.52260502931463 0.0182865140052453 green

ALS2CR11 -0.243413841989748 0.243413841989748 -0.339670969202659 -0.249259933306138 0.066288225986417 0.0115225209230316 0.0115225209230316 0.000344450886160528 0.0096232133853161 0.49752847448839 turquoise

ALS2CR12 -0.0746420885926297 0.0746420885926297 -0.295682017747185 -0.313604169881648 -0.106184362032629 0.444806584690361 0.444806584690361 0.00198758254165625 0.00100460738354658 0.276348214548718 pink

ALX1 0.0795537567197518 -0.0795537567197518 -0.179654759562127 -0.208292441467587 -0.0946204658961292 0.415337367464839 0.415337367464839 0.0640787549879361 0.0313218194069755 0.33232429436533 green

ALX3 0.127502875728387 -0.127502875728387 -0.000793943569339388 -0.0330920784279557 -0.0549853725516351 0.19061650745674 0.19061650745674 0.993524316294986 0.735077409732799 0.573762864824456 green

ALX4 -0.249272638458375 0.249272638458375 -0.219781417814918 -0.0871607777292631 0.168610118712807 0.00961940501694318 0.00961940501694318 0.0229286469088952 0.372013105053178 0.0825490488263164 turquoise

ALYREF -0.241861240781687 0.241861240781687 -0.0778050836057864 -0.0315775404657282 0.0584658591117011 0.0120790278340607 0.0120790278340607 0.42569642414853 0.746782643085438 0.549717044420648 red

AMACR 0.0477137668793304 -0.0477137668793304 -0.121717992602942 -0.13173401521149 -0.0481859037243626 0.625524272392194 0.625524272392194 0.211693651855462 0.176194771198941 0.622103808046927 green

AMBN -0.10955743720461 0.10955743720461 -0.172023028451719 -0.0386801763643474 0.182075983306911 0.261290845350189 0.261290845350189 0.0764356495755928 0.692430065784189 0.0605168685414619 turquoise

AMBP -0.0978903478160562 0.0978903478160562 -0.262172170082425 -0.166498210165972 0.0950785053270096 0.315810728721581 0.315810728721581 0.00637260013715003 0.0865236434884398 0.329978567625321 turquoise

AMBRA1 0.171761017584115 -0.171761017584115 0.0973574138755308 0.0757253755291791 -0.0117371898567405 0.0768916974432396 0.0768916974432396 0.318465290321618 0.438207894412789 0.904492234562256 grey

AMD1 0.461389885134573 -0.461389885134573 0.606272653376345 0.378755696343275 -0.230505451412304 5.68153462845909e-07 5.68153462845909e-07 4.51019025902717e-12 5.75093758276624e-05 0.0169117204495065 blue

AMDHD1 0.0843941868639191 -0.0843941868639191 0.164240726963955 0.162207634679737 0.038648007710902 0.387439639256122 0.387439639256122 0.0909390958878779 0.0950667921042657 0.692672991284674 turquoise

AMELX -0.303171778765396 0.303171778765396 -0.449152885142086 -0.298957950317781 0.139628378864478 0.00150221704408396 0.00150221704408396 1.21823397116873e-06 0.00176008790206839 0.151458228657909 turquoise

AMELY -0.0360999291106665 0.0360999291106665 -0.306531325180146 -0.267060265351497 -0.0116187043837325 0.712012253603506 0.712012253603506 0.00132175357964181 0.00542351433305713 0.905451890139097 green

AMER1 0.276397221218158 -0.276397221218158 -0.0462517656522702 -0.103528475514454 -0.10900361096801 0.00395338300300975 0.00395338300300975 0.636166195683544 0.288606702461883 0.26372397176718 green

AMER2 -0.222648019566147 0.222648019566147 -0.332636642023192 -0.279364202912782 0.00509924970957032 0.021163672025868 0.021163672025868 0.000464135413885216 0.00356740732245938 0.958426829397801 turquoise

AMER3 -0.0730937645795833 0.0730937645795833 -0.266377469155193 -0.17993340767671 0.0783455153109519 0.454334240105485 0.454334240105485 0.0055480408130458 0.0636603407985863 0.422479169366902 green

AMFR -0.353604372753483 0.353604372753483 -0.25650116095399 -0.075116737298952 0.241908272219273 0.000186817243143706 0.000186817243143706 0.00765594497401875 0.441908477294534 0.0120618313353083 grey

AMHR2 -0.120202053010889 0.120202053010889 -0.328554182363194 -0.242752982159567 0.0613186873268495 0.217481544054174 0.217481544054174 0.000550067861559519 0.0117565941905524 0.530379867936209 turquoise

AMICA1 -0.00360447085156924 0.00360447085156924 0.357641056531347 0.333726757886686 0.0511048916908801 0.970606902429069 0.970606902429069 0.000155637647126201 0.000443377187388034 0.60113721394256 tan

AMIGO1 0.800798978196553 -0.800798978196553 0.608825189959019 0.265571658648245 -0.426155698967144 4.06703237769044e-25 4.06703237769044e-25 3.47026762407896e-12 0.00569826883344086 4.7259928925892e-06 brown

AMIGO2 0.586002931962128 -0.586002931962128 0.414012075361364 0.12167176707611 -0.389732594423423 3.33641414324332e-11 3.33641414324332e-11 9.29882905930677e-06 0.211868503769691 3.33376459581796e-05 turquoise

AMMECR1 0.475741503922622 -0.475741503922622 0.221180264363055 -0.0364139437037856 -0.380223176506704 2.23455754485647e-07 2.23455754485647e-07 0.0220522287698993 0.709618776742929 5.35262210737061e-05 turquoise

AMMECR1L 0.338925037876546 -0.338925037876546 0.438050841678037 0.268038160401351 -0.176087443928733 0.000355637021148623 0.000355637021148623 2.37320365972703e-06 0.00524950005346423 0.0696357387005882 yellow

AMN -0.108603452902463 0.108603452902463 -0.238747050960629 -0.221957428698523 -0.0327156483467312 0.26549154445503 0.26549154445503 0.0132664217950936 0.0215778408478081 0.737981142281225 yellow

AMN1 -0.348178829647977 0.348178829647977 0.0188736455264702 0.195046697687513 0.303650188377059 0.000237874947424808 0.000237874947424808 0.846994523664954 0.0440871749293797 0.00147522151359692 green

AMOT 0.0113847424140142 -0.0113847424140142 0.101842356562442 0.137338655311722 0.0863095268761228 0.907347228936817 0.907347228936817 0.296573828701886 0.15834785829722 0.376719208442857 turquoise

AMOTL1 -0.196674932887636 0.196674932887636 -0.54295272546079 -0.446193731797496 0.0249521902977381 0.0423156623880913 0.0423156623880913 1.52332729323326e-09 1.4585563073745e-06 0.798633834143967 turquoise

AMOTL2 -0.13615752837722 0.13615752837722 -0.367699020075336 -0.337148308897003 -0.0424266384713184 0.161991967896643 0.161991967896643 9.76957783714078e-05 0.000383643082812841 0.664357184449599 turquoise

AMPD1 -0.407998489468181 0.407998489468181 -0.517543219639093 -0.401492157771441 0.0641868088335356 1.28771434435975e-05 1.28771434435975e-05 1.14120634078901e-08 1.81869502530016e-05 0.511288404144486 blue

AMPD2 0.606310273792251 -0.606310273792251 0.734110953594119 0.534271856006043 -0.150794016372189 4.49287685625138e-12 4.49287685625138e-12 2.3412740701257e-19 3.08845357846924e-09 0.121049371414903 black

AMPD3 -0.343381922775124 0.343381922775124 -0.387165184197683 -0.265515686996501 0.107099554682937 0.000293467377860692 0.000293467377860692 3.7938282913039e-05 0.00570883674568217 0.272206342526578 brown

AMPH -0.543034688341712 0.543034688341712 -0.24728335051395 0.0561124365010919 0.451218252465201 1.51305032757737e-09 1.51305032757737e-09 0.0102320719104882 0.565922684140524 1.07329364365461e-06 purple

AMT 0.121188647641518 -0.121188647641518 -0.00394691307033323 -0.0518986576043413 -0.0823441148499068 0.213702101533593 0.213702101533593 0.967815844019558 0.595490592049848 0.39911500795033 yellow

AMZ1 -0.195692639618535 0.195692639618535 -0.295809732282926 -0.163585773942299 0.148450503654781 0.0433771458652346 0.0433771458652346 0.0019782375938353 0.0922530127823004 0.127005783137011 turquoise

AMZ2 -0.164460978854241 0.164460978854241 -0.0708419301515389 -0.0794574528431164 -0.0327705209612344 0.0905005911817284 0.0905005911817284 0.468391120008177 0.415904018949592 0.737557631474779 yellow

AMZ2P1 0.289830187685132 -0.289830187685132 0.316852056673476 0.214717724123539 -0.0920211661631974 0.00246106314755188 0.00246106314755188 0.000883688602408365 0.0263549083199964 0.345836324684577 yellow

ANAPC10 0.0162124910302532 -0.0162124910302532 0.0758115912607649 0.0607669840980319 -0.00608643466752619 0.86835807922553 0.86835807922553 0.437685117830251 0.534092729644 0.950387907880753 yellow

ANAPC11 -0.321222775665033 0.321222775665033 -0.396311362259282 -0.260907174765941 0.128085104504388 0.000741927760562286 0.000741927760562286 2.38195204380125e-05 0.00664104576322305 0.188582696379951 greenyellow

ANAPC13 0.0869694925513649 -0.0869694925513649 0.400648250401257 0.397330817006872 0.0970630279559272 0.373067475055735 0.373067475055735 1.9009891934489e-05 2.25959281315008e-05 0.319937793851269 grey

ANAPC15 -0.695795289025147 0.695795289025147 -0.685538842493506 -0.428667608429472 0.25997753507352 8.95204038431728e-17 8.95204038431728e-17 3.7658868647681e-16 4.09505036107604e-06 0.00684466547457651 brown

ANAPC16 0.168857870580195 -0.168857870580195 0.293325917582876 0.196270769859227 -0.0894362016655623 0.0820925010359388 0.0820925010359388 0.00216729856195978 0.0427497561259411 0.3596109517804 turquoise

ANAPC2 0.320786608604673 -0.320786608604673 0.364061598756328 0.202644919542192 -0.180471586309413 0.000755076106296578 0.000755076106296578 0.000115820867592768 0.0363201026442811 0.0628585165723859 blue

ANAPC4 0.0678900914384287 -0.0678900914384287 0.11943897968466 0.0808563011538823 -0.0348280193732902 0.48717162248564 0.48717162248564 0.220437037751905 0.407717467748278 0.721735400888524 yellow

ANAPC5 0.400035026764157 -0.400035026764157 0.57026597458106 0.343083509662033 -0.239166949973212 1.9629670413862e-05 1.9629670413862e-05 1.43800082849393e-10 0.000297293895250108 0.0131006094198178 red

ANAPC7 0.168864331601253 -0.168864331601253 0.203252296699309 0.123354349098308 -0.0834220249710949 0.0820806219686257 0.0820806219686257 0.0357522553458883 0.205570090862722 0.392950396289392 yellow

ANG -0.463987576883294 0.463987576883294 -0.0698416544194661 0.193163456662681 0.428190267628633 4.81362901427829e-07 4.81362901427829e-07 0.474710547419843 0.0462126527096395 4.20846718497794e-06 grey

ANGEL1 0.485575744582624 -0.485575744582624 0.310425160581937 0.141854778645264 -0.206352744758784 1.14976449899689e-07 1.14976449899689e-07 0.00113739856166267 0.144977353052823 0.0329685802618962 brown

ANGEL2 0.357672838729882 -0.357672838729882 0.299470863553837 0.171861173188961 -0.139685708054405 0.00015541254782299 0.00015541254782299 0.0017266877815959 0.0767171123346775 0.151288664323865 yellow

ANGPT1 0.177990295896227 -0.177990295896227 0.209944259105555 0.100206106743194 -0.132319700129268 0.0666249304406201 0.0666249304406201 0.0299748179361763 0.30444267286063 0.174263422628362 grey

ANGPT2 0.0324510689101891 -0.0324510689101891 -0.0637206755350381 -0.0581801855872625 -0.00693479769850273 0.740024283775139 0.740024283775139 0.5143669353382 0.551672107190371 0.943483395517825 green

ANGPT4 -0.287002294979409 0.287002294979409 -0.390527647420408 -0.317389261363726 0.0239564993807097 0.0027244721101199 0.0027244721101199 3.20224470501141e-05 0.000865021464019014 0.806508718724968 turquoise

ANGPTL1 -0.11100961061295 0.11100961061295 -0.342292969280205 -0.368266141827233 -0.131786761297583 0.254983816127388 0.254983816127388 0.00030765462708144 9.51201543545774e-05 0.17602019447926 turquoise

ANGPTL2 -0.285431794698514 0.285431794698514 -0.168361743754697 -0.00958065198267629 0.226160988153793 0.00288147988318317 0.00288147988318317 0.0830087788162383 0.921978939818801 0.0191609042735706 turquoise

ANGPTL3 -0.235313198842475 0.235313198842475 -0.193500000622745 -0.14257901291764 0.0367728628139166 0.0146924030390571 0.0146924030390571 0.0458266987203956 0.142914950997426 0.706886485600838 turquoise

ANGPTL4 -0.106865650378465 0.106865650378465 -0.290748066633867 -0.169953087400373 0.130362820050004 0.273260913369518 0.273260913369518 0.0023806405888486 0.0800984055192786 0.180777842340641 green

ANGPTL6 -0.118536146045935 0.118536146045935 -0.126958255581426 -0.0135504523980172 0.159814005649538 0.22397037433535 0.22397037433535 0.192533310227165 0.889823945094069 0.100114524674738 turquoise

ANGPTL7 -0.108135181619018 0.108135181619018 -0.202687157689249 -0.123717900323271 0.0819917035794685 0.267570176122083 0.267570176122083 0.036280369857317 0.204227013248871 0.401142838714151 turquoise

ANHX 0.0425274276304997 -0.0425274276304997 -0.188585097465601 -0.165487099568044 -0.00915882841976861 0.66360811374469 0.66360811374469 0.0517365277161062 0.0884797209242104 0.9254039947547 turquoise

ANK1 -0.0581961801089511 0.0581961801089511 -0.409941280076322 -0.393027015683004 -0.0763829228919585 0.551562556619366 0.551562556619366 1.15994618793065e-05 2.81974101769464e-05 0.434229748901326 pink

ANK2 -0.332324728799049 0.332324728799049 -0.385608191696013 -0.212540032785774 0.194711464448386 0.000470237269476581 0.000470237269476581 4.10108572138506e-05 0.0279573254623679 0.0444594632868009 turquoise

ANK3 0.754329093461354 -0.754329093461354 0.631394732889235 0.217618378468715 -0.53998692745367 6.59370400555404e-21 6.59370400555404e-21 3.07551116317621e-13 0.0243425094497666 1.94375575357123e-09 blue

ANKAR -0.0856610160282947 0.0856610160282947 -0.349642359696948 -0.32384086932837 -0.0458537906844399 0.380328649051077 0.380328649051077 0.000222961198506699 0.000667311601712774 0.639076044147353 turquoise

ANKDD1A -0.449462686359083 0.449462686359083 -0.195744898690028 0.0685298795280074 0.398073643236598 1.19536894303704e-06 1.19536894303704e-06 0.0433201206567053 0.48306738259089 2.17417579330409e-05 blue

ANKEF1 0.734455462850786 -0.734455462850786 0.462961800024784 0.0705554922346082 -0.54691142084408 2.2091392343822e-19 2.2091392343822e-19 5.14010767057753e-07 0.470196039439953 1.09621593956823e-09 blue

ANKFN1 -0.0373177161542368 0.0373177161542368 -0.0794578510154042 -0.0522680966161542 0.0257516145738714 0.70274587426821 0.70274587426821 0.415901675180226 0.592870668551806 0.792325755055945 green

ANKFY1 0.382853982408803 -0.382853982408803 0.445319016611751 0.442407337529858 0.109200745475002 4.70229906701949e-05 4.70229906701949e-05 1.53778728731269e-06 1.83193005520537e-06 0.262856140469943 cyan

ANKH 0.489765360631015 -0.489765360631015 0.160845986805883 -0.128529458072313 -0.449592572394505 8.60781621995895e-08 8.60781621995895e-08 0.0979130230741957 0.187041151082623 1.18590421077247e-06 purple

ANKHD1 0.352346370526768 -0.352346370526768 0.39497280988528 0.304471670816624 -0.0522666569214713 0.000197658468643104 0.000197658468643104 2.55205907022739e-05 0.00142989309074348 0.592880868214928 blue

ANKIB1 0.339856157534776 -0.339856157534776 0.438296060917056 0.267030942850012 -0.1781488680175 0.000341724488518564 0.000341724488518564 2.33909576124673e-06 0.00542881054723534 0.0663788730480969 yellow

ANKLE1 -0.364891212281486 0.364891212281486 -0.417080131171943 -0.217212700299754 0.232100025646523 0.000111431217092238 0.000111431217092238 7.85666410657722e-06 0.0246158107898422 0.016145492051585 blue

ANKLE2 0.332156710570253 -0.332156710570253 0.279305215196778 0.0676273833596376 -0.287445008324187 0.000473554636586624 0.000473554636586624 0.00357473942341064 0.488862254204778 0.00268162692496481 turquoise

ANKMY1 0.326346821432239 -0.326346821432239 0.10125286089103 -0.0333937062211323 -0.202426256989153 0.00060239543367945 0.00060239543367945 0.299393158659481 0.732753380389427 0.036526380084574 yellow

ANKMY2 0.271003953521951 -0.271003953521951 0.518444090457623 0.424112376921612 -0.0271170165092946 0.00475174504814055 0.00475174504814055 1.06554790625426e-08 5.30580660020764e-06 0.781582802990538 yellow

ANKRA2 0.169786180361386 -0.169786180361386 0.290415466805392 0.237180946098388 -0.0158566171700007 0.0803997682526122 0.0803997682526122 0.0024095041537359 0.0139010897185924 0.871222556070343 yellow

ANKRD1 0.0208792288749378 -0.0208792288749378 -0.0544423771597139 -0.0731902062828426 -0.0457528618598618 0.830965901262067 0.830965901262067 0.577558115770203 0.453737492127968 0.639814872620118 yellow

ANKRD10 0.0744706598732578 -0.0744706598732578 0.184534823355658 0.11785849602469 -0.0657938922464875 0.44585591367211 0.44585591367211 0.0570668490705461 0.226648521155612 0.500747756843848 yellow

ANKRD10-IT1 -0.505859196960257 0.505859196960257 -0.129976430098948 -0.00423444359875941 0.179960821840817 2.72909966604019e-08 2.72909966604019e-08 0.182084927958411 0.965472639317692 0.0636192965329262 brown

ANKRD11 0.199501407120513 -0.199501407120513 0.261481634259604 0.11797393033138 -0.176387783569551 0.0393814789210203 0.0393814789210203 0.00651792162481002 0.226190728351605 0.06915333534895 pink

ANKRD12 0.128630243928651 -0.128630243928651 0.327501659165374 0.223627145099781 -0.092243191471974 0.186692785996147 0.186692785996147 0.000574475040708202 0.0205881692721719 0.344668866786576 yellow

ANKRD13A -0.169157683371974 0.169157683371974 0.30064435509697 0.390977145809567 0.230274247396429 0.0815427187813319 0.0815427187813319 0.0016524221175083 3.13005350350073e-05 0.0170253897480338 black

ANKRD13B 0.323866240581008 -0.323866240581008 -0.000478157189677191 -0.149816238621804 -0.253419617059974 0.000666623425846403 0.000666623425846403 0.996099954462666 0.123507813682056 0.00844492529139926 green

ANKRD13C 0.228361359914118 -0.228361359914118 0.155296919019959 0.0204775196216228 -0.188867459113861 0.0179914571047663 0.0179914571047663 0.110211735606776 0.834171037766034 0.0513807781807406 yellow

ANKRD13D 0.00838365061926327 -0.00838365061926327 0.360056962178127 0.409878266997426 0.176789422482896 0.931701667782929 0.931701667782929 0.000139362391372224 1.16389606256851e-05 0.068512457888253 black

ANKRD16 0.395251889342801 -0.395251889342801 0.283366878474107 0.0207491423912535 -0.372804699270459 2.5156799236772e-05 2.5156799236772e-05 0.00310029025031641 0.832003527716954 7.66717235677093e-05 blue

ANKRD17 0.629644130759201 -0.629644130759201 0.440060991402581 0.137603301137146 -0.400216412219589 3.7378280606941e-13 3.7378280606941e-13 2.10694565028398e-06 0.157539806859937 1.94443986778977e-05 blue

ANKRD18A -0.0747245224581489 0.0747245224581489 -0.133761472710287 -0.149856610008736 -0.061583819236376 0.444302495119693 0.444302495119693 0.169575518579175 0.123405552688036 0.528600186147649 grey

ANKRD18B -0.215344773254916 0.215344773254916 -0.230002820720355 -0.15885111596105 0.061730813923858 0.0259082702178731 0.0259082702178731 0.017159676777689 0.102203539480985 0.527614786265944 turquoise

ANKRD19P -0.316851521469226 0.316851521469226 -0.463925936854314 -0.368364059551533 0.0431770606487876 0.000883707382152353 0.000883707382152353 4.83267746273274e-07 9.46819032927911e-05 0.658787980289485 turquoise

ANKRD2 0.0423013852381777 -0.0423013852381777 -0.0365295770775746 0.00644176428579651 0.0635222116531961 0.665288534150226 0.665288534150226 0.708738107501876 0.947495535370902 0.515680547469116 green

ANKRD20A11P 0.245257376474356 -0.245257376474356 0.255582834790643 0.119412242933375 -0.165455034956572 0.0108909640053258 0.0108909640053258 0.00788396121486253 0.220541105391552 0.0885423225707669 greenyellow

ANKRD20A12P -0.0989975685196278 0.0989975685196278 -0.206455724775667 -0.087018249405768 0.149665210886576 0.310341545884224 0.310341545884224 0.0328793521405278 0.372798552662858 0.12389094622762 yellow

ANKRD22 -0.705131989620501 0.705131989620501 -0.695900816543797 -0.333492950891803 0.436325681047136 2.29495773131529e-17 2.29495773131529e-17 8.81792968862735e-17 0.000447756073795212 2.62679981074688e-06 brown

ANKRD23 0.596794380810879 -0.596794380810879 0.561416877773574 0.329627939954036 -0.249248268334469 1.1698987451733e-11 1.1698987451733e-11 3.16281748016513e-10 0.000526152478952007 0.00962671112616033 blue

ANKRD24 -0.253157890724505 0.253157890724505 -0.327053687731453 -0.190742935175696 0.147374122880988 0.00851512679600978 0.00851512679600978 0.000585161495187758 0.0490687411304255 0.129815801164843 yellow

ANKRD26 0.287544312695742 -0.287544312695742 0.0631870223963247 -0.149266364710734 -0.344153521857187 0.00267210011381368 0.00267210011381368 0.517903020623518 0.12490714843804 0.000283783027225496 brown

ANKRD26P3 -0.29933876163023 0.29933876163023 -0.236870903542373 -0.160871806610632 0.0681922696354583 0.00173523468145316 0.00173523468145316 0.0140298212601444 0.0978584351322996 0.485230840091807 turquoise

ANKRD27 0.207216714733502 -0.207216714733502 0.0824594328421253 -0.0272356586877919 -0.164922227149697 0.0322262804114643 0.0322262804114643 0.3984527725683 0.780651233897522 0.0895877137446641 yellow

ANKRD28 -0.148366258651013 0.148366258651013 -0.247970070984487 -0.282065977641954 -0.121388175893114 0.12722401746064 0.12722401746064 0.0100168112722808 0.00324569577326245 0.212943462748798 brown

ANKRD29 -0.10531249527492 0.10531249527492 -0.182842023744882 -0.0804696892659121 0.126772878960583 0.280333204410799 0.280333204410799 0.0594242205657563 0.409970536404593 0.193188921982301 turquoise

ANKRD30A -0.123476473449303 0.123476473449303 -0.395551776112483 -0.333053560930966 0.00462165269438964 0.205118219615626 0.205118219615626 2.47713062673778e-05 0.000456093044635584 0.962317580845701 turquoise

ANKRD30B 0.229565787601264 -0.229565787601264 -0.0139011201557299 -0.109481508440486 -0.16567988562846 0.0173778212790608 0.0173778212790608 0.886991365100335 0.261623515506036 0.0881040741745424 brown

ANKRD30BP2 -0.206833110982509 0.206833110982509 -0.167211957556984 -0.069216736776865 0.123354851611858 0.0325541016432946 0.0325541016432946 0.0851636812583068 0.478681877156157 0.205568230057046 red

ANKRD30BP3 -0.189983474641565 0.189983474641565 -0.208718010327778 -0.112842307466614 0.109121481603117 0.049994398310526 0.049994398310526 0.030969978846972 0.247174141824161 0.263204844329113 turquoise

ANKRD31 0.248841804071364 -0.248841804071364 0.033183956804098 -0.112692965808374 -0.238921109224647 0.00974928909345281 0.00974928909345281 0.734369236388301 0.247804282529993 0.013197467974778 grey

ANKRD32 -0.414753135258375 0.414753135258375 -0.210160364909409 -0.0948814467063433 0.141662185038235 8.92926162837896e-06 8.92926162837896e-06 0.0298022758358719 0.330986452552902 0.145529566039478 brown

ANKRD33 -0.126677715751224 0.126677715751224 -0.303000067792265 -0.251586879560559 0.00954187531514429 0.19352610788557 0.19352610788557 0.00151201515243907 0.00894741948579894 0.922293733795663 turquoise

ANKRD33B -0.262160369935481 0.262160369935481 -0.460721200181128 -0.345431478680474 0.0774594576938015 0.00637505911418851 0.00637505911418851 5.92791320778168e-07 0.00026839019212424 0.427761344688946 blue

ANKRD34A 0.188286031821632 -0.188286031821632 0.121953016613956 0.0901231448420302 -0.0227299636895998 0.0521155353860475 0.0521155353860475 0.210806243896299 0.355917683617305 0.816235909678803 yellow

ANKRD34C -0.3155330625711 0.3155330625711 -0.311850303873492 -0.212666057510828 0.0882994363326199 0.000931093467279835 0.000931093467279835 0.00107600722114533 0.0278623865140735 0.365774535453539 turquoise

ANKRD35 -0.262813215109781 0.262813215109781 -0.288479412171848 -0.179199240036778 0.111413632921177 0.00624027346320679 0.00624027346320679 0.00258387485649233 0.0647675745843138 0.253247791291354 turquoise

ANKRD36 0.339454563543696 -0.339454563543696 0.420965031019004 0.133492692201266 -0.379693750866662 0.000347662277966315 0.000347662277966315 6.33180106101099e-06 0.170442311255196 5.4932545633207e-05 blue

ANKRD36B 0.697151552807639 -0.697151552807639 0.640568184350933 0.20666707866022 -0.571769913295013 7.36978526489006e-17 7.36978526489006e-17 1.08432785003153e-13 0.0326968605135464 1.25478027209798e-10 blue

ANKRD36BP2 -0.0112142057773742 0.0112142057773742 -0.0692810360399054 -0.213612002972428 -0.262561684593874 0.908729083954755 0.908729083954755 0.478272433830184 0.0271584795334197 0.00629190177715356 red

ANKRD36C 0.529640186958135 -0.529640186958135 0.381388127985962 0.0929810935099347 -0.391423012214159 4.46677315843433e-09 4.46677315843433e-09 5.05492642962039e-05 0.340806646420233 3.059953455865e-05 brown

ANKRD37 -0.445843245121724 0.445843245121724 -0.26337883790043 -0.0663058859704097 0.266755246277305 1.4898270789804e-06 1.4898270789804e-06 0.00612554636054688 0.497413667501309 0.00547883219237804 brown

ANKRD39 -0.316184105394935 0.316184105394935 -0.173163812777663 -0.0535749972295292 0.158454921729606 0.000907412095827494 0.000907412095827494 0.0744753146514692 0.583644691190314 0.103072958315917 yellow

ANKRD40 0.613383602204794 -0.613383602204794 0.444909006797023 0.182882020942377 -0.330398007855476 2.16037090485701e-12 2.16037090485701e-12 1.57631352732291e-06 0.0593676163634561 0.000509593227301123 blue

ANKRD44 0.164505418761042 -0.164505418761042 0.592110172873406 0.561623957720868 0.100054672570499 0.0904123183259755 0.0904123183259755 1.8525717364603e-11 3.10583494833108e-10 0.305177774781281 black

ANKRD44-IT1 0.0605920774748566 -0.0605920774748566 0.274810041770744 0.174785537991556 -0.0992183595661513 0.535272513863417 0.535272513863417 0.00417484663373681 0.0717584280022252 0.309258350188042 black

ANKRD45 -0.19782840541148 0.19782840541148 -0.338478017253757 -0.296417428885853 -0.0154147278314707 0.0410969356776577 0.0410969356776577 0.000362500102988309 0.00193431429958107 0.874781697454601 turquoise

ANKRD46 0.62231661098024 -0.62231661098024 0.582003484506207 0.287731330514732 -0.349951501397291 8.34506035391713e-13 8.34506035391713e-13 4.87240480726729e-11 0.00265424120077799 0.000219923744609947 blue

ANKRD49 0.0566337659956435 -0.0566337659956435 0.143975194759768 0.10270141217241 -0.0331036153267671 0.56231339702263 0.56231339702263 0.139001781786458 0.292496739193576 0.73498847427635 yellow

ANKRD50 -0.425740426529421 0.425740426529421 -0.0717164835666004 0.210829546001677 0.460853724371899 4.83875657739682e-06 4.83875657739682e-06 0.4629037994179 0.0292733204178249 5.8782919016015e-07 brown

ANKRD52 0.343268617199537 -0.343268617199537 -0.0651464478085854 -0.212210366411486 -0.266137411097028 0.00029491489589732 0.00029491489589732 0.504980555767759 0.0282069757688441 0.0055924223534946 green

ANKRD53 0.25781885204403 -0.25781885204403 0.0174002904336186 -0.0633241016589075 -0.132459339897775 0.00733894730026714 0.00733894730026714 0.858809848231212 0.51699352643698 0.173805257192228 green

ANKRD54 0.237603202259648 -0.237603202259648 0.19439008681782 0.118291870436676 -0.0792486372889681 0.0137274201791498 0.0137274201791498 0.0448188154119676 0.224933195659952 0.41713423270421 grey

ANKRD55 -0.499658098802948 0.499658098802948 -0.600417325131664 -0.491189297416731 0.0313726123098044 4.2784612968852e-08 4.2784612968852e-08 8.15687418041786e-12 7.79438037487718e-08 0.748370946786741 brown

ANKRD6 -0.266558460868584 0.266558460868584 -0.290909981373172 -0.283026036400827 -0.0611907260640098 0.00551478676870888 0.00551478676870888 0.00236670257857063 0.00313781004213811 0.53123987177345 yellow

ANKRD60 0.175216633289554 -0.175216633289554 -0.165526762664732 -0.228634949896521 -0.149465824976124 0.0710498388906186 0.0710498388906186 0.0884023329586666 0.0178504494624268 0.124398154824427 turquoise

ANKRD65 -0.189983159835937 0.189983159835937 -0.250183043438788 -0.0739347415244353 0.23481612015497 0.0499947849776573 0.0499947849776573 0.00934993530889607 0.449145261341201 0.0149094835850897 turquoise

ANKRD7 -0.0103122405137589 0.0103122405137589 -0.185211312985225 -0.22939513078073 -0.122412968868859 0.916042121499892 0.916042121499892 0.056146477386182 0.0174636528236848 0.209077228616851 turquoise

ANKRD9 -0.464659190239416 0.464659190239416 -0.599696875764385 -0.39951102204375 0.185833743146044 4.61065401552818e-07 4.61065401552818e-07 8.76649862774115e-12 2.01742522542894e-05 0.0553104490930765 pink

ANKS1A -0.57559489302456 0.57559489302456 -0.576214771868474 -0.303526382339605 0.314826295254417 8.84446132517323e-11 8.84446132517323e-11 8.35353574127189e-11 0.00148216501348408 0.00095743958732177 brown

ANKS1B -0.0112675970983497 0.0112675970983497 -0.174562839300565 -0.206806632125194 -0.0994317949129297 0.908296425660819 0.908296425660819 0.0721267057857368 0.0325768335178741 0.308213585119641 yellow

ANKS3 0.286939371515413 -0.286939371515413 0.101601608928755 0.0453052938499785 -0.0694445229554674 0.00273061153044838 0.00273061153044838 0.297723109743795 0.643095436950706 0.47723223590585 green

ANKS4B -0.0217661016049037 0.0217661016049037 -0.195696307593469 -0.210564168713304 -0.0753766419237772 0.823899645232892 0.823899645232892 0.0433731413246432 0.0294821279572275 0.440326077930366 turquoise

ANKS6 0.516302975414312 -0.516302975414312 0.265884756379876 0.0248820269245524 -0.340623646948318 1.25382482662724e-08 1.25382482662724e-08 0.00563947347282227 0.799188103057862 0.00033063604389413 brown

ANKUB1 -0.00705683592902886 0.00705683592902886 -0.215440451390099 -0.188295262659006 -0.0091775577449211 0.942490503756371 0.942490503756371 0.0258406905266648 0.0521038029529998 0.925251890018691 turquoise

ANKZF1 0.401921512565619 -0.401921512565619 0.431286560421866 0.186858213071281 -0.304039947379652 1.77812147326132e-05 1.77812147326132e-05 3.52242115588396e-06 0.0539566979457025 0.00145355484085527 brown

ANLN -0.441486554346683 0.441486554346683 -0.600502657750807 -0.48050144511568 0.0496234626319723 1.93553487128453e-06 1.93553487128453e-06 8.08743902180006e-12 1.62420392511691e-07 0.611738865432915 magenta

ANO1 -0.086497507040004 0.086497507040004 -0.252871475771341 -0.219957697463469 -0.00898715631322014 0.375676866608139 0.375676866608139 0.00859253927060486 0.0228165857817665 0.926798304084988 yellow

ANO10 -0.732654806066727 0.732654806066727 -0.690824004069902 -0.416941154641256 0.287476833942917 2.98991262004861e-19 2.98991262004861e-19 1.80956125728502e-16 7.91715368815779e-06 0.00267857039358124 brown

ANO2 -0.22928268628619 0.22928268628619 -0.464219047374646 -0.293069990446315 0.171307741213224 0.0175204065807978 0.0175204065807978 4.74273350598598e-07 0.00218767849330377 0.0776858159414573 turquoise

ANO3 -0.115747768096213 0.115747768096213 -0.34421093660859 -0.336955617333008 -0.0759184777619555 0.235134358084982 0.235134358084982 0.000283074342993117 0.000386799502432262 0.437037493549034 turquoise

ANO4 -0.283669628135706 0.283669628135706 -0.403253217047585 -0.238185450976497 0.176619190171031 0.00306730228115808 0.00306730228115808 1.65761664359459e-05 0.0134910464908241 0.0687835000959583 turquoise

ANO5 -0.162209234842487 0.162209234842487 -0.0122831767831292 0.0738435942245142 0.142934047831836 0.09506348616256 0.09506348616256 0.900071891674307 0.449706050247895 0.141912060018926 red

ANO6 -0.0488911529679746 0.0488911529679746 0.0267495905730384 0.122801702422132 0.16977317894174 0.617009490559517 0.617009490559517 0.784469757234102 0.207623847780632 0.0804232813156362 salmon

ANO7 0.158312986328819 -0.158312986328819 -0.101047273476086 -0.180502321929299 -0.160665565275479 0.103385831465473 0.103385831465473 0.300380535115933 0.0628129735927072 0.0982951377333692 green

ANO8 0.0166752915591646 -0.0166752915591646 -0.0230554372207197 -0.0297690618444057 -0.0172965315477888 0.864635486825136 0.864635486825136 0.813651915425756 0.760835201721009 0.859643133056673 yellow

ANO9 0.652405872781831 -0.652405872781831 0.421739067694175 0.162780435424552 -0.331132321123611 2.67859383364552e-14 2.67859383364552e-14 6.06340968192695e-06 0.0938891714276118 0.000494250130340694 brown

ANP32A -0.0108962368586111 0.0108962368586111 0.151460464554256 0.159351246441911 0.0522045427304182 0.911306303776024 0.911306303776024 0.119395455031239 0.101114265900305 0.593320997972066 tan

ANP32A-IT1 -0.421364629715386 0.421364629715386 0.0600619146521003 0.231412262338909 0.306027207941348 6.19187473792122e-06 6.19187473792122e-06 0.538856471978174 0.0164722088753969 0.00134751107682737 purple

ANP32B 0.413844184450187 -0.413844184450187 0.119149812163157 -0.0979399048120567 -0.337673575309039 9.38453423324349e-06 9.38453423324349e-06 0.221564410078652 0.315564613557561 0.000375158948131336 brown

ANP32C -0.0022867584254015 0.0022867584254015 -0.107550564297863 -0.118388669938008 -0.0459490800374818 0.981349864339544 0.981349864339544 0.270180699787715 0.224551308824677 0.638378822063696 green

ANP32D 0.0891347121764843 -0.0891347121764843 -0.149648400181448 -0.180261887771487 -0.0902807655448878 0.361239338914731 0.361239338914731 0.123933648702468 0.0631699591434566 0.35507359184944 turquoise

ANP32E -0.268784879940275 0.268784879940275 -0.371231174393729 -0.305752813045761 0.0159099314144725 0.00511998858766017 0.00511998858766017 8.26526409249274e-05 0.00136172209661515 0.87079331552924 yellow

ANPEP -0.472329508286924 0.472329508286924 -0.16453773810287 -0.0156209856310955 0.210409892587469 2.80042569939575e-07 2.80042569939575e-07 0.0903481639525637 0.873120105092828 0.0296040968734949 purple

ANTXR1 -0.439070496614918 0.439070496614918 -0.514625567132849 -0.308370103805455 0.217933173299515 2.23439820656771e-06 2.23439820656771e-06 1.4231712366047e-08 0.00123155261329179 0.0241322252943637 turquoise

ANTXR2 -0.316393063542401 0.316393063542401 -0.103724298239892 -0.102504840380083 -0.0245171576687762 0.000899928778773361 0.000899928778773361 0.287690719371942 0.293426382060071 0.802072059140092 brown

ANXA1 -0.629775186384139 0.629775186384139 -0.605594991702291 -0.323648067208467 0.322999540847553 3.68380615008301e-13 3.68380615008301e-13 4.83332006735529e-12 0.000672562520037666 0.000690504051141611 brown

ANXA10 -0.25036407871715 0.25036407871715 -0.237658928245607 -0.166209340666146 0.0602737452098058 0.00929714918349037 0.00929714918349037 0.0137046423291592 0.0870789402880719 0.537423053296125 turquoise

ANXA11 -0.0231034334843954 0.0231034334843954 0.0970840386158248 0.0315947024964849 -0.0861949805188345 0.81327103261344 0.81327103261344 0.319832554387234 0.746649676081276 0.377355224307437 grey

ANXA13 -0.0510840367724841 0.0510840367724841 -0.0432155611309841 0.0391904728537156 0.128694954934724 0.601285890580878 0.601285890580878 0.658502750835928 0.68858068447391 0.186469362381978 grey

ANXA2 -0.0101513619943601 0.0101513619943601 0.204145025101024 0.303507913861088 0.220856819234466 0.917347261421577 0.917347261421577 0.0349311870610934 0.00148320333398496 0.0222522818331953 turquoise

ANXA2P1 -0.0520274078027325 0.0520274078027325 -0.165433700828312 -0.0470519433829198 0.158388910950021 0.594576952815107 0.594576952815107 0.0885839939472653 0.630332341778512 0.10321837574602 pink

ANXA2P2 -0.134614561991263 0.134614561991263 0.0488935356658868 0.216983340818759 0.297634330786832 0.166845964766465 0.166845964766465 0.616992309884763 0.0247714842512417 0.00184900159389156 grey

ANXA2P3 -0.0813637078741188 0.0813637078741188 -0.0360316898089361 0.176972654715202 0.352048197047795 0.404771504448425 0.404771504448425 0.71253275715417 0.0682216848949242 0.000200312082038057 grey

ANXA2R 0.611365817360457 -0.611365817360457 0.70284225919975 0.466981914912909 -0.219905278101878 2.66715168540825e-12 2.66715168540825e-12 3.2200757922436e-17 3.96959799232693e-07 0.0228498597170729 blue

ANXA3 -0.895004552953557 0.895004552953557 -0.695063686857273 -0.346802672779847 0.412545324315095 1.32883694943625e-38 1.32883694943625e-38 9.93783659920204e-17 0.000252734722636722 1.00731585345186e-05 brown

ANXA4 -0.384446791474023 0.384446791474023 -0.28457206059445 -0.0536160511517797 0.318793347747856 4.34526423519769e-05 4.34526423519769e-05 0.00297083557384146 0.583355944824826 0.000817920405298367 yellow

ANXA5 -0.241347026594638 0.241347026594638 -0.0818299161456089 0.155906242459147 0.382258111901724 0.012268445949306 0.012268445949306 0.402075828011575 0.108805170315475 4.84277439214316e-05 brown

ANXA6 0.723032899183617 -0.723032899183617 0.263873500965799 -0.028498364273002 -0.42826797719627 1.44682045752092e-18 1.44682045752092e-18 0.0060267509669099 0.770756037812835 4.18980333898763e-06 blue

ANXA7 -0.154281037926857 0.154281037926857 -0.281043348987647 -0.197435412817895 0.0697760465477347 0.112588232495375 0.112588232495375 0.00336426568944422 0.0415088262791925 0.475126643569094 yellow

ANXA9 -0.416646820257451 0.416646820257451 -0.257434493963234 -0.0963499425233394 0.20723779482357 8.04671447681716e-06 8.04671447681716e-06 0.00743019136951386 0.323522770732835 0.0322083468349742 turquoise

AOAH -0.0999529155261693 0.0999529155261693 0.371809029331467 0.507321015161603 0.325144278460385 0.305672382118669 0.305672382118669 8.04074707611162e-05 2.45143015862197e-08 0.00063278751237082 purple

AOC1 -0.261100322413192 0.261100322413192 -0.0273683657541219 0.151806972978131 0.296890174432924 0.00659942029212497 0.00659942029212497 0.779609596037531 0.118542453100843 0.00190075746315752 grey

AOC2 0.21870784912547 -0.21870784912547 0.47289788930286 0.393361120306431 -0.0136967392249123 0.0236213387382966 0.0236213387382966 2.69753584035308e-07 2.77198244428851e-05 0.888642117096421 black

AOC3 0.268662847832042 -0.268662847832042 0.480098823866332 0.330260604971659 -0.131091598266316 0.00514095737308592 0.00514095737308592 1.6689424450113e-07 0.000512512336008595 0.178331235571769 black

AOC4P 0.17941365852102 -0.17941365852102 0.0313179104541719 -0.0852157364566396 -0.18962936000619 0.0644425925352174 0.0644425925352174 0.748795093999231 0.382819042068788 0.0504309123302617 green

AOX1 -0.126351723941002 0.126351723941002 -0.259602375148113 -0.20771005481768 0.0214777176423035 0.194684403159121 0.194684403159121 0.00692839032048744 0.0318087827717142 0.826195861905319 turquoise

AOX2P -0.042068554207517 0.042068554207517 -0.219833243958773 -0.200955708663552 -0.0243264855086959 0.667021156120995 0.667021156120995 0.0228956521861369 0.037939345068353 0.803580207918588 turquoise

AP000230.1 -0.00213910662097678 0.00213910662097678 -0.287125131024743 -0.264077512388387 -0.0345009933095787 0.982553873750034 0.982553873750034 0.00271252284434808 0.0059864190955241 0.724242612049412 turquoise

AP000253.1 0.0102340026670747 -0.0102340026670747 -0.265634162647093 -0.283757655330415 -0.0988242921150721 0.916676804676394 0.916676804676394 0.00568648811543184 0.00305777011171407 0.311193362646128 turquoise

AP000265.1 -0.142058887743114 0.142058887743114 -0.168747612151115 -0.0548703953673374 0.149899187731087 0.14439385070239 0.14439385070239 0.0822954312491232 0.574565521828555 0.123297773636767 turquoise

AP000347.2 -0.435510349141605 0.435510349141605 -0.298846030724153 -0.148532629134533 0.178354327660632 2.75537920090502e-06 2.75537920090502e-06 0.00176745328856512 0.126793315894573 0.066061157804576 brown

AP000462.1 0.335428153733848 -0.335428153733848 0.189393879264176 0.0306857872778297 -0.220646456859169 0.000412679616497582 0.000412679616497582 0.05072292634214 0.753701855473122 0.0223832255389155 yellow

AP000473.8 0.0708219158029627 -0.0708219158029627 0.0316894216296057 0.0942876095550505 0.114297070705964 0.468517112610046 0.468517112610046 0.745915950401382 0.334035573522523 0.241093670493304 grey

AP000525.9 0.0406834927365667 -0.0406834927365667 -0.0582208900373947 -0.0851439205578394 -0.0605875285520978 0.677364087465848 0.677364087465848 0.551393332714711 0.383221619739647 0.535303214628281 yellow

AP000696.2 -0.601093706752104 0.601093706752104 -0.412997191109351 -0.213460254050664 0.232585960996328 7.62192844062832e-12 7.62192844062832e-12 9.82836685046882e-06 0.0272703712839932 0.0159180501122071 blue

AP001063.1 -0.132131223638468 0.132131223638468 -0.468257437305893 -0.409403496072904 -0.020194647691449 0.174883232771683 0.174883232771683 3.65458826429155e-07 1.19406568149131e-05 0.836429635152028 turquoise

AP001171.1 0.0286402310460363 -0.0286402310460363 -0.244772775666439 -0.219115419696534 -0.0192192757775106 0.76964656025318 0.76964656025318 0.011053969104242 0.0233562855743514 0.844227600937785 green

AP001189.4 0.0162792144992706 -0.0162792144992706 -0.0968997495287129 -0.0806391785777525 0.00274378303030656 0.867821201394656 0.867821201394656 0.320756393348766 0.408981902211444 0.977623387871839 salmon

AP001462.6 0.128190727746018 -0.128190727746018 0.207936058657354 0.164903415684151 -0.0196930742896414 0.188215435616633 0.188215435616633 0.0316190550380166 0.0896248008760507 0.840437717704962 grey

AP001605.4 -0.290386558879318 0.290386558879318 -0.337237678468177 -0.230386980148796 0.094795756495426 0.00241202768259026 0.00241202768259026 0.000382187216524131 0.0169698834649759 0.33142533922365 turquoise

AP001630.5 0.0362708569446654 -0.0362708569446654 -0.115527812618107 -0.120689302442421 -0.0383653786431129 0.710709061696466 0.710709061696466 0.236031239958558 0.215609111867811 0.694808635987651 turquoise

AP006216.10 0.409159756221528 -0.409159756221528 0.249818338599687 0.0972730349063766 -0.194706187163872 1.20983854949891e-05 1.20983854949891e-05 0.00945707643970081 0.318886901697425 0.0444653447351406 brown

AP006222.2 -0.518027929701218 0.518027929701218 -0.274042598582719 -0.0543580824997089 0.302374266926066 1.09988150464387e-08 1.09988150464387e-08 0.0042858567376368 0.578148333859414 0.0015482174014751 brown

AP006547.3 -0.214925413454398 0.214925413454398 -0.308116815986258 -0.240001952705051 0.0365585973645093 0.0262062534140422 0.0262062534140422 0.00124363551773298 0.0127762385968533 0.708517147797615 yellow

AP1AR 0.103796819693502 -0.103796819693502 0.202321553225882 0.187052430754281 0.0259584283568409 0.287351983067745 0.287351983067745 0.0366255012046303 0.053703154725751 0.7906959948305 yellow

AP1B1 0.25062898975176 -0.25062898975176 -0.0634764091386312 -0.0928622698816045 -0.0661118689862635 0.00922037898867098 0.00922037898867098 0.515983953136985 0.341426723440394 0.498675728096665 brown

AP1G1 -0.222762958573179 0.222762958573179 -0.371916110915178 -0.334021122786049 -0.0310506458336944 0.0210954063218493 0.0210954063218493 7.99977124372981e-05 0.000437920158471031 0.75086848532838 brown

AP1G2 0.361105368164568 -0.361105368164568 0.328612351941058 0.150540330925192 -0.217807256493791 0.000132803624975032 0.000132803624975032 0.000548747128548492 0.121683561620359 0.0242161511885437 blue

AP1M1 0.115109236757875 -0.115109236757875 -0.165125843939835 -0.240889116642117 -0.170827773741185 0.237744590472072 0.237744590472072 0.0891870586344558 0.0124393016298129 0.0785338588080937 greenyellow

AP1M2 -0.283384303815821 0.283384303815821 -0.187298033130067 -0.0446344357320536 0.193970042533064 0.00309838295194409 0.00309838295194409 0.0533839355245307 0.648025518024473 0.0452921298237025 turquoise

AP1S1 0.394509497852812 -0.394509497852812 0.0974619951705759 0.0635359116502135 -0.0325626723823359 2.61354458767832e-05 2.61354458767832e-05 0.31794323479499 0.515589813486702 0.739162235415899 brown

AP1S2 0.299263014985861 -0.299263014985861 0.278449212718067 0.202077966776748 -0.0581666054097062 0.00174015271246565 0.00174015271246565 0.00368267071036892 0.0368569742230358 0.551765129571565 yellow

AP1S3 -0.0863446161396905 0.0863446161396905 -0.188589836531701 -0.267744511942623 -0.182594078292455 0.376524506642873 0.376524506642873 0.0517305402144356 0.00530122439798292 0.0597760996381946 grey

AP2A1 0.136701202334781 -0.136701202334781 -0.0962131379492966 -0.132127924330301 -0.0855765209594577 0.160306905951406 0.160306905951406 0.324213482617237 0.174894097061677 0.380800463599378 pink

AP2A2 0.336867161537553 -0.336867161537553 0.112063345388478 0.0256720477818811 -0.117808132629325 0.000388256463829232 0.000388256463829232 0.250473119465639 0.792953006119533 0.226848457765862 blue

AP2B1 0.250263972259901 -0.250263972259901 -0.21500973043616 -0.268196749257992 -0.145321171287249 0.0093263056624189 0.0093263056624189 0.0261461071858602 0.00522175289505458 0.13530683952546 tan

AP2M1 0.0297987800857797 -0.0297987800857797 -0.344551058245144 -0.387218343435952 -0.160680949323109 0.760603635947459 0.760603635947459 0.000278909546598138 3.78372818490424e-05 0.098262510156906 pink

AP2S1 -0.123518141364268 0.123518141364268 -0.352820504210739 -0.219492151394827 0.135711313449047 0.204964207486933 0.204964207486933 0.000193505838746078 0.0231135548388834 0.163384782479558 tan

AP3B1 -0.271240694783045 0.271240694783045 -0.216467241139177 -0.0969593339444694 0.147218623456966 0.00471388874698951 0.00471388874698951 0.0251248531159901 0.32045750956746 0.130225652802175 brown

AP3B2 -0.482430442971553 0.482430442971553 -0.67619605069284 -0.519332840255072 0.0927454083190137 1.42525122793341e-07 1.42525122793341e-07 1.32559300953981e-15 9.95629212919248e-09 0.342037255239473 blue

AP3D1 0.0771022457076189 -0.0771022457076189 0.0628674970993988 0.0364970346853598 -0.0286142382247623 0.429901513425345 0.429901513425345 0.520026170898068 0.708985913159146 0.769849803960776 red

AP3M1 0.509606292986319 -0.509606292986319 0.367213615410323 0.109597472883017 -0.342830699300511 2.07075997604205e-08 2.07075997604205e-08 9.99516653407921e-05 0.261115551324051 0.000300571633579319 turquoise

AP3M2 0.729249780336994 -0.729249780336994 0.610017329901088 0.243378545703378 -0.465514645497655 5.26410667853155e-19 5.26410667853155e-19 3.06795899821817e-12 0.0115349188641469 4.36388241724992e-07 blue

AP3S1 -0.505068544890744 0.505068544890744 -0.484256284896767 -0.305771338296562 0.178614615159798 2.89156453176258e-08 2.89156453176258e-08 1.25850494836728e-07 0.00136075839489928 0.0656604333599686 brown

AP4B1 -0.051010883485627 0.051010883485627 -0.320001841868508 -0.385758800321069 -0.193551875664745 0.601807536408764 0.601807536408764 0.000779271329502278 4.07037803638915e-05 0.0457674463332609 yellow

AP4E1 -0.105651665355781 0.105651665355781 -0.245217542642762 -0.12684266560285 0.137927841480148 0.278778431384652 0.278778431384652 0.0109042830256579 0.19294192177932 0.156553086259134 yellow

AP4M1 -0.129569299756287 0.129569299756287 -0.152150046924517 -0.156505205847109 -0.0463843874846814 0.18346963553987 0.18346963553987 0.117702553512135 0.107436199935977 0.635197738167571 grey

AP4S1 0.496523686159861 -0.496523686159861 0.15379959929637 -0.141223393339857 -0.460977623738483 5.35208129172851e-08 5.35208129172851e-08 0.113728300058844 0.146793615351112 5.83225711009737e-07 blue

AP5B1 -0.752692635650073 0.752692635650073 -0.25304856914837 0.111966892114931 0.554255652158743 8.91535209653391e-21 8.91535209653391e-21 0.00854460149651933 0.250883706505662 5.885912509434e-10 blue

AP5M1 0.0200376765172358 -0.0200376765172358 0.170355815135773 0.13426239427281 -0.017555440645866 0.837683549960155 0.837683549960155 0.0793749756511341 0.167968792922974 0.85756413046905 yellow

AP5S1 0.011772501697534 -0.011772501697534 -0.146050069464908 -0.0854969409077068 0.0652721208784917 0.904206258054864 0.904206258054864 0.133337355513461 0.381245156858131 0.50415749183657 tan

APAF1 -0.620203774769697 0.620203774769697 -0.364365993522924 -0.126498955192751 0.310063459482932 1.0479023868509e-12 1.0479023868509e-12 0.000114191917030701 0.194160650348996 0.00115347919767155 brown

APBA1 0.00462985629301791 -0.00462985629301791 -0.237155952534759 -0.197695622506258 0.0061446702561869 0.9622507423744 0.9622507423744 0.0139114289739346 0.0412357203441358 0.949913826776383 yellow

APBA2 0.808331417857343 -0.808331417857343 0.617998539185278 0.237373705990495 -0.487191146526171 6.63154465813979e-26 6.63154465813979e-26 1.32662382844762e-12 0.0138215738381285 1.02880979992686e-07 blue

APBA3 0.524660551326594 -0.524660551326594 0.520593478064138 0.345875060192124 -0.162912548251761 6.60113735301392e-09 6.60113735301392e-09 9.03947682572005e-09 0.000263230230551422 0.0936192039826927 blue

APBB1 0.744171495338638 -0.744171495338638 0.480239656431387 0.17121924419813 -0.401049447320566 4.13216191849712e-20 4.13216191849712e-20 1.65316151234001e-07 0.077841623973507 1.86143977237498e-05 brown

APBB1IP -0.468536351983796 0.468536351983796 -0.133408791138442 0.056488105546876 0.287896251609039 3.58894775703647e-07 3.58894775703647e-07 0.170713553492651 0.563320734814716 0.00263858166607064 brown

APBB2 -0.298006613052344 0.298006613052344 -0.587184076199631 -0.437277109491995 0.103761292671504 0.00182359134495761 0.00182359134495761 2.9804366344461e-11 2.48393429288949e-06 0.287517891038829 turquoise

APC -0.342605735248162 0.342605735248162 0.062577834177591 0.168598035160648 0.195863502056396 0.000303516351506978 0.000303516351506978 0.521954710219775 0.082571367718102 0.0431909320086133 brown

APC2 -0.0762277584791039 0.0762277584791039 -0.286023613876502 -0.196532452873727 0.0784783361376495 0.435166631303106 0.435166631303106 0.00282138257936907 0.0424682725709636 0.42169062605131 green

APCDD1 -0.179990356122601 0.179990356122601 -0.0541832972797519 0.043581197349772 0.151933807559821 0.0635751021571255 0.0635751021571255 0.579373042869554 0.655796393670735 0.118231403838023 brown

APCDD1L 0.0315288629007785 -0.0315288629007785 -0.126742159840375 -0.064903635444986 0.0724011189977073 0.747159824760265 0.747159824760265 0.193297720613078 0.506572768350852 0.458632879574868 green

APCDD1L-AS1 0.00529693760751592 -0.00529693760751592 -0.205756283901737 -0.227574636037714 -0.0897440112282331 0.956816642245495 0.956816642245495 0.0334894181826635 0.0184023036688637 0.357953125819951 turquoise

APCS -0.0633200515555368 0.0633200515555368 -0.209178100718906 -0.190954881400863 -0.0227052487057925 0.517020386389018 0.517020386389018 0.0305933648259603 0.0488129588718512 0.816432207326556 turquoise

APEH 0.116076691938606 -0.116076691938606 -0.0720921431527743 -0.0835017108377344 -0.0378300061156739 0.233797592486277 0.233797592486277 0.460557643654047 0.392496938663427 0.698860638615962 red

APELA -0.294495673075017 0.294495673075017 -0.422039637785606 -0.305814369944873 0.0889607034643749 0.0020763154243388 0.0020763154243388 5.96211085991059e-06 0.00135852223872087 0.36218125539046 turquoise

APEX1 0.562854270185353 -0.562854270185353 0.440873419181067 0.141272301644955 -0.39516305885468 2.78711859694275e-10 2.78711859694275e-10 2.00757446213351e-06 0.146652314255785 2.52720626143747e-05 red

APEX2 0.626253294300972 -0.626253294300972 0.248493888638685 0.0146504494292586 -0.332937988379436 5.43465509569982e-13 5.43465509569982e-13 0.00985529725122506 0.880943338103483 0.000458309503365682 brown

APH1A 0.581012695248286 -0.581012695248286 0.408945230903451 0.242390315327135 -0.177682619142076 5.34739411278348e-11 5.34739411278348e-11 1.22388282092709e-05 0.0118868071717631 0.0671044661921713 blue

APH1B -0.641054237186939 0.641054237186939 -0.541614829080941 -0.206675254489547 0.429280768499715 1.02505204215069e-13 1.02505204215069e-13 1.70086391216593e-09 0.032689818376259 3.95359492569261e-06 brown

API5 0.0118900065881041 -0.0118900065881041 -0.0831504144795342 -0.154706988257787 -0.142681595646231 0.903254721217508 0.903254721217508 0.394498362875976 0.11158698525575 0.142624629554314 yellow

APIP -0.0815807162827663 0.0815807162827663 -0.0571728876897177 -0.0867922709829555 -0.0648923290116892 0.403515411033188 0.403515411033188 0.558592494940589 0.374045952012779 0.506646972250112 yellow

APLF -0.193151229357775 0.193151229357775 -0.193252742442931 -0.0914583780420279 0.12312419738959 0.0462267258088858 0.0462267258088858 0.0461099963796771 0.348806698635653 0.206423610257619 grey

APLN 0.225806393917329 -0.225806393917329 0.0872176269389439 0.0450599030223161 -0.0491503797166438 0.0193553623084441 0.0193553623084441 0.371700101743496 0.644897012439347 0.615141524256688 green

APLNR -0.0619699418790134 0.0619699418790134 -0.183597099372538 -0.136224081508585 0.0332932519137089 0.526013732844331 0.526013732844331 0.0583630544886829 0.161784988326014 0.733527109576996 turquoise

APLP1 -0.208252282426563 0.208252282426563 -0.23747513290065 -0.158215541271254 0.0735676480295382 0.0313551948757397 0.0313551948757397 0.0137798931226838 0.103601063452385 0.451406207876358 turquoise

APLP2 -0.346533741849451 0.346533741849451 0.00691946154613252 0.227980146689902 0.376721588050032 0.000255737028914686 0.000255737028914686 0.943608175189191 0.0181895363811459 6.34894980546144e-05 purple

APMAP -0.694989684142693 0.694989684142693 -0.537413204800876 -0.358452228868889 0.165797430549083 1.00432332324289e-16 1.00432332324289e-16 2.39700521133931e-09 0.000149986083947587 0.0878756587089558 brown

APOA1 -0.326121267745225 0.326121267745225 -0.48417998725518 -0.3280903530383 0.140648803682917 0.000607991816208536 0.000607991816208536 1.26508376828641e-07 0.0005607039202257 0.148461357481529 turquoise

APOA1BP 0.357710222569612 -0.357710222569612 0.358818856807554 0.151114424053141 -0.260325518938624 0.000155148161096743 0.000155148161096743 0.000147494732092915 0.120252027876881 0.00676780779812792 red

APOA2 -0.439236350671966 0.439236350671966 -0.25297489666112 -0.0289495953917845 0.315136620872474 2.21256017811034e-06 2.21256017811034e-06 0.00856451535282564 0.767228772040016 0.000945788912548123 blue

APOA4 0.163498301150141 -0.163498301150141 -0.117832586237491 -0.168306676623517 -0.115812283264852 0.0924296258825681 0.0924296258825681 0.226751364409757 0.0831109818047911 0.234871745170403 green

APOA5 -0.247215481713739 0.247215481713739 -0.407537865422586 -0.310211078275372 0.0606233629142579 0.0102535649604499 0.0102535649604499 1.319890553666e-05 0.00114689146107489 0.53506139154655 turquoise

APOB -0.232513746129781 0.232513746129781 -0.215028005111908 -0.07382842534019 0.184379421111601 0.0159516735985279 0.0159516735985279 0.0261330867432683 0.449799415624318 0.0572800133667832 blue

APOBEC1 -0.167960382430595 0.167960382430595 -0.443989845924101 -0.385435697874048 -0.014483130296334 0.0837559985599538 0.0837559985599538 1.66603058299087e-06 4.13652169094958e-05 0.882293232627347 turquoise

APOBEC2 -0.0836331860058461 0.0836331860058461 -0.258328949068309 -0.230892674122169 -0.0196765608471618 0.391749454596243 0.391749454596243 0.00721938424758175 0.0167228162295436 0.84056974688279 green

APOBEC3B -0.321530051028695 0.321530051028695 -0.477802125648788 -0.400887814734204 0.00799171918181713 0.000732791072504577 0.000732791072504577 1.94746226096307e-07 1.87727945468815e-05 0.934887413227115 magenta

APOBEC3C 0.643831632045236 -0.643831632045236 0.526903795137323 0.296531150832955 -0.255692042288425 7.41987030994831e-14 7.41987030994831e-14 5.54044236182793e-09 0.00192619338143657 0.00785653503251419 blue

APOBEC3D -0.135922505771157 0.135922505771157 -0.0255376150825176 0.0947271489129961 0.197439344069374 0.162724462037029 0.162724462037029 0.794013082962084 0.331776999588571 0.04150468898773 turquoise

APOBEC3F 0.0330562611081642 -0.0330562611081642 -0.102199824779852 -0.0604039588793362 0.0446965305768782 0.735353540003276 0.735353540003276 0.294872750241706 0.536542859240929 0.647568544496229 green

APOBEC3G 0.352603608612809 -0.352603608612809 0.343597605727376 0.201767507206312 -0.152495290829358 0.000195395356690673 0.000195395356690673 0.000290730063235787 0.0371537736387161 0.116861995323408 blue

APOBEC4 0.0774255916020958 -0.0774255916020958 -0.111419132348614 -0.118695538447127 -0.0408994696567274 0.427963984287707 0.427963984287707 0.253224217164387 0.223343691319842 0.675747270349995 yellow

APOBR -0.214406288386039 0.214406288386039 0.111053379765611 0.255535666607056 0.273525007017394 0.0265791658906934 0.0265791658906934 0.254795353517514 0.00789583306441293 0.00436220811900127 purple

APOC1 -0.310677959007301 0.310677959007301 -0.234153486825321 -0.0445122731752891 0.261640811230191 0.00112628110471858 0.00112628110471858 0.0152031915796266 0.648924928296073 0.00648416517338928 turquoise

APOC2 0.185325975182969 -0.185325975182969 -0.0745335859825645 -0.147497948626297 -0.142860809121754 0.0559916922764448 0.0559916922764448 0.445470576536186 0.1294901386881 0.142118504916316 green

APOC3 -0.146347184362808 0.146347184362808 -0.255211722992151 -0.16859108653231 0.081506931730223 0.13254085316459 0.13254085316459 0.0079777942827 0.0825842043461334 0.403942234219502 yellow

APOD -0.0504821750803214 0.0504821750803214 -0.225050390215801 -0.139494232904277 0.0874320312875915 0.605583646531963 0.605583646531963 0.0197756333193034 0.151855553848322 0.370521072359712 turquoise

APOE -0.205653316404456 0.205653316404456 -0.307453520257497 -0.136504254103875 0.211149319203049 0.0335800293464605 0.0335800293464605 0.00127579047465684 0.160915811878932 0.0290233797542365 green

APOF -0.0746276634649387 0.0746276634649387 -0.207461645523861 -0.1869992944408 -0.0184674762031246 0.444894828523296 0.444894828523296 0.0320184285836813 0.0537724243857851 0.85024846364071 turquoise

APOH -0.137056568892856 0.137056568892856 -0.231315997669912 -0.093514707476791 0.174440710700337 0.159212562960605 0.159212562960605 0.0165183922568574 0.338030772461594 0.0723293172089099 turquoise

APOL1 0.331865465935857 -0.331865465935857 0.50755750473917 0.558311489335767 0.216177084112692 0.000479356012278583 0.000479356012278583 2.40912944958937e-08 4.14792121196716e-10 0.0253254032548942 cyan

APOL2 0.496115794302467 -0.496115794302467 0.427049807313264 0.353083008448687 -0.0160006360379395 5.50940518659221e-08 5.50940518659221e-08 4.49156947157114e-06 0.000191241631859421 0.87006312600461 cyan

APOL3 0.69262499676427 -0.69262499676427 0.795341831700426 0.608861297522877 -0.112442119972055 1.40460317264555e-16 1.40460317264555e-16 1.44272221600502e-24 3.45736659633263e-12 0.248865208059582 blue

APOL4 0.0070174764462107 -0.0070174764462107 -0.134154939536901 -0.00689356970385214 0.181466912374662 0.942810719320813 0.942810719320813 0.168312505438196 0.943818842306788 0.0613972860327662 yellow

APOL5 -0.141104524199653 0.141104524199653 -0.268161313764695 -0.105820873471486 0.206618568417203 0.147137468274811 0.147137468274811 0.0052279414346264 0.27800493861316 0.0327386704815724 turquoise

APOL6 0.113117788463139 -0.113117788463139 0.331263167121164 0.513339981451515 0.393732030273544 0.24601466542341 0.24601466542341 0.000491561153182244 1.56756153574595e-08 2.71985097293864e-05 cyan

APOLD1 -0.0453022292714241 0.0453022292714241 0.0313016508557244 0.00548575091775791 -0.0357642295385475 0.643117923316407 0.643117923316407 0.748921182113178 0.955278901526564 0.714574113421367 grey

APOM -0.0817720156778843 0.0817720156778843 -0.202840327953663 -0.149978811343499 0.0376702779884583 0.402410037579598 0.402410037579598 0.0361365910646365 0.12309641295458 0.700071197430836 turquoise

APOO -0.327632060937114 0.327632060937114 -0.521716629296953 -0.420553547216118 0.0378650962932299 0.000571398104992512 0.000571398104992512 8.291256516174e-09 6.47900175643545e-06 0.69859479565756 magenta

APOOL 0.467521137900023 -0.467521137900023 0.261599676271731 0.0868639665270402 -0.229324376292128 3.83340105766913e-07 3.83340105766913e-07 0.00649287372521406 0.373649917753665 0.0174993459524227 turquoise

APOPT1 -0.673119127442761 0.673119127442761 -0.553303159862332 -0.287132967660744 0.30964300647937 1.98636087913117e-15 1.98636087913117e-15 6.38574637504674e-10 0.00271176211465413 0.00117243196146491 blue

APP -0.0489283059001074 0.0489283059001074 -0.204713062605924 -0.173310770679515 0.000792546936546116 0.616741619248033 0.616741619248033 0.0344170522256251 0.0742257513469319 0.993535707478637 salmon

APPBP2 0.284553708266417 -0.284553708266417 0.482195892484655 0.386202105138742 -0.0392268725408357 0.00297276987429286 0.00297276987429286 1.44813912392435e-07 3.98123562212372e-05 0.688306408396556 tan

APPL1 0.289452266893407 -0.289452266893407 0.231298907351767 0.0784163836027706 -0.200024877260122 0.00249488182988603 0.00249488182988603 0.0165266031148717 0.422058325841191 0.0388571923595495 yellow

APPL2 -0.258964794262817 0.258964794262817 -0.396426594884678 -0.406436946751385 -0.11858667292945 0.00707276130687768 0.00707276130687768 2.36781621025245e-05 1.39988227352801e-05 0.223771583813026 brown

APRT 0.68594302864781 -0.68594302864781 0.587389130099853 0.296494282892822 -0.342842735396185 3.56256533670509e-16 3.56256533670509e-16 2.92249020142074e-11 0.00192882274560913 0.00030041482999859 red

APTR -0.0644884404639737 0.0644884404639737 0.0118372944051414 -0.00134594267842344 -0.0193264833654564 0.509301374286116 0.509301374286116 0.903681559648246 0.989022239520716 0.843369740030215 green

APTX -0.10120726345689 0.10120726345689 -0.0883056871833405 -0.0852567232661667 -0.0174620908018844 0.299611965453254 0.299611965453254 0.36574046642925 0.382589397251308 0.858313604725162 yellow

AQP1 -0.273900215901692 0.273900215901692 -0.521096637433509 -0.395119995993894 0.0801110921260783 0.00430674000916659 0.00430674000916659 8.69661549107656e-09 2.5328117492857e-05 0.412066849472241 pink

AQP10 -0.334525023311074 0.334525023311074 -0.433119330797423 -0.332165805545805 0.0602180195127213 0.000428721907426231 0.000428721907426231 3.16763955797606e-06 0.000473374515203208 0.537799955810853 salmon

AQP11 -0.0961861153453695 0.0961861153453695 0.0182511295495391 -0.00861176939369094 -0.0408851221600166 0.32435002876369 0.32435002876369 0.851982709593587 0.92984793776625 0.675854631033736 grey

AQP2 0.0150708698577153 -0.0150708698577153 -0.161492453188875 -0.111542638570969 0.0433294712865506 0.877552989866438 0.877552989866438 0.0965534690741373 0.252695185856404 0.657659138399577 green

AQP3 0.622079648206565 -0.622079648206565 0.454696544335095 0.0908544586633157 -0.500581189925088 8.56165291700771e-13 8.56165291700771e-13 8.65472271857224e-07 0.352011872499674 4.00373459895258e-08 red

AQP4 -0.218719924112801 0.218719924112801 -0.328422121043853 -0.21662591066899 0.105443519453494 0.0236134492575457 0.0236134492575457 0.000553077137728502 0.0250157584562139 0.279731896078745 turquoise

AQP4-AS1 -0.186826434894539 0.186826434894539 -0.16050831322713 -0.0602596030164652 0.128895237385277 0.0539982766474578 0.0539982766474578 0.0986291383303223 0.53751869208165 0.185779093893499 turquoise

AQP5 -0.31573101715832 0.31573101715832 -0.503181986241545 -0.357943715526142 0.11737305487179 0.000923833932692318 0.000923833932692318 3.31740946406924e-08 0.000153506265710381 0.228580826599281 turquoise

AQP6 -0.159314757228091 0.159314757228091 -0.359035784976984 -0.260627912982831 0.0748885290265915 0.101193429106601 0.101193429106601 0.000146038776135228 0.00670164115410821 0.443300538383949 green

AQP8 -0.23235722481626 0.23235722481626 -0.402772326069124 -0.252789719973378 0.151156016838457 0.0160247617841015 0.0160247617841015 1.70021865596148e-05 0.00861474975254258 0.12014882095903 turquoise

AQP9 -0.607839834034301 0.607839834034301 -0.256680462088796 0.0269932923523411 0.415358505110983 3.84083708657023e-12 3.84083708657023e-12 0.00761211138333461 0.782554604473014 8.63767352570554e-06 brown

AQPEP -0.216736279784952 0.216736279784952 -0.216686877892872 -0.173537698808691 0.0176478599058699 0.0249401117877719 0.0249401117877719 0.0249739475295716 0.0738417065515359 0.856822252204983 turquoise

AQR 0.414814855212957 -0.414814855212957 0.615810257773162 0.398073909941742 -0.211471684051581 8.89911403723771e-06 8.89911403723771e-06 1.67339724905782e-12 2.17414567643568e-05 0.0287732482109645 blue

AR -0.0716516938579159 0.0716516938579159 -0.215394703175475 -0.0817630099854264 0.171449301732983 0.463309102061106 0.463309102061106 0.0258729847869 0.402462034440982 0.0774371048078004 turquoise

ARAF -0.119891729573986 0.119891729573986 -0.211165315568514 -0.123516133663739 0.0945409314879486 0.218680065206238 0.218680065206238 0.0290109243913559 0.204971626409396 0.332732687798425 grey

ARAP1 0.206949954526348 -0.206949954526348 0.48776444710421 0.437151475346161 0.0391722662605481 0.03245395230398 0.03245395230398 9.88878712098152e-08 2.50236896628547e-06 0.688717888517967 black

ARAP3 0.158743286244271 -0.158743286244271 0.520527440078214 0.433059986539432 -0.0149403557642999 0.102439592055674 0.102439592055674 9.0854260638594e-09 3.17857781835192e-06 0.878605245721414 black

ARC -0.173664993772253 0.173664993772253 -0.336176649255329 -0.265399056224574 0.0338829484556197 0.0736269792424567 0.0736269792424567 0.000399804856079806 0.00573091359258011 0.728988928400057 green

ARCN1 0.281095126293875 -0.281095126293875 0.197738924429853 0.0883417414406472 -0.134869729763341 0.00335817013559334 0.00335817013559334 0.0411904182515782 0.365543997941301 0.166035885521118 turquoise

AREG -0.265878104291609 0.265878104291609 -0.435835461737807 -0.255093194860225 0.194854033509113 0.00564071701207179 0.00564071701207179 2.70341073073827e-06 0.00800797027683025 0.0443008170333055 blue

AREL1 -0.287930365037724 0.287930365037724 -0.119308850001874 -0.00928103460162087 0.156041715360152 0.00263535294639883 0.00263535294639883 0.220943869205686 0.92441158168423 0.108494354057703 turquoise

ARF1 0.26479942060091 -0.26479942060091 0.20349474278272 0.109872820075698 -0.106637477115212 0.00584561993312743 0.00584561993312743 0.0355276818569543 0.259912130035659 0.274292295299286 brown

ARF3 0.029977222672588 -0.029977222672588 -0.266208717909485 -0.172337622718115 0.090985999588665 0.759213649107687 0.759213649107687 0.00557920642069426 0.0758909516416586 0.351312185982625 greenyellow

ARF4 -0.431864063280242 0.431864063280242 -0.502563609328191 -0.345627148549467 0.137373245576312 3.40676758589461e-06 3.40676758589461e-06 3.46958954965478e-08 0.000266102651195674 0.158242067379824 greenyellow

ARF5 0.0193350697308084 -0.0193350697308084 -0.138911205208547 -0.0537087356270243 0.108910445343439 0.843301040980572 0.843301040980572 0.153591508349585 0.58270430445917 0.264134785336105 grey

ARF6 0.531282446251536 -0.531282446251536 0.610523487217462 0.39942744340578 -0.20156393517418 3.92146828392515e-09 3.92146828392515e-09 2.91112722141797e-12 2.02624129632254e-05 0.0373494751788349 turquoise

ARFGAP1 0.342648394261668 -0.342648394261668 0.169681329850004 0.113335221515634 -0.052079500839033 0.000302955919107694 0.000302955919107694 0.0805895472322081 0.24510216570434 0.594207468852317 brown

ARFGAP2 0.38152981224561 -0.38152981224561 0.155193682567635 0.0617489371310647 -0.118717019751568 5.01979401863602e-05 5.01979401863602e-05 0.110451443526382 0.527493358719459 0.223259327925056 brown

ARFGAP3 -0.373135615665771 0.373135615665771 -0.192608842161528 -0.0742114230251362 0.151450198953721 7.5466376825105e-05 7.5466376825105e-05 0.0468545774941721 0.447445360120686 0.11942079802764 brown

ARFGEF1 -0.0833805210903511 0.0833805210903511 -0.0355511334603995 -0.050223353914411 -0.0339981300203343 0.393186700711763 0.393186700711763 0.716201984721625 0.607435988566432 0.728103601092847 green

ARFGEF2 0.0399438196037043 -0.0399438196037043 -0.0444530401449416 -0.162662243429813 -0.211892114218368 0.682912398827445 0.682912398827445 0.649361207701562 0.0941312133475176 0.0284497760035411 yellow

ARFIP1 0.413938620336316 -0.413938620336316 0.648162252017532 0.531831540095051 -0.0311822420136862 9.33623539834809e-06 9.33623539834809e-06 4.45315440264627e-14 3.75383561747123e-09 0.749847362951655 blue

ARFIP2 0.492517836273265 -0.492517836273265 0.369508600900132 0.173078934957865 -0.238462138060545 7.10201233614642e-08 7.10201233614642e-08 8.96967287157934e-05 0.0746197613727169 0.0133799686095081 red

ARFRP1 0.557184536065849 -0.557184536065849 0.325348238865741 0.188282061632538 -0.149092528095699 4.5736767495329e-10 4.5736767495329e-10 0.000627535753882668 0.052120582182776 0.125352058152135 brown

ARG1 -0.797154890817221 0.797154890817221 -0.697568457892262 -0.404116144136901 0.318940557801177 9.5136601885811e-25 9.5136601885811e-25 6.94058276060273e-17 1.58367308163379e-05 0.000813120474888094 brown

ARG2 -0.46534685276318 0.46534685276318 -0.601710875822017 -0.451537918208979 0.100489071879652 4.41127313224671e-07 4.41127313224671e-07 7.1634885309881e-12 1.05237811044708e-06 0.30307219252028 magenta

ARGLU1 0.0308774151544971 -0.0308774151544971 0.197444468163826 0.155003555361888 -0.0213785574356198 0.752213326907513 0.752213326907513 0.0414992968521053 0.110893967616203 0.826985750744098 yellow

ARHGAP1 -0.0882444129394627 0.0882444129394627 -0.197081791788529 -0.164390155382544 0.00493545467601055 0.366074513782511 0.366074513782511 0.0418823913709801 0.0906414120467845 0.959761082811597 yellow

ARHGAP10 0.471150568794916 -0.471150568794916 0.299447116055121 0.113135415795394 -0.239258144368561 3.02587215784261e-07 3.02587215784261e-07 0.00172822140914542 0.245940601608985 0.0130648377004217 brown

ARHGAP11A -0.0336953695238056 0.0336953695238056 -0.338212561654256 -0.402301915896826 -0.195391012793719 0.730431490408261 0.730431490408261 0.000366633174478922 1.74288535533371e-05 0.0437075045412959 magenta

ARHGAP12 0.141179799143078 -0.141179799143078 0.419687086233383 0.417190081363095 0.103334409637035 0.146919649932841 0.146919649932841 6.79957776703624e-06 7.80911704053397e-06 0.289516377192965 tan

ARHGAP17 0.829741953818448 -0.829741953818448 0.649643771083039 0.396127742578421 -0.263486890933358 2.38873882870738e-28 2.38873882870738e-28 3.73243877929842e-14 2.40464068119749e-05 0.00610384385290186 blue

ARHGAP18 -0.52379685670956 0.52379685670956 -0.433815719946614 -0.207392437781295 0.272852760461697 7.05930409965673e-09 7.05930409965673e-09 3.04191214245668e-06 0.0320770441641439 0.00446318625248498 brown

ARHGAP19 -0.185978730690279 0.185978730690279 -0.127906409667374 -0.0517958244604464 0.0963100083910997 0.0551171820971698 0.0551171820971698 0.189205218864491 0.596220771375134 0.32372429620737 brown

ARHGAP20 -0.215638051580856 0.215638051580856 -0.298771694377599 -0.195584843373998 0.09844056849002 0.0257015963564022 0.0257015963564022 0.00177236075777339 0.0434949708144625 0.313085130783408 turquoise

ARHGAP21 -0.14478404757703 0.14478404757703 -0.207925973135601 -0.124409590459488 0.0883614311025289 0.136772267582297 0.136772267582297 0.0316275012851557 0.201689080587732 0.36543673156634 salmon

ARHGAP22 0.187336945233025 -0.187336945233025 -0.0788590178223675 -0.159327232726385 -0.156696974054251 0.0533335035460998 0.0533335035460998 0.419435277708034 0.101166358089729 0.107000754251847 yellow

ARHGAP22-IT1 -0.251723015074374 0.251723015074374 -0.24454410598509 -0.126237581855346 0.137984562881475 0.00890920942582146 0.00890920942582146 0.01113162575387 0.19509114872886 0.156381107858482 yellow

ARHGAP23 -0.0346331710632279 0.0346331710632279 -0.236409658866938 -0.19585209683386 0.00819703272699187 0.723228891860512 0.723228891860512 0.0142232515548314 0.0432033411795888 0.933218426968026 green

ARHGAP24 -0.545901843965357 0.545901843965357 -0.363310915494234 -0.0905045542763803 0.369595628476018 1.19265642103261e-09 1.19265642103261e-09 0.000119930867800048 0.353877297687832 8.93278469277557e-05 brown

ARHGAP25 0.360323632051882 -0.360323632051882 0.657046680962093 0.51646603596843 -0.0700361648779529 0.000137666190609372 0.000137666190609372 1.52207607881644e-14 1.23843287910155e-08 0.473478089322406 black

ARHGAP26 -0.281291986878717 0.281291986878717 0.0508502965608092 0.233131978803546 0.322207172994338 0.00333508483722876 0.00333508483722876 0.602953364080921 0.0156658044174227 0.000713019654407843 purple

ARHGAP26-AS1 -0.0342428289697026 0.0342428289697026 -0.257175327070222 -0.211251862783365 0.0119758732158328 0.726223943775667 0.726223943775667 0.00749228055994665 0.0289436141330464 0.902559472770454 green

ARHGAP27 -0.329028924056194 0.329028924056194 0.0140922812638327 0.219289368526955 0.35165326487342 0.000539373659946763 0.000539373659946763 0.88544782458698 0.0232439395111516 0.000203877595366882 purple

ARHGAP28 -0.279226264321156 0.279226264321156 -0.34882102507434 -0.213295577188092 0.140463000614379 0.00358457406278623 0.00358457406278623 0.000231220727831269 0.0273922387756859 0.149003689745167 turquoise

ARHGAP29 -0.541132816234604 0.541132816234604 -0.559047315520621 -0.283749751874571 0.323651958010524 1.76956688118134e-09 1.76956688118134e-09 3.89078817398319e-10 0.00305862486113468 0.000672456181782445 brown

ARHGAP30 0.310029548687958 -0.310029548687958 0.293560412484164 0.195892054795865 -0.0904161410250824 0.00115499738403589 0.00115499738403589 0.00214877607328995 0.0431598789230692 0.354349622858486 black

ARHGAP31 0.182710009091875 -0.182710009091875 0.355223338601161 0.457633404361018 0.264747968145247 0.059611361587793 0.059611361587793 0.000173675539249188 7.20325751732658e-07 0.0058555568281726 cyan

ARHGAP32 0.560315841043363 -0.560315841043363 0.155348309938807 -0.0425093261304396 -0.295775408300678 3.48308808295094e-10 3.48308808295094e-10 0.110092560550998 0.663742620728435 0.00198074518613183 brown

ARHGAP33 0.0991054029425327 -0.0991054029425327 -0.255323092813204 -0.317307180930805 -0.170574835727618 0.309812204701462 0.309812204701462 0.00794953221384473 0.000867850125504157 0.0789837473121978 green

ARHGAP35 0.621138772561503 -0.621138772561503 0.486709677761937 0.190290902003272 -0.378017126981692 9.47654386952162e-13 9.47654386952162e-13 1.0635283195895e-07 0.0496179714698355 5.9617140753158e-05 brown

ARHGAP36 -0.194142689970698 0.194142689970698 -0.443021865377786 -0.367113632322592 0.0151997658462199 0.0450970869339277 0.0450970869339277 1.76573056418773e-06 0.000100422308688678 0.876513989063111 turquoise

ARHGAP39 0.239071089869725 -0.239071089869725 0.0282031919518847 -0.0614222345667651 -0.144787784092952 0.0131383033023006 0.0131383033023006 0.773065930304496 0.52968445665232 0.136762031769794 yellow

ARHGAP4 0.169410264675865 -0.169410264675865 0.0203398323245836 -0.0844316855429305 -0.172492993050901 0.0810818361670486 0.0810818361670486 0.835270237892238 0.387228010318628 0.0756230926455838 grey

ARHGAP42 -0.279335846602287 0.279335846602287 -0.395531783352774 -0.35187837683738 -0.0273364779473968 0.00357093029258454 0.00357093029258454 2.47968329102297e-05 0.000201838112099836 0.779859852740389 blue

ARHGAP44 -0.121027904767431 0.121027904767431 -0.312377455718701 -0.260462672299221 0.00798910293533221 0.214314668427748 0.214314668427748 0.00105407523180226 0.00673772598860382 0.934908682434175 turquoise

ARHGAP5 0.56611917054599 -0.56611917054599 0.601265222317627 0.321781575538055 -0.319931288098676 2.08656427069538e-10 2.08656427069538e-10 7.49175825331835e-12 0.000725388903020134 0.000781480965071155 turquoise

ARHGAP5-AS1 -0.170662453647181 0.170662453647181 -0.302197467155708 -0.205071984106117 0.0872816346809823 0.0788276722675304 0.0788276722675304 0.00155858674178822 0.0340954866251644 0.371347877346398 turquoise

ARHGAP6 -0.274460348713056 0.274460348713056 -0.359864124767664 -0.259103208293305 0.0786672895616572 0.00422510661720476 0.00422510661720476 0.000140601032487588 0.0070411951599279 0.420570296661779 salmon

ARHGAP9 0.0139651726765536 -0.0139651726765536 0.327658347016379 0.272019257203131 -0.0103893611547007 0.886474122259564 0.886474122259564 0.000570779698227277 0.00459128238405048 0.915416552970106 black

ARHGDIA 0.110235543416961 -0.110235543416961 -0.116317856646742 -0.168715782718128 -0.118687127293697 0.258332610264089 0.258332610264089 0.23282086447449 0.0823540877066431 0.223376730513977 grey

ARHGDIB -0.300727300108303 0.300727300108303 -0.421199056835328 -0.300041993211042 0.0975411630707674 0.00164728410573332 0.00164728410573332 6.24949536349645e-06 0.00169017345571047 0.3175484072455 brown

ARHGDIG -0.159760033021891 0.159760033021891 -0.25059025571251 -0.153898039269088 0.099774148068358 0.100230724970324 0.100230724970324 0.00923156904368454 0.113494464122615 0.306542579696855 green

ARHGEF1 -0.241320619866885 0.241320619866885 -0.0478734791900739 0.0665463720923595 0.181800746933355 0.0122782428754091 0.0122782428754091 0.624366318855082 0.49585165996685 0.0609134395329288 turquoise

ARHGEF10 0.152237446934733 -0.152237446934733 -0.0120723543424815 -0.165223766886646 -0.262859242561321 0.117489321768669 0.117489321768669 0.901778368301288 0.0889948836817521 0.00623086681372721 brown

ARHGEF10L 0.625192451177559 -0.625192451177559 0.530733987343343 0.324222295304311 -0.214239016634266 6.10411149132296e-13 6.10411149132296e-13 4.09606130468197e-09 0.00065703406893774 0.0267002835371107 brown

ARHGEF11 -0.281357262278493 0.281357262278493 -0.0481431198535343 0.173668073654134 0.363881478549467 0.00332746167685691 0.00332746167685691 0.622413432323604 0.0736217901907124 0.000116794914425258 blue

ARHGEF12 -0.21895527119488 0.21895527119488 -0.49011980651878 -0.412338535264283 0.00630504426211737 0.0234601290290906 0.0234601290290906 8.39808019590483e-08 1.01870869574832e-05 0.948608353632684 pink

ARHGEF15 -0.121200443929524 0.121200443929524 -0.31531656640837 -0.235591973966075 0.0544048799457148 0.213657196746363 0.213657196746363 0.000939092579801205 0.0145718643279294 0.577820630901033 turquoise

ARHGEF16 -0.186258868445678 0.186258868445678 -0.342906466821088 -0.249236818044509 0.0709859861235533 0.0547453328886492 0.0547453328886492 0.000299585808740375 0.00963014558109212 0.467484816953371 turquoise

ARHGEF17 -0.216027959577148 0.216027959577148 -0.281418816774243 -0.165773428616802 0.124019590867241 0.0254290049762144 0.0254290049762144 0.00332028739307113 0.08792226142113 0.20311725599086 yellow

ARHGEF18 0.795151768693943 -0.795151768693943 0.763014977183393 0.40075317798937 -0.418875957789459 1.50672592766307e-24 1.50672592766307e-24 1.27702434754214e-21 1.89057009822875e-05 7.11318196355486e-06 blue

ARHGEF19 0.556839267604851 -0.556839267604851 0.336699342919584 0.155465119584103 -0.221097964947844 4.71231958211818e-10 4.71231958211818e-10 0.00039103451528448 0.109822053382589 0.022102984709765 brown

ARHGEF2 0.26230865678094 -0.26230865678094 0.320591226403727 0.190508856912763 -0.138466302326786 0.00634421939168463 0.00634421939168463 0.000761034813512997 0.0493525202764728 0.154926170874897 yellow

ARHGEF25 0.184428509638004 -0.184428509638004 -0.0437904791731579 -0.158446991946775 -0.205696409931015 0.0572126085312473 0.0572126085312473 0.6542493356934 0.103090418618589 0.03354208202319 green

ARHGEF26 -0.167079922589304 0.167079922589304 -0.0322361914085559 0.0913417960439273 0.20134210556253 0.0854139659955697 0.0854139659955697 0.741684948020717 0.349424005268042 0.037563712271962 turquoise

ARHGEF26-AS1 -0.0857168721027468 0.0857168721027468 -0.251779518465913 -0.154248115855058 0.100892702085199 0.380016947106537 0.380016947106537 0.00889339247434116 0.112665909440095 0.301124303701205 turquoise

ARHGEF28 -0.399405457067832 0.399405457067832 -0.30340878305059 -0.0595019660454731 0.33593162731547 2.02856646565332e-05 2.02856646565332e-05 0.0014887878864547 0.542654614930631 0.00040397814540421 turquoise

ARHGEF3 0.618828358502023 -0.618828358502023 0.698774603040498 0.487916781957117 -0.17854029668228 1.21422472094005e-12 1.21422472094005e-12 5.83114366288635e-17 9.78519629550082e-08 0.0657746480640128 turquoise

ARHGEF33 -0.216251285292731 0.216251285292731 -0.405843391836084 -0.439290805163518 -0.160752616428325 0.0252739875337984 0.0252739875337984 1.4448782417466e-05 2.20543430176115e-06 0.0981106258470889 turquoise

ARHGEF37 -0.140800150260364 0.140800150260364 -0.405652559908746 -0.277993313378416 0.112554412113349 0.148020697908731 0.148020697908731 1.45963113826321e-05 0.00374133773430263 0.248389894130553 green

ARHGEF38 -0.170949099488125 0.170949099488125 -0.183072831207358 -0.112008966685097 0.073610447769045 0.0783187932239357 0.0783187932239357 0.0590981875473386 0.250704544506183 0.451142275844063 turquoise

ARHGEF39 -0.013102762556632 0.013102762556632 -0.229823766788739 -0.237321499870239 -0.0716231086907291 0.893442248787974 0.893442248787974 0.017248763326029 0.0138430703367272 0.463487983211831 turquoise

ARHGEF4 -0.24904706236143 0.24904706236143 -0.270727351479415 -0.112451327987454 0.199067096935487 0.00968721877766202 0.00968721877766202 0.00479631866948123 0.248826208487079 0.0398209473373523 turquoise

ARHGEF40 -0.107096834131989 0.107096834131989 0.117805689267327 0.174583677659683 0.126497668346632 0.272218592481861 0.272218592481861 0.226858160770761 0.0720921807319171 0.194165223688522 black

ARHGEF5 -0.00343717861195552 0.00343717861195552 -0.239636204414363 -0.269527168640732 -0.112120003984235 0.9719705406896 0.9719705406896 0.0129174472546775 0.00499407428913156 0.25023214808861 turquoise

ARHGEF6 0.329552701832209 -0.329552701832209 0.253619847545336 0.083892059222993 -0.222875579219873 0.000527796593185517 0.000527796593185517 0.00839156342942583 0.390280157214218 0.0210287010488115 turquoise

ARHGEF7 0.337271429795591 -0.337271429795591 0.0921681974498253 -0.135849479278506 -0.363124475500618 0.000381638718937964 0.000381638718937964 0.345062924384622 0.16295256413816 0.000120972429988016 grey

ARHGEF7-AS2 -0.0934429860859788 0.0934429860859788 -0.267198573592586 -0.262784730146683 -0.0609989846644241 0.338403034130991 0.338403034130991 0.00539859510826881 0.00624610125698278 0.532529833472882 turquoise

ARHGEF7-IT1 -0.226784144349649 0.226784144349649 -0.28995059803004 -0.183542432763361 0.106165276758 0.0188232445087865 0.0188232445087865 0.0024503752605734 0.0584393570901729 0.276435036852949 turquoise

ARHGEF9 0.801370082812628 -0.801370082812628 0.577205403175111 0.218759923485641 -0.460027691027913 3.55422018153398e-25 3.55422018153398e-25 7.62303471973284e-11 0.023587330808843 6.19414160864342e-07 blue

ARID1A 0.650737076510559 -0.650737076510559 0.59853925630951 0.356662749094997 -0.256843278538654 3.27444429908403e-14 3.27444429908403e-14 9.83920085131245e-12 0.000162716299144502 0.00757250003297794 black

ARID1B 0.626920157393929 -0.626920157393929 0.717353824430684 0.453328853430905 -0.263956737151034 5.05079376537089e-13 5.05079376537089e-13 3.55820249758501e-18 9.42166485385948e-07 0.00601026661100589 brown

ARID2 0.307364977221681 -0.307364977221681 0.368787626213854 0.180028029554818 -0.22563748243381 0.00128013957719727 0.00128013957719727 9.28077009908921e-05 0.0635187648400207 0.0194485887616089 turquoise

ARID3A -0.428485338407064 0.428485338407064 -0.304219565890756 -0.130568540296048 0.216560773952671 4.13801295973453e-06 4.13801295973453e-06 0.00144366749988571 0.180084736664416 0.0250604947810103 purple

ARID3B 0.0168340881603183 -0.0168340881603183 0.0257920404227668 0.0380296625204345 0.0273673460052701 0.863358864761881 0.863358864761881 0.792007115576806 0.69734852953208 0.779617598716448 grey

ARID4A -0.380101111454233 0.380101111454233 -0.00745731778669653 0.101476971844095 0.182855514231382 5.38474504581124e-05 5.38474504581124e-05 0.93923284722574 0.298319253106149 0.0594051238416693 green

ARID4B -0.304183488493595 0.304183488493595 0.0793291446438342 0.210601895475 0.242988666650285 0.00144564851121107 0.00144564851121107 0.416659682253909 0.0294523665295943 0.0116726433584497 tan

ARID5A -0.452241851300315 0.452241851300315 -0.416925015729403 -0.19173513517822 0.275089973135322 1.00767496723025e-06 1.00767496723025e-06 7.92420652048673e-06 0.0478808165916026 0.00413499918922274 brown

ARID5B 0.0544409537240349 -0.0544409537240349 -0.000355505052582125 0.0281705231973426 0.0482927678343552 0.577568080124009 0.577568080124009 0.997100350350367 0.773321704254393 0.621330726904004 grey

ARIH1 0.298782242613468 -0.298782242613468 0.372477980321269 0.241183596268555 -0.127223059929818 0.00177166364317214 0.00177166364317214 7.78793610892892e-05 0.0123291885511717 0.191599588518793 turquoise

ARIH2 0.662800971881736 -0.662800971881736 0.432418567731756 0.168114267670709 -0.33746206439419 7.4479492458402e-15 7.4479492458402e-15 3.29910423911894e-06 0.0834688763593517 0.000378554279192812 blue

ARIH2OS -0.184436944269871 0.184436944269871 0.0108244185355851 0.107521510139987 0.166785429447161 0.0572010332498413 0.0572010332498413 0.91188853805551 0.270310884371469 0.0859743169301081 green

ARL1 0.0665444123311625 -0.0665444123311625 0.10466932222249 0.00999538980355299 -0.133751764232815 0.495864378588509 0.495864378588509 0.283297458597494 0.918612808031309 0.169606770715146 yellow

ARL10 0.684123434669595 -0.684123434669595 0.515384158063037 0.193126637162917 -0.414493511401577 4.57059058579047e-16 4.57059058579047e-16 1.34403821149037e-08 0.046255041132248 9.05713323029682e-06 blue

ARL11 -0.719560501881243 0.719560501881243 -0.342767644245773 -0.029137022231849 0.444104363561903 2.51486590991648e-18 2.51486590991648e-18 0.000301394336033937 0.765765051103652 1.65459467500564e-06 brown

ARL13B 0.0617645330400888 -0.0617645330400888 0.0511283444069234 0.0515827459318242 0.0138753550997154 0.5273888756048 0.5273888756048 0.600970036912593 0.597735039649996 0.887199438857587 yellow

ARL14 0.0929351294745669 -0.0929351294745669 -0.033037041547398 -0.133329247839257 -0.178576493649045 0.341046423798098 0.341046423798098 0.735501725306901 0.170971001768998 0.0657189994128485 grey

ARL14EP 0.528513886754288 -0.528513886754288 0.581282233370969 0.321801903158766 -0.291124841939316 4.88202450389356e-09 4.88202450389356e-09 5.2139320758635e-11 0.000724793677088383 0.00234832056195515 turquoise

ARL15 0.0981976388352585 -0.0981976388352585 0.302966576553171 0.294890550790203 0.0639551414842709 0.314286630400875 0.314286630400875 0.00151393296563669 0.00204639059835246 0.512817241798818 turquoise

ARL16 0.0859609338369528 -0.0859609338369528 -0.0138613002767012 0.0201494948752249 0.054134054485272 0.378656789610735 0.378656789610735 0.887312946201972 0.836790282387654 0.579718300580343 grey

ARL2 0.278515911754094 -0.278515911754094 0.16085439638113 0.0290825308030703 -0.182274009833637 0.00367415707972771 0.00367415707972771 0.0978952410119429 0.766190520401617 0.0602328509972929 brown

ARL2BP 0.539529234948154 -0.539529234948154 0.297789670956132 0.0277199916469901 -0.381747527813 2.01783354093894e-09 2.01783354093894e-09 0.00183836007414196 0.776851520956531 4.96625293141431e-05 blue

ARL3 -0.514213000350491 0.514213000350491 -0.438834640673283 -0.230939381094274 0.24014040328667 1.46806124262094e-08 1.46806124262094e-08 2.26580491511655e-06 0.0167001543942443 0.0127231377470708 brown

ARL4A -0.657447598932086 0.657447598932086 -0.79881260409149 -0.540919484900589 0.232677676677014 1.44886664909009e-14 1.44886664909009e-14 6.47731365568558e-25 1.80081721630857e-09 0.0158754355348367 blue

ARL4C 0.811567512510141 -0.811567512510141 0.856144594410398 0.486700837473711 -0.407187539464381 2.96789490049947e-26 2.96789490049947e-26 7.07200115962471e-32 1.06417611716655e-07 1.34486648271813e-05 blue

ARL4D 0.264826048215503 -0.264826048215503 0.0588973405510165 -0.0515524110760377 -0.172241400944217 0.00584048328824062 0.00584048328824062 0.546770512809851 0.597950757934384 0.0760572205268043 yellow

ARL5B -0.230333400540454 0.230333400540454 -0.0796084767229256 0.0423372653979701 0.186431638781052 0.016996244936569 0.016996244936569 0.415015595661016 0.665021686726094 0.0545170308066931 yellow

ARL5C -0.338359898986025 0.338359898986025 -0.40926700401903 -0.273378774254335 0.125585456346324 0.000364333841372442 0.000364333841372442 1.20287435117719e-05 0.00438399836039534 0.197426783016349 turquoise

ARL6 0.397185882266111 -0.397185882266111 0.311881778084742 0.0590725399427085 -0.348859619969101 2.27662152199265e-05 2.27662152199265e-05 0.00107468615878447 0.545576302873188 0.000230826357130879 blue

ARL6IP1 -0.118894144736567 0.118894144736567 -0.0832494370449893 -0.0190104192725601 0.0876202400417172 0.222564562523954 0.222564562523954 0.39393359131354 0.845899365193452 0.369487983826059 yellow

ARL6IP4 0.109224199259994 -0.109224199259994 -0.0263528642988953 -0.0516006397752688 -0.0495780250955495 0.262753021032361 0.262753021032361 0.787590215645309 0.597607808670602 0.612065319188394 greenyellow

ARL6IP5 -0.438896051269702 0.438896051269702 -0.0994537674346414 0.102134176275684 0.316428797283708 2.25758746190283e-06 2.25758746190283e-06 0.308106160528892 0.295184666746794 0.000898654722624169 brown

ARL6IP6 -0.619028839726981 0.619028839726981 -0.445924836066677 -0.249350026219056 0.219122126533754 1.18848311993289e-12 1.18848311993289e-12 1.48249123052107e-06 0.00959623662925597 0.0233519453216632 brown

ARL8A -0.734864459932092 0.734864459932092 -0.688221939113297 -0.369597983992593 0.364030570648683 2.06167616145279e-19 2.06167616145279e-19 2.60090208115888e-16 8.93178823125603e-05 0.000115988120040782 brown

ARL8B -0.43531352446399 0.43531352446399 -0.370401237986392 -0.135000297643352 0.304333749807173 2.78729876050933e-06 2.78729876050933e-06 8.59795506740581e-05 0.165622502052505 0.00143741389919352 yellow

ARL9 -0.0643209908161519 0.0643209908161519 0.001783617566745 0.0493178240888869 0.0810814076882965 0.510403973879217 0.510403973879217 0.985452823238617 0.613936238904547 0.406408965687635 grey

ARMC1 0.429731500539338 -0.429731500539338 0.422387178082954 0.158366085113454 -0.339552846524854 3.85256852498336e-06 3.85256852498336e-06 5.84697215514891e-06 0.10326869696176 0.000346200383683312 turquoise

ARMC10 0.209917011464485 -0.209917011464485 0.443280568978411 0.356161759979801 -0.03414810009607 0.0299966328205634 0.0299966328205634 1.73854513158068e-06 0.000166455760700293 0.726951412495167 yellow

ARMC12 -0.485167952471271 0.485167952471271 -0.427832357121135 -0.192051635116035 0.290257822183912 1.18237678426166e-07 1.18237678426166e-07 4.29544619870308e-06 0.0475069320190572 0.00242329477904848 blue

ARMC2 -0.0270703703817526 0.0270703703817526 0.0695590745906671 0.114859302940182 0.0946637867797772 0.781949149420049 0.781949149420049 0.476504120162425 0.238771759382792 0.33210198452736 grey

ARMC3 -0.0973499475155003 0.0973499475155003 -0.150509097053946 -0.135781318972471 -0.0135966359973495 0.318502582520638 0.318502582520638 0.121761820131231 0.163165680812282 0.889450808950814 turquoise

ARMC4 -0.0307701154928027 0.0307701154928027 -0.207206291377151 -0.166280913055998 0.0163059464467685 0.753046698570446 0.753046698570446 0.0322351510440325 0.0869410924565194 0.867606124655068 turquoise

ARMC5 0.194040062952284 -0.194040062952284 -0.00141070671171754 -0.0475069771935712 -0.0785469200841957 0.0452129421935385 0.0452129421935385 0.988494047202283 0.627024901981461 0.421283783637589 green

ARMC6 0.411063988819937 -0.411063988819937 0.0492832655790772 -0.194522955086661 -0.400895731486152 1.09165023475763e-05 1.09165023475763e-05 0.614184911302279 0.0446699555289671 1.87650069055211e-05 brown

ARMC7 -0.279519454527915 0.279519454527915 -0.19136767790904 -0.115508491798416 0.0796178623109022 0.00354817413778821 0.00354817413778821 0.0483179517532912 0.236110135578016 0.414960419878093 grey

ARMC8 -0.565626502681598 0.565626502681598 -0.507168749818984 -0.296176315732125 0.227878918875643 2.18017349516848e-10 2.18017349516848e-10 2.47904065678556e-08 0.00195163483396832 0.0182424497573548 brown

ARMC9 -0.024766759321481 0.024766759321481 -0.34475850339482 -0.32873242760594 -0.0611824229334864 0.800098897814686 0.800098897814686 0.000276397236051199 0.000546030028486276 0.531295699641104 yellow

ARMCX1 0.187138912711915 -0.187138912711915 0.181952767053091 0.270509077194185 0.196839356002645 0.0535905719761988 0.0535905719761988 0.0606941416658527 0.0048317557914903 0.042140117540577 turquoise

ARMCX2 0.619457148615599 -0.619457148615599 0.542857769847406 0.244495233237288 -0.366922730075165 1.13524429747953e-12 1.13524429747953e-12 1.53531718142821e-09 0.0111482848408037 0.000101326660699472 blue

ARMCX3 -0.209054754410712 0.209054754410712 -0.202053804160186 -0.111330393787817 0.102090597229955 0.0306939494964169 0.0306939494964169 0.0368800020456585 0.253604792397853 0.295391844631692 yellow

ARMCX4 0.30037930509549 -0.30037930509549 0.15748104605401 0.0718366842690506 -0.104900292299219 0.00166893815550547 0.00166893815550547 0.105234694311972 0.462152380570084 0.282230565280963 yellow

ARMCX5 0.555890899146196 -0.555890899146196 0.512095589558912 0.319123496792691 -0.196051270443972 5.11425016701213e-10 5.11425016701213e-10 1.72060907300747e-08 0.000807191465901981 0.0429870616263266 turquoise

ARMCX6 0.090120490002901 -0.090120490002901 -0.000498213737007516 0.0128983454753936 0.0225946187888308 0.355931911506677 0.355931911506677 0.995936366428424 0.895095113374741 0.817311019437851 salmon

ARNT -0.311872502130851 0.311872502130851 -0.269482271561409 -0.106256747552135 0.20778119333424 0.00107507534325467 0.00107507534325467 0.00500161102182941 0.276019086535484 0.0317489592915697 brown

ARNT2 -0.0624718826699394 0.0624718826699394 -0.147099063699196 -0.0955909334355445 0.0496617348010598 0.522661026333482 0.522661026333482 0.130541450316806 0.327366859598787 0.611463951446124 turquoise

ARNTL 0.148860793193901 -0.148860793193901 0.430778968864757 0.379157973730177 0.0228566118193887 0.125947041995829 0.125947041995829 3.62712800605949e-06 5.63908049060975e-05 0.815230191170847 tan

ARNTL2 -0.348644557229713 0.348644557229713 -0.454458656674446 -0.267401147027641 0.200792219744489 0.000233031864616237 0.000233031864616237 8.78371805953096e-07 0.00536228100692126 0.0380992249142644 blue

ARNTL2-AS1 -0.167304264922426 0.167304264922426 -0.332282352618982 -0.331032765923839 -0.0830475613796277 0.0849890513478071 0.0849890513478071 0.000471071927723672 0.000496305107673028 0.3950854926183 blue

ARPC1A -0.335242225383411 0.335242225383411 -0.506205412920714 -0.293183528253552 0.231568082998362 0.000415936375723311 0.000415936375723311 2.66074015769806e-08 0.00217861600644528 0.0163976901675063 brown

ARPC1B -0.499965572604276 0.499965572604276 -0.4650521930244 -0.219578260438905 0.297158991769526 4.18501090405299e-08 4.18501090405299e-08 4.49568190931115e-07 0.0230583783599795 0.00188191190276291 brown

ARPC2 -0.832232540837157 0.832232540837157 -0.584956913595622 -0.251492981241705 0.415668921277376 1.18010329367423e-28 1.18010329367423e-28 3.68561531006496e-11 0.00897385854088097 8.49165688358998e-06 brown

ARPC3 -0.727655783014888 0.727655783014888 -0.565229269867528 -0.264707686616375 0.364850714845915 6.84000376564596e-19 6.84000376564596e-19 2.25858039794643e-10 0.0058633467781912 0.000111641855101611 brown

ARPC4 -0.619322860603827 0.619322860603827 -0.566739441869798 -0.333572255564268 0.250221740191183 1.15168312954179e-12 1.15168312954179e-12 1.97420116073284e-10 0.000446266367034448 0.00933863004773444 brown

ARPC5 -0.560966980128859 0.560966980128859 -0.515728818194751 -0.271081509876377 0.282768031749549 3.29010358804808e-10 3.29010358804808e-10 1.30949291136341e-08 0.0047393135748202 0.00316648186131418 brown

ARPC5L -0.0352799170181617 0.0352799170181617 -0.277242422293186 -0.383715837237652 -0.251652726295842 0.718275685091173 0.718275685091173 0.00383979248475944 4.50584254245315e-05 0.00892891987008718 yellow

ARPIN 0.303790926427479 -0.303790926427479 0.152434540875056 0.118464860909028 -0.0185466502155429 0.00146736429093377 0.00146736429093377 0.117009564164869 0.224251046722625 0.84961397864404 yellow

ARPP19 -0.0680898148600929 0.0680898148600929 -0.139354292821511 -0.154252464789359 -0.0609870632844472 0.485888407713861 0.485888407713861 0.152270873734864 0.112655646097982 0.532610087456806 yellow

ARPP21 -0.101809308710269 0.101809308710269 -0.317521103460151 -0.226866511390305 0.0723785817643629 0.296731419086442 0.296731419086442 0.00086049554391032 0.0187789996964743 0.458773124745894 turquoise

ARR3 -0.391677473143579 0.391677473143579 -0.354595036760268 -0.165771590877285 0.229383446295229 3.02060838184951e-05 3.02060838184951e-05 0.000178670467049014 0.0879258304285855 0.0174695428915669 yellow

ARRB1 0.641892975074011 -0.641892975074011 0.711418467084 0.590468177507352 -0.0228045109610091 9.30064544337978e-14 9.30064544337978e-14 8.90450169934246e-18 2.17267833000694e-11 0.815643890206501 blue

ARRB2 -0.426438681383081 0.426438681383081 -0.140975675636957 0.127349358002446 0.418981252884342 4.65057754530006e-06 4.65057754530006e-06 0.147510873885691 0.191155409409091 7.07171555485332e-06 purple

ARRDC1 -0.317913716054553 0.317913716054553 -0.107244714585502 -0.0428624800839149 0.081712963585465 0.000847145682520389 0.000847145682520389 0.271553263331433 0.661120369383539 0.402751063535161 brown

ARRDC2 0.563488872230902 -0.563488872230902 0.325821436781247 0.0943937039704773 -0.309020906261965 2.63527164762184e-10 2.63527164762184e-10 0.000615504979238743 0.333489514439286 0.0012009949706719 blue

ARRDC3 0.400505614367434 -0.400505614367434 0.591227288906082 0.473229768969521 -0.0486023559606903 1.91523911455872e-05 1.91523911455872e-05 2.01856398951141e-11 2.63913259517629e-07 0.6190934175648 blue

ARRDC3-AS1 -0.322033161490534 0.322033161490534 -0.398142783848097 -0.217838503368057 0.203772144872009 0.000718053466122108 0.000718053466122108 2.16638138585817e-05 0.0241953013358137 0.0352721854479325 turquoise

ARRDC4 -0.225110571971834 0.225110571971834 -0.00980749005813776 0.183629055242809 0.325580137726277 0.0197418922812853 0.0197418922812853 0.920137682052556 0.0583184888896002 0.000621613179086775 brown

ARSA 0.0708661626558075 -0.0708661626558075 0.0947421967109347 0.131473630951087 0.0865846806197563 0.468238598384159 0.468238598384159 0.331699849081043 0.177058444366551 0.375194089280455 yellow

ARSB -0.160691539602238 0.160691539602238 -0.12009868147489 0.113416282714272 0.365289749202603 0.098240054498835 0.098240054498835 0.217880264017225 0.244762576465445 0.000109377938077769 brown

ARSD -0.0802896284004627 0.0802896284004627 -0.184081400919246 0.0183686469838932 0.296200181853774 0.411022367099591 0.411022367099591 0.0576906317999325 0.851040595697937 0.00194991415788343 blue

ARSE -0.233507280908777 0.233507280908777 -0.17330373542809 0.00326088039398646 0.255057513528776 0.0154944412823088 0.0154944412823088 0.0742376832103621 0.973407676120244 0.00801707406629013 turquoise

ARSF -0.0590116326842471 0.0590116326842471 -0.188258328104557 -0.190035712226095 -0.0512670339939843 0.545991319779595 0.545991319779595 0.0521507600034294 0.0499302705334507 0.599981845032058 turquoise

ARSG 0.207012181494655 -0.207012181494655 0.389515035302013 0.340867565916221 0.0173238224096173 0.0324007223538264 0.0324007223538264 3.37062528483415e-05 0.000327182080806856 0.859423945906823 black

ARSI -0.0166013061183862 0.0166013061183862 -0.38015794518067 -0.331267261809597 -0.0145129289238765 0.865230401111217 0.865230401111217 5.36976621351154e-05 0.000491477221827372 0.882052798977109 turquoise

ARSJ -0.16177031897345 0.16177031897345 -0.278245784896281 -0.167269452454193 0.116913520218223 0.0959736954058859 0.0959736954058859 0.00370874602751899 0.0850548770909438 0.230420636546128 turquoise

ARSK 0.582934621756537 -0.582934621756537 0.346151774464549 0.0223232700889931 -0.460533907426512 4.46326717672443e-11 4.46326717672443e-11 0.000260057939492955 0.81946749168344 5.99872058452161e-07 brown

ART1 -0.0457853398139871 0.0457853398139871 -0.278361491157696 -0.185389505292279 0.0863461432468986 0.639577086045381 0.639577086045381 0.00369389461998603 0.0559060824437929 0.376516034485722 turquoise

ART3 -0.265959834438969 0.265959834438969 -0.201691912447744 -0.0393898690375929 0.223590157132882 0.00562545529520279 0.00562545529520279 0.0372263450841896 0.687078705849079 0.0206096627811153 turquoise

ART4 -0.217438575756299 0.217438575756299 -0.595378996894472 -0.580015930540081 -0.126543378648138 0.0244633192383352 0.0244633192383352 1.34526278625711e-11 5.87013481386344e-11 0.19400282101516 pink

ART5 0.0310160959549714 -0.0310160959549714 0.0108469076701499 0.00532461844162023 -0.00658637977676011 0.75113664696817 0.75113664696817 0.911706212631197 0.95659119287296 0.946318562688321 yellow

ARTN -0.122037707318916 0.122037707318916 -0.244172998123274 -0.125618913967126 0.138499556288245 0.210487118151758 0.210487118151758 0.0112586713775168 0.197306463440377 0.154826113243721 green

ARV1 -0.163126624976194 0.163126624976194 -0.123267770993998 -0.108821398993571 -0.00709171823269506 0.0931830498699157 0.0931830498699157 0.205890869358361 0.264527841295652 0.942206720677142 yellow

ARVCF 0.0698435247753247 -0.0698435247753247 -0.179532900428544 -0.154815981795772 -0.00409276592476127 0.474698688200657 0.474698688200657 0.0642624390473063 0.111331898880611 0.966627193552671 green

ARX 0.0631067885296018 -0.0631067885296018 -0.137510072886044 -0.0965746860492579 0.034186709041212 0.518435734884875 0.518435734884875 0.157824111536023 0.322390117717507 0.726654886444647 green

AS3MT 0.180284606023705 -0.180284606023705 0.278501253208347 0.186291400080862 -0.0850176213225715 0.0631361575839653 0.0631361575839653 0.00367602661929423 0.0547022850017718 0.383930233808918 grey

ASAH1 -0.559625158528601 0.559625158528601 -0.191941697329024 0.0850044900907652 0.420540821008653 3.69968078826482e-10 3.69968078826482e-10 0.0476365274562545 0.384003953189215 6.48360535423882e-06 purple

ASAH2B 0.352020827523305 -0.352020827523305 0.158201000642821 0.0143590750417011 -0.203426479468969 0.000200557304398633 0.000200557304398633 0.103633210171698 0.883294301352372 0.035590792651492 brown

ASAP1 -0.554136409137829 0.554136409137829 -0.316020433047563 -0.135189606960496 0.225713961174285 5.94636250183667e-10 5.94636250183667e-10 0.000913313261443153 0.165024496959698 0.0194063304479936 blue

ASAP1-IT1 -0.422115051296506 0.422115051296506 -0.211924608223664 -0.111840608034961 0.115437426452061 5.93694647034506e-06 5.93694647034506e-06 0.0284249046865141 0.25142197840845 0.236400485600928 turquoise

ASAP1-IT2 -0.622488094025109 0.622488094025109 -0.242839580806183 0.00295527846304532 0.354658442992045 8.19163340869196e-13 8.19163340869196e-13 0.0117256866063442 0.975899059429013 0.000178160421173631 blue

ASAP2 -0.205748758206756 0.205748758206756 -0.265297481042577 -0.197993700659533 0.0461578705899799 0.0334960337869788 0.0334960337869788 0.00575020237847364 0.0409247142949133 0.636852225820853 salmon

ASAP3 -0.0566500205075 0.0566500205075 -0.263664410296105 -0.19592346462891 0.047317969971414 0.562201039590278 0.562201039590278 0.0060683371519864 0.0431257400324613 0.628397819940878 turquoise

ASB1 -0.165355030366265 0.165355030366265 -0.0624451173383645 -0.0462614629577731 0.0114443174859037 0.0887377934997282 0.0887377934997282 0.522839531519735 0.636095361288935 0.906864558221857 brown

ASB10 -0.207508619300568 0.207508619300568 -0.328106167897843 -0.214744189320859 0.108180223246945 0.0319786956601563 0.0319786956601563 0.000560338183423261 0.0263359258586922 0.267369760768262 turquoise

ASB11 0.0534708100882714 -0.0534708100882714 -0.124862704869319 -0.102850098277164 0.00533283864708049 0.584377769664714 0.584377769664714 0.200038859551005 0.291794857530187 0.956524243230716 green

ASB12 0.217989314683032 -0.217989314683032 0.0823958416656342 0.00374441186250933 -0.112284370221145 0.0240948862059313 0.0240948862059313 0.398817876217186 0.969466275335119 0.249533994747501 yellow

ASB13 0.0731116053269325 -0.0731116053269325 0.237678126803429 0.27182890255467 0.118843327183319 0.454223814859625 0.454223814859625 0.0136968025927617 0.00462099318340831 0.222763736024981 grey

ASB14 -0.182058441096617 0.182058441096617 -0.25778503944379 -0.187391377933306 0.0533242450526728 0.0605420809384187 0.0605420809384187 0.00734693425220923 0.0532630219322164 0.585409740426834 turquoise

ASB15 -0.0595477834320145 0.0595477834320145 -0.149401573777234 -0.0393782126931207 0.148321120553611 0.542343341368521 0.542343341368521 0.124561940143728 0.687166475407271 0.127341064700448 turquoise

ASB16 -0.188132285587382 0.188132285587382 -0.261516072228107 -0.131933952396173 0.152759367941197 0.052311267916072 0.052311267916072 0.00651060523245157 0.17553370011988 0.116222194228799 turquoise

ASB16-AS1 -0.0519309517297059 0.0519309517297059 0.0600765580304393 0.0766597570745846 0.0435254802017618 0.595261366651538 0.595261366651538 0.538757322167146 0.432561080738627 0.656208513057695 grey

ASB17 -0.078606131511092 0.078606131511092 -0.201837620437966 -0.144394739917984 0.0456978739510451 0.420932721994751 0.420932721994751 0.037086570644511 0.137841932855086 0.640217548438325 turquoise

ASB2 0.534266243178424 -0.534266243178424 0.389055271391274 0.146648059287 -0.311436094345323 3.08984535194636e-09 3.08984535194636e-09 3.44978093504272e-05 0.131737977259301 0.00109353126034062 brown

ASB4 -0.20375204134905 0.20375204134905 -0.306648327842024 -0.276275701354149 -0.0270808358032962 0.0352906493615792 0.0352906493615792 0.0013158398018828 0.00396995956122555 0.781866952597178 yellow

ASB5 -0.133936804205729 0.133936804205729 -0.0322768783559368 -0.00931255528367828 0.0306776075035977 0.169011848855925 0.169011848855925 0.741370410801623 0.924155626991402 0.753765414513599 grey

ASB6 0.389087246579895 -0.389087246579895 0.194376930441193 -0.010476027056033 -0.297637019748654 3.4442200926115e-05 3.4442200926115e-05 0.0448335776606199 0.914713619139093 0.00184881691416677 blue

ASB7 -0.495982173254951 0.495982173254951 -0.455331841854239 -0.313179147923124 0.124403942319338 5.56189621985493e-08 5.56189621985493e-08 8.31898383555127e-07 0.00102150156799867 0.201709712395712 brown

ASB8 0.288072716370219 -0.288072716370219 -0.0226897255890236 -0.108136463054777 -0.150744494743592 0.00262191825985662 0.00262191825985662 0.81655550478853 0.267564472892556 0.121172970286251 greenyellow

ASB9 -0.128567791838864 0.128567791838864 -0.057570774265567 0.018862615936285 0.114885176741224 0.186908595167128 0.186908595167128 0.555853952236493 0.847082851208624 0.238665281184789 grey

ASCC1 -0.295000136963311 0.295000136963311 -0.352317791107227 -0.195326161534878 0.175976196637518 0.00203815530318787 0.00203815530318787 0.000197911395268193 0.0437788061513919 0.0698151129561843 greenyellow

ASCC2 0.377127770743597 -0.377127770743597 -0.0250626179730619 -0.186108938716153 -0.279579564686011 6.22508710881517e-05 6.22508710881517e-05 0.797761692367395 0.0549440892834934 0.00354075245698012 pink

ASCC3 0.376393885137189 -0.376393885137189 0.315533729141101 0.123296620668783 -0.245185291245193 6.45055226877653e-05 6.45055226877653e-05 0.000931068935180986 0.205783939315152 0.0109150771989729 turquoise

ASCL1 -0.166709952196539 0.166709952196539 -0.332960997352364 -0.226146561300309 0.0958304011402971 0.0861184026013178 0.0861184026013178 0.000457867447478258 0.01916878292932 0.326150904719597 turquoise

ASCL2 0.546449860826405 -0.546449860826405 0.682377841220788 0.604224630721319 0.0423415864899437 1.13933863041583e-09 1.13933863041583e-09 5.7949200530548e-16 5.55644943119955e-12 0.664989552782281 brown

ASCL3 -0.166537453394003 0.166537453394003 -0.317732891611106 -0.242212575797295 0.0466545297087544 0.0864484237700973 0.0864484237700973 0.000853270494374961 0.011951082260926 0.633226954821907 turquoise

ASCL5 0.00428110818893287 -0.00428110818893287 -0.244052293905659 -0.259592766106816 -0.0889115395845305 0.965092377541921 0.965092377541921 0.0113002660472874 0.00693054663794072 0.362447656058761 green

ASF1A 0.445631128723155 -0.445631128723155 0.346045655036169 0.0459496800791354 -0.420307644417095 1.50905975759989e-06 1.50905975759989e-06 0.000261270307806391 0.638374432620177 6.5685029144748e-06 turquoise

ASF1B 0.288018391321446 -0.288018391321446 0.0483901688847393 -0.124996353157297 -0.281683547847492 0.00262703799481001 0.00262703799481001 0.620626462540038 0.199553979933173 0.00328959053743738 grey

ASGR1 -0.203610026136261 0.203610026136261 0.255596868636913 0.494894856280702 0.47139278199062 0.0354213137567897 0.0354213137567897 0.00788043205257468 6.00714913884932e-08 2.97818920725939e-07 grey

ASGR2 -0.538170216963849 0.538170216963849 -0.203041547057687 0.115720223999563 0.488620575763169 2.25410629413109e-09 2.25410629413109e-09 0.0359484373419701 0.235246540389254 9.31989386457875e-08 brown

ASH1L 0.521966288267329 -0.521966288267329 0.60624260083265 0.332854468366064 -0.308316715446238 8.13319279984813e-09 8.13319279984813e-09 4.52406711160749e-12 0.000459917417210896 0.0012340905410484 blue

ASH1L-AS1 -0.271759592072565 0.271759592072565 -0.304440140769819 -0.202137473709931 0.0954879858064981 0.00463185379309409 0.00463185379309409 0.00143160925941732 0.0368003134858888 0.327890491343799 yellow

ASH2L -0.219769403204948 0.219769403204948 -0.460386553535225 -0.281768074572163 0.184959154413269 0.0229363017293404 0.0229363017293404 6.05499270873338e-07 0.00327984305515081 0.0564881074736584 brown

ASIC1 -0.055324063645 0.055324063645 -0.240577783431902 -0.192249568160244 0.0203087181528069 0.571401507096537 0.571401507096537 0.0125566492216878 0.0472743436920855 0.835518675933662 turquoise

ASIC2 -0.220002933033543 0.220002933033543 -0.299950146511122 -0.0669100726662016 0.318386720864017 0.0227879052197351 0.0227879052197351 0.00169599789423289 0.493494259690801 0.000831313818098128 turquoise

ASIC4 0.0408967987215435 -0.0408967987215435 -0.12729735857893 -0.148394352352376 -0.0684107493996741 0.675767256161456 0.675767256161456 0.19133819623083 0.12715120957615 0.483830197237601 green

ASIC5 -0.0400776594150006 0.0400776594150006 0.0342134019871559 0.0204488033547162 -0.0145773579436664 0.681907199462183 0.681907199462183 0.726449901747923 0.834400262290472 0.881532982675141 yellow

ASIP 0.0330024719302128 -0.0330024719302128 -0.143913504356992 -0.105524451039849 0.0282265293176598 0.735768285951061 0.735768285951061 0.139172950124272 0.279360909329288 0.772883229603871 turquoise

ASL -0.358526179206012 0.358526179206012 -0.46890004885973 -0.305588109820685 0.156815182380633 0.000149480446419101 0.000149480446419101 3.50503754786455e-07 0.00137031743911223 0.106733027482743 greenyellow

ASMT -0.0892485829764503 0.0892485829764503 -0.264454078175729 -0.240489896825467 -0.0271355864400102 0.360623771865206 0.360623771865206 0.00591260331871349 0.0125899497551577 0.781436972921403 green

ASMTL 0.217295155004096 -0.217295155004096 0.1700616512225 0.0542346171247143 -0.152869508697605 0.0245600507098218 0.0245600507098218 0.079902871741679 0.57901332351772 0.11595615030425 pink

ASMTL-AS1 0.0907266665246041 -0.0907266665246041 -0.16551126607691 -0.190557270090489 -0.084903392213875 0.352692450842268 0.352692450842268 0.0884325624546693 0.0492937166747816 0.384571807512331 turquoise

ASNA1 -0.0889179842383031 0.0889179842383031 -0.370282678823901 -0.309527989778869 0.00814110148527464 0.362412728004319 0.362412728004319 8.64648641248292e-05 0.00117766574328705 0.933673061606735 tan

ASNS -0.19414080868035 0.19414080868035 -0.27205590306755 -0.416073756849188 -0.314003681612665 0.0450992084937441 0.0450992084937441 0.00458558226228583 8.30471759981305e-06 0.000988960028643009 magenta

ASNSD1 -0.0864347547556884 0.0864347547556884 0.0952112854886731 0.139031883102036 0.0987290182923055 0.376024629936601 0.376024629936601 0.32930054994992 0.15323097708705 0.311662370269703 yellow

ASPA -0.116275229559676 0.116275229559676 -0.25342651981609 -0.184428028027278 0.0520750182196801 0.232993298209838 0.232993298209838 0.00844308072585418 0.0572132695284931 0.594239258974248 turquoise

ASPDH 0.193663857034898 -0.193663857034898 -0.0368041671709042 -0.114861985164651 -0.141829576091893 0.0456397565301148 0.0456397565301148 0.706648355266076 0.238760719716219 0.145049524836429 green

ASPG -0.162145604465363 0.162145604465363 -0.345898284556193 -0.288053684758077 0.00945514656672435 0.0951950167161115 0.0951950167161115 0.000262962615027863 0.00262371082493059 0.922997852291677 turquoise

ASPH -0.77418530671593 0.77418530671593 -0.624185231494857 -0.290391976226446 0.406172920107375 1.39069009410358e-22 1.39069009410358e-22 6.81311511690723e-13 0.00241155459166914 1.41973237950654e-05 brown

ASPHD1 -0.0552813616428879 0.0552813616428879 -0.31518600091536 -0.257557420553491 0.016960583310654 0.571698974684204 0.571698974684204 0.000943947001051967 0.00740090093258141 0.862342176896168 turquoise

ASPHD2 0.0765371870923573 -0.0765371870923573 0.171532172740988 0.280972032534683 0.229589678993803 0.433299441694634 0.433299441694634 0.0772918036599441 0.00337267777307615 0.0173658342877836 cyan

ASPM -0.489506078948476 0.489506078948476 -0.672458809006842 -0.532527873132643 0.0649839086228335 8.76439596075264e-08 8.76439596075264e-08 2.16510629902289e-15 3.55111961450266e-09 0.506046099040416 magenta

ASPN -0.281357892555906 0.281357892555906 -0.420970306305121 -0.334781255274544 0.0382894011589453 0.00332738814647305 0.00332738814647305 6.32993456203193e-06 0.000424113120591001 0.695383156829674 blue

ASPRV1 0.234332405169009 -0.234332405169009 0.512764208420308 0.493206874658859 0.098254366296058 0.0151233964554835 0.0151233964554835 1.63668176271033e-08 6.76644472060961e-08 0.31400579638552 yellow

ASPSCR1 0.635493805297121 -0.635493805297121 0.478407396251364 0.212340356312614 -0.328664530485618 1.93853658941627e-13 1.93853658941627e-13 1.8700452765319e-07 0.0281083116138711 0.000547564898885442 blue

ASRGL1 0.287574723618885 -0.287574723618885 0.107197074255184 -0.00244249224116759 -0.158487555796212 0.00266918873120335 0.00266918873120335 0.271767482046637 0.980079996536382 0.103001126936772 red

ASS1 0.310008138807393 -0.310008138807393 0.334935605874475 0.144785882514524 -0.236671894778651 0.00115595684154575 0.00115595684154575 0.000421358901220186 0.136767240881341 0.014112996275382 brown

ASTE1 0.668884824867413 -0.668884824867413 0.631967325836345 0.304860351707597 -0.39283746918156 3.43848743434958e-15 3.43848743434958e-15 2.88467068055181e-13 0.00140889033180902 2.84717737882611e-05 blue

ASTN1 -0.340668255364407 0.340668255364407 -0.397784290994001 -0.246142876809372 0.155248047490578 0.000330001873988641 0.000330001873988641 2.20708155773046e-05 0.0105985410618235 0.110325161385486 blue

ASTN2 0.259711600488654 -0.259711600488654 0.0974098357427835 -0.0454666736938627 -0.217370302832693 0.00690392131810415 0.00690392131810415 0.318203538398718 0.641911766946471 0.0245093258952861 brown

ASUN -0.169380708136541 0.169380708136541 -0.109435121455901 -0.0963300576508776 -0.00582132159465924 0.0811356597777653 0.0811356597777653 0.261826894838605 0.323623108609659 0.952546342213545 yellow

ASXL1 0.687418521926702 -0.687418521926702 0.510250408351133 0.109675760715347 -0.548645484503798 2.90695051910099e-16 2.90695051910099e-16 1.97412814346484e-08 0.260773003985008 9.47794436377712e-10 blue

ASXL2 -0.176037256280893 0.176037256280893 0.0439886381714968 0.0182613331001954 -0.03236222796932 0.0697166146430309 0.0697166146430309 0.652785847964378 0.851900901484336 0.740710739834404 green

ASXL3 -0.216933583426075 0.216933583426075 -0.374525994959892 -0.259491727599042 0.0991188284796628 0.0248053668674289 0.0248053668674289 7.0592401175049e-05 0.00695325618489595 0.309746342150302 turquoise

ASZ1 0.131279988500477 -0.131279988500477 -0.113842778426357 -0.207220699860909 -0.187560249470771 0.177702753041412 0.177702753041412 0.242981221928545 0.0322228894766481 0.0530448482248661 grey

ATAD1 0.374639465124938 -0.374639465124938 0.287156619021533 0.0733199368246508 -0.289094310003716 7.02078699828869e-05 7.02078699828869e-05 0.0027094673590273 0.452935452120734 0.0025272998242726 yellow

ATAD2 -0.225873724511575 0.225873724511575 -0.29516014041338 -0.287944147149765 -0.0634130380814902 0.0193183083035573 0.0193183083035573 0.00202618501555223 0.00263404952631081 0.5164038870322 magenta

ATAD2B -0.0866499306920856 0.0866499306920856 -0.0223482061774172 0.0021888500568324 0.0358900184999226 0.374832974224621 0.374832974224621 0.819269262274036 0.982148242196952 0.713613793516461 yellow

ATAD3A 0.379342378522011 -0.379342378522011 0.0752258636657169 -0.0684195012939748 -0.22436033631707 5.58848681747828e-05 5.58848681747828e-05 0.441243683579361 0.483774135151533 0.0201660742682021 brown

ATAD3B 0.203121480622749 -0.203121480622749 -0.0189952863069931 -0.0873139942509449 -0.120746160458413 0.0358739227899707 0.0358739227899707 0.846020521678817 0.371169885495425 0.215391362370785 green

ATAD3C 0.269368124241619 -0.269368124241619 0.0111098752826765 -0.0496913898225407 -0.10027935797486 0.00502081824087518 0.00502081824087518 0.909574606396137 0.611250972648837 0.304087507975015 green

ATAD5 -0.331831371467627 0.331831371467627 -0.243095075674288 -0.123209074381176 0.141034928504398 0.000480039403413429 0.000480039403413429 0.0116349125210158 0.206108548562003 0.1473390693696 blue

ATAT1 0.181073274939051 -0.181073274939051 -0.0871511773590047 -0.15965735550274 -0.145317682352894 0.0619718333246924 0.0619718333246924 0.372065979158571 0.100452076955639 0.135316319609173 green

ATCAY -0.195991955884276 0.195991955884276 -0.300812608878709 -0.173078290850144 0.139553184411061 0.0430513758764063 0.0430513758764063 0.0016420147891321 0.0746208583895251 0.151680849896558 turquoise

ATE1 -0.310592642785736 0.310592642785736 -0.423534794175523 -0.355010031686439 0.00767114393803239 0.00113002198560011 0.00113002198560011 5.48149025745274e-06 0.000175356607524752 0.93749391901991 brown

ATF1 0.0776015507815284 -0.0776015507815284 0.0710305039062424 -0.0213424065680741 -0.138470872205802 0.426911723407985 0.426911723407985 0.467204934988801 0.827273763730364 0.154912417727271 yellow

ATF2 0.000509923229693909 -0.000509923229693909 0.00302142731539994 -0.00738095794411284 -0.0168694252498679 0.995840859893503 0.995840859893503 0.975359768318692 0.939853908653635 0.863074825102573 yellow

ATF3 0.067395920405865 -0.067395920405865 0.034538490729879 0.24938088856272 0.373253540985845 0.490354389069995 0.490354389069995 0.723954981915228 0.00958701073067719 7.50411245222178e-05 cyan

ATF4 -0.247906780660715 0.247906780660715 -0.317543417601914 -0.281762854410116 -0.0207007677360375 0.010036482693308 0.010036482693308 0.000859731681371218 0.00328044428348599 0.832389458754647 magenta

ATF5 0.175222915022579 -0.175222915022579 -0.109063517982265 -0.0867525577889885 0.00988835945213704 0.0710395555864152 0.0710395555864152 0.26346004155622 0.374265431198203 0.919481362025523 green

ATF6 -0.391307775754627 0.391307775754627 -0.0617788929905393 0.133043207042343 0.314609465397962 3.07792890845364e-05 3.07792890845364e-05 0.527292681863019 0.171899164159002 0.000965657702107372 brown

ATF6B 0.47558051251924 -0.47558051251924 0.222985740060592 0.0678182684648827 -0.206031146949515 2.25860898715872e-07 2.25860898715872e-07 0.0209636280523968 0.487633522740466 0.0332485484108767 yellow

ATF7 -0.639450109349227 0.639450109349227 -0.681503675920549 -0.425437717997328 0.259645906927253 1.23355148422139e-13 1.23355148422139e-13 6.52226275765681e-16 4.92254961539791e-06 0.00691862900798791 brown

ATF7IP 0.15676640152118 -0.15676640152118 0.55756008093266 0.471656262693083 -0.00279640708628598 0.106843446420797 0.106843446420797 4.42733188550247e-10 2.92713167172805e-07 0.977194332464859 black

ATF7IP2 0.680764717099194 -0.680764717099194 0.52365596322776 0.171608589703389 -0.462900881668482 7.20557138180979e-16 7.20557138180979e-16 7.13687761682519e-09 0.0771580127648359 5.16014396569734e-07 blue

ATG10 -0.487028557013004 0.487028557013004 -0.430652460174012 -0.236799554269432 0.218419879162776 1.04041136792996e-07 1.04041136792996e-07 3.65367851351518e-06 0.0140595922721641 0.0238101598282806 brown

ATG101 0.180248026849836 -0.180248026849836 0.0805913745842285 0.0175401150869695 -0.0862869053914796 0.0631905894823401 0.0631905894823401 0.409260602071315 0.857687165522429 0.376844762012064 greenyellow

ATG12 -0.451919537375726 0.451919537375726 -0.149796414952187 0.0206921809199631 0.250776914079408 1.02791515686695e-06 1.02791515686695e-06 0.123558051032858 0.83245796818969 0.00917775415778233 yellow

ATG13 0.307544025695638 -0.307544025695638 -0.096624638064492 -0.236447578179377 -0.26192389678155 0.00127135887882224 0.00127135887882224 0.322138718419805 0.0142072624639242 0.00642451436720424 grey

ATG14 0.465037855796179 -0.465037855796179 0.0947612380279842 -0.129969861515891 -0.356885393549083 4.49982790036681e-07 4.49982790036681e-07 0.331602240066027 0.182107207824048 0.000161079578548411 turquoise

ATG16L1 0.71582663532227 -0.71582663532227 0.639623194576266 0.301725407913476 -0.409177853817814 4.51546611699381e-18 4.51546611699381e-18 1.20921528983224e-13 0.00158658264654979 1.20866071802817e-05 blue

ATG16L2 0.243743313843519 -0.243743313843519 0.582962860778118 0.486692917903479 -0.0138677393008447 0.0114073538472 0.0114073538472 4.45139174813253e-11 1.06475676603052e-07 0.887260944088077 black

ATG2A 0.591324734038705 -0.591324734038705 0.475294177887426 0.25300959659463 -0.255201653972187 1.99956161218153e-11 1.99956161218153e-11 2.30199646743989e-07 0.00855513078570579 0.00798035384112192 black

ATG2B 0.147481689469324 -0.147481689469324 0.125778473587878 -0.00892088446437833 -0.19622968283913 0.129532864674592 0.129532864674592 0.196733386253075 0.927336619111738 0.0427940929439218 yellow

ATG3 -0.52957896366809 0.52957896366809 -0.271785735577696 0.00298056992500604 0.39637863550666 4.48844668786018e-09 4.48844668786018e-09 0.00462775456228643 0.9756928646757 2.37368990640267e-05 brown

ATG4A -0.315652381713185 0.315652381713185 -0.578953154372774 -0.491916330103847 -0.00076511152335413 0.00092671149822652 0.00092671149822652 6.48166007996453e-11 7.40788343615666e-08 0.993759475813059 yellow

ATG4B 0.520029323007826 -0.520029323007826 0.303276320129581 0.0735274530830652 -0.311951800217151 9.43929657363802e-09 9.43929657363802e-09 0.00149628001290423 0.451654155378168 0.00107175243270956 brown

ATG4C 0.023717207538379 -0.023717207538379 0.136070170089111 0.113225439962628 -0.00387163233217141 0.808404186985049 0.808404186985049 0.162263950203102 0.245562592188769 0.968429383817428 yellow

ATG4D -0.170862995610829 0.170862995610829 -0.400929483293643 -0.28869756116855 0.0875981800751124 0.0784713747466593 0.0784713747466593 1.87318395529477e-05 0.0025636742591059 0.369608980842282 pink

ATG5 -0.092262549761026 0.092262549761026 -0.00435745508084888 0.00727876770364313 0.0186196899331123 0.34456719423341 0.34456719423341 0.964470256670692 0.940685114132382 0.849028737795116 yellow

ATG7 -0.381880385663663 0.381880385663663 -0.032516652067476 0.195787302382621 0.378899274684496 4.93384284195179e-05 4.93384284195179e-05 0.739517666796984 0.0432738955503787 5.71077869003903e-05 purple

ATG9A -0.487475992147963 0.487475992147963 -0.607644373731122 -0.395303188214157 0.204413784604331 1.00878149379993e-07 1.00878149379993e-07 3.91875762491816e-12 2.50904602543601e-05 0.0346871309486101 blue

ATG9B 0.429306398625582 -0.429306398625582 0.204607156099341 0.029570768616083 -0.24444207471599 3.94778376703096e-06 3.94778376703096e-06 0.0345124227322666 0.762380850500023 0.011166429581003 brown

ATHL1 0.205791729724717 -0.205791729724717 0.478149575375159 0.439372529736906 0.0567832037671731 0.0334582736744125 0.0334582736744125 1.90265642184218e-07 2.19478062633427e-06 0.561280828548955 black

ATIC 0.712291810383494 -0.712291810383494 0.597104080969439 0.221925989872729 -0.483308195824865 7.79150695159804e-18 7.79150695159804e-18 1.13458231016531e-11 0.0215968600434771 1.34262031194881e-07 blue

ATL1 -0.0600115688025359 0.0600115688025359 -0.249413075784507 -0.297849681453779 -0.146081752123981 0.539197430763406 0.539197430763406 0.0095773970814117 0.00183426395109602 0.133252247485071 salmon

ATL2 0.121520966059089 -0.121520966059089 0.0540375374896184 -0.0406272693879086 -0.146713673250337 0.212439636333983 0.212439636333983 0.580395289317161 0.677785219879593 0.131563382770448 yellow

ATL3 -0.489848427261478 0.489848427261478 -0.634752122843353 -0.439209910942882 0.168972535961652 8.55821950246914e-08 8.55821950246914e-08 2.10844982337431e-13 2.21602791515642e-06 0.0818818843248252 brown

ATM 0.653269853287088 -0.653269853287088 0.716634708716448 0.317244327908713 -0.493738634134653 2.41285299412176e-14 2.41285299412176e-14 3.98141596578851e-18 0.000870021882382167 6.51787753665365e-08 blue

ATMIN 0.528336293132155 -0.528336293132155 0.38319387421257 0.118110848818974 -0.351399625873011 4.95078925609479e-09 4.95078925609479e-09 4.62388512402264e-05 0.225648576598391 0.000206198379004539 turquoise

ATN1 -0.0492389999508006 0.0492389999508006 -0.247013214006844 -0.25842712188693 -0.0826712413710071 0.614503497018073 0.614503497018073 0.0103178552627004 0.00719657198868512 0.397238122710798 turquoise

ATOH1 -0.421515270217566 0.421515270217566 -0.434278680828648 -0.261746797429181 0.181327158748231 6.13988628194198e-06 6.13988628194198e-06 2.96095513363886e-06 0.00646177486651623 0.0616007685702728 blue

ATOH7 -0.325167305669065 0.325167305669065 -0.163134776394547 -0.0214347362961654 0.198529035200321 0.000632192575489594 0.000632192575489594 0.0931664742088267 0.826538220808692 0.0403710803862094 blue

ATOH8 0.103593090298448 -0.103593090298448 -0.102063677673871 -0.0908464598935293 -0.00713396564540993 0.288304245516597 0.288304245516597 0.295519870063598 0.352054447222362 0.941863028840518 turquoise

ATOX1 -0.508065875704021 0.508065875704021 -0.545066585197778 -0.23976406995292 0.378127705681374 2.32054832618239e-08 2.32054832618239e-08 1.27855710491303e-09 0.0128679267818686 5.92970424827668e-05 greenyellow

ATP10A 0.619893076541212 -0.619893076541212 0.469543383070507 0.219410504157958 -0.303910028917563 1.08342903410877e-12 1.08342903410877e-12 3.36116115867022e-07 0.0231659766399375 0.00146074466757927 brown

ATP10B -0.268612083136027 0.268612083136027 -0.32962394880709 -0.191164044465286 0.150360591075875 0.00514970277901639 0.00514970277901639 0.000526239575786842 0.0485616179874517 0.122134442319973 turquoise

ATP10D 0.329674450451336 -0.329674450451336 0.603785943159481 0.581430275661274 0.116837745677337 0.000525138478936411 0.000525138478936411 5.80925703773584e-12 5.14200084481178e-11 0.230725004013741 blue

ATP11A -0.631376028547073 0.631376028547073 -0.538909655370229 -0.287983140359939 0.287476919338547 3.08194682123804e-13 3.08194682123804e-13 2.12244151802619e-09 0.00263036495450389 0.00267856219641054 brown

ATP11A-AS1 -0.246907574220812 0.246907574220812 -0.275699818743607 -0.128231085303389 0.179461993570297 0.0103515729631178 0.0103515729631178 0.00404936477546783 0.188075246461841 0.0643695173469159 turquoise

ATP11B -0.75255531444052 0.75255531444052 -0.734983636457477 -0.446746479718351 0.300505112241137 9.14293320546358e-21 9.14293320546358e-21 2.02054090608867e-19 1.41050027051431e-06 0.00166108017362909 brown

ATP11C -0.187175386539334 0.187175386539334 -0.130642502643531 -0.0436320639938897 0.114096291821677 0.0535431483928965 0.0535431483928965 0.179836021455009 0.655420241850776 0.241926633435062 yellow

ATP12A -0.18015268858759 0.18015268858759 -0.411637874230896 -0.288623721581301 0.103141622232433 0.0633326379280408 0.0633326379280408 1.05821908475422e-05 0.00257049581304994 0.290421941618972 turquoise

ATP13A1 0.526241122026071 -0.526241122026071 0.54456525427086 0.325393645075034 -0.232166828730021 5.83545886258646e-09 5.83545886258646e-09 1.33293703782133e-09 0.000626372045600628 0.0161140589239865 blue

ATP13A2 0.244677920090149 -0.244677920090149 -0.0606420004374775 -0.102111278868096 -0.085880452182972 0.0110861244721328 0.0110861244721328 0.534935640344481 0.295293510688109 0.379104987580397 green

ATP13A3 -0.643311667904635 0.643311667904635 -0.409137387077854 -0.130834064355144 0.367173422324943 7.88462101259937e-14 7.88462101259937e-14 1.21129587448262e-05 0.179193021560737 0.00010014061622417 brown

ATP13A4 -0.298276997534575 0.298276997534575 -0.400183407272701 -0.310212375967986 0.0500320302356099 0.00180533488359639 0.00180533488359639 1.94779881545627e-05 0.00114683370164409 0.608806861674709 turquoise

ATP13A4-AS1 -0.164688106032645 0.164688106032645 -0.147312718097083 -0.011823468642017 0.192050003827093 0.090050155959107 0.090050155959107 0.129977528388304 0.903793518713121 0.0475088528563495 turquoise

ATP13A5 -0.219467890563087 0.219467890563087 -0.408267140546464 -0.301599138266094 0.0762803732253396 0.0231291209505531 0.0231291209505531 1.26929016021114e-05 0.00159414813231781 0.434848814805216 turquoise

ATP1A1 0.639048564428635 -0.639048564428635 0.403595514013225 0.159533906163685 -0.310515384299694 1.29184976611462e-13 1.29184976611462e-13 1.62790569005663e-05 0.100718717024654 0.00113341932073898 blue

ATP1A1-AS1 -0.0486156204034049 0.0486156204034049 -0.246469424771348 -0.152346882227021 0.0964718374085137 0.618997636505499 0.618997636505499 0.0104924536451122 0.117222751541106 0.322908133167524 green

ATP1A2 -0.200345880965225 0.200345880965225 -0.19899210809497 -0.0618587853700723 0.181592683560586 0.0385385882133038 0.0385385882133038 0.0398972404742188 0.526757663782704 0.0612146313407988 turquoise

ATP1A4 -0.234777608009737 0.234777608009737 -0.360110971692514 -0.281599543790864 0.040865982211141 0.0149264179828836 0.0149264179828836 0.000139017295511139 0.00329930350535732 0.675997863206741 turquoise

ATP1B1 0.268178454530536 -0.268178454530536 0.136887051848337 0.0166711466985229 -0.168816596832925 0.00522494711515879 0.00522494711515879 0.159733889778977 0.86466881350511 0.0821684183185728 blue

ATP1B2 0.0241722234115523 -0.0241722234115523 -0.2758041639988 -0.226588410529709 0.0127852644657928 0.804800894048841 0.804800894048841 0.00403487264822316 0.0189287468090563 0.89600965103387 green

ATP1B3 -0.0338645416597308 0.0338645416597308 -0.0576610958450662 0.0619063912287929 0.188023160951136 0.729130442766071 0.729130442766071 0.555233194609908 0.526438989911085 0.0524505605133174 greenyellow

ATP1B4 0.0138940162790254 -0.0138940162790254 -0.143563727441076 -0.073875414776178 0.0814038906198386 0.887048733909492 0.887048733909492 0.140146479274534 0.449510228068659 0.40453874399008 turquoise

ATP2A1 0.038510678291509 -0.038510678291509 -0.168345852669851 -0.116089922809853 0.0454843705933163 0.693710401247081 0.693710401247081 0.0830382618976363 0.233743932757879 0.6417820202723 green

ATP2A2 0.251786775866528 -0.251786775866528 0.246077058921252 0.198583866721322 -0.0174828611302877 0.00889136270827954 0.00889136270827954 0.0106200371680022 0.0403147296343903 0.858146835886968 turquoise

ATP2A3 -0.149897181682972 0.149897181682972 -0.0877182937781951 -0.118709203753652 -0.0750476778869948 0.123302850016859 0.123302850016859 0.368950461273468 0.223290021044596 0.442329476421457 grey

ATP2B1 0.529464511618919 -0.529464511618919 0.780553468211961 0.584917474518766 -0.131761380556136 4.52923438528169e-09 4.52923438528169e-09 3.70884957578033e-23 3.69944811389299e-11 0.176104182687644 blue

ATP2B2 0.0684095665459974 -0.0684095665459974 -0.169506698220429 -0.14669787473247 -0.00475934515939196 0.483837774520239 0.483837774520239 0.0809064255534841 0.131605405432266 0.961195772276255 green

ATP2B3 -0.222180218033746 0.222180218033746 -0.44387060804837 -0.345033179052636 0.0538731686831587 0.0214434737790247 0.0214434737790247 1.67801739681504e-06 0.000273102870806713 0.581549043158761 yellow

ATP2B4 -0.0732514806926996 0.0732514806926996 -0.0215775088292005 -0.0313208717225374 -0.0220565561063629 0.453358573733803 0.453358573733803 0.825401121806787 0.748772130999276 0.821588443057284 grey

ATP2C1 -0.487383394239189 0.487383394239189 -0.562901124556893 -0.374623159070523 0.175067473550261 1.01525116021778e-07 1.01525116021778e-07 2.77562539653812e-10 7.02630080286724e-05 0.071294369505156 yellow

ATP2C2 -0.445945329761809 0.445945329761809 -0.620779349475516 -0.492980197614998 0.05765271090429 1.48065402597578e-06 1.48065402597578e-06 9.85041905451711e-13 6.87513149627519e-08 0.555290808151493 magenta

ATP4A -0.0459920520249688 0.0459920520249688 -0.113266766736171 -0.0248092308216013 0.121004323262113 0.638064503627204 0.638064503627204 0.245389196582436 0.799763275140101 0.214404638860913 turquoise

ATP4B -0.241562027061771 0.241562027061771 -0.25879451819844 -0.172736200353843 0.0796348392533105 0.0121889342381084 0.0121889342381084 0.00711176552620894 0.0752053298037606 0.414860627068727 turquoise

ATP5A1 -0.0374323896520045 0.0374323896520045 0.105980287660236 0.0428573826177581 -0.0799010314272405 0.701875514690507 0.701875514690507 0.277277534977133 0.661158190226231 0.413297742047266 red

ATP5B -0.0325741350820279 0.0325741350820279 -0.191955681684903 -0.131710496778284 0.0529837818554134 0.73907371341885 0.73907371341885 0.0476200263568292 0.176272652492819 0.58781017042058 greenyellow

ATP5C1 -0.417600267292593 0.417600267292593 -0.307413932273126 -0.109073274785153 0.257618867041036 7.63411703100581e-06 7.63411703100581e-06 0.00127773331345007 0.263417073372033 0.00738629802084636 yellow

ATP5D 0.365571171145764 -0.365571171145764 0.227389355935706 0.0865788455767105 -0.180551173269082 0.000107949291624449 0.000107949291624449 0.0185002306614732 0.375226392617553 0.062740642701462 red

ATP5E -0.604860562874176 0.604860562874176 -0.555423191648667 -0.352912320399907 0.201125021209323 5.20875006780217e-12 5.20875006780217e-12 5.32441362951829e-10 0.000192711078243253 0.0377743633666977 brown

ATP5F1 -0.444195301264132 0.444195301264132 -0.38346243792783 -0.25427022205872 0.120842050843719 1.64556650196008e-06 1.64556650196008e-06 4.56279282937161e-05 0.00822028231578867 0.215024484198647 yellow

ATP5G1 0.121769057117175 -0.121769057117175 0.0986219831309518 -0.00304132260459429 -0.147156615988492 0.21150061560094 0.21150061560094 0.312189823430499 0.975197570347033 0.130389362353657 red

ATP5G2 0.192931579063908 -0.192931579063908 0.393139917063139 0.33211724003021 -0.00273709837898292 0.0464801402209123 0.0464801402209123 2.80351675673101e-05 0.00047433706095781 0.977677889751474 red

ATP5G3 -0.0723377097200509 0.0723377097200509 -0.174982900509497 -0.151916873354823 -0.00572670049717688 0.459027524599764 0.459027524599764 0.0714333192443357 0.118272896765388 0.953316789578141 greenyellow

ATP5H -0.33995244481566 0.33995244481566 -0.327297629754157 -0.213058076337577 0.109875921566276 0.000340314793756603 0.000340314793756603 0.000579319798793505 0.0275688143250488 0.259898596375123 greenyellow

ATP5I -0.549575691199896 0.549575691199896 -0.388462718151722 -0.167219538787773 0.275690991406971 8.76339839113036e-10 8.76339839113036e-10 3.55436572176778e-05 0.0851493280828235 0.00405059290537896 yellow

ATP5J -0.577770316852286 0.577770316852286 -0.424090141343034 -0.182884780830085 0.300417846825861 7.23444107047172e-11 7.23444107047172e-11 5.31247051784289e-06 0.0593637121857041 0.00166652724446289 yellow

ATP5J2 -0.0857734551505009 0.0857734551505009 -0.0996780351985459 -0.0418178453648391 0.0725901313066046 0.379701346173856 0.379701346173856 0.307011102423765 0.668888775285821 0.457457622709758 grey

ATP5L -0.461231780658897 0.461231780658897 -0.313852622187646 -0.132431488296383 0.22727081485862 5.738894738495e-07 5.738894738495e-07 0.000994850115025494 0.173896568877164 0.0185631192563461 yellow

ATP5O -0.520967521267768 0.520967521267768 -0.448960558386718 -0.297680006971778 0.141519008591334 8.78338959008121e-09 8.78338959008121e-09 1.23263669579906e-06 0.00184586678947323 0.145941115511139 yellow

ATP5S -0.20213064024006 0.20213064024006 -0.37722230316177 -0.333953104977521 -0.0232953168121525 0.0368068163945167 0.0368068163945167 6.19658531859786e-05 0.000439175575923475 0.811748744305749 black

ATP5SL -0.0753270846893477 0.0753270846893477 -0.199260401527724 -0.201340488515657 -0.0546003914760768 0.440627554058128 0.440627554058128 0.0396248430137781 0.0375652777482959 0.576452478077203 greenyellow

ATP6 0.432126767602616 -0.432126767602616 0.135295377827894 -0.0641236663701175 -0.303563530315983 3.35535315275303e-06 3.35535315275303e-06 0.164691078049194 0.511704866221313 0.00148007850764567 brown

ATP6AP1 -0.216816476679945 0.216816476679945 -0.304720433289324 -0.121339662800787 0.232935316348632 0.0248852676169106 0.0248852676169106 0.00141641845126329 0.213127740816651 0.0157562549949342 purple

ATP6AP1L 0.0267351885301154 -0.0267351885301154 -0.0883197598367832 -0.128784306191589 -0.0912703978656823 0.784582977399821 0.784582977399821 0.365663773362462 0.186161185081612 0.349802398969364 green

ATP6AP2 -0.0105434649505179 0.0105434649505179 -0.169704622529728 -0.11547013731472 0.0484919823302786 0.914166686173892 0.914166686173892 0.0805473566293771 0.236266808699502 0.619890660334194 yellow

ATP6V0A1 -0.432499033972286 0.432499033972286 -0.324905404990606 -0.139422478884069 0.231327238796456 3.28375062195184e-06 3.28375062195184e-06 0.00063898946039129 0.15206840202686 0.0165129935049833 blue

ATP6V0A2 0.590223253876441 -0.590223253876441 0.708610836454087 0.427354172762581 -0.295424852424081 2.22478055526987e-11 2.22478055526987e-11 1.36328184827873e-17 4.41430529836268e-06 0.00200652073920882 blue

ATP6V0A4 0.161678089982935 -0.161678089982935 -0.10813581824967 -0.130226418791438 -0.0651848057912775 0.096165828131257 0.096165828131257 0.267567342687868 0.181238476009658 0.504729265905458 green

ATP6V0B -0.577501678160141 0.577501678160141 -0.295555091158082 -0.0265357281903985 0.380538822419779 7.41679075534807e-11 7.41679075534807e-11 0.00199690938118599 0.786151475231953 5.2703836633761e-05 purple

ATP6V0C 0.318178922299729 -0.318178922299729 -0.151289120085609 -0.25687327399519 -0.217861419823118 0.00083823539334626 0.00083823539334626 0.119819002482189 0.00756522238828476 0.0241800198069168 green

ATP6V0D1 -0.584548052393075 0.584548052393075 -0.434642758095163 -0.183362011486516 0.314802326215688 3.83147253713664e-11 3.83147253713664e-11 2.89872510200818e-06 0.0586917653916841 0.000958344893923983 purple

ATP6V0D2 -0.0205267969708314 0.0205267969708314 -0.211510249719704 -0.194803833422538 -0.0258757504089822 0.833777718981338 0.833777718981338 0.0287434469593123 0.0443566242756385 0.791347416299506 turquoise

ATP6V0E1 -0.755495585995887 0.755495585995887 -0.418524368166617 -0.0942299485693467 0.442774403577369 5.31026708982574e-21 5.31026708982574e-21 7.25330827177998e-06 0.33433258802423 1.7921109946721e-06 brown

ATP6V0E2 0.835117612845791 -0.835117612845791 0.754966827415695 0.393239158113624 -0.420032827835747 5.13835218360332e-29 5.13835218360332e-29 5.85863751759443e-21 2.78932779730643e-05 6.6699066813901e-06 blue

ATP6V0E2-AS1 0.432519524064714 -0.432519524064714 0.370996449012572 0.170239941996185 -0.245419683372851 3.2798517464098e-06 3.2798517464098e-06 8.35811988761009e-05 0.0795825828270718 0.0108368415516522 brown

ATP6V1A 0.0458597471960644 -0.0458597471960644 0.190417720749087 0.190011218615639 0.0481163643026907 0.639032451762974 0.639032451762974 0.0494633734376931 0.0499603308512026 0.62260709408948 purple

ATP6V1B1 -0.192935105225457 0.192935105225457 -0.255943357941203 -0.0744360883981124 0.242259627682557 0.0464760629420203 0.0464760629420203 0.00779373979466306 0.446067696729485 0.0119340376267437 turquoise

ATP6V1B2 0.211093755621711 -0.211093755621711 0.444356756861674 0.452233364071907 0.127252518217428 0.0290666788691773 0.0290666788691773 1.62965226137693e-06 1.00820305482337e-06 0.191495919481842 black

ATP6V1C1 -0.699349565501559 0.699349565501559 -0.594510226579223 -0.304069386592849 0.34024748457436 5.36492206395419e-17 5.36492206395419e-17 1.46518923706803e-11 0.00145193011713063 0.000336028579614008 brown

ATP6V1C2 -0.155960522875859 0.155960522875859 -0.404209354860648 -0.393922577846631 -0.086154880350853 0.108680551192403 0.108680551192403 1.5758740710644e-05 2.69342807578469e-05 0.377578033598146 turquoise

ATP6V1D -0.707464808308911 0.707464808308911 -0.674210405808322 -0.3708354883996 0.341757474119328 1.61980663827344e-17 1.61980663827344e-17 1.72188029651318e-15 8.42235540388537e-05 0.000314860968605649 brown

ATP6V1E1 -0.636917235419606 0.636917235419606 -0.544468867386161 -0.24791529384358 0.363441531990294 1.64879891561168e-13 1.64879891561168e-13 1.34364379892528e-09 0.0100338347177393 0.000119206138834338 brown

ATP6V1E2 0.124957869590057 -0.124957869590057 0.30006061923974 0.199640243305722 -0.093417873931901 0.199693512522849 0.199693512522849 0.00168899450408807 0.0392418527212036 0.338533437188138 turquoise

ATP6V1G1 -0.303878345231853 0.303878345231853 -0.053906125965516 0.154643605647685 0.339911182511569 0.00146250296890041 0.00146250296890041 0.581317621429154 0.111735533573787 0.000340918237038913 green

ATP6V1G2 0.131082103916617 -0.131082103916617 -0.0334279385468146 -0.0817515016144165 -0.090531021307272 0.17836295245848 0.17836295245848 0.732489773850976 0.402528486989155 0.353735980648514 turquoise

ATP6V1G3 -0.0479306652260759 0.0479306652260759 -0.0981232638408149 -0.0932240678105289 -0.0168402105124115 0.623951928046049 0.623951928046049 0.314655076567638 0.339540908088005 0.863309652024816 yellow

ATP6V1H -0.0281707082588608 0.0281707082588608 0.158610116831672 0.155490232210496 0.0353614909984508 0.773320255281222 0.773320255281222 0.10273170615885 0.109763965366929 0.717651760709332 pink

ATP7A 0.00900461835700977 -0.00900461835700977 0.122221964796402 0.116227376518667 0.0211590056695667 0.926656468342252 0.926656468342252 0.209794002209542 0.233186978153478 0.82873526574054 tan

ATP7B -0.0665098520989959 0.0665098520989959 -0.267271434826345 -0.335986545554316 -0.185054027835579 0.496088698659367 0.496088698659367 0.00538550859713777 0.000403039288964281 0.0563593712640377 grey

ATP8A1 0.0262572329388484 -0.0262572329388484 0.158299845465268 0.100811581995926 -0.0569340121554767 0.788342911039047 0.788342911039047 0.103414835923134 0.301515121704465 0.560239707033652 green

ATP8A2 0.292903513478686 -0.292903513478686 0.0875479193539565 -0.0516495140979411 -0.213657833104159 0.00220102811957931 0.00220102811957931 0.369884747212182 0.597260358354764 0.0271247636583799 yellow

ATP8B1 -0.367536145903162 0.367536145903162 -0.0888433961070692 0.0812981572035754 0.265811118842152 9.84473900950547e-05 9.84473900950547e-05 0.36281710002473 0.405151379184709 0.00565325284534196 grey

ATP8B2 0.73909040034799 -0.73909040034799 0.732101247422925 0.339412476183119 -0.478407598888749 1.00217941994713e-19 1.00217941994713e-19 3.27983079411721e-19 0.000348290038095082 1.87001985607626e-07 blue

ATP8B3 -0.100910534061002 0.100910534061002 -0.130088103146007 -0.0456472274118282 0.109880072904225 0.301038437934228 0.301038437934228 0.181706450918801 0.640588524867962 0.259880482354388 green

ATP8B4 -0.583270691130027 0.583270691130027 -0.545364229921604 -0.315449677228496 0.250183480101424 4.32389904196116e-11 4.32389904196116e-11 1.24728626955357e-09 0.00093416699770461 0.00934980767074919 brown

ATP8B5P -0.0983537329980305 0.0983537329980305 -0.111170874845788 -0.0715135686758062 0.0387698842869586 0.313514265479641 0.313514265479641 0.254289911752779 0.464173817152837 0.691752790266991 turquoise

ATP9A -0.679182423517663 0.679182423517663 -0.773379876914513 -0.532466347460253 0.210396679011324 8.91039678952218e-16 8.91039678952218e-16 1.63868315823445e-22 3.56859921840773e-09 0.0296145632299962 brown

ATP9B -0.185985755519312 0.185985755519312 -0.439021872975239 -0.351198242963857 0.0364350833488833 0.0551078321788706 0.0551078321788706 2.24083905334975e-06 0.000208058412614126 0.709457747601256 blue

ATPAF1 0.169728543658728 -0.169728543658728 0.0207963859706854 -0.0778651222708308 -0.162012625135314 0.080504046106409 0.080504046106409 0.831626658880739 0.42533831238834 0.095470362560283 yellow

ATPAF2 0.0755783748327662 -0.0755783748327662 0.0113673325456325 0.056388313053453 0.0792750839193129 0.439100061466914 0.439100061466914 0.907488288161374 0.564011360202497 0.416978308462214 greenyellow

ATPIF1 -0.0659274877169961 0.0659274877169961 -0.0771406152938983 -0.110464822601358 -0.0762941169605397 0.49987666243935 0.49987666243935 0.429671335831481 0.257337578259638 0.434765818286457 yellow

ATR 0.673675226693554 -0.673675226693554 0.748677120977846 0.506520101414919 -0.21883738421236 1.84700763644656e-15 1.84700763644656e-15 1.85065902842319e-20 2.60002871262457e-08 0.0235368214130323 blue

ATRAID 0.0917102227437799 -0.0917102227437799 0.0851702704834769 0.0351744036357102 -0.0629696309196185 0.3474755027486 0.3474755027486 0.383073880547678 0.719082986345056 0.519347041433764 greenyellow

ATRN 0.550704153506516 -0.550704153506516 0.388925982291797 0.149937920721827 -0.305669937236584 7.96591101436482e-10 7.96591101436482e-10 3.47235154985934e-05 0.123199789879316 0.00136604097862039 blue

ATRNL1 -0.191494263888089 0.191494263888089 -0.30168694564418 -0.231538942051203 0.0416551144014325 0.0481669907584259 0.0481669907584259 0.00158888366806821 0.0164116041223632 0.670102098467252 turquoise

ATRX 0.161399211642517 -0.161399211642517 0.441606986274564 0.269484711004844 -0.178754112932682 0.096748637425573 0.096748637425573 1.92167549605188e-06 0.00500120125980695 0.0654464844211971 tan

ATXN1 -0.616951559482025 0.616951559482025 -0.25083834372397 -0.0452178038836936 0.284467368602954 1.48285046451078e-12 1.48285046451078e-12 0.00916010402692708 0.643737521812507 0.00298188511596653 brown

ATXN10 0.65684656395947 -0.65684656395947 0.443133647966627 0.147980271454528 -0.387039791173841 1.55994743248665e-14 1.55994743248665e-14 1.75393505186054e-06 0.128227585517976 3.81775261784156e-05 blue

ATXN1L 0.552645141233114 -0.552645141233114 0.37153373390895 0.116637089704443 -0.337110778085466 6.75462027726099e-10 6.75462027726099e-10 8.14699084736477e-05 0.231532349093677 0.000384255996470951 blue

ATXN2 0.274226977499723 -0.274226977499723 0.275242738572004 0.107463521616002 -0.214028407509395 0.00425894837356472 0.00425894837356472 0.00411339721786742 0.270570843504489 0.0268534486370596 blue

ATXN2L 0.452580120191108 -0.452580120191108 0.207780595535797 -0.00518669393992579 -0.307964517163964 9.8683973114975e-07 9.8683973114975e-07 0.0317494616099071 0.957714566633089 0.00125095265694903 brown

ATXN3 -0.34287783167794 0.34287783167794 -0.180455408897004 -0.059373801270072 0.159117966224508 0.000299958034391968 0.000299958034391968 0.0628824985095313 0.54352580490451 0.101621205570342 yellow

ATXN3L -0.131391473827143 0.131391473827143 -0.254279361781082 -0.292725785983523 -0.130384347814831 0.177331596864466 0.177331596864466 0.00821789743390764 0.00221536108811031 0.180705220394077 turquoise

ATXN7 0.288596412408618 -0.288596412408618 0.638809007289824 0.550476119921647 0.0139083833542249 0.00257302286895408 0.00257302286895408 1.32789095610548e-13 8.12121970048629e-10 0.886932710258892 black

ATXN7L1 0.349741582758118 -0.349741582758118 0.30769900807981 0.216942908426986 -0.0750681765604636 0.000221982084475371 0.000221982084475371 0.00126380268664193 0.0247990139374911 0.442204489066577 brown

ATXN7L2 0.223209888840064 -0.223209888840064 0.164894438224962 0.0444966402038647 -0.161946578402215 0.0208317551819466 0.0208317551819466 0.0896425043872205 0.649040061032068 0.0956073517806403 brown

ATXN7L3 -0.150178355296798 0.150178355296798 -0.344276780672176 -0.234589958732045 0.0978019960223136 0.122592896983553 0.122592896983553 0.000282263625666657 0.0150091694906878 0.316249818779174 brown

ATXN7L3B 0.553816481443369 -0.553816481443369 0.327028924457293 0.0343393842436666 -0.412619489558957 6.11151274297263e-10 6.11151274297263e-10 0.000585757514741738 0.725482699631452 1.00325909078677e-05 blue

ATXN8OS -0.198541213013458 0.198541213013458 -0.23694704930626 -0.161618177862096 0.067035967659725 0.0403585594761604 0.0403585594761604 0.0139981093842147 0.0962908003621858 0.49267962556788 turquoise

AUH -0.413898920158206 0.413898920158206 -0.241292786095191 -0.0280502211137052 0.299841930133164 9.35651130354827e-06 9.35651130354827e-06 0.0122885766305568 0.774263796094327 0.0017028837576261 brown

AUNIP -0.46199776371257 0.46199776371257 -0.473852720894939 -0.342257205714067 0.101750478813473 5.4660293162007e-07 5.4660293162007e-07 2.5327418308415e-07 0.000308131125384135 0.297012089059962 magenta

AUP1 0.030221889829971 -0.030221889829971 -0.0645405880160295 -0.112870117064913 -0.0985155991503134 0.757309049415257 0.757309049415257 0.508958251447785 0.247056922685832 0.312714641828416 greenyellow

AURKA -0.454811172195259 0.454811172195259 -0.717308748311963 -0.586991447849145 0.0371824651315854 8.59320917045708e-07 8.59320917045708e-07 3.58339300961275e-18 3.03587996676643e-11 0.703772908845074 magenta

AURKAIP1 -0.266232520225065 0.266232520225065 -0.232539628493656 -0.0779004106811405 0.202686459228506 0.00557480111044121 0.00557480111044121 0.0159396156125299 0.425127909044937 0.0362810265917562 greenyellow

AURKB -0.572575038543944 0.572575038543944 -0.642175978921099 -0.436924003677589 0.183538757882208 1.16615664732312e-10 1.16615664732312e-10 8.99981654243566e-14 2.53607632262996e-06 0.0584444893467554 magenta

AURKC 0.0610293114757627 -0.0610293114757627 -0.0573345898304524 -0.0908098434143893 -0.0714738238999779 0.532325702474757 0.532325702474757 0.557478764936826 0.352249385270175 0.464422798208875 turquoise

AUTS2 0.796979133645912 -0.796979133645912 0.695623902315799 0.354525048357043 -0.400253772653645 9.90735784766787e-25 9.90735784766787e-25 9.17408187431821e-17 0.000179235031460383 1.94064421802847e-05 blue

AVEN 0.343941385519002 -0.343941385519002 0.308899013392119 0.134370543624877 -0.216849612122413 0.000286415711184575 0.000286415711184575 0.00120666522250856 0.167623383345329 0.0248626373857804 red

AVIL -0.0673162187523249 0.0673162187523249 0.333831471160699 0.339987684918221 0.0960058647482249 0.49086874697666 0.49086874697666 0.000441428851955063 0.00033980020244566 0.325261785230444 black

AVL9 -0.601451759508216 0.601451759508216 -0.410115486480114 -0.229084370069456 0.201936215821508 7.35262443031733e-12 7.35262443031733e-12 1.14909185210115e-05 0.0176208905491084 0.0369922403331106 brown

AVP -0.0496257724649305 0.0496257724649305 -0.219425691377608 -0.205285600816391 -0.0322573553005877 0.611722272072128 0.611722272072128 0.0231562179538275 0.0339053070050932 0.741521331718836 green

AVPI1 0.128051523515886 -0.128051523515886 -0.0479373415980209 -0.0237253582396454 0.0287799177479323 0.188699569328917 0.188699569328917 0.623903556268429 0.808339605697268 0.76855458600397 green

AVPR1A -0.216035916752941 0.216035916752941 -0.425830137289621 -0.295836071648743 0.111342887277132 0.0254234677500553 0.0254234677500553 4.81418343740348e-06 0.00197631527809128 0.253551187537658 turquoise

AVPR1B -0.0904499423443625 0.0904499423443625 -0.215594962667993 -0.149587174286591 0.0566989433770344 0.354169001638976 0.354169001638976 0.0257318728453859 0.124089270139388 0.561862930432849 turquoise

AVPR2 0.0153183160774586 -0.0153183160774586 -0.1668669481399 -0.166424053237586 -0.0420183065506956 0.875558568102385 0.875558568102385 0.0858189138869653 0.0866659264958718 0.667395306306584 green

AWAT1 -0.0415823844378954 0.0415823844378954 -0.292368161662729 -0.3022261163878 -0.0916570379508883 0.670644648224134 0.670644648224134 0.00224445774216377 0.00155690219120428 0.347756360255298 turquoise

AX746627 0.273181205698833 -0.273181205698833 -0.00763610439067722 -0.126959988044951 -0.204346226421039 0.00441359260484368 0.00441359260484368 0.937778856103166 0.192527190726077 0.0347483439687816 green

AX746699 -0.240580695107421 0.240580695107421 -0.275170899887587 -0.198132656447453 0.0601381544009737 0.0125555472945966 0.0125555472945966 0.00412354304551566 0.0407804027616219 0.538340354007557 turquoise

AX746710 -0.037123576501005 0.037123576501005 -0.12532771351572 -0.12184697680238 -0.0262188412879649 0.704220248857505 0.704220248857505 0.198355446940422 0.211206302039959 0.788645138924412 green

AX746823 -0.249779161182419 0.249779161182419 -0.329495262895399 -0.14444068106349 0.229424265658358 0.00946864972842606 0.00946864972842606 0.000529054931695914 0.137715374779068 0.0174489736083372 brown

AX746830 -0.107887087757414 0.107887087757414 -0.157187400919859 -0.147260103237358 -0.0234505498350038 0.268675908339342 0.268675908339342 0.105893416941891 0.130116227395181 0.810517730501074 grey

AX746968 -0.0112811219359767 0.0112811219359767 0.00746331836425391 0.00864472748814037 0.00391672678058455 0.908186830953492 0.908186830953492 0.939184043959173 0.92958014574357 0.968061860760504 turquoise

AX747031 -0.128548614315167 0.128548614315167 -0.336744336839994 -0.265828370244423 0.0339721331483283 0.186974901367536 0.186974901367536 0.000390287900214621 0.00565002200673457 0.728303391291183 turquoise

AX747064 0.305154526892591 -0.305154526892591 0.281613832510133 0.152213352392221 -0.147300060712215 0.00139318108833866 0.00139318108833866 0.00329764955737318 0.117548075900768 0.130010884418368 brown

AX747191 -0.0969851175414558 0.0969851175414558 -0.185694889336542 -0.175475900784803 -0.0302625567377298 0.320328231069346 0.320328231069346 0.0554960613952286 0.0706264121131649 0.756992620208855 turquoise

AX747250 -0.20077119913023 0.20077119913023 -0.227885053289766 -0.147018193975151 0.0787518148369556 0.0381198224012255 0.0381198224012255 0.0182392394257282 0.130755386220538 0.420069693531184 turquoise

AX747261 -0.188191710204667 0.188191710204667 -0.251048634000058 -0.183629935373295 0.0500049045964194 0.0522355434212372 0.0522355434212372 0.00909990873500056 0.0583172618555935 0.609001332219398 turquoise

AX747405 0.0948639852518916 -0.0948639852518916 0.0532097397875523 -0.0507726828916575 -0.162729717796529 0.331075856260715 0.331075856260715 0.58621655695384 0.603507503560496 0.0939929742462489 grey

AX747444 -0.332300848559207 0.332300848559207 -0.302765711957978 -0.193487216090194 0.107749108204068 0.0004707074568939 0.0004707074568939 0.0015254815159204 0.0458413111680204 0.269292207369488 blue

AX747507 0.0255312571191684 -0.0255312571191684 0.128049969309742 0.0516250112251178 -0.0968064199491299 0.794063228356294 0.794063228356294 0.188704979754883 0.597434539342199 0.32122490833941 grey

AX747630 -0.153556648443135 0.153556648443135 -0.221082145187734 -0.160096114686591 0.0467749319550541 0.114307009866741 0.114307009866741 0.0221127525861733 0.0995088803342224 0.632349402537753 turquoise

AX747652 0.377857109111125 -0.377857109111125 0.514223732501781 0.290877697015426 -0.247023997826648 6.0083209463912e-05 6.0083209463912e-05 1.46687653299498e-08 0.00236947580210779 0.0103144187496031 blue

AX747730 0.02589037033928 -0.02589037033928 0.0655994264186653 -0.00978534196092801 -0.111048753209938 0.791232215062058 0.791232215062058 0.502017165666776 0.92031744100707 0.254815270173715 grey

AX747826 0.231253927439843 -0.231253927439843 0.186450871965393 0.0918389167230535 -0.112685266453495 0.0165482301136699 0.0165482301136699 0.0544916641935509 0.346796486017215 0.247836799639594 brown

AX748157 -0.0314124274708997 0.0314124274708997 -0.280675952335849 -0.26330626040807 -0.0424785409434676 0.748062275196677 0.748062275196677 0.00340780392803605 0.0061401620141905 0.663971401027415 green

AX748267 -0.074003564197596 0.074003564197596 -0.147002353488506 -0.0497101045508581 0.127342458079999 0.448722085193924 0.448722085193924 0.130797322634286 0.611116582397457 0.191179656519998 turquoise

AX748273 -0.0795791917482414 0.0795791917482414 -0.196989975777964 -0.156904811583416 0.0174993162390433 0.415187782978795 0.415187782978795 0.0419798430825998 0.106530377793238 0.858014719180817 grey

AX748292 -0.134756971340366 0.134756971340366 -0.11811063330365 -0.058613954165953 0.0706413548662468 0.166393498573001 0.166393498573001 0.225649429248372 0.548704853325857 0.469654599730051 turquoise

AX748339 0.402433937287082 -0.402433937287082 0.575287703055222 0.411257084531438 -0.130765988196767 1.73081047366081e-05 1.73081047366081e-05 9.09793285940066e-11 1.08029224416118e-05 0.179421334082995 blue

AXDND1 -0.219990785649287 0.219990785649287 -0.242969086471799 -0.211781831302682 -0.00937754324385072 0.0227956039527807 0.0227956039527807 0.0116795978001372 0.0285343257374248 0.923627935284836 turquoise

AXIN1 0.318897969215333 -0.318897969215333 0.127116439060129 -0.0147196946736048 -0.207991681369437 0.000814506463248171 0.000814506463248171 0.191975146781034 0.880384781974402 0.0315725073435283 grey

AXIN2 0.479957503052028 -0.479957503052028 0.242739126563479 -0.056855308895284 -0.445935366250604 1.68492230582331e-07 1.68492230582331e-07 0.0117615459405203 0.560782928149159 1.48154695828674e-06 brown

AXL 0.159380350049909 -0.159380350049909 -0.0235711466085671 0.0548137285012699 0.126909615774174 0.101051160600508 0.101051160600508 0.809561704253803 0.574961306417656 0.192705175894836 green

AY927499 -0.0787431680066646 0.0787431680066646 0.0436396724333327 0.140243345861003 0.175037821334765 0.420120888757028 0.420120888757028 0.655363985894203 0.149646751293704 0.0713430618964498 grey

AY940074 -0.388761806317248 0.388761806317248 -0.0344300952392528 0.152152064445974 0.307643181871635 3.50121173570324e-05 3.50121173570324e-05 0.724786552210313 0.117697627947688 0.00126651978131059 grey

AZGP1 -0.301052985094686 0.301052985094686 -0.28592078807331 -0.192517681167624 0.0851398192867544 0.00162724944927143 0.00162724944927143 0.00283174253911804 0.0469607938253519 0.383244617906411 blue

AZGP1P1 -0.383096733278253 0.383096733278253 -0.395061570150107 -0.232447678283291 0.17455668564997 4.64617016450347e-05 4.64617016450347e-05 2.54043567036538e-05 0.0159824889062488 0.0721369037239745 red

AZI2 -0.628436178113651 0.628436178113651 -0.573001140037194 -0.268366122963868 0.369835650767114 4.2732131750597e-13 4.2732131750597e-13 1.1217255922656e-10 0.00519226307708257 8.83177761052699e-05 brown

AZIN1 -0.217665693153593 0.217665693153593 -0.428990659801616 -0.389274833610062 -0.0425910006394421 0.024310803454161 0.024310803454161 4.01993752124288e-06 3.41176523752448e-05 0.663135805875936 yellow

AZIN2 -0.0205121198681965 0.0205121198681965 -0.208214369904485 -0.253039487407283 -0.129396567403562 0.833894863455325 0.833894863455325 0.0313867308929993 0.00854705410683542 0.184059439949734 green

AZU1 -0.474366828342911 0.474366828342911 -0.289576462011153 -0.125963749537498 0.203287555644613 2.44802763672825e-07 2.44802763672825e-07 0.00248372214293642 0.196069454479629 0.0357195216477127 magenta

B2M -0.147204025119838 0.147204025119838 0.390148138084617 0.524986883588342 0.328702834727672 0.130264180570738 0.130264180570738 3.26440614145995e-05 6.43555950238439e-09 0.000546698513616425 tan

B3GALNT1 -0.169938738635155 0.169938738635155 -0.381355078781839 -0.375091730403369 -0.0871213877340106 0.0801242776581136 0.0801242776581136 5.06315436872546e-05 6.86945404121826e-05 0.372230074952287 brown

B3GALNT2 0.246363997076748 -0.246363997076748 0.401095692518519 0.287539905255475 -0.0898010797763818 0.0105266022859828 0.0105266022859828 1.85693096808414e-05 0.00267252229630293 0.357646283069394 yellow

B3GALT1 -0.203718606572138 0.203718606572138 -0.265736740398993 -0.111273882391993 0.193878590720835 0.0353213752585415 0.0353213752585415 0.00566720123756388 0.253847358671898 0.0453957281803656 turquoise

B3GALT2 0.520753617933656 -0.520753617933656 0.467458194639812 0.283585403240405 -0.192058654670414 8.92897408662901e-09 8.92897408662901e-09 3.8490671039872e-07 0.00307644770577511 0.0474986672514413 blue

B3GALT4 -0.110711363390662 0.110711363390662 -0.294065405489321 -0.1739008452132 0.128443262589906 0.256270561989562 0.256270561989562 0.00210937209208155 0.0732304637951325 0.187339459903895 grey

B3GALT5 -0.193630390781364 0.193630390781364 -0.311925437484573 -0.26267276597934 0.00358962453017711 0.0456778863015511 0.0456778863015511 0.00107285609737934 0.00626905504472912 0.970727914907997 turquoise

B3GALT6 0.573329796031419 -0.573329796031419 0.598170046174562 0.419380151664012 -0.149934691392383 1.08857392938047e-10 1.08857392938047e-10 1.02072150703206e-11 6.91668203378911e-06 0.12320795689922 blue

B3GALTL 0.638016761329433 -0.638016761329433 0.498276961768667 0.216334726769178 -0.350498206021965 1.45415086759687e-13 1.45415086759687e-13 4.72338261304906e-08 0.0252162753878976 0.000214645673504852 blue

B3GAT1 0.256358752427353 -0.256358752427353 0.298926110098105 0.223543824198971 -0.0512407968039872 0.00769091818849699 0.00769091818849699 0.00176218045927122 0.0206366138049328 0.600168734829515 brown

B3GAT2 -0.0268779641096614 0.0268779641096614 -0.0372523269082678 0.0177180867764312 0.0836889359738757 0.783460760895843 0.783460760895843 0.703242343737423 0.856258602500026 0.3914327530126 grey

B3GAT3 0.0827405890145283 -0.0827405890145283 -0.0934241375491078 -0.0546382136646065 0.0418406024484308 0.396840916369484 0.396840916369484 0.33850090833539 0.576187979495698 0.668719166127701 grey

B3GNT1 0.504740162221583 -0.504740162221583 0.124836239692274 -0.129414199346627 -0.399245552332147 2.96172416056834e-08 2.96172416056834e-08 0.200134976426403 0.183999171342466 2.04555269799879e-05 brown

B3GNT2 -0.552307923984161 0.552307923984161 -0.425997797284981 -0.235428644187635 0.214043233816722 6.95151219585632e-10 6.95151219585632e-10 4.76857426835934e-06 0.0146423813036204 0.0268426418202713 brown

B3GNT3 0.0889313073699833 -0.0889313073699833 -0.0591228168543352 -0.0458244135668796 0.00740213129556491 0.362340527289455 0.362340527289455 0.545233836508483 0.639291056850694 0.939681694718598 green

B3GNT4 -0.00272329353517036 0.00272329353517036 -0.179484308135991 -0.240321828271462 -0.149191960122157 0.977790444927596 0.977790444927596 0.064335803896475 0.0126538461398871 0.125097427463031 turquoise

B3GNT5 -0.651540442167143 0.651540442167143 -0.621825908943786 -0.352586714216683 0.297285441846451 2.97311857792885e-14 2.97311857792885e-14 8.79960518457394e-13 0.00019554324966731 0.00187310569825489 brown

B3GNT6 -0.452705129172296 0.452705129172296 -0.292091925530565 -0.124833468271887 0.208826522813015 9.7924389916164e-07 9.7924389916164e-07 0.00226716832616874 0.200145043660679 0.0308808026602367 turquoise

B3GNT7 0.514370570456676 -0.514370570456676 0.161724189059396 -0.055977365940233 -0.327799133473988 1.45075889568871e-08 1.45075889568871e-08 0.0960697560012809 0.566859594386237 0.000567477995617258 brown

B3GNT8 -0.648704365006243 0.648704365006243 -0.497449711938262 -0.176785635083824 0.416387605101086 4.17503413515419e-14 4.17503413515419e-14 5.01068514836962e-08 0.0685184787225667 8.16246801033471e-06 blue

B3GNT9 -0.220925000161647 0.220925000161647 -0.104590591531703 -0.0836006106082593 0.00879425622911737 0.0222099825590542 0.0222099825590542 0.28366174474432 0.391934578172417 0.928365289825939 grey

B3GNTL1 -0.455842039032085 0.455842039032085 -0.505092140687686 -0.231777248659648 0.334118315638857 8.05834531008322e-07 8.05834531008322e-07 2.88658491035908e-08 0.0162981190831105 0.000436131980721223 blue

B4GALNT1 -0.0411222645904478 0.0411222645904478 -0.109543715875687 0.00558854975445404 0.167202439614612 0.674080956521586 0.674080956521586 0.261350941885135 0.95444175184824 0.0851817038455371 turquoise

B4GALNT2 -0.038624044887573 0.038624044887573 -0.0407834393663707 0.0349663377211476 0.118028434854267 0.692853969710489 0.692853969710489 0.676615697895767 0.720675839690018 0.225974798879186 yellow

B4GALNT3 -0.30055804012349 0.30055804012349 -0.449821052496078 -0.308019654405517 0.125220589967496 0.00165778430773529 0.00165778430773529 1.16942716459634e-06 0.00124829909682771 0.198742344745817 turquoise

B4GALNT4 -0.0479150469476569 0.0479150469476569 -0.325757856626263 -0.284388111856931 -0.0133262066278222 0.624065092136894 0.624065092136894 0.000617109071915216 0.00299027469997526 0.891636058688935 green

B4GALT1 -0.226553146076976 0.226553146076976 -0.0985218534115935 -0.0167135805059814 0.113505554912766 0.0189478087517443 0.0189478087517443 0.312683772179522 0.864327636442878 0.244388965677196 turquoise

B4GALT2 -0.0911251144636602 0.0911251144636602 -0.361918739429156 -0.300555780316255 0.0113165794169131 0.350573158165487 0.350573158165487 0.00012791394319575 0.00165792490664264 0.907899519439372 green

B4GALT3 0.0713024204350733 -0.0713024204350733 -0.142034197026039 -0.230936057830095 -0.187193949300366 0.465497393518314 0.465497393518314 0.144464341479735 0.0167017659358198 0.0535190261974584 pink

B4GALT4 -0.458519349697759 0.458519349697759 -0.505030801731099 -0.291153047231767 0.233320792046793 6.81291057147379e-07 6.81291057147379e-07 2.89954687120884e-08 0.00234591709941734 0.0155793878848087 brown

B4GALT5 -0.732947834537004 0.732947834537004 -0.42932937596171 -0.0875898214782813 0.469594230176043 2.84670069811964e-19 2.84670069811964e-19 3.94258093883311e-06 0.369654833366468 3.35003245403302e-07 brown

B4GALT6 0.0352067681332455 -0.0352067681332455 -0.0203143920761903 -0.0423550046688982 -0.0425906062877995 0.71883532704439 0.71883532704439 0.835473370020713 0.664889771916351 0.663138735256554 grey

B4GALT7 0.548759322069919 -0.548759322069919 0.401778164881181 0.194752814776613 -0.248162813035687 9.38758276495687e-10 9.38758276495687e-10 1.7915722017405e-05 0.0444134014051278 0.0099571129654726 blue

B9D1 -0.260525727136434 0.260525727136434 -0.458963615920006 -0.398658759132721 -0.0153516088991948 0.00672393597736062 0.00672393597736062 6.62481242453875e-07 2.10903647180804e-05 0.875290286417344 turquoise

B9D2 -0.441579696962378 0.441579696962378 -0.255095521379715 -0.0594360774762678 0.266480863438239 1.92480770805243e-06 1.92480770805243e-06 0.00800737700203675 0.543102401613863 0.00552902215872575 brown

BAALC 0.0537942845314897 -0.0537942845314897 -0.0827882967834387 -0.116461437298211 -0.0783334712707216 0.582103129723968 0.582103129723968 0.396567795130863 0.232240713101214 0.422550715653496 grey

BAALCOS -0.116153929241859 0.116153929241859 -0.230853549009309 -0.103394130857872 0.157018080614367 0.233484466827554 0.233484466827554 0.0167418198864175 0.289236234262801 0.106274709092509 turquoise

BAAT -0.0230326752851525 0.0230326752851525 -0.203706722703934 -0.221253439417539 -0.0819734692830411 0.813832561968403 0.813832561968403 0.0353323017164548 0.0220071839878118 0.401247927412673 green

BABAM1 0.278698436896044 -0.278698436896044 -0.124368997217847 -0.271498841546594 -0.281428530719945 0.00365094921853271 0.00365094921853271 0.201837395327709 0.00467291645455378 0.00331915648513879 pink

BACE1 0.116441730098178 -0.116441730098178 0.00243449109234266 -0.014438838183814 -0.0279954164520251 0.232320281997015 0.232320281997015 0.980145237451715 0.882650628374215 0.774693082980647 yellow

BACE2 0.249506207373665 -0.249506207373665 0.0642032108962248 -0.129779490978228 -0.312564335300706 0.00954962832842545 0.00954962832842545 0.5111802513453 0.182753788572926 0.00104639856190033 brown

BACH1 -0.514995857589287 0.514995857589287 -0.0816596889314331 0.211219022725199 0.475830742497101 1.38400260042055e-08 1.38400260042055e-08 0.403058871217469 0.0289691391746483 2.22133093486515e-07 brown

BACH2 0.787968462579861 -0.787968462579861 0.675828597397433 0.274334924338236 -0.507765076912509 7.5209830795425e-24 7.5209830795425e-24 1.39154611234337e-15 0.00424326472694052 2.37257724983078e-08 blue

BAD -0.719046900825861 0.719046900825861 -0.467218489882861 -0.190701894022393 0.349256170392404 2.72723143569737e-18 2.72723143569737e-18 3.90928682606876e-07 0.049118398574773 0.000226810202076673 brown

BAG1 0.376950663810101 -0.376950663810101 -0.0523109844738419 -0.254180357725117 -0.355804843912671 6.27881486022333e-05 6.27881486022333e-05 0.592566861531617 0.00824376367484229 0.000169168269638116 pink

BAG2 0.345931668244853 -0.345931668244853 0.153628662591273 -0.152181491892356 -0.479317862118719 0.000262578373464694 0.000262578373464694 0.114135234188597 0.117625802081428 1.75909068463362e-07 turquoise

BAG3 0.662210511311411 -0.662210511311411 0.546701297366363 0.286815632359936 -0.300675747613166 8.02048601327942e-15 8.02048601327942e-15 1.11564928875027e-09 0.00274272106440737 0.00165047580764069 blue

BAG4 -0.562513003936488 0.562513003936488 -0.446332509323533 -0.241915131743827 0.232319674968164 2.8722222835121e-10 2.8722222835121e-10 1.44634716669886e-06 0.0120593250178372 0.0160423389136313 brown

BAG5 0.494373966631327 -0.494373966631327 0.477014100530515 0.250008483553982 -0.262768330147152 6.23231927563229e-08 6.23231927563229e-08 2.052836827069e-07 0.00940108271886796 0.00624945875470135 blue

BAG6 0.215106363520528 -0.215106363520528 -0.154443896999924 -0.277116529765538 -0.247654341620035 0.0260773201122269 0.0260773201122269 0.112204591680699 0.00385652436760631 0.0101152816499711 pink

BAGE -0.400809410442193 0.400809410442193 -0.412205406275875 -0.256005307722462 0.1592839020836 1.88500841581229e-05 1.88500841581229e-05 1.02610736077186e-05 0.00777832890721997 0.10126040718289 blue

BAHCC1 -0.0783011540735738 0.0783011540735738 -0.333354118724253 -0.233197515309997 0.0844370743123822 0.422742726951656 0.422742726951656 0.000450374998477726 0.0156357625224826 0.387197603770222 green

BAHD1 0.713050821225279 -0.713050821225279 0.567769214279546 0.316979324124875 -0.279848198373883 6.93505334982856e-18 6.93505334982856e-18 1.80040206088731e-10 0.000879233261926718 0.00350775462110382 blue

BAI1 -0.0426200603109657 0.0426200603109657 -0.222360693320966 -0.202373189974038 -0.0230916675260576 0.66291995454266 0.66291995454266 0.0213351539050516 0.0365765894571719 0.813364399426567 turquoise

BAI2 0.0348841805454308 -0.0348841805454308 -0.207345813632819 -0.222823477816149 -0.0793967946459841 0.721305125398511 0.721305125398511 0.0321165836248603 0.0210595381897204 0.416261162255207 turquoise

BAI3 -0.163505564521445 0.163505564521445 -0.238136609328041 -0.177931611049782 0.041078981394176 0.0924149504956918 0.0924149504956918 0.0135107374672198 0.0667161795804588 0.674404554476934 turquoise

BAIAP2 0.118235707039059 -0.118235707039059 0.0487028819185871 0.10068629616712 0.10065373392066 0.225154977209625 0.225154977209625 0.618367689872176 0.302119373870566 0.302276551168039 green

BAIAP2-AS1 0.320877354930484 -0.320877354930484 0.416810496657139 0.292974747328723 -0.103209385728129 0.000752323099516247 0.000752323099516247 7.97442254524109e-06 0.00219530696548639 0.290103427859742 brown

BAIAP2L1 -0.00344752321777892 0.00344752321777892 -0.22880727295402 -0.227646520453777 -0.0566766121095528 0.971886216999602 0.971886216999602 0.0177621252629436 0.0183644308297198 0.562017251268788 turquoise

BAIAP2L2 0.0770012896851547 -0.0770012896851547 -0.210041999606714 -0.233784961691687 -0.094106869948561 0.430507481586818 0.430507481586818 0.0298966759200157 0.0153687008180895 0.334967133095923 green

BAIAP3 0.159605499333706 -0.159605499333706 0.0765859581311883 0.0171482542421548 -0.0811844773234279 0.100564014240598 0.100564014240598 0.433005559168186 0.86083421134307 0.405810666763797 yellow

BAK1 -0.0486176441169618 0.0486176441169618 0.122648361784311 0.262812686303523 0.269173054416442 0.618983024056556 0.618983024056556 0.208196286303772 0.00624038160873664 0.00505379422288814 cyan

BAMBI -0.529285800289622 0.529285800289622 -0.547192188863562 -0.363240119373729 0.17175651103912 4.59363400434511e-09 4.59363400434511e-09 1.07075603539875e-09 0.0001203253907878 0.0768995604738893 blue

BANCR -0.0281866450325138 0.0281866450325138 -0.158313896161352 -0.105305783847604 0.0493314388646075 0.7731954783041 0.7731954783041 0.103383823522957 0.280364028398867 0.613838283183389 green

BANF1 0.362900793668241 -0.362900793668241 0.277087303312596 0.146686826866952 -0.150156355316652 0.000122233136464403 0.000122233136464403 0.00386041806557064 0.1316347978705 0.12264833235833 red

BANF2 -0.0539610816719744 0.0539610816719744 -0.0841003952629015 -0.0390465370934123 0.0548616068633985 0.580931824096439 0.580931824096439 0.389100096702503 0.689665653050678 0.574626896011383 turquoise

BANK1 0.609633167524003 -0.609633167524003 0.433673551797619 0.0492559822666234 -0.540868326473428 3.19240844185008e-12 3.19240844185008e-12 3.0671898102682e-06 0.614381264513152 1.80838966978196e-09 blue

BANP 0.566042369785407 -0.566042369785407 0.72284570945024 0.493619581538776 -0.20352568297064 2.10089783651445e-10 2.10089783651445e-10 1.49090740162233e-18 6.57275867589447e-08 0.0354991080433094 black

BAP1 0.316766110851882 -0.316766110851882 -0.0372333940073701 -0.122881259196214 -0.154813321993025 0.000886709025741637 0.000886709025741637 0.703386115554305 0.207327296800416 0.111338118427453 red

BARD1 -0.314868115080889 0.314868115080889 -0.449907539038374 -0.35137143101281 0.0518147495828374 0.000955861928478127 0.000955861928478127 1.1632469324622e-06 0.000206457865291709 0.596086361043273 brown

BARHL1 -0.0863299403975004 0.0863299403975004 -0.0980028250915506 -0.0709293673648271 0.0208011377688032 0.376605931325483 0.376605931325483 0.315252311200356 0.467840909686094 0.831588755213214 turquoise

BARX1 -0.127818992919728 0.127818992919728 -0.236627224036 -0.149099834558115 0.087808259354515 0.189510297461499 0.189510297461499 0.014131725085255 0.125333333841719 0.368457699307879 green

BARX1-AS1 -0.276661814544494 0.276661814544494 -0.168576573716555 -0.0401901549931361 0.174552542643637 0.00391750386163925 0.00391750386163925 0.0826110199047125 0.681062735319272 0.0721437702426315 turquoise

BARX2 0.0478520056448865 -0.0478520056448865 -0.123130238158209 -0.0828249585339995 0.0368036694475644 0.624521954227523 0.624521954227523 0.206401175658894 0.396357987022117 0.706652141195861 green

BASP1 -0.643322143567888 0.643322143567888 -0.556396267685601 -0.298868166235086 0.294192117868918 7.87498537514294e-14 7.87498537514294e-14 4.89613489532348e-10 0.00176599434729731 0.00209958793830124 blue

BATF -0.600046794534662 0.600046794534662 -0.539778429124545 -0.280231024465434 0.301876392756466 8.46510778789265e-12 8.46510778789265e-12 1.97717164710078e-09 0.00346120639982456 0.00157757911586119 brown

BATF2 -0.0755433116776826 0.0755433116776826 0.146352651459567 0.435414282116178 0.52779810333069 0.439313016271273 0.439313016271273 0.132526231136279 2.77091520321572e-06 5.16489554998793e-09 cyan

BATF3 0.296472725675149 -0.296472725675149 0.107457940725852 0.152120315100544 0.103295723439055 0.00193036167732568 0.00193036167732568 0.270595871213386 0.117775159091826 0.289697944204248 cyan

BAX 0.21484322965372 -0.21484322965372 0.239166882346409 0.221666965117387 0.0316185341566526 0.0262649913536131 0.0262649913536131 0.0131006359786086 0.0217541075114702 0.746465046831032 turquoise

BAZ1A -0.680452182746861 0.680452182746861 -0.577997267536874 -0.299757409436364 0.323785409835795 7.51503590471926e-16 7.51503590471926e-16 7.0837623020218e-11 0.00170827946849257 0.000668818165458997 brown

BAZ1B 0.665540913915877 -0.665540913915877 0.253222514467082 -0.0439814649719059 -0.439193832020685 5.27026268953506e-15 5.27026268953506e-15 0.0084977454569568 0.652838802140379 2.21813928143934e-06 brown

BAZ2A 0.258131816753484 -0.258131816753484 0.358822390592534 0.259649752697586 -0.0762403748384852 0.00726538482071191 0.00726538482071191 0.000147470906865686 0.00691776724049221 0.435090410833759 yellow

BAZ2B 0.0204646227730546 -0.0204646227730546 0.312451217666595 0.304941984618231 0.0673474662199609 0.834273983761159 0.834273983761159 0.00105103911285568 0.0014045149969386 0.490667056108047 tan

BBIP1 0.329627868816596 -0.329627868816596 0.653821749946352 0.507437700417583 -0.0807061400581756 0.000526154031233456 0.000526154031233456 2.25667204849247e-14 2.43047074729267e-08 0.408591700419329 blue

BBOX1 -0.173000462153109 0.173000462153109 -0.18319738723226 -0.072895943982209 0.140130633538318 0.0747535085196862 0.0747535085196862 0.0589228515911761 0.45555964906059 0.149977537149196 turquoise

BBS1 0.36550875879242 -0.36550875879242 0.104309304732453 -0.0323406053081875 -0.205040817262855 0.000108264624098253 0.000108264624098253 0.284965809146417 0.740877844597565 0.0341233089608447 yellow

BBS10 0.0699926582430585 -0.0699926582430585 0.344599815581475 0.304910941548688 0.0210065534794968 0.473753605580601 0.473753605580601 0.000278317171385992 0.00140617737649685 0.82995058593543 tan

BBS12 0.195092798114291 -0.195092798114291 0.0813802626447955 0.0342137995787717 -0.0591420682670457 0.0440361822252177 0.0440361822252177 0.404675600595163 0.72644684864531 0.545102731377914 turquoise

BBS2 0.667771184603226 -0.667771184603226 0.653255059508976 0.383572184166972 -0.28998241134347 3.96642126381703e-15 3.96642126381703e-15 2.41718039809803e-14 4.53804579440904e-05 0.00244755844098435 blue

BBS4 0.452348201043088 -0.452348201043088 0.427673717489204 0.298977269422678 -0.108669373467688 1.00107993620279e-06 1.00107993620279e-06 4.33453910508479e-06 0.00175881933736423 0.265199809117361 blue

BBS5 -0.0499311127517141 0.0499311127517141 -0.135248422726116 -0.256199613598087 -0.239814624731572 0.609530502365613 0.609530502365613 0.164839031701031 0.00773016698703287 0.0128483933622628 grey

BBS7 -0.167109709940888 0.167109709940888 -0.195084790628988 -0.140725507833372 0.0421984355920241 0.0853574501277923 0.0853574501277923 0.0440450359588957 0.148237902992692 0.66605442172373 yellow

BBS9 0.459938596367182 -0.459938596367182 0.276568595724823 0.0496553583860407 -0.313987687725227 6.22915575102834e-07 6.22915575102834e-07 0.00393011104120731 0.611509750323195 0.000989582150189439 blue

BBX 0.564220690536544 -0.564220690536544 0.781507166504995 0.601243978938164 -0.105442638907878 2.47004138152297e-10 2.47004138152297e-10 3.03129882131645e-23 7.50776349658402e-12 0.279735934274359 blue

BC010186 0.119576929091036 -0.119576929091036 -0.0686523795943152 -0.121172542270242 -0.106677354934515 0.219900647958595 0.219900647958595 0.482283661335111 0.213763420492339 0.274111852022199 grey

BC012193 -0.150478730632114 0.150478730632114 -0.0851930023279643 -0.0388899518937748 0.0567003539560768 0.121837942370795 0.121837942370795 0.382946454908055 0.690846685932013 0.561853183265577 grey

BC015159 -0.0354036611088757 0.0354036611088757 -0.280195095902116 -0.261967141494721 -0.0408995863726557 0.717329292366702 0.717329292366702 0.00346555130869095 0.00641544496554796 0.675746397004647 turquoise

BC016361 -0.248294814920274 0.248294814920274 -0.34278685001256 -0.20672698443851 0.142915991538043 0.00991640824125132 0.00991640824125132 0.000301143531495278 0.0326452911698193 0.14196293590745 salmon

BC017209 0.151394281931419 -0.151394281931419 0.105205248545619 0.134853727188754 0.0772528217682279 0.119558915007943 0.119558915007943 0.280826034698229 0.166086602924161 0.428998618797952 yellow

BC021061 -0.249225877888828 0.249225877888828 -0.341983980792087 -0.234238338949163 0.0950971776208956 0.00963342802959266 0.00963342802959266 0.000311793993794613 0.0151653032602795 0.329883167165631 blue

BC022047 0.348742382928313 -0.348742382928313 0.51262794415159 0.411387006387354 -0.0403267110420844 0.000232026239055536 0.000232026239055536 1.65346173128947e-08 1.07271285202148e-05 0.680038187043942 black

BC022892 -0.514423498140348 0.514423498140348 -0.144968451463508 0.0198970492391484 0.242476858061302 1.44499097212434e-08 1.44499097212434e-08 0.136267806284967 0.838807251971081 0.0118556206469874 brown

BC023201 -0.222729685051702 0.222729685051702 -0.204003763738669 -0.0220045848048292 0.256406544063068 0.0211151489854112 0.0211151489854112 0.0350600425078777 0.822001877177412 0.0076791656406238 turquoise

BC024169 -0.122989377563157 0.122989377563157 -0.176519489874087 -0.198826809433432 -0.0830797246659163 0.206924767091082 0.206924767091082 0.0689426447528067 0.0400658459618202 0.394901834814411 turquoise

BC027448 -0.00666556944751031 0.00666556944751031 -0.288620585684869 -0.280821584764537 -0.0607479747329579 0.94567411563476 0.94567411563476 0.00257078587968768 0.00339048567786083 0.534220889342278 green

BC028044 0.123090188381707 -0.123090188381707 -0.0103463324370014 -0.0512124391218949 -0.0719661906680103 0.206549947690983 0.206549947690983 0.915765576554211 0.600370758257242 0.461343539858488 yellow

BC028670 -0.170479412375373 0.170479412375373 -0.30579253357497 -0.235271402767128 0.0412357004464782 0.0791540080244836 0.0791540080244836 0.00135965655625684 0.0147105495809884 0.673233158628943 turquoise

BC030152 -0.0133656060591231 0.0133656060591231 -0.107587609557842 -0.0306631706854553 0.102898341641268 0.891317634113839 0.891317634113839 0.270014770500205 0.753877596475981 0.291567361744482 grey

BC032415 -0.152356237613911 0.152356237613911 0.121788696677315 0.103653301568766 0.000455564683715991 0.117199984759291 0.117199984759291 0.211426406702696 0.28802259075298 0.99628422683281 purple

BC033164 0.0461828397377217 -0.0461828397377217 -0.00878595042582312 -0.0294304959321243 -0.0372677774893479 0.636669762520189 0.636669762520189 0.92843276657093 0.763474807622846 0.703125023554924 yellow

BC033241 -0.110941972357029 0.110941972357029 -0.282678192833805 -0.254121212779535 -0.0240166506095975 0.255275242941497 0.255275242941497 0.00317652068465011 0.00825925038317032 0.806032427057227 grey

BC034416 -0.155299035661913 0.155299035661913 -0.153121660896796 -0.104096038880421 0.0439074357236945 0.110206825151406 0.110206825151406 0.115348855557631 0.285957183501026 0.653385403406361 turquoise

BC034444 -0.0940190953358622 0.0940190953358622 -0.117186322716105 -0.0747094923719725 0.0420103527039534 0.335420131416462 0.335420131416462 0.229327186911765 0.444394381316342 0.667454539095402 turquoise

BC034788 -0.298014330500369 0.298014330500369 -0.290678329861653 -0.232096800075117 0.0248584518252084 0.00182306795046998 0.00182306795046998 0.00238666648362024 0.016147011136199 0.799374361439306 blue

BC035400 -0.0320142857855478 0.0320142857855478 -0.0301448771116492 -0.00413247581592868 0.0363940295280592 0.743401173805127 0.743401173805127 0.757908395194329 0.966303584181664 0.709770482688555 grey

BC036209 -0.0445464139507449 0.0445464139507449 -0.0717354382849652 -0.0428470923470264 0.0306119867832081 0.648673519183917 0.648673519183917 0.462785261922272 0.661234541876379 0.754275364592509 grey

BC036311 -0.162766812737434 0.162766812737434 -0.125078953232487 0.0082373798692117 0.194063214234344 0.0939170438214782 0.0939170438214782 0.199254728068802 0.932890480318447 0.0451867852190504 turquoise

BC038205 0.00711698543205873 -0.00711698543205873 -0.0239360372453206 -0.0205183482425774 -0.000338154824857464 0.942001165268504 0.942001165268504 0.806670758889582 0.83384515158946 0.997241865580028 turquoise

BC039122 0.0194889969610386 -0.0194889969610386 -0.101246470809553 -0.0801313256959985 0.00986366938126569 0.842069677299434 0.842069677299434 0.299423816184737 0.411948400079977 0.919681736050664 yellow

BC039319 -0.151233379088762 0.151233379088762 -0.166132634254936 -0.174275988647518 -0.056393528317708 0.119957038748844 0.119957038748844 0.0872268691663311 0.0726033184701645 0.563975257416839 turquoise

BC039487 0.10387261247615 -0.10387261247615 0.00619557302655008 -0.0645911430638029 -0.118475598436298 0.28699825051623 0.28699825051623 0.94949945458555 0.508625721050102 0.224208753798236 grey

BC039673 -0.299173772719389 0.299173772719389 -0.0430895500890483 0.169528012302592 0.349583166270123 0.00174596318486826 0.00174596318486826 0.659436481118359 0.0808676966874785 0.000223547209002694 turquoise

BC039686 0.0273912210803739 -0.0273912210803739 -0.139573689930793 -0.203745350712113 -0.144617614325955 0.779430240428396 0.779430240428396 0.151620116764354 0.0352967961242854 0.137228786029215 grey

BC040311 -0.233681703228028 0.233681703228028 -0.20024558258448 -0.135824281736869 0.0579422388040852 0.0154153549311224 0.0154153549311224 0.0386379009561477 0.163031325114804 0.553303108863209 grey

BC040734 -0.165750922368193 0.165750922368193 0.0468934396754821 0.0989990097201746 0.100397129694665 0.0879659779237141 0.0879659779237141 0.631486156548744 0.310334467393665 0.303517050509907 brown

BC040833 0.0431129052379543 -0.0431129052379543 -0.12001852416457 -0.0561366358454419 0.0775902333215757 0.659263382058226 0.659263382058226 0.21818979944846 0.565754904025246 0.426979358668894 turquoise

BC041025 0.158884396643134 -0.158884396643134 0.456156409153046 0.380822000481801 -0.0108599730271792 0.102130770180755 0.102130770180755 7.90162617880142e-07 5.19761002661779e-05 0.911600290393357 blue

BC041363 -0.141480970134254 0.141480970134254 -0.0465350820890318 -0.00745355935060005 0.0543600245295651 0.146050601550537 0.146050601550537 0.634098052263301 0.939263415057672 0.578134732922577 grey

BC041998 -0.229868262927629 0.229868262927629 -0.162473231075553 -0.0469776823578314 0.154252273352436 0.0172265874515126 0.0172265874515126 0.094519310703661 0.630872808423598 0.112656097867547 grey

BC042029 -0.163547642875383 0.163547642875383 -0.184118330781603 -0.129669177141344 0.0451617076184551 0.0923299689448882 0.0923299689448882 0.0576396185238465 0.183129228858113 0.644149347730213 turquoise

BC042366 -0.176356845790919 0.176356845790919 -0.360341139402363 -0.319644630663207 -0.023332100113788 0.0692029020482569 0.0692029020482569 0.00013755550522588 0.000790517715084748 0.811457007358896 blue

BC042374 -0.0832690163870536 0.0832690163870536 -0.191101618068212 -0.142692188959453 0.0331276504736722 0.393821978405019 0.393821978405019 0.048636520305676 0.142594674642621 0.734803203779547 turquoise

BC042590 -0.349654206953942 0.349654206953942 -0.00781383776376331 0.139895325416271 0.248531469918634 0.000222844082371852 0.000222844082371852 0.936333633078154 0.150669885858392 0.00984379785787244 brown

BC043227 -0.115502469669769 0.115502469669769 0.00587267676228416 0.101567047915105 0.163815467354205 0.236134730402737 0.236134730402737 0.952128203687246 0.297888337557454 0.0917905188994487 turquoise

BC043291 -0.337554826480272 0.337554826480272 -0.198886133805745 -0.0766791706899304 0.156302768849149 0.000377061722981149 0.000377061722981149 0.0400052663915565 0.432444199115721 0.107897370722996 turquoise

BC043356 -0.744508416933128 0.744508416933128 -0.507516197656351 -0.222822793405969 0.352796463531182 3.89352975565623e-20 3.89352975565623e-20 2.41646734661632e-08 0.0210599435283076 0.000193714435640164 blue

BC043540 -0.102902863886907 0.102902863886907 -0.172534374028427 -0.0471189354403789 0.168498962390879 0.291546042726466 0.291546042726466 0.0755518800873329 0.629844945398228 0.0827545416674428 turquoise

BC044596 -0.381073152003094 0.381073152003094 -0.205325747649169 -0.0257484650663384 0.25195985089101 5.13385320203025e-05 5.13385320203025e-05 0.0338696648773878 0.792350581139518 0.00884307738389745 turquoise

BC044614 0.0635413465556085 -0.0635413465556085 0.0270544778628073 0.00725922091776724 -0.0266410650099177 0.515553820806456 0.515553820806456 0.782073975982875 0.940844113071308 0.785323030230132 yellow

BC045559 -0.274066745818434 0.274066745818434 -0.151668793214374 0.0127168993991615 0.23994566430769 0.00428232405148556 0.00428232405148556 0.118882044725412 0.896562616631009 0.0127978824585454 grey

BC045560 0.0766606150785415 -0.0766606150785415 0.0347920700570461 -0.0763541459316699 -0.179601105508056 0.432555914658842 0.432555914658842 0.722010870014377 0.434403417316022 0.0641595775741322 grey

BC045779 -0.109266854501223 0.109266854501223 -0.0182280235959533 0.0447127662775322 0.102083863939411 0.262565548976378 0.262565548976378 0.852167969962964 0.647449082960183 0.295423863732847 turquoise

BC045784 -0.161704895590771 0.161704895590771 -0.182727789871566 -0.183710589741215 -0.0485017596663627 0.0961099550659725 0.0961099550659725 0.0595861279058735 0.0582049072544709 0.619820019592189 turquoise

BC045788 0.0272472715283777 -0.0272472715283777 -0.15349138905356 -0.0756149068980387 0.0927475142861341 0.780560067431394 0.780560067431394 0.114462846551976 0.43887824746652 0.34202624673363 grey

BC045789 -0.443720226591871 0.443720226591871 -0.207108418733868 0.0014113173750801 0.300593178796404 1.69325152651507e-06 1.69325152651507e-06 0.0323185446456312 0.988489066873355 0.0016555994681834 blue

BC045791 -0.159745789101402 0.159745789101402 -0.313919028765476 -0.212109550866258 0.0922219632248937 0.10026140906798 0.10026140906798 0.000992256868171198 0.0282836985650689 0.344780382314925 turquoise

BC045805 -0.0639992815575338 0.0639992815575338 -0.266024457559515 -0.239313301784685 -0.0228788962327334 0.512525768157465 0.512525768157465 0.00561341402867579 0.0130432431166763 0.815053260630005 turquoise

BC047364 -0.270864746925744 0.270864746925744 -0.424794982516006 -0.346585590442617 0.0237745541247582 0.00477413139031063 0.00477413139031063 5.10502839831622e-06 0.00025515564630227 0.807949834235087 blue

BC047484 0.0659459386880947 -0.0659459386880947 0.0792510850659413 -0.00687026904026594 -0.125760383508967 0.499756417264485 0.499756417264485 0.417119799659652 0.944008429796345 0.196798298452925 yellow

BC047615 0.0942631354684294 -0.0942631354684294 0.0192325081619353 -0.0635270310109412 -0.13544159506685 0.334161620224506 0.334161620224506 0.844121707278288 0.515648628317821 0.164230984701561 greenyellow

BC047626 -0.289812227283639 0.289812227283639 -0.230665128987694 -0.134159979170263 0.10456386449219 0.0024626609503851 0.0024626609503851 0.0168336002428963 0.168296373679831 0.283785481551351 turquoise

BC047644 -0.429218079958307 0.429218079958307 -0.287778558682856 -0.162298837536037 0.139069858274012 3.96784246421451e-06 3.96784246421451e-06 0.00264974827457161 0.0948785114961428 0.153117655685392 blue

BC047651 -0.33362355083212 0.33362355083212 0.132367632217645 0.162742869987624 0.0854474273058836 0.000445305235359041 0.000445305235359041 0.174106054828319 0.0939660471964913 0.381521997309202 tan

BC048132 -0.0341221986669408 0.0341221986669408 -0.104287307549636 -0.0533809423290667 0.0596140864566797 0.727150364343523 0.727150364343523 0.285067957773703 0.585010433516041 0.541893048169061 turquoise

BC048141 -0.0796565140940106 0.0796565140940106 -0.24113765869017 -0.203500177281402 0.00203232852774434 0.414733239689239 0.414733239689239 0.0123463095031251 0.0355226616011164 0.983424607812948 turquoise

BC048420 -0.114901334598909 0.114901334598909 -0.0584678423040815 -0.0280208790125415 0.0366561546354643 0.238598803667363 0.238598803667363 0.549703483827935 0.774493625375918 0.707774526636848 grey

BC048997 -0.137275827232839 0.137275827232839 -0.211160728800259 -0.182350115395739 -0.00525586238914022 0.158540146776455 0.158540146776455 0.0290144953552828 0.0601239875046704 0.957151189814533 turquoise

BC053951 -0.268862676147126 0.268862676147126 -0.249369331561764 -0.137366437936016 0.126056001054903 0.00510666065863893 0.00510666065863893 0.00959046463337404 0.158262883706746 0.195739477568485 turquoise

BC062753 0.402875656482941 -0.402875656482941 0.325915652461236 0.0835893608226964 -0.32748216332892 1.69097881642567e-05 1.69097881642567e-05 0.000613134999445025 0.391998522022025 0.000574936365141222 turquoise

BC062763 -0.612119725408396 0.612119725408396 -0.361289184305757 -0.142559941626117 0.278391883203745 2.46563704064831e-12 2.46563704064831e-12 0.00013168364917168 0.142968974285002 0.00369000251449526 blue

BC069004 -0.235449209208402 0.235449209208402 0.0381854002432137 0.195911580720667 0.277311645870777 0.0146334861544634 0.0146334861544634 0.696169862651381 0.0431386538333816 0.00383061997483078 grey

BC069739 -0.0656127430727345 0.0656127430727345 0.064252506529082 0.12015761214977 0.111290933053049 0.501930185443359 0.501930185443359 0.510855274066632 0.217652895890911 0.253774154630252 grey

BC069756 -0.0274810042321922 0.0274810042321922 -0.00836556155140729 0.0245317000389466 0.0536539276049579 0.778725785402639 0.778725785402639 0.931848678228937 0.801957063926807 0.583089604912972 grey

BC069776 -0.00505100437555057 0.00505100437555057 0.00889965368756005 -0.0203755883547262 -0.0473736215338928 0.958819817963587 0.958819817963587 0.927509080068519 0.834984755905329 0.627993444073484 grey

BC070118 -0.209717366396087 0.209717366396087 -0.337528455637712 -0.241537257800189 0.0763020692337727 0.0301568824361702 0.0301568824361702 0.000377485481420185 0.0121980714377127 0.434717799724154 turquoise

BC070490 -0.15055536475545 0.15055536475545 -0.149737134047764 -0.0816437555439959 0.0771163203481685 0.12164590719598 0.12164590719598 0.123708375129101 0.403150957343015 0.429817072030872 turquoise

BC079832 -0.361385291651798 0.361385291651798 -0.494873929153851 -0.27011615992644 0.254378029610834 0.000131101573434998 0.000131101573434998 6.01604376496804e-08 0.00489613528401387 0.00819219028087896 blue

BC113958 0.158403335289682 -0.158403335289682 0.176064045092626 0.140352441037819 -0.0154447094080037 0.103186586150175 0.103186586150175 0.0696734357961546 0.14932710374261 0.874540134868596 yellow

BC130595 -0.0855934758521984 0.0855934758521984 -0.105758631791456 0.0192067607355634 0.184850874566722 0.380705760262858 0.380705760262858 0.278289293356665 0.844327756535987 0.0566353298956134 turquoise

BCAM 0.0743621032822313 -0.0743621032822313 -0.173552945466246 -0.207384948498824 -0.101866756182567 0.446521115370065 0.446521115370065 0.0738159611811689 0.0320833926528259 0.296457513275487 green

BCAN -0.237067969290378 0.237067969290378 -0.357097992183896 -0.236091395023767 0.113715644486173 0.01394787884915 0.01394787884915 0.000159530969521918 0.0143580689014515 0.243511282882395 yellow

BCAP29 -0.357186283499285 0.357186283499285 -0.554533555105621 -0.444272607351386 0.0448849985353098 0.000158891905909026 0.000158891905909026 5.74731462383137e-10 1.63792837056241e-06 0.646182357530063 brown

BCAP31 -0.275624985882257 0.275624985882257 -0.416543024899461 -0.367175001349721 -0.0230291221190679 0.00405978669003861 0.00405978669003861 8.09287781539089e-06 0.000100133186836676 0.813860761996418 brown

BCAR1 -0.0870339478229279 0.0870339478229279 -0.174950501828664 -0.111201866108459 0.0632846893099086 0.372711991962315 0.372711991962315 0.0714866067487006 0.254156707848106 0.517254936149943 green

BCAR3 0.126378453856393 -0.126378453856393 -0.100572395578677 -0.133086652066222 -0.0809260902259759 0.194589239725113 0.194589239725113 0.302669403905356 0.171757952268959 0.407311532508682 green

BCAR4 -0.452867206103725 0.452867206103725 -0.260906543106325 -0.0735464967958898 0.250914547606456 9.6947828107e-07 9.6947828107e-07 0.00664118227371388 0.45153667241628 0.00913825042517769 blue

BCAS1 0.153849747470045 -0.153849747470045 0.00932416768798194 -0.0191799389151305 -0.0459568325069412 0.113609130574887 0.113609130574887 0.924061333729449 0.844542415026353 0.638322111917947 green

BCAS2 0.113126778908951 -0.113126778908951 0.121613322358904 0.00599964313846478 -0.164925440298426 0.245976888825087 0.245976888825087 0.212089723011534 0.951094487698125 0.0895813801841055 yellow

BCAS3 0.379879624311869 -0.379879624311869 0.104492555012712 -0.0071447719843708 -0.162569216739018 5.44349225558794e-05 5.44349225558794e-05 0.28411579564417 0.941775118647791 0.0943220665445627 grey

BCAS4 0.667799294046079 -0.667799294046079 0.514676993260156 0.158842071443488 -0.471626446917759 3.95217686100679e-15 3.95217686100679e-15 1.41766871442858e-08 0.102223322640785 2.93286732818583e-07 brown

BCAT1 -0.650765993776992 0.650765993776992 -0.324710454785546 0.0302397229321813 0.518816009936226 3.26310220534585e-14 3.26310220534585e-14 0.000644092256685405 0.757170285109114 1.03573481221783e-08 purple

BCAT2 0.393202408112474 -0.393202408112474 0.257906585543581 0.107665874566256 -0.188724209399693 2.79457425453407e-05 2.79457425453407e-05 0.00731825936258665 0.269664440532744 0.0515610065172237 brown

BCCIP 0.113744106823587 -0.113744106823587 0.129289212143935 0.00274400785332478 -0.181499326802698 0.24339254502804 0.24339254502804 0.184426707148429 0.977621554827072 0.0613501687895456 red

BCDIN3D 0.0974255266530167 -0.0974255266530167 0.0206772192825423 0.0513349655576392 0.0572993635935578 0.318125217848642 0.318125217848642 0.832577341779262 0.599498083206969 0.557721296268532 yellow

BCDIN3D-AS1 -0.124353510918991 0.124353510918991 -0.270324358228298 -0.209527899159489 0.0338321844746799 0.201893997965198 0.201893997965198 0.0048619273660025 0.0303096325887953 0.729379231983987 green

BCHE 0.00551858320982129 -0.00551858320982129 -0.147455512481371 -0.190808709450948 -0.111326936624454 0.955011524079338 0.955011524079338 0.129601675511308 0.0489892446361179 0.25361962715827 turquoise

BCKDHA 0.0299245776743815 -0.0299245776743815 -0.0389298022318461 0.0202098543129078 0.0903306163410951 0.759623650267733 0.759623650267733 0.690546047051315 0.836308183552416 0.354806889855107 grey

BCKDHB 0.521596380704646 -0.521596380704646 0.375291895366162 0.0987955404122376 -0.372783480422571 8.36843420477668e-09 8.36843420477668e-09 6.80345242392945e-05 0.31133485124446 7.67496209677078e-05 blue

BCKDK -0.478291140914905 0.478291140914905 -0.493546918075519 -0.180836549963755 0.403897371644346 1.88468366991645e-07 1.88468366991645e-07 6.60647174257015e-08 0.0623194599794219 1.60212059688989e-05 blue

BCL10 -0.34952830706167 0.34952830706167 -0.0309418028920841 0.110498785795555 0.231971158267135 0.000224091582972912 0.000224091582972912 0.751713378817577 0.257190407068874 0.0162062786322143 brown

BCL11A 0.712130943024729 -0.712130943024729 0.620622528392006 0.259043723000389 -0.454214043587672 7.98580802666432e-18 7.98580802666432e-18 1.00179786613025e-12 0.00705474582633982 8.91826161318903e-07 blue

BCL11B 0.814270707377754 -0.814270707377754 0.827614084351013 0.416391217370639 -0.485363053271034 1.49824958954421e-26 1.49824958954421e-26 4.32429501470493e-28 8.16084421492415e-06 1.16666548635793e-07 blue

BCL2 0.78098786858207 -0.78098786858207 0.652672608520828 0.251353457362036 -0.513404161391303 3.38366892002996e-23 3.38366892002996e-23 2.5936635542979e-14 0.00901327143656474 1.56003228518017e-08 blue

BCL2A1 -0.708733105214149 0.708733105214149 -0.578296439249078 -0.24161704957623 0.422829883466524 1.33836852838399e-17 1.33836852838399e-17 6.88974875087879e-11 0.0121686582195506 5.70334152749764e-06 brown

BCL2L1 0.00842560591417113 -0.00842560591417113 -0.23354500500755 -0.266764467164919 -0.116204333519646 0.931360704577663 0.931360704577663 0.015477306705938 0.0054771525744436 0.233280282299172 pink

BCL2L10 -0.131657568478244 0.131657568478244 -0.137744291761805 -0.0399717458807461 0.130530001510862 0.17644801707499 0.17644801707499 0.157110575053341 0.682702613635966 0.180214431873309 green

BCL2L11 0.0116305467552436 -0.0116305467552436 0.382755429951736 0.38255654022787 0.0977664055748879 0.905355968456975 0.905355968456975 4.72526670938822e-05 4.77193753842309e-05 0.316426807444604 black

BCL2L12 0.0888202054629485 -0.0888202054629485 -0.239538877858396 -0.367193784545701 -0.277915577517492 0.362942882476063 0.362942882476063 0.0129552517205912 0.000100044850316825 0.00375142423980376 magenta

BCL2L13 0.117853719116288 -0.117853719116288 -0.269489039735797 -0.342872102105388 -0.193539883442981 0.226667479586472 0.226667479586472 0.00500047422105921 0.000300032563821683 0.045781138357834 pink

BCL2L14 -0.189668266260732 0.189668266260732 -0.38420520366777 -0.317761408255712 0.0142220824565934 0.0503827995331479 0.0503827995331479 4.39773544929841e-05 0.000852301909891949 0.884399981774547 turquoise

BCL2L15 -0.336704681143199 0.336704681143199 -0.367101693312028 -0.314050084505808 -0.00410909165191818 0.00039094586574952 0.00039094586574952 0.000100478645935168 0.000987157093343281 0.966494149006328 magenta

BCL2L2 -0.174683587010258 0.174683587010258 -0.278989368806746 -0.150110002054297 0.14708883563439 0.0719268358907818 0.0719268358907818 0.00361422907176515 0.122765196117695 0.130568493241265 black

BCL6 -0.833053953002894 0.833053953002894 -0.391722441711586 -0.0601380031474377 0.462008827912711 9.32854741782936e-29 9.32854741782936e-29 3.01370474308075e-05 0.538341377702093 5.46217975690425e-07 brown

BCL6B 0.0428876965074816 -0.0428876965074816 -0.206817327735233 -0.216461116160149 -0.0693663070062131 0.660933287720393 0.660933287720393 0.0325676498146978 0.0251290725248849 0.477729738505359 green

BCL7A 0.641305795656258 -0.641305795656258 0.192543707621989 -0.136046921506773 -0.507982266662785 9.95618337312616e-14 9.95618337312616e-14 0.046930448729492 0.162336389797519 2.33489955650047e-08 brown

BCL7B 0.186689850814347 -0.186689850814347 -0.0717485777003933 -0.105193206639432 -0.0751163257705768 0.054177284042425 0.054177284042425 0.462703101493494 0.28088140698611 0.441910985378939 greenyellow

BCL7C -0.190441485737486 0.190441485737486 -0.151508192993417 -0.0953742963582032 0.0563775283149857 0.0494344471517241 0.0494344471517241 0.119277680942289 0.328469382204547 0.564086021280244 yellow

BCL9 0.466815232889392 -0.466815232889392 0.191493587744128 -0.0384129042326605 -0.340870119748107 4.0126211897925e-07 4.0126211897925e-07 0.0481677960599688 0.694449348340876 0.000327146094503204 brown

BCL9L 0.834045022820475 -0.834045022820475 0.757807933279312 0.425900057745502 -0.368726323678765 7.01257989817475e-29 7.01257989817475e-29 3.44498773624599e-21 4.79511289097551e-06 9.30767990329449e-05 blue

BCLAF1 0.263340261369707 -0.263340261369707 0.469741019593425 0.309457028522584 -0.151463823023606 0.00613331105207663 0.00613331105207663 3.31810155574215e-07 0.00118090540634708 0.11938716477881 tan

BCMO1 -0.316128882385844 0.316128882385844 -0.256352969633663 -0.102703696226534 0.194903774580195 0.000909399243371438 0.000909399243371438 0.00769234132517435 0.292485948734954 0.0442455776931576 blue

BCO2 -0.0551574343204452 0.0551574343204452 0.0408207694670884 0.133331180125036 0.167372588165248 0.572562678908819 0.572562678908819 0.676336254810807 0.170964744376414 0.0848599792482624 yellow

BCOR 0.724532577378247 -0.724532577378247 0.732923527925328 0.467931061047707 -0.261607094993589 1.136553921643e-18 1.136553921643e-18 2.85832156289958e-19 3.73284834668524e-07 0.00649130236511241 blue

BCORL1 -0.201209109165525 0.201209109165525 -0.323931864995943 -0.211361188946902 0.107907981792666 0.0376926501163408 0.0376926501163408 0.000664846429034321 0.028858777473326 0.26858266640088 yellow

BCORP1 -0.189061420064494 0.189061420064494 -0.0336065946918278 -0.00669443660484811 0.0370328997977926 0.0511375765694092 0.0511375765694092 0.731114534904176 0.945439202849166 0.704909260142514 turquoise

BCR 0.697808907082581 -0.697808907082581 0.377013159451731 0.0601523068286161 -0.440805810297786 6.70418923559123e-17 6.70418923559123e-17 6.25980677133768e-05 0.538244573644007 2.01567165634071e-06 brown

BCRP3 -0.487797275474069 0.487797275474069 -0.588344756081267 -0.340238890490041 0.270022069954441 9.86637511626392e-08 9.86637511626392e-08 2.66646660933136e-11 0.000336152723726217 0.00491166496581218 blue

BCS1L 0.228548517008239 -0.228548517008239 0.0214335976559172 -0.165931566023042 -0.312302386822217 0.017894893291686 0.017894893291686 0.826547290851139 0.0876155795202565 0.00105717335520254 red

BD495725 -0.0536604008103778 0.0536604008103778 -0.0964227106603178 -0.0969851157408302 -0.0256679740407141 0.583044092174062 0.583044092174062 0.323155757444844 0.320328240096477 0.792985124311762 grey

BDH1 0.197544684102136 -0.197544684102136 -0.067623920787971 -0.229648041741586 -0.29214691040144 0.0413939561000321 0.0413939561000321 0.488884558025512 0.0173365820845581 0.00226263130764211 grey

BDH2 0.336669305493682 -0.336669305493682 0.376735776293202 0.17747029941334 -0.241419654900654 0.000391533678866026 0.000391533678866026 6.34458472072392e-05 0.0674370113414629 0.0122415359603784 blue

BDKRB1 -0.162694004816828 0.162694004816828 -0.214933970757794 -0.182043279552401 0.000697377458998151 0.0940661217375335 0.0940661217375335 0.0262001438237753 0.0605638786545961 0.994311929479361 turquoise

BDKRB2 0.19702610708669 -0.19702610708669 -0.160658662363809 -0.244435977109914 -0.183275669288278 0.0419414714750267 0.0419414714750267 0.0983097806883336 0.0111685125450324 0.0588128729100191 turquoise

BDNF -0.282376528505785 0.282376528505785 -0.358804441673364 -0.278166549727978 0.0448076175446857 0.00321043891594158 0.00321043891594158 0.00014759195776608 0.00371894704909573 0.646751350675141 turquoise

BDNF-AS -0.0547247721289708 0.0547247721289708 -0.241680601435755 -0.21719760339702 -0.0204185618420498 0.575582870319428 0.575582870319428 0.0121452757700867 0.0246260317852213 0.834641676920483 turquoise

BDP1 0.243224537828465 -0.243224537828465 0.237239813855673 0.0423808468292915 -0.269699747325943 0.0115891512759795 0.0115891512759795 0.0138767639153263 0.664697619768107 0.00496519818057474 yellow

BEAN1 0.0652224242949017 -0.0652224242949017 -0.0112069698405654 -0.0657710771539563 -0.0954203486562223 0.504482883616944 0.504482883616944 0.908787722658604 0.500896599361952 0.328234811642007 yellow

BECN1 -0.0482663459829698 0.0482663459829698 -0.100319929596957 -0.0444221065194036 0.069097156004101 0.621521830754374 0.621521830754374 0.303890909746424 0.649589095686541 0.479443842338136 yellow

BEGAIN -0.0986773494372601 0.0986773494372601 -0.160632752668777 -0.0529984813869574 0.141390258930401 0.311916914265309 0.311916914265309 0.0983647575240904 0.58770643900517 0.146311945525578 turquoise

BEND2 0.0368971915210002 -0.0368971915210002 -0.205208455485708 -0.304102747291486 -0.220334577670979 0.70594089176031 0.70594089176031 0.0339738849102284 0.00145009096413743 0.0225785749963923 salmon

BEND3 0.094317173681683 -0.094317173681683 -0.0427426893128275 -0.111862193793325 -0.128191115214517 0.33388335234911 0.33388335234911 0.662009386788552 0.251329915283244 0.188214089314707 grey

BEND4 0.199959624856817 -0.199959624856817 0.0178330210272588 -0.223328726770177 -0.404471355683683 0.0389222259818919 0.0389222259818919 0.855336281756179 0.0207621290936763 1.55414507057796e-05 grey

BEND5 0.295929180003185 -0.295929180003185 0.151261656458359 -0.00687317769832313 -0.229447749682792 0.00196953352481039 0.00196953352481039 0.119886997713022 0.943984763124321 0.0174371492615254 grey

BEND6 -0.317455814206649 0.317455814206649 -0.445800999165687 -0.357974178818757 0.0347029264513936 0.000862734103559223 0.000862734103559223 1.49363891797094e-06 0.000153293244094102 0.722694104699032 blue

BEND7 -0.572724642266637 0.572724642266637 -0.288132629906607 -0.0764953007587443 0.285113750193349 1.15036726166927e-10 1.15036726166927e-10 0.00261628227709554 0.433551929388491 0.00291424980361294 blue

BEST1 -0.418855998253486 0.418855998253486 -0.0767655962580313 0.148327455653264 0.362111761039006 7.12106804947325e-06 7.12106804947325e-06 0.431924084066254 0.127324632224516 0.000126778384572633 red

BEST2 -0.0289130693338102 0.0289130693338102 -0.218828709288841 -0.17203473903001 0.0232809367558662 0.767514119940851 0.767514119940851 0.023542473419393 0.0764153173764361 0.811862802893886 turquoise

BEST3 -0.130435523874396 0.130435523874396 -0.365397257230283 -0.256297259226187 0.0913932856901346 0.180532668355999 0.180532668355999 0.000108830108028196 0.00770606349039591 0.349151281164756 green

BEST4 -0.0173539972955563 0.0173539972955563 0.0348559690812652 0.0826807908694656 0.0900511115820444 0.859181608083953 0.859181608083953 0.721521255000858 0.397183411492231 0.356303851632642 grey

BET1 0.247731029933195 -0.247731029933195 0.185474587232154 0.0552347243502398 -0.173365120419672 0.0100912861401124 0.0100912861401124 0.0557915988432536 0.572023938902162 0.074133625646597 yellow

BET1L 0.460793606794215 -0.460793606794215 0.202801027222027 0.00823351849085486 -0.278032331402242 5.90075275241239e-07 5.90075275241239e-07 0.0361734363765058 0.932921865602723 0.0037362841697528 blue

BEX1 -0.463353910207852 0.463353910207852 -0.492852287987799 -0.3650897754735 0.0903794221979594 5.01289965029809e-07 5.01289965029809e-07 6.93719482134052e-08 0.000110403773164303 0.354545899086763 magenta

BEX2 0.600100952561567 -0.600100952561567 0.601277345928289 0.27537454508225 -0.398661157551634 8.41936377555591e-12 8.41936377555591e-12 7.48263882074534e-12 0.0040948401379112 2.10877327089684e-05 blue

BEX4 0.500649991516764 -0.500649991516764 0.458149950820377 0.257645052231855 -0.222654684001676 3.983947692174e-08 3.983947692174e-08 6.97316108893282e-07 0.00738008278945606 0.0211597086452136 turquoise

BEX5 0.509584132297528 -0.509584132297528 0.440945597151904 0.110644648249757 -0.4472154899107 2.07416380208941e-08 2.07416380208941e-08 1.99896403797953e-06 0.256559002436373 1.37090492509178e-06 blue

BFAR 0.166093686202349 -0.166093686202349 0.146459517471103 0.0319742265094034 -0.156643234063171 0.0873020572470493 0.0873020572470493 0.132240659765766 0.743711127864464 0.107122641746656 turquoise

BFSP1 0.280001556034306 -0.280001556034306 0.216147781401111 0.12416999904967 -0.100605786472403 0.00348904071997764 0.00348904071997764 0.0253457323432735 0.202565604945903 0.30250809011389 brown

BFSP2 -0.377766218135813 0.377766218135813 -0.290667393016227 -0.158386128217482 0.149865719059077 6.0349450774659e-05 6.0349450774659e-05 0.00238761277443306 0.103224509453474 0.123382488413783 blue

BFSP2-AS1 -0.180235089843305 0.180235089843305 -0.206069345971706 -0.038513224068869 0.231379783868024 0.0632098496344753 0.0632098496344753 0.0332151895219338 0.693691164853578 0.0164877780479847 turquoise

BGLT3 0.399931465883081 -0.399931465883081 -0.0105535426333157 -0.161627973071082 -0.258947083888516 1.97361918361528e-05 1.97361918361528e-05 0.914084957861004 0.0962703595303361 0.007076809287037 green

BGN 0.0336983600817945 -0.0336983600817945 -0.206347799322185 -0.124277041471097 0.0863140067870456 0.730408484351551 0.730408484351551 0.0329728704238638 0.20217366207456 0.376694347097228 green

BHLHB9 0.169876043925687 -0.169876043925687 0.0594986923823523 -0.0989143195407466 -0.253439322124079 0.0802374004800598 0.0802374004800598 0.542676858858945 0.310750603507738 0.00843966061268615 brown

BHLHE22 0.00830986574412607 -0.00830986574412607 -0.198510068456367 -0.202368401538383 -0.0574241970002604 0.93230133424455 0.93230133424455 0.0403905879635625 0.0365811228864452 0.556862051082085 turquoise

BHLHE23 -0.449855731624486 0.449855731624486 -0.413661324599357 -0.253286149242688 0.165992233727067 1.16694530293152e-06 1.16694530293152e-06 9.47872664764185e-06 0.00848066067403548 0.0874981503165453 blue

BHLHE40 0.59014318697032 -0.59014318697032 0.635077444770076 0.386520412981503 -0.258809301186854 2.24207295236941e-11 2.24207295236941e-11 2.03221758227678e-13 3.91835820133443e-05 0.0071083717383003 blue

BHLHE41 -0.0632376984806196 0.0632376984806196 -0.110596475671718 -0.254466109163398 -0.272368729737107 0.517566699669288 0.517566699669288 0.25676741288493 0.00816930177222551 0.00453717983114986 red

BHMT -0.313014301278095 0.313014301278095 -0.199125273876955 -0.0696889859796456 0.168503334316555 0.0010281235056452 0.0010281235056452 0.0397618425919404 0.475679100778465 0.0827464516487798 turquoise

BHMT2 -0.175470331493111 0.175470331493111 -0.300495834141293 -0.239237265071365 0.026882729971082 0.0706354862016706 0.0706354862016706 0.00166165854078771 0.0130730202452231 0.783423308852326 yellow

BICC1 -0.322272029452376 0.322272029452376 -0.292990762242573 -0.121887971723115 0.215116644483197 0.000711151765565001 0.000711151765565001 0.00219402257704474 0.211051575718494 0.0260700108181791 blue

BICD1 0.770425758416587 -0.770425758416587 0.57408014078662 0.196137291703863 -0.493898834438397 2.97433295860065e-22 2.97433295860065e-22 1.01638556253217e-10 0.0428939317690089 6.44471808598317e-08 blue

BICD2 -0.280867651122257 0.280867651122257 0.135726030382103 0.179036680754365 0.108248418384329 0.00338502404489507 0.00338502404489507 0.16333870362466 0.0650148437776644 0.267066516168204 purple

BID -0.237174229017685 0.237174229017685 0.236110365103183 0.355090240584715 0.262322817503105 0.0139038677612449 0.0139038677612449 0.0143500021068547 0.000174722721191362 0.00634128127099814 purple

BIK -0.67090980732585 0.67090980732585 -0.593488181686299 -0.408770173459787 0.161189587739013 2.64781470809499e-15 2.64781470809499e-15 1.61950313224665e-11 1.23545671877446e-05 0.0971885467646457 brown

BIN1 0.824820555049305 -0.824820555049305 0.769419870289153 0.399336315742325 -0.430501057438813 9.31083470563262e-28 9.31083470563262e-28 3.63633966620037e-22 2.03589484751577e-05 3.68569493658128e-06 blue

BIN2 -0.263798903240405 0.263798903240405 -0.127809342443483 -0.0658027151034421 0.0724127014288437 0.00604155863311794 0.00604155863311794 0.189543998864687 0.500690203938029 0.458560813394882 brown

BIN3 -0.320741471179548 0.320741471179548 -0.227290213528403 -0.0567674871206882 0.230972441170536 0.000756448888656566 0.000756448888656566 0.0185528152445416 0.561389383125478 0.0166841299865395 brown

BIN3-IT1 0.368786798070612 -0.368786798070612 0.324195138097803 0.235035776986681 -0.0681318153946203 9.28113314382326e-05 9.28113314382326e-05 0.000657760995851269 0.0148132155975422 0.485618785552248 yellow

BIRC2 -0.46365691442805 0.46365691442805 -0.339840028917607 -0.0849430836578483 0.345234659187816 4.91665455944148e-07 4.91665455944148e-07 0.000341961145449162 0.384348805229451 0.00027070948851856 brown

BIRC3 0.52348203278464 -0.52348203278464 0.636484418298744 0.386948548977232 -0.260108902744977 7.23376772282056e-09 7.23376772282056e-09 1.73214421260311e-13 3.83524977991475e-05 0.0068155605214408 blue

BIRC5 -0.529605106223765 0.529605106223765 -0.702842778529122 -0.521505852908684 0.127426235569681 4.47917970779653e-09 4.47917970779653e-09 3.21982961777258e-17 8.42699017277534e-09 0.19088540376932 magenta

BIRC6 0.542087257080502 -0.542087257080502 0.58618815584474 0.372166342027179 -0.212763786450046 1.63601686766896e-09 1.63601686766896e-09 3.2779998336842e-11 7.90477505501304e-05 0.0277889525230183 blue

BIRC7 0.158615207758644 -0.158615207758644 0.0163570386511277 -0.0259403317402764 -0.0675494321000366 0.102720526950651 0.102720526950651 0.867195079928195 0.790838566189981 0.48936450054209 green

BIVM 0.610445650668433 -0.610445650668433 0.524443188062123 0.241190128275086 -0.34601489952837 2.93473031592131e-12 2.93473031592131e-12 6.71368251729409e-09 0.0123267557540513 0.00026162265098648 blue

BLACAT1 0.181624152491362 -0.181624152491362 -0.0152930759803793 -0.0874999130297337 -0.126392041560304 0.0611689991550058 0.0611689991550058 0.875761968362178 0.370148261972116 0.194540877888354 green

BLCAP 0.837543722226726 -0.837543722226726 0.663732405716423 0.349366543549277 -0.363085182485925 2.52195634289861e-29 2.52195634289861e-29 6.6244763333499e-15 0.00022570392803416 0.000121193013236626 blue

BLID -0.278499589423245 0.278499589423245 -0.239215987543283 -0.166536807174559 0.0619602357092606 0.00367623887101737 0.00367623887101737 0.0130813634510194 0.0864496619938669 0.526078671685204 blue

BLK 0.506724852109323 -0.506724852109323 0.465791203674644 0.141912679045177 -0.429954049079228 2.56124088329415e-08 2.56124088329415e-08 4.28682707528843e-07 0.14481164814607 3.80359085007053e-06 blue

BLM -0.347697826048007 0.347697826048007 -0.491717488071018 -0.381039542237691 0.0616926524558608 0.000242974454337123 0.000242974454337123 7.51173243521013e-08 5.14234276970023e-05 0.527870518361667 magenta

BLMH 0.546270411331267 -0.546270411331267 0.463690089924012 0.223014905358034 -0.289368775526567 1.15654036153432e-09 1.15654036153432e-09 4.90622414348002e-07 0.0209464288333645 0.00250240942893846 red

BLNK 0.531068940154583 -0.531068940154583 0.437976793146164 0.0846978339141788 -0.48695016427759 3.98856875861768e-09 3.98856875861768e-09 2.38359532918693e-06 0.385727963082122 1.04604956146154e-07 red

BLOC1S1 -0.745761931257737 0.745761931257737 -0.646444849333754 -0.301326388784293 0.419676609724122 3.1179786476267e-20 3.1179786476267e-20 5.45788700611194e-14 0.00161060182602966 6.80354385473546e-06 brown

BLOC1S2 -0.466941640134012 0.466941640134012 -0.314010006157892 -0.134773117362961 0.223525698766872 3.97995316151868e-07 3.97995316151868e-07 0.000988714118400241 0.166342256516415 0.0206471652772411 brown

BLOC1S3 0.616916492966514 -0.616916492966514 0.359600396777596 0.229285681458944 -0.128862017144683 1.4883793324126e-12 1.4883793324126e-12 0.000142311570035421 0.0175188927731866 0.185893457631729 brown

BLOC1S4 0.524476942202213 -0.524476942202213 0.410110559555755 0.13661282393063 -0.358773079260713 6.69608567591578e-09 6.69608567591578e-09 1.14939751820128e-05 0.16057993328668 0.000147803693131545 turquoise

BLOC1S6 -0.323006833203596 0.323006833203596 -0.26319866264195 -0.10963294953476 0.193007420346859 0.000690299893538204 0.000690299893538204 0.00616188701003598 0.260960285740954 0.0463925108352998 yellow

BLVRA -0.217595070818762 0.217595070818762 0.114603068733354 0.370976958173117 0.464217697092283 0.0243581410973382 0.0243581410973382 0.239828021352504 8.36587386067371e-05 4.7431441819173e-07 yellow

BLVRB -0.0104531917732814 0.0104531917732814 -0.272205626594615 -0.248663724308801 -0.0298386588722439 0.91489882625779 0.91489882625779 0.00456235892168097 0.00980342334316413 0.76029293200379 pink

BLZF1 -0.303979832685835 0.303979832685835 -0.249294996974518 -0.0963793052806296 0.195468523198259 0.00145687764703018 0.00145687764703018 0.00961270626934598 0.323374644558074 0.0436224114908104 yellow

BMF 0.387541832681637 -0.387541832681637 0.547040423589586 0.440039874673706 -0.041275498495349 3.72280641187225e-05 3.72280641187225e-05 1.08444663704517e-09 2.10958956866316e-06 0.672935812508955 brown

BMP1 -0.0505750590377779 0.0505750590377779 -0.225897445704408 -0.160330515290245 0.0533105708793353 0.604919499262609 0.604919499262609 0.0193052683962162 0.099007853107358 0.585506063404605 turquoise

BMP10 -0.129714466364557 0.129714466364557 -0.166412228780691 -0.0330272190632314 0.183585558126345 0.182975024159145 0.182975024159145 0.0866886309822505 0.735577461679097 0.0583791567418969 turquoise

BMP15 0.0469526631246999 -0.0469526631246999 -0.143957982350369 -0.151790994048856 -0.0501835745078113 0.631054940538593 0.631054940538593 0.13904952385693 0.118581684659406 0.607720904291796 turquoise

BMP2 -0.201026949450519 0.201026949450519 -0.227593986771045 -0.124136864458712 0.117142473639086 0.0378698530875415 0.0378698530875415 0.0183921019692728 0.202687039953652 0.229502697297312 turquoise

BMP2K -0.340802828084218 0.340802828084218 -0.353486838223475 -0.148475567962747 0.257124188106989 0.000328095528391087 0.000328095528391087 0.000187806301698193 0.126940910204648 0.00750458606209351 brown

BMP3 -0.100123899489691 0.100123899489691 -0.292393676633406 -0.222572231108571 0.0434832060884063 0.304841584926583 0.304841584926583 0.00224237044390337 0.0212087887446116 0.656521268056191 turquoise

BMP4 -0.170024732554973 0.170024732554973 -0.332997228906902 -0.290879458672759 -0.0139129508482761 0.0799693228272109 0.0799693228272109 0.000457172149844176 0.00236932440029269 0.886895825218643 turquoise

BMP5 -0.239886845694139 0.239886845694139 -0.316474079532374 -0.224416789245455 0.0750260633137262 0.0128205333933433 0.0128205333933433 0.000897042589496295 0.0201338853906117 0.442461289369666 blue

BMP6 -0.0264607181554798 0.0264607181554798 -0.298644089939442 -0.324214541265153 -0.119916092126474 0.786741554016136 0.786741554016136 0.00178081362360027 0.000657241549014917 0.218585804133253 pink

BMP7 -0.089983118670206 0.089983118670206 -0.262994810580679 -0.23140728135123 -0.0138313994535395 0.356668598194001 0.356668598194001 0.00620323378352477 0.0164745957799291 0.887554434088815 yellow

BMP8A 0.0524249934133767 -0.0524249934133767 -0.168281610110036 -0.166025547703751 -0.039305558857235 0.591759592657563 0.591759592657563 0.0831575377907236 0.0874337205975942 0.687713635508342 turquoise

BMP8B 0.00448592836017324 -0.00448592836017324 -0.102955801022347 -0.171312013054264 -0.142329605833324 0.963423426627481 0.963423426627481 0.291296560386575 0.0776783012677097 0.143622667334704 green

BMPER -0.223980012213932 0.223980012213932 -0.361663721698153 -0.206168605503718 0.171042444176403 0.0203840867968707 0.0203840867968707 0.000129428717852806 0.0331286389547879 0.0781536495007403 turquoise

BMPR1A 0.636682713471085 -0.636682713471085 0.453282457788868 0.125089474690254 -0.440478063832004 1.69346543517152e-13 1.69346543517152e-13 9.44877972603592e-07 0.199216633091536 2.05536440916637e-06 brown

BMPR1B -0.169727068896414 0.169727068896414 -0.227686150776408 -0.165659918781446 0.046846608805185 0.080506715705522 0.080506715705522 0.018343580069456 0.0881429209622494 0.631827227627831 turquoise

BMPR2 0.0558907162533662 -0.0558907162533662 0.197193651424906 0.287406832628888 0.203555983683641 0.567461021250453 0.567461021250453 0.0417639208279187 0.00268529746139238 0.0354711435285289 turquoise

BMS1 0.727497210240921 -0.727497210240921 0.605052317048941 0.292519335259516 -0.37501661727288 7.0198313660954e-19 7.0198313660954e-19 5.10809726500669e-12 0.00223211626624444 6.89437524649203e-05 blue

BMS1P20 0.0288911368888066 -0.0288911368888066 0.0487844690302221 -0.0959351553604961 -0.232959653138633 0.767685474935766 0.767685474935766 0.617778957100778 0.325619900857901 0.0157450373322721 red

BMS1P5 0.480838158259864 -0.480838158259864 0.473066946275501 0.151895720396073 -0.423497300734417 1.58766873942602e-07 1.58766873942602e-07 2.66763364537603e-07 0.118324742478767 5.49308229917353e-06 blue

BMS1P6 0.248935500802142 -0.248935500802142 0.368999901014467 0.251972404084847 -0.103915963875226 0.00972091190053897 0.00972091190053897 9.18814690721094e-05 0.00883958422898188 0.286796055477259 blue

BMX -0.799147158011443 0.799147158011443 -0.754587916724614 -0.451410397166269 0.320821174710327 5.99117580819622e-25 5.99117580819622e-25 6.2851407418295e-21 1.0606749586452e-06 0.000754026375594446 brown

BNC1 -0.333372809129581 0.333372809129581 -0.221725972190424 -0.0612094612801859 0.2154267534226 0.000450021604571476 0.000450021604571476 0.0217181996742914 0.531113912328529 0.0258503564949581 turquoise

BNC2 0.591776970054875 -0.591776970054875 0.378343816282464 0.214650567143763 -0.180672310956043 1.91360869975727e-11 1.91360869975727e-11 5.86760805687406e-05 0.0264031297284086 0.0625615751036229 brown

BNIP1 0.3409350030983 -0.3409350030983 0.326573432450359 0.213354177315863 -0.108330987794635 0.000326233040330159 0.000326233040330159 0.00059682043605675 0.0273488193577866 0.266699665348001 blue

BNIP2 -0.577432724306874 0.577432724306874 -0.477589870118052 -0.268560905624214 0.232129965643373 7.46430559438891e-11 7.46430559438891e-11 1.97532655593274e-07 0.00515853271239955 0.0161313977004882 yellow

BNIP3 0.251001901557249 -0.251001901557249 0.0652464566747227 -0.0781194065471032 -0.226444160983954 0.00911325566758098 0.00911325566758098 0.504325516338408 0.42382351019767 0.0190068244581888 yellow

BNIP3L -0.0252473807112014 0.0252473807112014 -0.296070884048174 -0.256254454415861 -0.00835179385968802 0.796303019802585 0.796303019802585 0.0019592525958893 0.00771662153058341 0.931960570245814 pink

BNIPL -0.197428214649332 0.197428214649332 -0.209684588275111 -0.153661943082158 0.0412776967962114 0.0415164026015171 0.0415164026015171 0.0301832617070392 0.114055917781353 0.672919389648589 yellow

BOC -0.088660118172236 0.088660118172236 -0.339595270847949 -0.28689307561752 0.00234848247657182 0.36381190480177 0.36381190480177 0.00034557110275954 0.00273513655635388 0.980846555021801 turquoise

BOD1 0.55429245364374 -0.55429245364374 0.595797580450114 0.330955124629165 -0.296499297172741 5.867375693591e-10 5.867375693591e-10 1.2909183004297e-11 0.000497913193428849 0.0019284649442197 blue

BOD1L1 0.288302854387291 -0.288302854387291 0.475237590912719 0.304523111785073 -0.167746468364504 0.00260032901225586 0.00260032901225586 2.31066432566958e-07 0.00142709718994504 0.0841564325115435 tan

BOD1L2 0.0960282574968392 -0.0960282574968392 0.00695089904000985 0.0802914600071288 0.126176837074782 0.32514842731499 0.32514842731499 0.943352391502358 0.411011659772646 0.195307863153523 grey

BOK 0.249115020107813 -0.249115020107813 0.266338189580594 0.284599450825029 0.0992384546978324 0.00966674492676644 0.00966674492676644 0.00555528125248124 0.00296795080070161 0.309159886357072 brown

BOLA1 0.27369422008851 -0.27369422008851 0.372289798419389 0.261366703870468 -0.0927188939122564 0.00433711437535088 0.00433711437535088 7.85829499824247e-05 0.00654239151833136 0.342175872906476 turquoise

BOLA2 -0.0443646965172805 0.0443646965172805 -0.105000997572112 -0.15979425762745 -0.11984932475476 0.650012120748944 0.650012120748944 0.28176623085296 0.100157028864573 0.218844202190751 grey

BOLA3 0.0159012829917656 -0.0159012829917656 -0.0836631248434312 -0.228465850196942 -0.267048149740557 0.870862942661044 0.870862942661044 0.391579360522869 0.0179374899631873 0.00542570209047292 red

BOLA3-AS1 0.101899772751245 -0.101899772751245 -0.169696947211613 -0.278230694139384 -0.227582416833665 0.296300167894375 0.296300167894375 0.0805612571678946 0.00371068693112738 0.0183982010494064 green

BOLL -0.0971027959443358 0.0971027959443358 -0.0874994573100687 -0.017205641172113 0.0968006617195932 0.319738620402411 0.319738620402411 0.370150764044384 0.860373197865231 0.321253829104546 turquoise

BORA 0.28416137814275 -0.28416137814275 0.0910636786558961 -0.0608542920855136 -0.234332447802298 0.00301439253144754 0.00301439253144754 0.350899407201846 0.533504302418166 0.0151233774850272 yellow

BPESC1 -0.165875322881446 0.165875322881446 -0.323104478326392 -0.336772301932311 -0.105997114227096 0.0877245563825563 0.0877245563825563 0.000687571531697973 0.000389824517994146 0.277200830335292 grey

BPGM -0.395225588576983 0.395225588576983 -0.527050380497166 -0.338831255880656 0.184156538796149 2.51908747452754e-05 2.51908747452754e-05 5.4771428534119e-09 0.000357066844981935 0.0575868785731211 pink

BPHL 0.0806392516547013 -0.0806392516547013 0.133353002438669 0.145137998899279 0.0541685289903397 0.408981476254133 0.408981476254133 0.170894088104972 0.135805237050833 0.57947657830255 yellow

BPIFA1 -0.229273165229873 0.229273165229873 -0.192400502035777 -0.0577860390081959 0.179009852570217 0.017525219423735 0.017525219423735 0.0470976184085561 0.554375042169985 0.0650557258638859 turquoise

BPIFA2 -0.162566934379627 0.162566934379627 -0.32123912220446 -0.238638905639549 0.0577642929712999 0.094326752863888 0.094326752863888 0.000741439089978639 0.0133094218854932 0.554524355418974 turquoise

BPIFA3 -0.115588634027896 0.115588634027896 -0.221156158670689 -0.165254634988147 0.0381320372267613 0.235782998721689 0.235782998721689 0.0220670849458546 0.0889343728732348 0.696573647690652 turquoise

BPIFA4P -0.116212478332926 0.116212478332926 -0.0901799321465545 -0.021165879623215 0.0939429751750754 0.233247299893776 0.233247299893776 0.355613432132827 0.828680477370131 0.335813296641082 green

BPIFB1 -0.242755023856155 0.242755023856155 -0.453352365076803 -0.337960520445969 0.0795213770966372 0.0117558646776513 0.0117558646776513 9.40795221056927e-07 0.000370597530869341 0.415527838805031 turquoise

BPIFB2 -0.18831440217933 0.18831440217933 -0.239948415083563 -0.131431785935075 0.122557535117384 0.0520794834798806 0.0520794834798806 0.0127968239949328 0.177197530259792 0.208535884254226 turquoise

BPIFB4 -0.176437034552319 0.176437034552319 -0.264646477346992 -0.141279340879383 0.141415582900999 0.0690744876075242 0.0690744876075242 0.0058752015196371 0.146631985632164 0.146238950311251 turquoise

BPIFB6 -0.0774389206849938 0.0774389206849938 -0.0309298980044108 -0.0249280556938314 0.00225225971245934 0.42788422242444 0.42788422242444 0.751805808343131 0.798824478768998 0.981631176419528 turquoise

BPIFC -0.0903388010590359 0.0903388010590359 -0.264960468575891 -0.201650633667169 0.0394700433436856 0.354763113491843 0.354763113491843 0.00581461387433864 0.0372660231533543 0.68647512468977 turquoise

BPNT1 -0.3703752671942 0.3703752671942 -0.486417940512884 -0.375693505690924 0.0631298010074715 8.60856427792891e-05 8.60856427792891e-05 1.08510604168546e-07 6.67280670772187e-05 0.518282914561935 brown

BPTF 0.478346520555958 -0.478346520555958 0.516946749836179 0.202515912719313 -0.400817910678984 1.87769693243388e-07 1.87769693243388e-07 1.19410207493282e-08 0.0364416837920109 1.88416903561785e-05 blue

BPY2 0.0733575668662057 -0.0733575668662057 0.048299595871127 -0.00630375478183553 -0.0802348300756497 0.452702957631498 0.452702957631498 0.621281345202519 0.948618849695631 0.411342786251033 green

BRAF -0.45973504883277 0.45973504883277 -0.424378836011997 -0.329971383803102 0.0513552382928322 6.30985648595628e-07 6.30985648595628e-07 5.22656096894081e-06 0.000518707069955125 0.599353748485342 brown

BRAP -0.200528999238547 0.200528999238547 0.056803933895696 0.146419728595688 0.166559558165311 0.0383578210052233 0.0383578210052233 0.561137661257664 0.132346930142872 0.0864060772253526 brown

BRAT1 0.278329202290651 -0.278329202290651 0.2682175409022 0.208814771648599 -0.0320082781010887 0.00369803366827824 0.00369803366827824 0.00521812482973413 0.0308904493327993 0.743447654964216 grey

BRCA1 -0.46272260388384 0.46272260388384 -0.3523406813131 -0.1717530344294 0.215992254542973 5.21920754622978e-07 5.21920754622978e-07 0.00019770879406395 0.0769056269123555 0.0254538639991163 magenta

BRCA2 -0.300689415128447 0.300689415128447 -0.428367087268404 -0.303179073932711 0.102540905185297 0.00164962908285787 0.00164962908285787 4.16611304422988e-06 0.00150180204948275 0.293255675332489 magenta

BRCC3 -0.010590997896472 0.010590997896472 0.101234547299816 0.0199278241756115 -0.111959567715916 0.913781209829984 0.913781209829984 0.299481026812031 0.838561312747753 0.250914904271655 yellow

BRD1 0.780509870841467 -0.780509870841467 0.66823228918073 0.363324569387532 -0.345889570399527 3.74312026832818e-23 3.74312026832818e-23 3.7389263861941e-15 0.000119854917644583 0.000263062999216313 blue

BRD2 0.673423713955745 -0.673423713955745 0.638423489030422 0.366197635694719 -0.298097063684243 1.90881768335287e-15 1.90881768335287e-15 1.38794475685119e-13 0.000104831104604059 0.00181746555727958 blue

BRD3 0.703223171891957 -0.703223171891957 0.567567926875169 0.349918673688601 -0.223688902326552 3.04434182724813e-17 3.04434182724813e-17 1.83317249055947e-10 0.000220244464774024 0.020552325413091 brown

BRD4 0.52960105519236 -0.52960105519236 0.211920386389449 -0.00248192036910122 -0.309337407266225 4.48061451006082e-09 4.48061451006082e-09 0.0284281350872511 0.979758503735778 0.00118638496795464 brown

BRD7 0.397166653362521 -0.397166653362521 0.489687611501711 0.36344212970106 -0.0886175195795649 2.27888979133221e-05 2.27888979133221e-05 8.65448638353199e-08 0.000119202831795145 0.364043364074254 blue

BRD7P3 -0.0937155920349049 0.0937155920349049 -0.192991428633808 -0.0882970937081674 0.128109922481057 0.336989484711343 0.336989484711343 0.0464109767459572 0.365787303967204 0.188496355432414 turquoise

BRD8 -0.337208852067883 0.337208852067883 -0.315270448427135 -0.242039180214523 0.0434031505565385 0.000382656253245899 0.000382656253245899 0.000940804635247068 0.0120140789122706 0.657113702449767 brown

BRD9 0.656158567881116 -0.656158567881116 0.512341552004845 0.283272550177038 -0.257213332482005 1.69724335433594e-14 1.69724335433594e-14 1.68926712210286e-08 0.00311063332157496 0.00748314692223335 blue

BRDT 0.0586996134588821 -0.0586996134588821 0.0239681878285695 0.0666849555087801 0.0785964977407493 0.548119808636901 0.548119808636901 0.806416161285621 0.494952705042112 0.420989828615191 yellow

BRE 0.00439637466264463 -0.00439637466264463 -0.305359219181019 -0.268883661639168 -0.016398994170181 0.964153124446835 0.964153124446835 0.00138234442101401 0.00510307075273918 0.866857567713182 grey

BRE-AS1 -0.160843198142591 0.160843198142591 -0.135541919855953 0.0407447707109365 0.264264804958567 0.0979189202693666 0.0979189202693666 0.163915850986072 0.676905207326396 0.00594960366004553 grey

BREA2 -0.126617849690606 0.126617849690606 -0.03179978713565 0.0811188712283536 0.183374338756238 0.193738445749127 0.193738445749127 0.745061309179881 0.406191437211746 0.0586744912618511 grey

BRF1 0.217083657586054 -0.217083657586054 0.0424704991417054 0.0401538593504012 0.00695628679070814 0.0247032930167814 0.0247032930167814 0.664031168811238 0.681335150764667 0.943308555928249 green

BRF2 0.540245167049943 -0.540245167049943 0.415243371827881 0.268352618917186 -0.142715330678747 1.90311906787887e-09 1.90311906787887e-09 8.69242975556916e-06 0.00519460882667601 0.142529252924215 blue

BRI3 -0.640320819905434 0.640320819905434 -0.323634553836839 -0.012637777220784 0.444540991110144 1.1157558236396e-13 1.1157558236396e-13 0.000672931969332275 0.897202652113528 1.61167089900164e-06 brown

BRI3BP 0.375361083481014 -0.375361083481014 0.379315757382667 0.137387982985129 -0.31311927743272 6.78077646894224e-05 6.78077646894224e-05 5.59576438182352e-05 0.158197010495275 0.00102390207070531 turquoise

BRICD5 0.20218392554551 -0.20218392554551 0.236881383269324 0.128533282586349 -0.123057762961184 0.0367561342621642 0.0367561342621642 0.0140254531386826 0.187027923065269 0.206670453897674 brown

BRINP1 -0.128807830726128 0.128807830726128 -0.217268052615591 -0.182837897389551 0.00271027760817557 0.186080109431196 0.186080109431196 0.024578366816338 0.0594300627119539 0.977896568219286 turquoise

BRINP2 0.0570264264265956 -0.0570264264265956 -0.151775217032742 -0.142797317254548 -0.0236736766450238 0.559602171585171 0.559602171585171 0.118620430323578 0.142297659350314 0.808749121554716 green

BRINP3 -0.0823874046294746 0.0823874046294746 -0.101757104508636 -0.0297222717841614 0.0960992615751678 0.398866331696678 0.398866331696678 0.296980469968642 0.761199834614832 0.324789153760251 turquoise

BRIP1 -0.252673452049663 0.252673452049663 -0.18729188007549 -0.126924946649087 0.0543856026293564 0.00864642320576088 0.00864642320576088 0.0533919137759802 0.192650993233998 0.577955611456409 magenta

BRIX1 0.0325338763404422 -0.0325338763404422 0.195784057599122 0.0959712987266537 -0.119114347194469 0.739384631139332 0.739384631139332 0.0432774313012274 0.325436817161164 0.221702956536651 yellow

BRK1 -0.468400191527488 0.468400191527488 -0.501077207079171 -0.322043412858546 0.175234168057431 3.620850762329e-07 3.620850762329e-07 3.86315886994273e-08 0.000717756014478616 0.0710211371832255 brown

BRMS1 -0.193663349823703 0.193663349823703 -0.22137292704489 -0.0990208744920075 0.15078531616906 0.0456403342237873 0.0456403342237873 0.021933799899105 0.310227090936535 0.121071078829729 grey

BRMS1L 0.165837385481846 -0.165837385481846 0.157643960757205 0.0121472503900232 -0.206376007759874 0.0877981248292814 0.0877981248292814 0.104870619004136 0.901172079700764 0.0329484059463164 yellow

BROX -0.518859151987195 0.518859151987195 -0.418215422430621 -0.194539758668365 0.272190919673876 1.03232870819838e-08 1.03232870819838e-08 7.37858228106059e-06 0.0446511588347558 0.00456463542582097 brown

BRPF1 0.588234610484711 -0.588234610484711 0.693453479220631 0.498721945470224 -0.152551803855805 2.69483437159705e-11 2.69483437159705e-11 1.24931781039906e-16 4.57541976621534e-08 0.116724847852194 blue

BRPF3 -0.213958136112772 0.213958136112772 -0.109188063310384 -0.0757029224010445 0.0288092779557358 0.0269047194613256 0.0269047194613256 0.262911911710905 0.438344098964037 0.768325125633746 turquoise

BRS3 -0.136955057463331 0.136955057463331 -0.247894730964955 -0.164058077200181 0.0786602947691876 0.159524595048855 0.159524595048855 0.0100402317289995 0.0913040135065666 0.420611738964372 turquoise

BRSK1 0.00624770432775547 -0.00624770432775547 0.0288822869193404 0.112559872042012 0.149330970518835 0.949075095535723 0.949075095535723 0.767754621626847 0.248366799129908 0.124742108861185 green

BRSK2 -0.137420754380663 0.137420754380663 -0.361929097460171 -0.27333466450555 0.0575020642222776 0.158096852375133 0.158096852375133 0.000127852767137282 0.00439059024564623 0.556326402659454 turquoise

BRWD1 0.323789156263466 -0.323789156263466 0.495672810704914 0.31626936384879 -0.177246373755018 0.000668716295229488 0.000668716295229488 5.68526366004224e-08 0.000904351938565894 0.0677891810762527 blue

BRWD1-IT2 -0.183066968864517 0.183066968864517 -0.322953371542272 -0.285565964656415 -0.019361946645072 0.0591064504001196 0.0591064504001196 0.000691797896204788 0.00286775530452957 0.843086007826756 turquoise

BRWD3 -0.0499782185575926 0.0499782185575926 0.128881308380738 0.163935862908044 0.0924906287011676 0.609192677739376 0.609192677739376 0.185827039531187 0.0915488337675229 0.343370710905932 tan

BSDC1 0.145049583463208 -0.145049583463208 -0.0903150333387623 -0.189619256978747 -0.191581634377982 0.136046307875259 0.136046307875259 0.354890245595008 0.0504434122818697 0.0480630244400912 grey

BSG -0.103450125698847 0.103450125698847 -0.33113373736418 -0.299299929178887 -0.0308783214037457 0.288973734739389 0.288973734739389 0.000494220953379775 0.00173775439492637 0.752206289489949 pink

BSN -0.0068055633543212 0.0068055633543212 -0.0818785501547913 -0.00707585685052523 0.10588899385474 0.944534928526992 0.944534928526992 0.401795232584069 0.942335759192483 0.277693950196541 yellow

BSN-AS2 -0.38496389827205 0.38496389827205 -0.344562529236578 -0.162814272524808 0.219954454091383 4.2349130663187e-05 4.2349130663187e-05 0.000278770075614053 0.0938199680756726 0.0228186433534955 blue

BSND -0.225490413607668 0.225490413607668 -0.352074480771995 -0.178282124838505 0.204534811773604 0.0195300743448896 0.0195300743448896 0.000200076849450314 0.0661726685497864 0.0345776977931431 turquoise

BSPRY -0.336769938450602 0.336769938450602 -0.285010913417661 -0.171006226416254 0.120315989930183 0.000389863661329923 0.000389863661329923 0.00292491715320459 0.0782176920981346 0.217042671237099 turquoise

BST1 -0.773388693198992 0.773388693198992 -0.632943097259062 -0.336106707115816 0.341244477520017 1.63574841842483e-22 1.63574841842483e-22 2.5855479159304e-13 0.000400992065524066 0.000321910295524563 brown

BST2 0.174376276681139 -0.174376276681139 0.263431026533303 0.406961463745077 0.310966325972736 0.0724363982345592 0.0724363982345592 0.00611505570963694 1.36121933963158e-05 0.00111372037895624 cyan

BTAF1 0.23485035680537 -0.23485035680537 0.256283649558923 0.145020630760339 -0.123028886129709 0.0148944432003644 0.0148944432003644 0.00770941900480619 0.136125320165165 0.206777814430076 yellow

BTBD1 0.147877285016217 -0.147877285016217 0.199851229664159 0.171312580560627 0.00281840776337217 0.128496373022806 0.128496373022806 0.0390304591593215 0.0776773030007924 0.977014958066436 yellow

BTBD10 -0.531021729714365 0.531021729714365 -0.409955922647964 -0.185303013149239 0.2759654441431 4.00355397036616e-09 4.00355397036616e-09 1.15903014884051e-05 0.0560226611682136 0.00401256432176911 brown

BTBD11 0.871460302047522 -0.871460302047522 0.653866055087845 0.302406669287693 -0.42852953261836 2.90219612245145e-34 2.90219612245145e-34 2.24456801345346e-14 0.00154632380013335 4.12755708698057e-06 blue

BTBD16 -0.308183367845533 0.308183367845533 -0.313225750437573 -0.176385274592236 0.151816851692056 0.00124045030568312 0.00124045030568312 0.00101963659817418 0.0691573540143895 0.118518203844179 turquoise

BTBD17 0.0818406299155582 -0.0818406299155582 -0.0604051555179841 -0.07450508008345 -0.0393977452692357 0.402014004635366 0.402014004635366 0.536534773742295 0.445645113352136 0.687019402013249 turquoise

BTBD18 0.54451705965153 -0.54451705965153 0.404316099621894 0.212311790831277 -0.222034663887074 1.33828026493953e-09 1.33828026493953e-09 1.56698698202781e-05 0.0281299680448469 0.021531177746487 blue

BTBD19 -0.686012967985253 0.686012967985253 -0.470627625109241 -0.0845626831354956 0.534190818591941 3.52848210796115e-16 3.52848210796115e-16 3.13130981469115e-07 0.386489252953181 3.10860632969768e-09 blue

BTBD2 0.477490449305785 -0.477490449305785 0.294015875164917 0.12926061218128 -0.204087640749384 1.98850843608725e-07 1.98850843608725e-07 0.00211320778784294 0.184524639042898 0.0349834836344561 brown

BTBD3 -0.298725233963228 0.298725233963228 -0.462348768036501 -0.306968854655271 0.145040529648557 0.0017754342048548 0.0017754342048548 5.34515223094212e-07 0.0012997621574975 0.136071012097975 yellow

BTBD6 0.389628119482364 -0.389628119482364 0.139714113503351 -0.0979306843915445 -0.367266899210903 3.35141826454306e-05 3.35141826454306e-05 0.151204701471183 0.315610395575097 9.97016852620788e-05 red

BTBD7 -0.133998292031874 0.133998292031874 -0.116197862362944 -0.224639307391098 -0.213713558420134 0.168814501351584 0.168814501351584 0.233306489559162 0.0200074378539855 0.027083816161974 brown

BTBD8 -0.299947023966348 0.299947023966348 -0.334427588123595 -0.273566910986926 0.0175108597545346 0.00169619622916095 0.00169619622916095 0.000430486513584378 0.00435598184346135 0.857922039589527 grey

BTBD9 0.0701078887293349 -0.0701078887293349 0.00141549887382186 -0.0511363329931381 -0.0887720243670661 0.47302407042732 0.47302407042732 0.988454964234874 0.60091309692853 0.363204295257559 yellow

BTC -0.364533819467499 0.364533819467499 -0.408052977313751 -0.185267767061868 0.273285325505906 0.000113302939293039 0.000113302939293039 1.2839573408475e-05 0.0560702249139817 0.00439797409488299 blue

BTD 0.250746107973936 -0.250746107973936 0.109397579175975 -0.0504387532832037 -0.243063855784939 0.00918661670930716 0.00918661670930716 0.261991574225911 0.605894235350506 0.0116459714944948 blue

BTF3 0.126617347029581 -0.126617347029581 -0.0530718083210744 -0.182468177401922 -0.233076113482502 0.193740229342041 0.193740229342041 0.587189111389513 0.0599554265944153 0.0156914525491737 grey

BTF3L4 0.27311889322684 -0.27311889322684 0.31491649329621 0.287367432069282 0.0339891693218196 0.00442296347859335 0.00442296347859335 0.000954039814229459 0.00268909051123419 0.728172463465549 yellow

BTF3P11 0.179058581083642 -0.179058581083642 0.031637431459202 -0.0545999929208664 -0.138161093424186 0.0649814864825098 0.0649814864825098 0.746318655358608 0.576455265558794 0.155846773789717 brown

BTG1 0.442814572403917 -0.442814572403917 0.73869979609269 0.541617503392399 -0.144941983691546 1.78780360995208e-06 1.78780360995208e-06 1.07190082404239e-19 1.70048997517786e-09 0.136340125250948 black

BTG2 0.537338579942654 -0.537338579942654 0.513701737262014 0.215747037306437 -0.3737039579403 2.41155355778972e-09 2.41155355778972e-09 1.52557224766804e-08 0.0256251528883034 7.34373388587607e-05 blue

BTG3 0.0490598153834554 -0.0490598153834554 -0.0700246147818713 -0.174493502998245 -0.195140990366491 0.615793844676678 0.615793844676678 0.473551224621576 0.0722416784642662 0.0439829281807464 red

BTG4 -0.152549897106308 0.152549897106308 -0.385214363207919 -0.292993147200057 0.0576853600807028 0.116729473175914 0.116729473175914 4.18241065012351e-05 0.00219383136267683 0.555066489490155 turquoise

BTK -0.256655220514072 0.256655220514072 -0.162573587096847 0.0126907866296141 0.255602370830845 0.00761826872785558 0.00761826872785558 0.094313093496435 0.896773840850097 0.00787904876868352 brown

BTLA 0.329761490265622 -0.329761490265622 0.321535038258314 0.0686358044034573 -0.346538011815515 0.000523245698657011 0.000523245698657011 0.000732643632772375 0.482389664912228 0.000255689103207937 turquoise

BTN1A1 -0.402371538158857 0.402371538158857 -0.365492275904586 -0.267937715905446 0.0717863329211062 1.73650777126264e-05 1.73650777126264e-05 0.000108348045173426 0.00526714193867318 0.462467063690802 blue

BTN2A1 0.0982224266193937 -0.0982224266193937 0.506802426881776 0.543443165632009 0.192045651325589 0.314163896392541 0.314163896392541 2.54669021844637e-08 1.4628180459123e-09 0.047513978227306 tan

BTN2A2 0.434862164050791 -0.434862164050791 0.738666566537626 0.622970412620358 -0.00690887082822252 2.86182241326496e-06 2.86182241326496e-06 1.07804548975315e-19 7.77458358400026e-13 0.943694345336787 blue

BTN2A3P 0.0201473906118363 -0.0201473906118363 -0.15866004390835 -0.110940014138767 0.0402734365034056 0.836807090509386 0.836807090509386 0.102622112065804 0.255283683536718 0.6804378243813 turquoise

BTN3A1 0.539975327932346 -0.539975327932346 0.821837272814007 0.733056537912017 0.0600606262110202 1.94560047047711e-09 1.94560047047711e-09 2.08081672238351e-27 2.79529038211895e-19 0.538865196399923 blue

BTN3A2 0.560075072376731 -0.560075072376731 0.711319921401872 0.576735086332849 -0.0459557623867737 3.55716823620043e-10 3.55716823620043e-10 9.03939526373237e-18 7.96182747965585e-11 0.638329939837711 blue

BTN3A3 0.559446938846373 -0.559446938846373 0.897153153972585 0.754709406524538 -0.0116547636653835 3.75763811415271e-10 3.75763811415271e-10 4.75190994421981e-39 6.14519911252334e-21 0.905159818846366 blue

BTNL2 -0.0959962963140811 0.0959962963140811 -0.16191727423749 -0.14732895494549 -0.0167571890188092 0.325310230858338 0.325310230858338 0.0956681819582529 0.129934748978036 0.863977040467666 turquoise

BTNL3 0.00744914611599096 -0.00744914611599096 0.0348119485653595 0.0318887081019087 0.00396444970354898 0.939299308560585 0.939299308560585 0.721858542408798 0.744372953662513 0.967672922727131 yellow

BTNL8 0.123593702094998 -0.123593702094998 0.13547211846909 0.166689234127806 0.0876710800068862 0.204685132824596 0.204685132824596 0.164135058997757 0.086157986898446 0.369209223430434 grey

BTNL9 -0.0256724007687276 0.0256724007687276 -0.252348245542906 -0.26919836003284 -0.0932592112308645 0.792950223116751 0.792950223116751 0.00873556066410024 0.00504950551778702 0.339358079971149 turquoise

BTRC 0.0757687230925912 -0.0757687230925912 -0.344538472589957 -0.366037044974262 -0.124772823578065 0.437945008473468 0.437945008473468 0.000279062644450955 0.000105622349706862 0.200365427843631 black

BUB1 -0.510023648784966 0.510023648784966 -0.639506284147011 -0.498673502580846 0.0749595918655462 2.0076444144694e-08 2.0076444144694e-08 1.22560165740186e-13 4.59131027172032e-08 0.442866793397259 magenta

BUB1B -0.423864979677606 0.423864979677606 -0.554561388906637 -0.441603861125266 0.0494516427925207 5.38039694718693e-06 5.38039694718693e-06 5.73360731281168e-10 1.92203394953538e-06 0.612973733683314 magenta

BUB3 0.614970781844746 -0.614970781844746 0.542588978814184 0.21369308859985 -0.418780215958645 1.82844268196639e-12 1.82844268196639e-12 1.56975035294108e-09 0.0270988514674368 7.1510850145076e-06 turquoise

BUD13 0.353892757486771 -0.353892757486771 0.221407732672091 0.18197670437979 -0.0101313882882529 0.000184410880266905 0.000184410880266905 0.021912463304997 0.0606596695115888 0.917509315111594 turquoise

BUD31 -0.311918164048685 0.311918164048685 -0.498882637521297 -0.359553956632207 0.10845158637509 0.00107316077901717 0.00107316077901717 4.5230840033099e-08 0.000142614773138458 0.266164467505308 greenyellow

BVES -0.0990378900447041 0.0990378900447041 -0.346327585763221 -0.281402919430995 0.0213538362970158 0.310143545406136 0.310143545406136 0.0002580608066401 0.00332213892497766 0.827182700911039 turquoise

BVES-AS1 -0.0728841212773729 0.0728841212773729 -0.106116691507059 -0.0402956878361889 0.0844424185798276 0.455632943372501 0.455632943372501 0.276656142507479 0.680270895918251 0.387167449745338 turquoise

BX648501 -0.170635339714415 0.170635339714415 -0.130402885800599 -0.0133553847899767 0.165104540852711 0.0788759442555702 0.0788759442555702 0.1806427013067 0.891400240331212 0.0892289097990528 turquoise

BYSL 0.170014307654371 -0.170014307654371 0.0673951433496746 -0.0316710177687304 -0.150755267328757 0.0799880949499557 0.0799880949499557 0.490359402454825 0.746058495032578 0.121146075183141 red

BZRAP1 0.527558571824915 -0.527558571824915 0.426920887494955 0.259557791610679 -0.174446078835515 5.26301675680592e-09 5.26301675680592e-09 4.52467961892054e-06 0.00693840010943678 0.0723204018287521 brown

BZRAP1-AS1 0.78646617752974 -0.78646617752974 0.592894867084779 0.233366279241028 -0.457843437375187 1.04445509710146e-23 1.04445509710146e-23 1.71615854793296e-11 0.015558631087327 7.10884157474569e-07 blue

BZW1 -0.0308605377219875 0.0308605377219875 -0.014459838559161 -0.0241199921155683 -0.0200910919104593 0.75234439118335 0.75234439118335 0.882481172129746 0.8052143102001 0.83725681213078 yellow

BZW2 0.459940262977997 -0.459940262977997 0.395166125518248 0.131882463720352 -0.345279053831107 6.22849905212775e-07 6.22849905212775e-07 2.52680751807724e-05 0.175703767359261 0.000270184736345211 turquoise

C10orf10 -0.313341424113353 0.313341424113353 -0.59134593835242 -0.499611442300615 0.0040263918088477 0.00101502090830281 0.00101502090830281 1.99544956726704e-11 4.2928146442077e-08 0.967168110803275 pink

C10orf107 -0.0832311360432443 0.0832311360432443 -0.0643611394726967 -0.0455863462483121 0.0153481711139397 0.394037934049171 0.394037934049171 0.510139496408495 0.641034585176741 0.875317988276659 grey

C10orf11 -0.353934102236497 0.353934102236497 -0.269245551288029 -0.0466166807563216 0.308597758084743 0.000184068251348584 0.000184068251348584 0.00504151637466711 0.633502922271813 0.00122078381990976 greenyellow

C10orf113 0.0533676488676299 -0.0533676488676299 0.108121854751964 0.192310076758603 0.170506966419871 0.585104045351769 0.585104045351769 0.26762949430309 0.0472034295570591 0.0791048140761803 grey

C10orf12 -0.162044667890103 0.162044667890103 -0.0947181919917743 -0.0280598933884856 0.088784109347175 0.0954039576964485 0.0954039576964485 0.331822927416923 0.774188039876344 0.363138715722213 grey

C10orf126 -0.0139601816348883 0.0139601816348883 -0.216699330991433 -0.212692816793075 -0.0487464907383568 0.886514424671369 0.886514424671369 0.0249654146142716 0.0278422630863415 0.618052978463173 turquoise

C10orf128 0.110556858777611 -0.110556858777611 0.356790023601145 0.351384249917251 0.0822796490360018 0.256938895411948 0.256938895411948 0.000161778790998451 0.000206339851375834 0.399485499415788 cyan

C10orf2 0.489826868679256 -0.489826868679256 0.3535420336623 0.0375692069625075 -0.445315453193617 8.5710652532958e-08 8.5710652532958e-08 0.00018734122697103 0.700837590070443 1.53811824458384e-06 blue

C10orf25 0.182057920499704 -0.182057920499704 0.106914016393508 0.135548609109482 0.075971097205419 0.0605428292931085 0.0605428292931085 0.273042626943359 0.163894855118601 0.436718872724989 brown

C10orf32 0.218871623784399 -0.218871623784399 0.363327265672631 0.292813470546554 -0.0264765528359028 0.0235145245018221 0.0235145245018221 0.000119839924770293 0.00220827919765602 0.786616977832906 yellow

C10orf35 0.353709670932171 -0.353709670932171 0.148186041232942 0.0571997763731855 -0.116343203863548 0.000185935254173324 0.000185935254173324 0.127691831139833 0.558407224207192 0.232718373033452 brown

C10orf40 -0.114172971640031 0.114172971640031 -0.159250412579481 -0.122455519402128 0.0215916901931763 0.241608279837112 0.241608279837112 0.101333143113784 0.208917789321762 0.825288195276633 turquoise

C10orf53 -0.426729466468761 0.426729466468761 -0.413351773540253 -0.157854046922742 0.327411931695569 4.5742671416339e-06 4.5742671416339e-06 9.64021021834358e-06 0.10440258256295 0.000576601055953754 turquoise

C10orf54 -0.447233483706919 0.447233483706919 -0.111956895424271 0.147710709902961 0.411734913247144 1.3694070159068e-06 1.3694070159068e-06 0.250926287375697 0.128932035930587 1.05266229133642e-05 purple

C10orf55 -0.101075268586415 0.101075268586415 -0.225216356446917 -0.202620917256112 -0.0194002087148265 0.300245957085829 0.300245957085829 0.0196827040567141 0.036342697515097 0.842779905766172 turquoise

C10orf62 -0.236626207831342 0.236626207831342 -0.301987379929849 -0.179308466717686 0.130677476344828 0.0141321513940771 0.0141321513940771 0.00157099032029604 0.0646018596915868 0.179718502089609 turquoise

C10orf67 -0.308365009066042 0.308365009066042 -0.390288974106875 -0.266156460598497 0.110510455212738 0.00123179459722776 0.00123179459722776 3.24120734871217e-05 0.00558888901573781 0.257139853796292 yellow

C10orf71 0.150003293847799 -0.150003293847799 -0.0829926435457785 -0.101399777435868 -0.0524926990036288 0.123034549894126 0.123034549894126 0.395399200354993 0.298688870312746 0.591280421980868 green

C10orf71-AS1 0.0202075640937622 -0.0202075640937622 -0.143343608037354 -0.108072980477648 0.0230833554703691 0.83632647473167 0.83632647473167 0.140761776352849 0.267847111027091 0.813430359943278 green

C10orf76 0.606675243898944 -0.606675243898944 0.356148014662798 0.142996778818598 -0.270248547519115 4.32821122422028e-12 4.32821122422028e-12 0.000166559472063609 0.141735414817022 0.00487435865668129 blue

C10orf82 -0.00331074427127642 0.00331074427127642 -0.344199954070228 -0.360439837587468 -0.115766593683514 0.973001190437319 0.973001190437319 0.000283209776598268 0.000136933057457589 0.235057706227219 green

C10orf85 -0.208485969200516 0.208485969200516 -0.183891304462541 -0.0647017001589469 0.155028276453237 0.0311614028395899 0.0311614028395899 0.0579538082345469 0.507898913125523 0.110836350972475 turquoise

C10orf88 0.431797256430415 -0.431797256430415 0.2395137428364 -0.00709409523773679 -0.356889826039056 3.41996074189122e-06 3.41996074189122e-06 0.0129650305534554 0.942187382945012 0.000161047149644964 turquoise

C10orf90 -0.0698191123230483 0.0698191123230483 -0.331993846320995 -0.318883079275915 -0.0628555007716813 0.474853490939903 0.474853490939903 0.000476790765770145 0.000814991545796845 0.520105968989301 turquoise

C10orf91 0.123476915493715 -0.123476915493715 -0.0665701787823942 -0.0719460935589399 -0.0261807973067124 0.205116585304703 0.205116585304703 0.495697171029208 0.461469006550902 0.788944660807749 grey

C10orf95 0.24569535102935 -0.24569535102935 0.0863539056858752 0.0250016141754695 -0.0819282497130129 0.0107454579323792 0.0107454579323792 0.376472971454219 0.79824346083564 0.401508609113556 green

C10orf99 -0.10784404090843 0.10784404090843 -0.0954705767451675 -0.139884054674849 -0.0998011005562087 0.26886807871424 0.26886807871424 0.327979093531549 0.150703107998273 0.30641127791029 grey

C11orf1 0.228509331461811 -0.228509331461811 0.356935025386246 0.146985790351941 -0.264615821515107 0.017915073974562 0.017915073974562 0.000160716808471933 0.130841183201487 0.00588114680482378 turquoise

C11orf16 0.229092064388421 -0.229092064388421 -0.0119555345548147 -0.106175964736618 -0.162874572411556 0.017616982685213 0.017616982685213 0.902724145041769 0.276386413081827 0.0936967432124748 green

C11orf21 0.380237466428031 -0.380237466428031 0.430509340305149 0.286641423245229 -0.133675428344539 5.34887325945268e-05 5.34887325945268e-05 3.68393656615975e-06 0.00275985171853938 0.169852648566031 blue

C11orf24 0.360207500018125 -0.360207500018125 0.205389984342088 0.00319737088050662 -0.290301958831885 0.000138402499202785 0.000138402499202785 0.0338127015901809 0.973925409601919 0.00241942658182061 red

C11orf30 0.457371491657491 -0.457371491657491 0.45191485469132 0.237980597698874 -0.247030761676021 7.32266342469712e-07 7.32266342469712e-07 1.02821203546037e-06 0.0135738027966728 0.0103122638052622 turquoise

C11orf31 0.154403857692445 -0.154403857692445 0.350812951537666 0.306532226610512 0.0148106669924136 0.112298816401153 0.112298816401153 0.000211660434336693 0.00132170792553965 0.879651056919022 yellow

C11orf40 -0.0638170847906771 0.0638170847906771 -0.105210440467461 0.0125630789021761 0.172792994569275 0.5137294317415 0.5137294317415 0.280802162942071 0.89780696260286 0.075108041029594 turquoise

C11orf42 0.108020044123008 -0.108020044123008 -0.183887612203225 -0.190924046709634 -0.0590667852768464 0.268082949675755 0.268082949675755 0.0579589296517615 0.0488501022657087 0.545615508080083 yellow

C11orf44 -0.302980851501054 0.302980851501054 -0.273249165701162 -0.138262625851825 0.158918676847656 0.00151311526754995 0.00151311526754995 0.00440339264790992 0.155540068058178 0.102055857889634 turquoise
[truncated: 4,033,416 more chars]
