# Supplementary material for: Identification of a novel four-gene diagnostic signature for patients with sepsis by integrating weighted gene co-expression network analysis and support vector machine algorithm
Source: Hereditas. 2022 Feb 21;159:14. doi: 10.1186/s41065-021-00215-8 (PMC8859894; doi:10.1186/s41065-021-00215-8)
Supplement: Supplementary file 3 — Additional file 3. [file 41065_2021_215_MOESM3_ESM.docx]

RBX1

ALPK1

RP11-373D23.2

SEMA4A

FKBP9

HIST1H2AE

ARL4A

NRN1

HIST1H4H

ALOX5AP

CLIC2

NQO2

FTX

ROPN1L

MAOA

CTNNAL1

C9orf84

C8orf88

YOD1

BC043356

HIST1H1C

MKNK1

AP5B1

EIF1AY

SLC26A8

VSIG4

XIST

CCR3

LRRN3

GNLY

NELL2

CD247

IL2RB

KLRF1

ITK

TGFBR3

FAIM3

HLA-DQA1

TRBC1

TXK

FGFBP2

LBH

SGK223

PRF1

HLA-DPA1

TRAC

CCR7

IL7R

LCK

NLRC3

RASGRP1

CLIC3

FCER1A

GPR18

KLRB1

EOMES

CD2

CD3E

GPRASP1

PCED1B

PVRIG

PLEKHA1

BCL11B

EVL

TBC1D4

TRIB2

UBASH3A

GZMK

ESYT1

BACH2

NOG

CHRM3-AS2

MARCKSL1

NR1D2

CACNA2D3

MS4A1

HLA-DMB

PRKX

CD8A

GZMB

GPR183

CTSW

CD160

CECR1

CDC25B

MAL

TCL1A

GATA3

NMT2

LEF1

ABLIM1

DDHD2

TGFBI

DYRK2

SGK1

SH2D1B

LGALS2

AL833181

FYN

PRKCQ

TCF7

HLA-DMA

PPP1R16B

THEMIS

CD7

FLT3LG

LINC01215

CD3G

ZAP70

MYBL1

ANKRD36B

SPOCK2

CD27

GZMA

STAT4

TRG-AS1

TSHZ1

MPEG1

TRAT1

SIDT1

P2RY10

RTN1

ARL4C

TBX21

IL32

SYTL2

ZNF600

CX3CR1

LDLRAP1

RUNX3

CCR6

TRDV3

LDOC1L

ABHD14B

MAN1C1

KDM2B

FCRL3

LOC93622

RCAN3

LY9

SEPT1

BZRAP1-AS1

DPP4

PASK

CAMK4

CXXC5

SEPT9

BTN3A3

BTBD11

SKAP1

ALDH1A1

USP11

HLA-DRA

ZC3H12D

KLRD1

PYHIN1

MAP3K14

SLFN5

GPR171

CD81

MYC

ATP6V0E2

MSANTD2

DNMT1

PTPN4

RP11-732A19.1

ID3

KIAA0355

LAT

IL10RA

LOC102724356

CCDC92

LPIN1

ETS1

RP11-285F7.2

BTN3A2

DENND2D

THEM4

BCL9L

LOC283588

DOCK10

HLA-DPB1

APOL3

GZMH

CD3D

CD79A

SEPT6

PAQR8

BAG3

KLRG1

FAM169A

FCRLA

SCRN1

TAGAP

KLRC3

TESPA1

C9orf91

PEBP1

GIMAP6

TIAM1

ZNF266

PWAR6

CD96

ECI2

DDX24

DHRS3

ABHD15

PLEKHO1

OXNAD1

RRS1

ATP8B2

IMP3

ZNF121

ATIC

SLAMF6

VPREB3

HVCN1

BANK1

GOLGA8A

GSPT2

GIMAP4

GABPB2

CRY1

RNF125

ZNF83

FAM171A1

ZNF831

CCL4

BCL11A

CTSO

ZBTB4

CXCL8

SATB1

MLLT3

NR3C2

SACS

KLF12

LOC102724611

MAP4K1

TMEM109

PDE4B

TRMT13

SLC38A1

MTR

FTO

SCML4

RUNX1-IT1

PIK3C2B

RHOH

LRBA

CDKN1C

BIN1

SH2D2A

OSBPL3

RSAD1

KIAA1147

PRSS33

TKTL1

ITGB7

EPHX2

PFAS

SIGIRR

PTCD3

SLC39A10

GRAMD1C

PRKACB

LINC00954

HMG20A

SBK1

FLVCR1

RORA

SAMD3

PHOSPHO2

CHMP7

LOC101928054

FAM102A

RRAS2

BEX2

PIK3IP1

ITGA6

STK39

PTER

MEN1

ZNF275

GPR56

GIMAP1

PARP1

MAML2

NFATC2

APBA2

PTPRCAP

ZNF559

CSF1R

CRIP1

PJA1

PEA15

SPIB

C6orf136

TC2N

PLA2G7

ARHGEF18

HNRNPA0

EEF2

CD74

BRD1

CAMK2D

IKZF3

TTC39C

ZFP3

ZNF304

PTGDR

FBLN5

SETD6

MAP3K4

LINC00926

ANKRD46

RP11-158G18.1

KPNA5

CLC

TRIM28

HLA-DQB1

NAA25

ATP2B1

FBXO21

ZHX2

MYOM2

IFFO2

PRKCH
