# Supplementary material for: Identification of a novel four-gene diagnostic signature for patients with sepsis by integrating weighted gene co-expression network analysis and support vector machine algorithm
Source: Hereditas. 2022 Feb 21;159:14. doi: 10.1186/s41065-021-00215-8 (PMC8859894; doi:10.1186/s41065-021-00215-8)
Supplement: Supplementary file 4 — Additional file 4. [file 41065_2021_215_MOESM4_ESM.docx]

ZAK

UBE2F

C9orf72

GK

ALOX5

MTF1

ENTPD1

LOC645984

AZI2

ETS2

CTSD

CASP5

MARCO

LACTB

PRO2852

GNS

GALNT2

CDKN2C

EMILIN2

DIRC2

IL18

NFIL3

AGTPBP1

SIGLEC9

DPH3

HMGB2

PET100

CAPG

LMO2

FAR1

HIST1H2BE

BIK

PNPLA1

ANO10

GSR

CEACAM21

PIWIL4

UBL5

CDC42EP3

C1QC

DDIAS

CORO2A

SGMS2

GK3P

CHMP5

CSF2RA

C2orf76

BAZ1A

PPP4R2

PHTF1

MMADHC

EMB

TIMP1

ZBTB8OS

PDCD10

POR

RP2

PLP2

FAM126B

FLOT2

POMP

FLOT1

CD58

PDZD8

KBTBD7

SIGLEC5

B4GALT5

KIF1B

SH3GLB1

TSPAN2

GCA

SRPK1

H2BFS

CYYR1

ANKRD55

SLC40A1

ANXA1

TMEM167A

FAR2

HIST1H2BH

CPEB4

SPTLC2

LOC100505812

CDK5RAP2

NDUFAF1

CARD16

OAT

LOC101926918

SLC25A40

GLRX

HIST1H4D

WDFY3

GNG10

TMEM260

MCTP1

SNX3

RP11-173M1.8

GMFG

RAB27A

MEF2A

NABP1

CEBPD

LTB4R

MILR1

WIPI1

CKLF

NSUN7

PECR

BLOC1S1

SLC22A15

PPP1R3D

AGPAT9

IFNGR1

DYSF

CTD-2165H16.3

FAM105A

CCPG1

SMPDL3A

NME8

TPST2

QPCT

RP11-330O11.3

EMR1

C1QA

PADI4

VNN2

OSCAR

IDI1

MCTP2

CSTA

EXOC6

ITGAM

HAT1

MANSC1

NDUFA1

NDUFB3

RAB32

ACSL1

GCLM

MAN1A1

LAMTOR5

VMP1

DRAM1

KCNJ2-AS1

UBE2J1

PGM2

ACSL4

CKAP4

VSTM1

ZNF438

CNIH4

PLAC8

JAK2

IL1R1

HIST1H2BD

DKFZp667F0711

mir-223

EMC2

G0S2

NEDD4

PLBD1

WSB1

PYGL

LDHA

ACN9

PCMT1

HGF

CD63

GAS7

SIPA1L2

ENTPD7

AGFG1

CHPT1

LY96

ITGA7

F5

CD55

ERLIN1

STOM

ATP9A

S100A9

TSPO

PDGFC

EXOSC4

LOC441081

DDAH2

SLC37A3

CD59

BCL6

ADAM9

GPR97

CCNA1

LRG1

C1QB

METTL9

ACER3

ZDHHC20

ADM

CLEC4E

CSGALNACT2

LOC101928429

KCNE1

SORT1

ATP11B

ATP6V1C1

PLSCR1

FGD4

MGAM

BST1

CARD6

PLA2G4A

ATP8B4

PGS1

HTATSF1P2

SLC22A4

FAM20A

MAPK14

ARG1

CR1

LILRA5

SLC2A3

LINC00266-1

ASPH

LIN7A

AIM2

CEACAM1

PSTPIP2

KLHL2

TP53I3

SLPI

OPLAH

DPY19L3

FOLR3

GPR160

LRRN1

ECRP

SERPINB1

MAP2K6

FCER1G

PFKFB2

TXN

C3AR1

SLC51A

IL10RB-AS1

RP1-193H18.2

CYP1B1

CD163

RAB13

FCAR

TNFAIP6

GALNT14

CST7

UPP1

FKBP5

ST3GAL4-AS1

NAIP

ORM1

FGF13

FCGR1B

IL18RAP

SULT1B1

UGCG

ST6GALNAC3

HK3

PFKFB3

TLR5

ZDHHC19

PGLYRP1

NLRC4

DHRS9

CYSTM1

BCL2A1

DAAM2

ALPL

METTL7B

CA4

LOC100134822

MS4A4A

PCOLCE2

S100P

DACH1

CLEC5A

GRB10

HPGD

RGL4

BMX

GYG1

ANKRD22

MMP9

IRAK3

IL18R1

SAMSN1

GADD45A

RNASE2

S100A12

CLEC4D

IL1R2

TDRD9

VNN1

GPR84

RETN

ANXA3

OLAH

HP

MCEMP1

CD177

MMP8

A2M-AS1

SULF2

TUBB2A

MAP3K7CL

CPA3

CCL5

NOV

CHI3L1

HSH2D

SSBP3

TIGD3

PDCD4-AS1

EMR3

SIGLEC17P

RPS6KA5

DDX11L2

RP11-44F14.8

CD5

LFNG

CNNM3

AMIGO1

LOC100128751

GSE1

DUSP2

ZNF703

MATK
